# Store‐operated calcium entry controls innate and adaptive immune cell function in inflammatory bowel disease

**DOI:** 10.15252/emmm.202215687

**Published:** 2022-08-02

**Authors:** Marilena Letizia, Yin‐Hu Wang, Ulrike Kaufmann, Lorenz Gerbeth, Annegret Sand, Max Brunkhorst, Patrick Weidner, Jörn Felix Ziegler, Chotima Böttcher, Stephan Schlickeiser, Camila Fernández, Megumi Yamashita, Kenneth Stauderman, Katherine Sun, Désirée Kunkel, Murali Prakriya, IBDome Researchers, Ashley Sanders, Britta Siegmund, Stefan Feske, Carl Weidinger

**Affiliations:** ^1^ Charité – Universitätsmedizin Berlin, Corporate Member of Freie Universität Berlin Humboldt‐Universität zu Berlin and Berlin Institute of Health Berlin Germany; ^2^ Department of Gastroenterology, Infectious Diseases and Rheumatology Campus Benjamin Franklin Berlin Germany; ^3^ Department of Pathology New York University Grossman School of Medicine New York NY USA; ^4^ Berlin Institute for Medical Systems Biology Max Delbrück Center for Molecular Medicine in the Helmholtz Association Berlin Germany; ^5^ Single Cell Approaches for Personalized Medicine Berlin Institute of Health at Charité – Universitätsmedizin Berlin Berlin Germany; ^6^ Experimental and Clinical Research Center, Berlin, A Cooperation of Charité and MDC Berlin Germany; ^7^ Berlin Institute of Health at Charité – Universitätsmedizin Berlin, Flow & Mass Cytometry Core Facility Berlin Germany; ^8^ Department of Pharmacology Northwestern University Chicago IL USA; ^9^ CalciMedica Inc. La Jolla CA USA; ^10^ TRR 241 Research Initiative Berlin‐Erlangen Germany; ^11^ Clinician Scientist Program Berlin Institute of Health Berlin Germany

**Keywords:** Crohn's disease, mass cytometry, store‐operated calcium entry (SOCE), T cell transfer models of colitis, ulcerative colitis, Digestive System, Immunology

## Abstract

Inflammatory bowel disease (IBD) is characterized by dysregulated intestinal immune responses. Using mass cytometry (CyTOF) to analyze the immune cell composition in the lamina propria (LP) of patients with ulcerative colitis (UC) and Crohn's disease (CD), we observed an enrichment of CD4^+^ effector T cells producing IL‐17A and TNF, CD8^+^ T cells producing IFNγ, T regulatory (Treg) cells, and innate lymphoid cells (ILC). The function of these immune cells is regulated by store‐operated Ca^2+^ entry (SOCE), which results from the opening of Ca^2+^ release‐activated Ca^2+^ (CRAC) channels formed by ORAI and STIM proteins. We observed that the pharmacologic inhibition of SOCE attenuated the production of proinflammatory cytokines including IL‐2, IL‐4, IL‐6, IL‐17A, TNF, and IFNγ by human colonic T cells and ILCs, reduced the production of IL‐6 by B cells and the production of IFNγ by myeloid cells, but had no effect on the viability, differentiation, and function of intestinal epithelial cells. T cell‐specific deletion of CRAC channel genes in mice showed that *Orai1*, *Stim1*, and *Stim2*‐deficient T cells have quantitatively distinct defects in SOCE, which correlate with gradually more pronounced impairment of cytokine production by Th1 and Th17 cells and the severity of IBD. Moreover, the pharmacologic inhibition of SOCE with a selective CRAC channel inhibitor attenuated IBD severity and colitogenic T cell function in mice. Our data indicate that SOCE inhibition may be a suitable new approach for the treatment of IBD.

## Graphical abstract

The immune cell composition, signaling cascades, and cytokine networks controlling inflammation in therapy‐refractory inflammatory bowel diseases (IBD) remain incompletely understood.

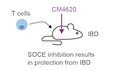

## Introduction

Inflammatory bowel disease (IBD) is a chronic inflammatory disease of the gastrointestinal (GI) tract that manifests predominantly as two related disease entities, ulcerative colitis (UC) and Crohn's disease (CD). Both forms of IBD are associated with diarrhea, abdominal pain, fatigue as well as the development of colorectal cancer in patients with longstanding colitis and the development of fistulae and stricturing disease in patients with CD (Uhlig & Powrie, 2018). While UC predominantly affects the colon and is characterized by a superficial inflammation of the LP, CD can involve the entire GI tract and cause trans‐mural inflammation. New single‐cell technologies such as single‐cell RNA‐sequencing and mass cytometry have greatly advanced our pathophysiological understanding of IBD (Corridoni *et al*, 2020; Mitsialis *et al*, 2020) and have helped better delineate the composition of intestinal immune cells in IBD patients that drive inflammation. The LP of UC patients is characterized by a significant enrichment of TNF producing CD8^+^ effector T cells (Corridoni *et al*, 2020), IL‐17 producing effector memory CD4^+^ T cells, and an expansion of T regulatory (Treg) cells producing inflammatory cytokines (Mitsialis *et al*, 2020). HLA‐DR^+^CD56^+^ granulocytes as well as TNF and IFNγ‐producing B cells are increased in the mucosa of CD patients (Mitsialis *et al*, 2020). Despite these findings, the immune cell composition, signaling cascades, and the cytokine networks controlling inflammation in therapy‐refractory IBD remain incompletely understood. Recent advances that have revolutionized IBD treatment include blocking antibodies against pro‐inflammatory cytokines such as TNF (e.g. infliximab or adalimumab), IL‐12, and IL‐23 (ustekinumab) and against integrins such as vedolizumab (Neurath, 2019). Nevertheless, intestinal and colonic resections are still frequently required in patients with therapy‐refractory IBD, and novel treatment modalities are urgently needed to improve IBD outcomes.

SOCE is the predominant Ca^2+^ influx pathway in most immune cells and is required for the activation, differentiation, and function of murine and human lymphocytes including B, NK, and T cells. Stimulation of the T cell receptor (TCR) leads to production of the second messenger inositol‐1,4,5 triphosphate (IP_3_), which triggers a transient release of Ca^2+^ from endoplasmic reticulum (ER) Ca^2+^ stores through IP_3_ receptors into the cytoplasm (Taylor *et al*, 2009). The concomitant reduction of Ca^2+^ concentrations in the ER is sensed by stromal interaction molecules (STIM) 1 and STIM2 in the ER membrane, which are subsequently activated and translocated to the plasma membrane (Liou *et al*, 2005). Activated STIM1 and STIM2 bind to ORAI1 and its homologs ORAI2 and ORAI3, which form the pore of the Ca^2+^ release‐activated Ca^2+^ (CRAC) channel. SOCE through CRAC channels causes a sustained elevation of intracellular Ca^2+^ levels (Prakriya *et al*, 2006; Vaeth *et al*, 2020), which is required for the activation of numerous Ca^2+^‐dependent enzymes including calcineurin, calmodulin kinases, or Erk1/2 and transcription factors such as NF‐κΒ, CREB, and NFAT (Berry *et al*, 2018; Vaeth & Feske, 2018b; Wang *et al*, 2020). SOCE is essential for the transcription of cytokines including IL‐2, IFNγ, and TNF by T cells and other immune cells. In addition, SOCE regulates several metabolic pathways such as glycolysis and mitochondrial respiration, thereby controlling lymphocyte proliferation and effector functions (Vaeth *et al*, 2017; Kaufmann *et al*, 2019; Wang *et al*, 2020). The pathophysiological importance of SOCE for immune function is emphasized by patients with loss‐of‐function mutations in *STIM1* or *ORAI1* genes, who suffer from combined immunodeficiency with recurrent infections (McCarl *et al*, 2009; Picard *et al*, 2009; Fuchs *et al*, 2012; Lacruz & Feske, 2015; Kahlfuss *et al*, 2020). Previous studies indicate that SOCE is increased in T cells isolated from inflamed compared to non‐inflamed mucosa of IBD patients (Schwarz *et al*, 2004). Another study has attributed this increase to an elevated expression of STIM1 in CD45^+^ lamina propria mononuclear cells (LPMCs) from inflamed intestinal tissue (Liang *et al*, 2022). Together, these findings suggest that SOCE acts as an important signaling axis that promotes intestinal inflammation. Accordingly, genetic or pharmacological inhibition of SOCE suppresses pro‐inflammatory T cell functions and intestinal inflammation in animal models of colitis (McCarl *et al*, 2010; Vaeth *et al*, 2017).

Given the critical role of SOCE in immune cells, we hypothesized that pharmacologic blockade of SOCE might attenuate the pro‐inflammatory function of lymphoid and myeloid immune cells from patients with therapy‐refractory IBD and therefore represent a new strategy for immune modulation. Using a murine T cell transfer model of colitis, we show that deletion of the CRAC channel genes *Stim1*, *Stim2*, or *Orai1* in T cells prevents IBD and that the severity of intestinal inflammation correlates with the level of SOCE. By applying mass cytometry (CyTOF), we characterized immune cell subsets in the colonic LP of therapy refractory IBD patients and investigated the effects of the SOCE inhibitors BTP2 and CM4620 on intestinal immune cell populations from IBD patients. SOCE inhibition efficiently suppressed various pro‐inflammatory functions of human T cells in a dose‐dependent manner and inhibited the function of innate lymphoid cells (ILC) and, to a lesser degree, that of myeloid cells and B cells. Importantly, treatment with BTP2 had no detectable effects on epithelial barrier functions of primary human and murine intestinal epithelial cells (IEC) *in vitro*. Treatment of mice in which IBD had been induced by adoptive T cell transfer with the selective SOCE inhibitor CM4620 attenuated the clinical course of colitis, which was associated with reduced neutrophil infiltration of the LP and decreased IFNγ and TNFα production by CD4^+^ T cells, suggesting that SOCE inhibition may be a new treatment option for IBD.

## Results

### SOCE in T cells is required for the induction of colitis in mice

**Figure 1:**
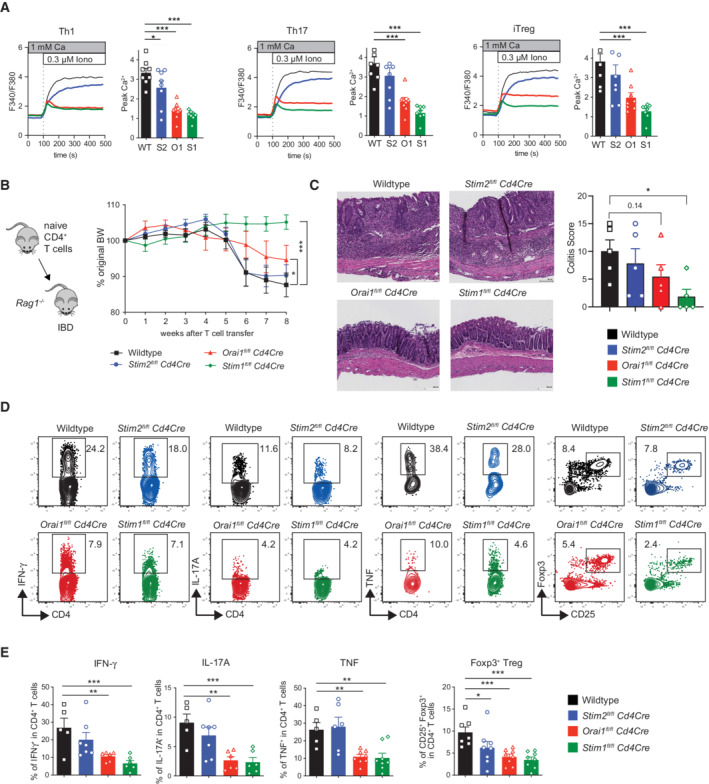
Deletion of SOCE in CD4^+^ T cells suppresses the induction of colitis in mice A–ENaïve CD4^+^ T cells were isolated from the spleen and LNs of WT, *Stim2* ^*fl/fl*^ *Cd4Cre* (S2), *Orai1* ^*fl/fl*^ *Cd4Cre* (O1), and *Stim1* ^*fl/fl*^ *Cd4Cre* (S1) mice and differentiated into Th1, Th17, and iTreg cells. (A) Fura‐2 loaded cells were stimulated with 0.3 μM ionomycin to induce SOCE. Representative Ca^2+^ traces (left) and peak Ca^2+^ levels (right). Data are from the mean ± SEM of four mice per group. (B) Induction of colitis in *Rag1* ^*−/−*^ mice by adoptive transfer of naive CD4^+^ T cells from wildtype, *Stim2* ^*fl/fl*^ *Cd4Cre*, *Orai1* ^*fl/fl*^ *Cd4Cre* and *Stim1* ^*fl/fl*^ *Cd4Cre* mice. Weight curves of recipient *Rag1* ^*−/−*^ mice following injection of naive CD4^+^ T cells. Data are from two independent experiments with eight recipient mice per group. (C) H&E stains of colon tissues and histological inflammation scores of *Rag1* ^*−/−*^ mice 8 weeks after T cell transfer. Data are from one representative experiment with five mice per group. (D, E) Representative flow cytometry plots and frequencies of donor CD4^+^ T cells producing IFNγ, IL‐17A, TNF or expressing Foxp3 that were isolated from the mesenteric LNs of recipient *Rag1* ^*−/−*^ mice. Data are from two independent experiments with 3–8 mice per group. Statistical analyses by unpaired student's *t*‐test: ***P < 0.001 **P < 0.01 *P < 0.05. Source data are available online for this figure.

We had previously shown that murine T cells lacking functional CRAC channels are unable to induce colitis upon adoptive transfer into lymphopenic mice (McCarl *et al*, 2010; Vaeth *et al*, 2017). Naive CD4^+^ T cells isolated from *Stim1* ^*fl/fl*^ *Cd4Cre* mice lacked STIM1 expression and had strongly suppressed SOCE. Likewise, CD4^+^ T cells from *Orai1* ^*R93W*^ knock‐in mice that express a channel‐dead version of ORAI1 lacked SOCE almost completely, likely because of a dominant negative effect of the mutant ORAI1 protein on other ORAI paralogues (ORAI2 and ORAI3) with which ORAI1 can form heteromeric channels (Thompson *et al*, 2009; McCarl *et al*, 2010; Vaeth *et al*, 2017). To better understand the role of CRAC channel components in colitogenic T cells and the quantitative requirements of SOCE in T cell‐mediated intestinal inflammation, we here used mice with T cell‐specific deletion of *Orai1*, *Stim1*, and *Stim2*. Naïve CD4^+^ T cells from *Orai1* ^*fl/fl*^ *Cd4*Cre, *Stim1* ^*fl/fl*^ *Cd4Cre*, and *Stim2* ^*fl/fl*^ *Cd4Cre* mice were polarized into Th1, Th17, and induced Treg (iTreg) cells *in vitro*. Whereas deletion of *Stim1* strongly suppressed SOCE, lack of *Orai1* reduced the amplitude of SOCE by approximately half compared to wildtype T cells (Fig 1A). Deletion of *Stim2* resulted in only moderately reduced SOCE. It is noteworthy that deletion of *Orai* and *Stim* genes affected SOCE in Th1, Th17, and iTreg cells to a similar degree. Moreover, Th1, Th17, and Treg cells from wildtype mice expressed comparable levels of *Orai* and *Stim* genes (Appendix Fig S1). We used T cells from this allelic series of mice with gradual defects of SOCE to investigate the dose–response relationship of SOCE and colitogenic T cell function *in vivo*. We isolated naive CD4^+^ T cells from *Orai1* ^*fl/fl*^ *Cd4*Cre, *Stim1* ^*fl/fl*^ *Cd4Cre*, and *Stim2* ^*fl/fl*^ *Cd4Cre* and wildtype mice and injected them into lymphopenic *Rag1* ^−/−^ host mice (Fig 1B). Whereas the transfer of *Stim2*‐deficient CD4^+^ T cells induced weight loss comparable to wildtype T cells, the injection of *Orai1*‐deficient and *Stim1‐*deficient CD4^+^ T cells resulted in significantly attenuated or no weight loss, respectively. The histological analysis of intestinal inflammation showed severe colitis in host mice that had received wildtype or *Stim2*‐deficient CD4^+^ T cells, whereas inflammation was partially reduced or absent following transfer of *Orai1*‐deficient and *Stim1*‐deficient CD4^+^ T cells, respectively (Fig 1C). Flow cytometric analysis of CD4^+^ T cells obtained from mesenteric lymph nodes (mLN) showed similar frequencies of cells producing IFNγ, TNF, and IL‐17A in mice that had received T cells from *Stim2* ^*fl/fl*^ *Cd4Cre* and wildtype mice (Fig 1D and E). By contrast, IFNγ, TNF, and IL‐17A production by adoptively transferred CD4^+^ T cells from *Orai1* and *Stim1‐*deficient mice was significantly reduced *in vivo* compared to wildtype T cells. The frequencies of Treg cells were significantly reduced in the absence of *Orai1* or *Stim1*, but not in the absence of *Stim2* (Fig 1D and E). Together, these findings suggest that moderate inhibition of SOCE in *Stim2‐*deficient CD4^+^ T cells has no effect on colitogenic T cell function, whereas more pronounced inhibition of SOCE in *Orai1 or Stim1‐*deficient CD4^+^ T cells gradually suppresses inflammatory cytokine production and their ability to induce intestinal inflammation.

### Inhibition of SOCE gradually inhibits the production of pro‐inflammatory cytokines by LPMCs in IBD patients

**Figure 2:**
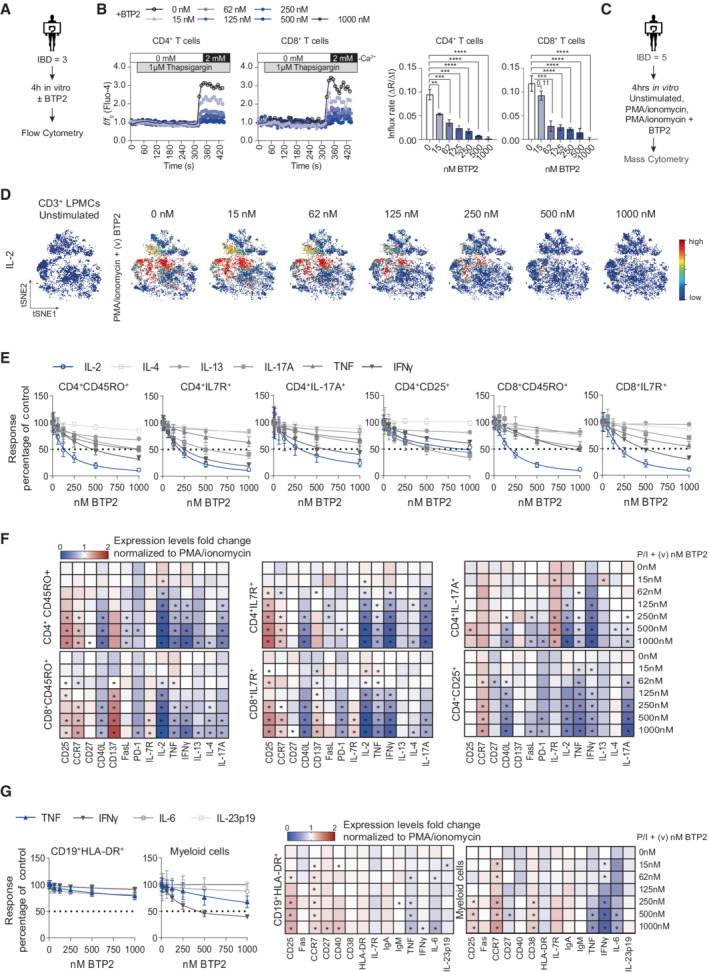
Inhibition of SOCE reduces pro‐inflammatory cytokine production by human lamina propria mononuclear cells (LPMC) in a dose‐dependent manner A, BCa^2+^ influx in human lamina propria CD4^+^ and CD8^+^ T cells from three IBD patient samples analyzed by flow cytometry. (A) Experimental design. (B) Ca^2+^ influx rates in T cells pre‐incubated for 4 h in the presence of 15–1,000 nM BTP2, which was present until data acquisition. SOCE was induced by stimulation with the sarco‐endoplasmic reticulum Ca^2+^ ATPase (SERCA) inhibitor thapsigargin (TG) in Ca^2+^ free buffer followed by the addition of 2 mM Ca^2+^. Bar graphs show the mean ± SEM of Ca^2+^ influx rates after Ca^2+^ readdition from one experiment (*n* = three IBD patients). Statistical difference were calculated using a RM one‐way ANOVA test: ****P < 0.0001 ***P < 0.001 **P < 0.01 *P < 0.05.C–GAnalysis of LPMC from five CD patients. (C) Experimental design for mass cytometry. (D) viSNE plots of CD45^+^CD3^+^ LPMCs stimulated with 20 ng/ml PMA and 1 μg/ml ionomycin for 4 h in the presence of various (v) concentrations (15–1,000 nM) of BTP2. viSNE plots are colored according to the expression level of IL‐2 (blue: low, red: high) and are representative of one CD patient. (E) Dose–response curves of the frequencies of cytokine producing CD4^+^ and CD8^+^ T cells after PMA/ionomycin stimulation for 4 h in the presence of increasing doses of BTP2. The frequencies of cytokine producing cells were normalized to control samples treated with PMA/ionomycin alone. Bar graphs show the mean ± SEM of five CD patient samples. (F) Heatmaps representing the median fold change of cytokine and cell surface marker expression in CD45^+^CD3^+^ LPMCs stimulated for 4 h with PMA/ionomycin in the presence of BTP2; data are normalized to PMA/ionomycin treatment alone. Statistical significance was calculated using a one‐tailed Wilcoxon matched‐pairs signed rank test, *P < 0.05. (G) Cytokine production by CD19^+^HLA‐DR^+^ B cells and CD11c^+^ myeloid cells in the presence of various (v) increasing doses (15–1,000 nM) of BTP2. Left: Dose–response curves showing frequencies of cytokine‐producing cells after 4 h stimulation with PMA/ionomycin ex vivo. Data are normalized to control samples stimulated with PMA/ionomycin in the absence of BTP2. Error bars represent SEM obtained from one experiment (*n* = 5 CD patients). Right: heatmaps showing the median fold change of cytokines and surface markers in CD19^+^HLA‐DR^+^ B cells and CD11c^+^ myeloid cells treated as described for the left panel. Significant fold changes in expression levels between samples were calculated using a one‐tailed Wilcoxon matched‐pairs signed rank test, *P < 0.05. Source data are available online for this figure.

To investigate the role of SOCE in the function of lamina propria mononuclear cells (LPMCs) and intestinal inflammation in IBD patients, we isolated LPMCs from surgical colon resectates of IBD patients and peripheral blood mononuclear cells (PBMCs) from healthy donors (HD) (Appendix Table S1 for clinical details) and assessed Ca^2+^ influx by flow cytometry after *ex vivo* incubation with increasing concentrations (15–1,000 nM) of the CRAC channel inhibitor BTP2 (Fig 2A) (Ishikawa *et al*, 2003; Ohga *et al*, 2008). Treatment with BTP2 for 4 h caused a dose‐dependent inhibition of SOCE in intestinal CD4^+^ and CD8^+^ T cells with almost complete inhibition of SOCE inhibition at 1 μM (Fig 2B). 1 μM BTP2 also significantly reduced SOCE in PBMCs of HD, specifically in CD4^+^ and CD8^+^ T cells, CD19^+^ B cells, CD14^+^ monocytes and, to a lesser degree, CD56^+^ NK cells (Appendix Fig S2A), demonstrating that BTP2 is a potent inhibitor of SOCE in human gut‐resident lymphocytes and PBMCs. To exclude that the observed differences in Ca^2+^ influx were caused by BTP2‐induced cell death of lymphocytes, we measured apoptosis of LPMCs of IBD patients after 4 h of incubation with 1 μM BTP2. As shown in Appendix Fig S2B, we did not observe increased cell death in BTP2‐treated LPMCs compared to untreated control cells.

**Figure EV1:**
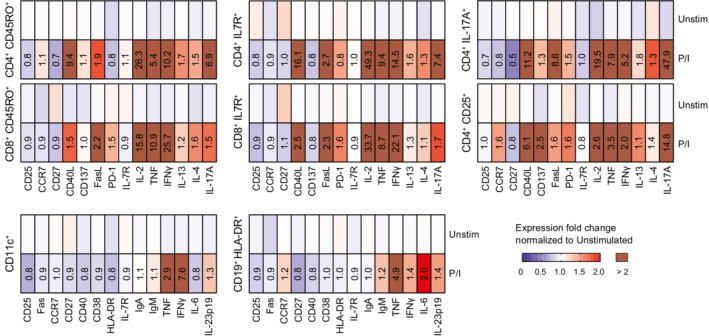
Effects of PMA/ionomycin stimulation on protein expression of human lamina propria (LP) immune cells Heatmaps representing the median fold change (FC) of cytokine and surface marker expression in CD45^+^CD3^+^ T cells (top row) and CD45^+^CD3^−^ immune cells (bottom row) isolated from colon lamina propria of five patients with Crohn's disease. Cells were treated with 20 ng/ml PMA and 1 μg/m (P/I) or DMSO (unstimulated) for 4 h *in vitro*, and protein expression was measured by mass cytometry (CyTOF). FC values are relative to unstimulated samples.Source data are available online for this figure.

To determine the effects of SOCE inhibition on the function of LPMCs isolated from IBD patients, we next evaluated the production of various pro‐inflammatory cytokines and factors in LPMCs of five CD patients after BTP2 treatment (15–1,000 nM) *in vitro* by mass cytometry (Fig 2C). LPMCs were stained with a panel of antibodies against lineage markers of T cells (CD3, CD4, and CD8), myeloid cells (CD11b, CD11c, and CD14), B cells (CD19, IgM, and IgA), and NK cells (CD56) similar to our previously published protocol (Ziegler *et al*, 2019; Hecker *et al*, 2022). We furthermore included antibodies against differentiation and homing markers (CCR7, CD25, CD33, FAS (CD95), CD103, IL7R (CD127), CD137), activation markers (CD40, CD45RA, CD45RO, CD86, HLA‐DR), cytokines (IL‐2, IL‐4, IL‐6, IL‐13, IL‐17A, IL‐23p40, IFNγ, TNF, CD40L, FasL), and additional markers (CD27, CD38, PD‐1). A detailed description of the antibody panel used for mass cytometry is provided in Appendix Table S2. To examine the effects of SOCE inhibition by BTP2 on the function of different human intestinal T cell subsets in a dose‐dependent manner, we applied the t‐distributed stochastic linear embedding (t‐SNE) algorithm and the *FlowSOM/ConsensusClusterPlus* self‐organizing map method (Bottcher *et al*, 2019), which allowed us to cluster CD45^+^CD3^+^ LPMCs into nine major cell subtypes (Appendix Fig S3A and B) according to the expression of major lineage markers listed in Appendix Table S3. We compared the expression of cytokines and activation markers between LPMCs stimulated with PMA/ionomycin for 4 h and samples treated with PMA/ionomycin in the presence of increasing concentrations of BTP2. Stimulation with PMA/ionomycin resulted in an increased expression of IL‐2 in CD45^+^CD3^+^ LPMCs (Fig EV1), which was inhibited gradually by increasing concentrations of the CRAC channel inhibitor BTP2 (Fig 2D). BTP2 also efficiently decreased the production of other cytokines including IFNγ, TNF, IL‐17A, IL‐13, and IL‐4 in a dose‐dependent manner (Fig 2E). Similar suppression of cytokine production was observed in both CD4^+^ and CD8^+^ effector (CD45RO^+^) and memory (IL‐7R^+^) T cells, as well as CD4^+^IL‐17^+^ Th17 cells. The weakest effects of SOCE inhibition on cytokine production were observed in CD4^+^CD25^+^IL7R^−^ Treg cells. Only IL‐2, IL‐17A, and TNF were suppressed by ~50% with the highest concentration of BTP2 (1 μM) in Treg cells, whereas the same concentration of BTP2 strongly suppressed IL‐2 and IFNγ in effector and memory CD4^+^ and CD8^+^ T cells.

These findings are consistent with a greater resistance of murine iTreg cells to genetic (Taylor *et al*, 2009; Kaufmann *et al*, 2016) and pharmacologic ablation of SOCE compared to Th1 and Th17 cells (Appendix Fig S4). Whereas IFNγ and IL‐17A expression by murine Th1 and Th17 cells was suppressed by low (~ 20–100 nM) concentrations of BTP2, expression of the effector molecule CTLA4 on iTreg cells was not affected at these concentrations (Appendix Fig S4A–D). Low (~ 20–100 nM) concentrations of BTP2 decreased the expression of the Th17‐specific transcription factor RORγt but had weaker or no effects on Foxp3 or T‐bet expression, respectively (Appendix Fig S4E and F). These findings emphasize the strong dependence of human and murine Th17 function on SOCE compared to other T cell subsets.

Besides cytokines, the expression of several cell surface molecules on CD3^+^ LPMCs such as CD40L, FasL and PD‐1 was decreased by increasing concentrations of BTP2 (Fig 2F), which is consistent with the known role of Ca^2+^ and NFAT in their transcriptional regulation (Tsytsykova *et al*, 1996; Oestreich *et al*, 2008; Desvignes *et al*, 2015). For other markers such as CD25, CD137 (4‐1BB), or CCR7, we found a dose‐dependent upregulation upon treatment with BTP2 (Fig 2F). Interestingly, this increase was cell type–specific and did not occur in all T cell subsets. For instance, CD25 was upregulated in BTP2‐treated CD4^+^ and CD8^+^ effector and memory T cells, but not in Th17 and Treg cells after pharmacologic blockade of SOCE. Similarly, CD137 was significantly upregulated after BTP2 treatment only in effector and memory CD8^+^ T cells. These findings suggest that cell type–specific thresholds for the effects of SOCE may exist that control the functions of different T cell subsets.

To better understand the role of SOCE in other LPMC subsets besides T cells, we analyzed the effects of BTP2 treatment on the expression of cytokines and cell surface molecules by CD45^+^CD3^−^non‐T cells, in particular B cells and myeloid cells, among LPMCs of the same IBD patients (Fig 2G, Appendix Fig S5). In contrast to T cells, cytokine production by CD19^+^ HLA‐DR^+^ B cells was barely affected by increasing BTP2 concentrations. A slightly more pronounced suppression, especially of IFNγ and to a lesser degree of TNF, was observed in CD11c^+^ myeloid cells (cluster 3 as indicated in the heatmap of Appendix Fig S5). By contrast, SOCE inhibition resulted in a significant induction of CD25 and CCR7 expression in both B cells and myeloid cells (Fig 2G). Similar observations were made in independent experiments using CD3^−^ LPMCs isolated from three UC patients (Appendix Fig S6). Collectively, these data demonstrate the significant effects of SOCE inhibition on pro‐inflammatory cytokine expression in LP T cells and, to a lesser degree, myeloid cells of CD and UC patients and the complex, subset‐dependent regulation of immune cell activation markers.

### Altered immune cell composition in the lamina propria of IBD patients

**Figure 3:**
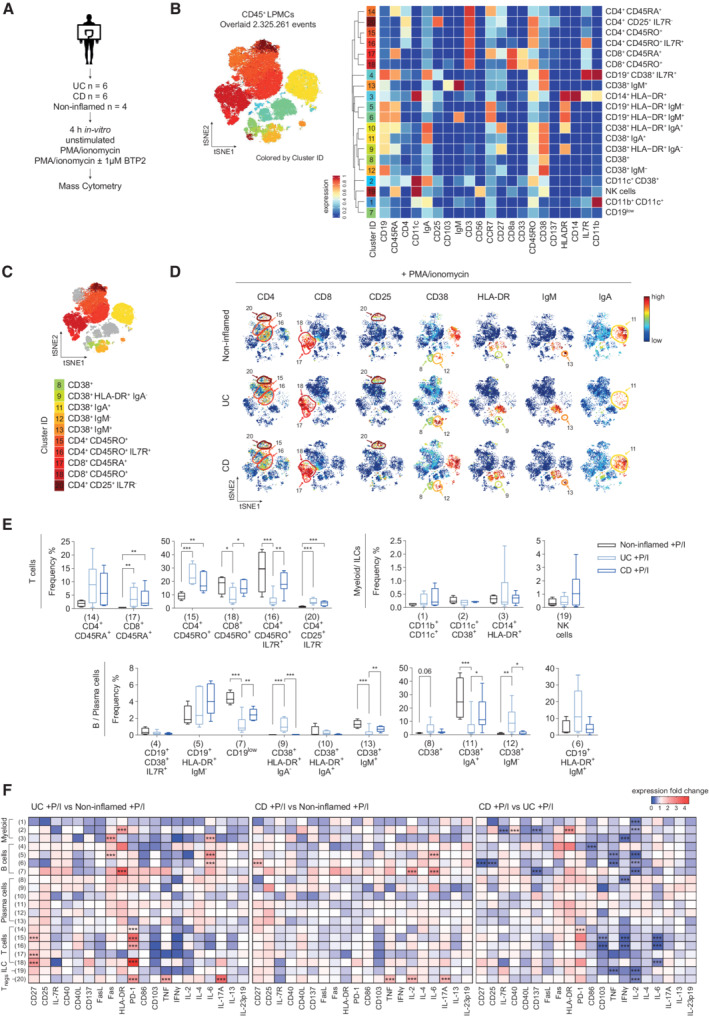
Global mass cytometric analysis of the immune cell composition in the colonic lamina propria of therapy refractory IBD patients AExperimental setup for mass cytometric assays. Non‐inflamed: *n* = 4, CD: *n* = 6, UC: *n* = 6.BFlowSOM plot of merged FCS files from LPMCs of non‐inflamed controls, UC or CD patients after stimulation with PMA/ionomycin ± 1 μM BTP2 for 4 h *ex vivo*. Colors of the t‐SNE plot represent 20 clusters of distinct CD45^+^ LPMC lineages. The heatmap shows the expression levels of 21 markers used for defining cell clusters.CFlowSOM map of merged FCS files from CD45^+^ cells of non‐inflamed controls, UC and CD patients that were left unstimulated or treated with PMA/ionomycin ± 1 μM BTP2. Colors indicate altered cell clusters in CD patients vs. controls, UC patients vs. controls and CD vs. UC patients.DviSNE plots of one exemplary non‐inflamed control, UC and CD patient colored by marker expression levels (blue: low, red: high).EQuantified frequencies (%) of each cell subset defined by the cluster analysis. Statistical significance was calculated using the edgeR statistical framework with negative binomial GLM and a false discovery rate adjusted to 10% using the Benjiamini–Hochberg procedure (*P_adj_ < 0.05, **P_adj_ < 0.01, ***P_adj_ < 0.001). Boxes extend from the 25^th^ to 75^th^ percentiles. Whisker plots show the min (smallest) and max (largest) values. The line in the box denotes the median from one experiment (non‐inflamed *n* = 4, CD: *n* = 6, UC: *n* = 6 patients).FHeatmaps representing the median fold change of cytokine and surface marker expression in CD45^+^ LPMCs that were isolated from non‐inflamed, UC and CD samples and activated for 4 h with PMA/ionomycin *ex vivo*. Statistical significance was calculated using an unpaired *t* test with FDR adjustment to 1% using the Benjamini, Krieger, and Yekutieli procedure; *P_adj_ < 0.05, **P_adj_ < 0.01, ***P_adj_ <0.001. Source data are available online for this figure.

**Figure 4:**
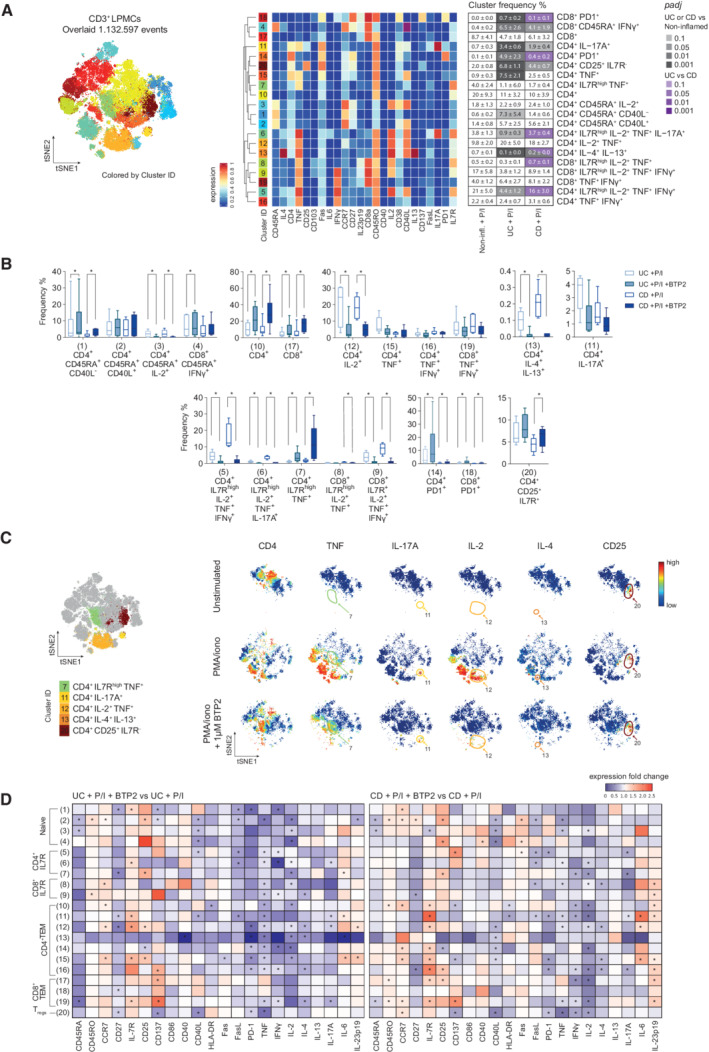
Inhibition of SOCE suppresses the activation and function of human colonic T cells AFlowSOM plot of merged FCS files from samples treated with PMA/ionomycin ± 1 μM BTP2 (non‐inflamed: *n* = 4, CD: *n* = 6, UC: *n* = 6). Colors of FlowSOM plot indicate 20 distinct clusters of CD45^+^CD3^+^ LPMCs. Heatmaps show the expression levels of 24 markers used for cluster determination. The table shows the mean ± SEM of frequencies (%) for each cell subset defined in non‐inflamed controls, UC and CD patients after 4 h stimulation with PMA/ionomycin *in vitro*. Statistical significance was calculated using the edgeR statistical framework with negative binomial GLM and a false discovery rate adjusted to 10% using the Benjiamini–Hochberg procedure.BBox plots showing frequencies (%) of each cell subset defined by the cluster analysis in non‐inflamed, UC and CD samples following stimulation with PMA/ionomycin ± BTP2 for 4 h *in vitro*. Statistical significances were calculated using a one‐tailed paired Wilcoxon matched‐pairs signed‐rank test, *P < 0.05. Boxes range from the 25^th^ to 75^th^ percentiles. Whisker plots show the min (smallest) and max (largest) values. The line in the box denotes the median obtained from one experiment (non‐inflamed: *n* = 4, CD: *n* = 6, UC: *n* = 6 patients).CviSNE plots of one exemplary CD patient. Identification of cell subsets (left) and expression levels of cytokines after PMA/ionomycin stimulation in the presence or absence of BTP2 (blue; low, red: high).DHeatmaps indicating the median fold change expression of cytokines and surface molecules in CD45^+^CD3^+^ LPMCs stimulated with PMA/ionomycin ± BTP2 for 4 h *in vitro*. Values are normalized to stimulated, non‐BTP2 treated samples. Statistical significance was calculated using a one‐tailed paired Wilcoxon matched‐pairs signed‐rank test, *P < 0.05. Source data are available online for this figure.

**Figure 5:**
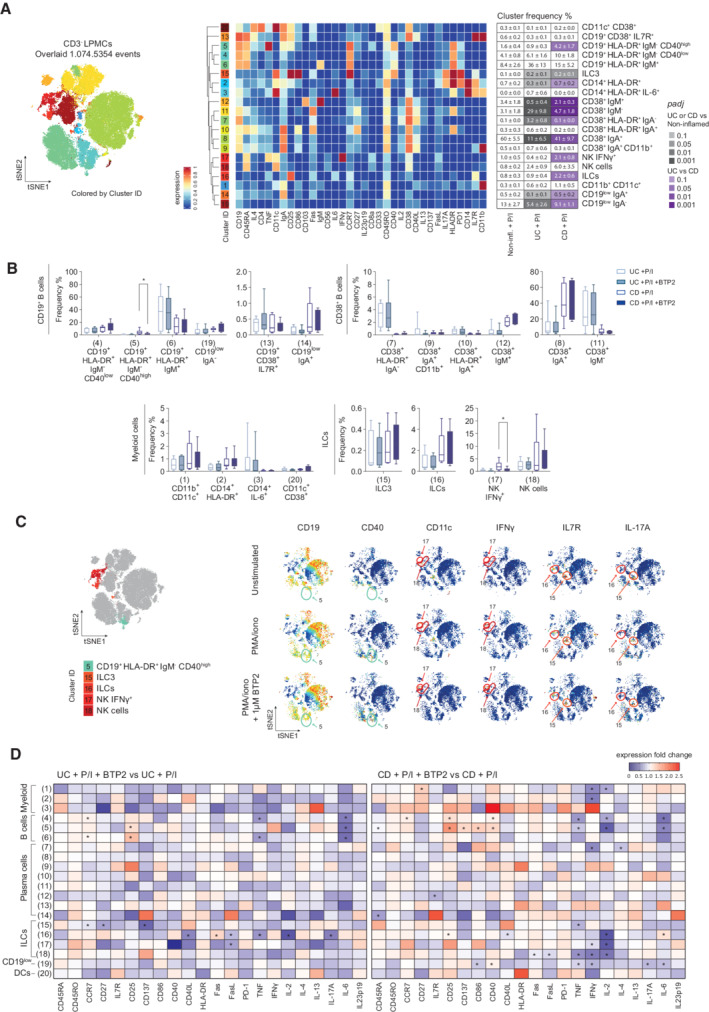
Effects of SOCE inhibition on CD3^−^ lamina propria cells AFlowSOM plot of merged FCS files from unstimulated samples or samples treated with PMA/ionomycin ± 1 μM BTP2 (non‐inflamed: *n* = 4, CD: *n* = 6, UC: *n* = 6). Colors of FlowSOM plot indicate 20 distinct clusters of CD45^+^CD3^−^ LPMCs. Heatmap clusters showing the expression levels of 34 markers used for cluster determination. The table shows the mean ± SEM of frequencies (%) for each cell subset defined in non‐Inflamed, UC, and CD samples after stimulation with PMA/ionomycin for 4 h. Statistical significance was calculated using the edgeR statistical framework with negative binomial GLM and a false discovery rate adjusted to 10% using the Benjiamini–Hochberg procedure.BBox plots show frequencies (%) of each cell subset defined by the cluster analysis in samples of non‐inflamed controls, UC or CD patients after stimulation with PMA/ionomycin ± BTP2 for 4 h *ex vivo*. Statistical significances were calculated by one‐tailed paired Wilcoxon matched‐pairs signed‐rank test, *P < 0.05. Boxes range from the 25^th^ to 75^th^ percentiles. Whisker plots show the min (smallest) and max (largest) values. The line in the box denotes the median obtained from one experiment (non‐inflamed: *n* = 4, CD: *n* = 6, UC: *n* = 6 patients).CviSNE plots of one exemplary CD patient. Identification of cell subsets (left) and expression levels of cell markers after PMA/ionomycin stimulation in the presence or absence of BTP2 (blue; low, red: high).DHeatmaps indicating the median fold change expression of cytokines and surface markers in CD45^+^CD3^−^ LPMCs activated with PMA/ionomycin ± BTP2 for 4 h *in vitro*. Values are normalized to stimulated, non‐BTP2 treated samples. Significant differences were calculated using a one‐tailed paired Wilcoxon matched‐pairs signed‐rank test, *P < 0.05. Source data are available online for this figure.

After establishing BTP2 as a potent inhibitor of SOCE and the function of human LPMC, we next investigated whether the function of disease‐modifying LPMC subsets in IBD patients can be modulated by pharmacologic SOCE inhibition. To address this question, we first aimed to characterize the composition of immune cell compartments in the LP of UC‐ and CD patients with therapy‐refractory IBD, which is currently not well‐defined. We used the following stepwise approach: First, we compared the frequencies and functional status of immune cell subsets within LPMCs isolated from six patients with UC and six patients with CD undergoing colon resection due to refractory disease. Using mass cytometry, we compared the LPMC composition of IBD patients to that in non‐inflamed colon mucosa obtained from non‐IBD control patients (Appendix Table S1 for clinical details). Isolated LPMC were stimulated with PMA/ionomycin for 4 h *ex vivo* and analyzed by CyTOF using a panel of 37 markers (Fig 3A and Appendix Table S2), which allowed us to determine functional aspects of disease‐specific cell subsets that drive inflammation in UC and CD. To investigate the effects of SOCE inhibition on these IBD‐characterizing LPMC subsets, we stimulated LPMC in the presence or absence of a single concentration of 1 μM BTP2 for which we had observed an almost complete suppression of Ca^2+^ influx and cytokine production in LP T cells (Fig 2A). For the evaluation of CyTOF data, we performed a general high‐dimensional analysis of all CD45^+^ cells that were clustered based on their expression of lineage markers (Fig 3). This was followed by in‐depth, separate analyses of CD45^+^CD3^+^ LP T cells (Fig 4) and CD45^+^CD3^−^ non‐T cell LPMCs (Fig 5) that were clustered according to their expression of surface makers and their expression of cytokines. For the abovementioned analyses, we first compared the frequencies of cell clusters between UC, CD, and non‐inflamed controls and next assessed the effects of BTP2 on the activation and function of LPMC subsets characteristic of IBD.

The analysis of all CD45^+^ cells (Appendix Fig S7) allowed us to identify 20 clusters of LPMC subsets based on the expression of cell lineage markers listed in Appendix Table S4 and Fig 3B. LPMC clusters that are significantly dysregulated in UC and/or CD patients compared to non‐inflamed controls are highlighted in the CD45^+^ FlowSOM map in Fig 3C, and complementary viSNE maps for individual markers are shown in Fig 3D. This analysis revealed several significant differences between LPMC populations in CD patients vs. controls, UC patients vs. controls, and between CD and UC patients. Compared to LPMC from non‐inflamed controls, UC and CD patients both showed a significant increase in the frequencies of CD4^+^CD45RO^+^ effector T cells (cluster 15), CD8^+^CD45RA^+^ naive T cells (cluster 17), and CD4^+^CD25^+^IL7R^−^ Treg cells (cluster 20) (Fig 3E). Increased Treg frequencies are consistent with previous reports (Lord *et al*, 2015; Martin *et al*, 2019) and the hypothesis that Treg numbers are elevated in the LP of IBD patients as a compensatory immune‐regulatory mechanism to control inflammation. We also observed differences in immune cell populations that were specific to UC patients. The frequencies of CD38^+^IgA^+^ (cluster 11) and CD38^+^IgM^+^ (cluster 13) plasma B cells were significantly reduced in UC patients compared to CD patients and non‐IBD controls, whereas CD38^+^ (cluster 8), CD38^+^HLA‐DR^+^IgA^−^ (cluster 9), and CD38^+^IgM^−^ (cluster 12) B cells were markedly expanded in UC patients compared to non‐inflamed controls. These findings suggest a profound, UC‐specific dysregulation of the B cell compartment. UC patients also had significantly fewer CD4^+^CD45RO^+^IL7R^+^ effector memory T cells (cluster 16) and CD8^+^CD45RO^+^ effector T cells (cluster 18) compared to CD patients and controls.

After defining the differences between LPMC subsets in IBD patients and controls, we next analyzed the expression levels of activation markers and cytokines in different LPMC subsets of UC patients, CD patients, and non‐inflamed controls after stimulation with PMA/ionomycin for 4 h *ex vivo* (Fig 3F). Treg cells (cluster 20) of both UC and CD patients produced more pro‐inflammatory cytokines such as TNF and IL‐17 than non‐inflamed controls, whereas Treg cells of CD patients also showed increased production of IL‐2. These findings are in line with a recent report (Mitsialis *et al*, 2020). Furthermore, we observed a significant upregulation of PD‐1 and CD27 on different T cell subsets of UC but not CD patients compared to controls, suggesting that T cell exhaustion might be a feature of the chronic inflammatory milieu in UC patients. Myeloid cells and B cells of both UC and CD patients were characterized by a higher expression of HLA‐DR and IL‐6 than non‐inflamed controls. Overall, these findings demonstrate that the balance of effector CD4^+^ and CD8^+^ T cells in the LP of IBD patients is severely perturbed compared to non‐inflamed controls. In addition, we observed profound differences in the composition of B cells and innate immune cells in the LP of UC and CD patients, with a particular reduction of IgA and IgM expressing CD38^+^ plasma cells in UC patients but not in CD patients.

### Inhibition of SOCE modulates the function of IBD‐defining colonic T cell populations

We next aimed to determine in more detail how SOCE regulates the activation of IBD‐characterizing LPMCs and their ability to produce pro‐inflammatory cytokines that are implicated in the pathophysiology of UC and CD. We therefore conducted separate analyses for CD45^+^CD3^+^ T cells (Fig 4) and CD45^+^ CD3^−^ non‐T cells (Fig 5) within LPMCs of IBD patients and non‐inflamed controls using the gating strategy shown in Appendix Figs S7 and S8. By subsequently clustering the cells not only based on their expression of lineage markers but also by their expression of differentiation markers and cytokines (Appendix Table S5), we identified 20 subsets of cells within CD45^+^CD3^+^ LP T cells (Fig 4A). UC and CD patients were both characterized by an enrichment of CD4^+^IL‐17A^+^ T cells (cluster 11), CD8^+^CD45RA^+^IFNγ^+^ T cells (cluster 4), and CD4^+^CD25^+^IL7R^−^ Treg cells (cluster 20) compared to non‐inflamed controls (Fig 4A). Unexpectedly, the frequency of CD4^+^IL‐4^+^IL‐13^+^ Th2 cells (cluster 13) was significantly reduced in both CD and UC compared to control samples, suggesting that these cells may not play a major pathogenic role in the maintenance of chronic inflammation in therapy‐refractory IBD. UC patients had higher frequencies of CD4^+^ PD‐1^+^ (cluster 14), CD8^+^ PD‐1^+^ (cluster 18) and CD4^+^ TNF^+^ (cluster 15) T cells than CD patients and non‐IBD controls. By contrast, the frequencies of CD4^+^IL7R^high^IL‐2^+^TNF^+^IFNγ^+^ (cluster 5) and CD4^+^IL7R^high^IL‐2^+^TNF^+^IL‐17A^+^ (cluster 6) T cells were significantly reduced in UC patients compared to CD patients and non‐inflamed control samples (Fig 4A). Together these findings demonstrate that the composition of CD4^+^ and CD8^+^ T cell populations and their activation status are significantly perturbed in the LP of IBD patients, with additional differences between UC and CD patients.

We next investigated how the inhibition of SOCE affects the function of the different CD3^+^ T cell subsets isolated from the LP of UC and CD patients. Inhibition of SOCE by 1 μM BTP2 during *ex vivo* stimulation with PMA/ionomycin resulted in strongly reduced frequencies of naive CD4^+^IL‐2^+^ T cells (cluster 3), CD4^+^IL‐2^+^TNF^+^ T cells (cluster 12), and IL‐4^+^IL‐13^+^ Th2 cells (cluster 13) in UC and CD patients (Fig 4B and C). Moreover, the frequencies of pro‐inflammatory CD4^+^IL‐7R^+^ and CD8^+^IL‐7R^+^ memory T cells producing IL‐2, IFNγ, and TNF (clusters 5 and 9) were significantly reduced in UC and CD patients after SOCE inhibition. We also observed a decrease in the percentages of CD4^+^IL‐17A^+^ T cells (cluster 11) and CD4^+^IL‐7R^+^ memory T cells producing IL‐2, TNF, and IL‐17A (cluster 6) in BTP2‐treated LPMC of UC and CD patients. Inhibition of SOCE in LPMC isolated from UC and CD patients also resulted in a relative increase of non‐activated CD4^+^ and CD8^+^ T cells (clusters 10 and 17 cells) and naive CD4^+^ T cells (cluster 1) as well as an increase of cells that were characterized by low or intermediate TNF expression such as CD4^+^IL‐7R^high^ TNF^+^ memory T cells (cluster 7) (Appendix Fig S9, Fig 4B and C). We attribute this increase to the fact that BTP2 potently suppresses T cell activation and TNF expression, thereby increasing the frequencies of T cells that are not activated and that are intermediate producers of TNF. BTP2 treatment also moderately increased the frequencies of CD4^+^PD‐1^+^ and CD8^+^PD‐1^+^ T cells (clusters 14 and 18) in both UC and CD patients and CD4^+^CD25^+^IL7R^−^ Treg cells (cluster 20) in CD patients (Fig 4B).

We next determined the effects of SOCE inhibition on the expression of individual cytokines and functional markers of T cells (Fig 4D). BTP2 treatment resulted in a significant suppression of TNF in the majority of T cell subsets of UC patients (Fig 4D, left panel) and to a somewhat lesser degree of CD patients (Fig 4D, right panel). IFNγ and IL‐2 production were similarly affected by BTP2 in UC and CD patient samples; a notable exception was Treg cells because IFNγ and IL‐2 production were significantly suppressed by BTP2 only in CD but not UC patients. The effects of SOCE inhibition on IL‐4, IL‐13, and IL‐17A production were more moderate and reached significance in only a few T cell subsets in both UC and CD patients. It is noteworthy that BTP2 inhibited the production of cytokines and suppressed the expression of activation markers in the IL‐4^+^IL‐13^+^ Th2 subset (cluster 13) in UC patients and to a lesser degree also in CD patients, suggesting that SOCE is a potent regulator of Th2 cell function. Among surface markers, we observed a significant decrease in the expression of FasL, CD40L, and PD‐1 upon inhibition of SOCE, which was expected since the transcription of these molecules, like that of many of the cytokines described above, is dependent on Ca^2+^ signaling and NFAT (Tsytsykova *et al*, 1996; Oestreich *et al*, 2008; Desvignes *et al*, 2015). In contrast, the increased expression of several T cell markers such as IL‐7R, CD137, CD25, and CCR7 as well as the cytokine IL‐23p19 in certain T cell subsets of UC and CD patients after BTP2 treatment was not anticipated. It is noteworthy that the expression of the co‐stimulatory receptor CD137 (4‐1BB) was differentially affected by SOCE inhibition; while it was upregulated in BTP2‐treated effector T cells, its expression decreased in Treg cells upon inhibition of SOCE. Collectively, our data demonstrate that SOCE is a potent regulator of T cell activation and function in LP T cells of both UC and CD patients.

### SOCE regulates the function of NK cells and ILCs and is required for the activation of B cells

We next focused our analysis on characterizing the composition of non‐T cell subsets within LPMCs of IBD patients and non‐inflamed controls and investigated the effects of SOCE inhibition on these cells. An in‐depth analysis of CD45^+^CD3^−^ cells allowed us to define 20 cell clusters (Fig 5A) based on the expression of lineage markers, cytokines, and functional markers described in Appendix Tables S2 and S6. We detected an increase of IL‐17‐producing ILC3 cells (cluster 15) in colonic mucosa samples of UC and CD patients compared to non‐inflamed tissue controls (Fig 5A), which is in line with a recent study that had observed a larger abundance of ILC3 cells in the peripheral blood of IBD patients with active disease (Mitsialis *et al*, 2020). In addition, we observed that the frequencies of CD11c^+^CD11b^+^ myeloid cells (cluster 1) were increased in CD and, to a lesser degree, in UC patients compared to non‐inflamed controls. Interestingly, CD patients had increased frequencies of CD3^−^CD19^−^IL7R^+^CD25^+^ ILCs (cluster 16) and IFNγ^+^ NK cells (cluster 17) as well as activated HLA‐DR^+^CD40^+^ B cells (cluster 5) compared to UC samples. A notable difference between both patient cohorts was the decrease in class switched CD38^+^IgA^+^ B cells (cluster 8) and increase in CD38^+^IgM^−^ B cells (cluster 11) in UC compared to CD patients and healthy controls (Fig 5A).

**Figure EV2:**
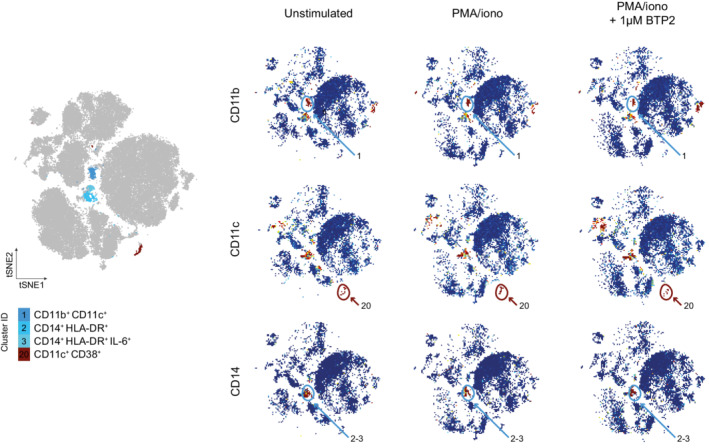
Four clusters of myeloid cells in the colon lamina propria (CLP) of IBD patients and effects of SOCE inhibition (Left) FlowSOM plot of merged FCS files from unstimulated IBD samples and samples treated with PMA/ionomycin ± 1 μM BTP2 (non‐inflamed: *n* = 4, CD: *n* = 6, UC: *n* = 6). Colors indicate myeloid cell clusters within CD45^+^CD3^−^ LPMCs. (Right) viSNE plots of one exemplary CD patient. Colors indicate expression levels of cell surface markers (blue: low; red: high) in cells left unstimulated, stimulated with PMA/ionomycin or stimulated in the presence of 1 μM BTP2.

**Figure EV3:**
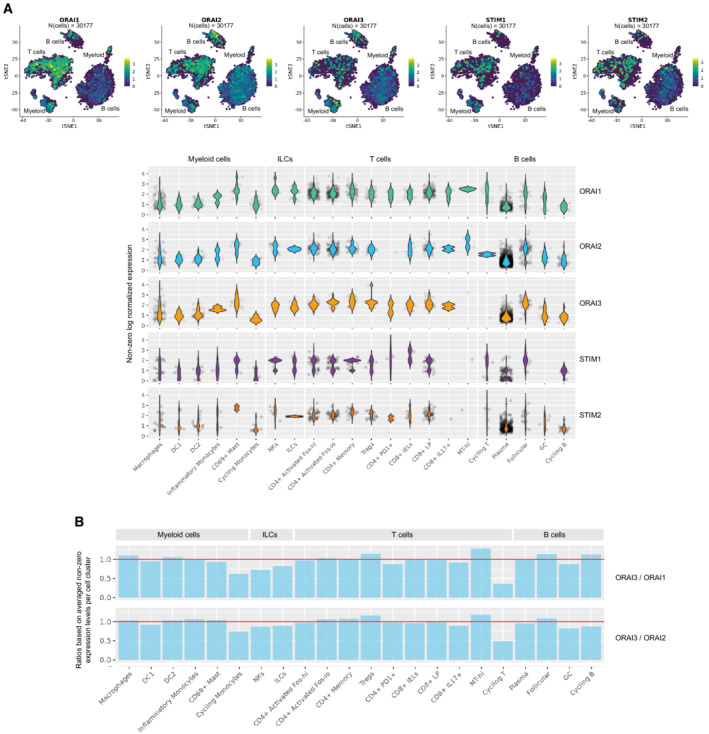
mRNA expression of ORAI and STIM genes in lamina propria mononuclear cells (LPMC) of healthy donors AviSNE plots and violin plots showing mRNA expression levels of ORAI1, ORAI2, ORAI3, STIM1 and STIM2 in subsets of LPMC. Expression data were reanalyzed from Smillie *et al* (2019).BBar graphs showing the ratios of mRNA expression for ORAI3/ORAI1 and ORAI3/ORAI2 in different subsets of LPMC. Expression data were reanalyzed from Smillie *et al* (2019).

To test the effects of SOCE inhibition on the function of non‐T cells in the LP of IBD patients, we stimulated CD45^+^CD3^−^ LPMC isolated from UC and CD patients *ex vivo* with PMA/ionomycin in the presence or absence of BTP2. SOCE inhibition significantly decreased the frequencies of IFNγ^+^CD56^+^ NK cells (cluster 17) and activated HLA‐DR^+^CD40^+^ B cells (cluster 5) in CD, but not in UC patients. No notable effects of SOCE inhibition on other cell clusters, including B cells, NK cells, and myeloid cells, were observed (Figs 5B and C, and EV2). In analogy to our analyses of CD3^+^ LPMC, we next analyzed how BTP2 treatment affects the expression of cytokines and cell surface molecules by CD3^‐^ non‐T cells in the LP. Several subsets of activated CD19^+^HLA‐DR^+^ B cells (clusters 4, 5, and 6) had a strong reduction of IL‐6 in samples of UC and CD patients treated with BTP2 (Fig 5D). In UC patients, the production of TNF, IL‐2, and IL‐17A and the expression of CD40L and FasL were significantly reduced in ILCs (cluster 16) after exposure to BTP2 (Fig 5D). UC patients also had decreased levels of CCR7, CD27, and CD137 in ILC3 cells (cluster 15). In CD patients, BTP2 suppressed the production of TNF, IL‐2, and IFNγ as well as the expression of FasL by NK cells (cluster 18). Inhibition of SOCE furthermore significantly reduced the expression of IFNγ by myeloid cells of CD patients (Fig 5D). By contrast, the activation markers CD25, CD137, CD86, and CD137 were significantly upregulated on CD19^+^ B cells (cluster 5) after BTP2 treatment. Taken together, inhibition of SOCE suppressed the expression of several inflammatory cytokines including IL‐6 (B cells), TNF (ILC and NK cells), and IFNγ (myeloid and NK cells) in IBD patients, suggesting that SOCE contributes to the pro‐inflammatory milieu in the LP of IBD patients generated by myeloid cells and ILCs. Compared to the pronounced effects of BTP2 on CD3^+^ T cells (Fig 4), the inhibition of SOCE had more moderate effects on CD3^−^ LPMC, suggesting that Ca^2+^ signals are more important for intestinal T cells than non‐T cells. Because BTP2 was reported to mainly act through the inhibition of the CRAC channel subunits ORAI1 and ORAI2, and only to a lesser extent through ORAI3 (Zhang *et al*, 2020), we investigated if CD3^−^ and CD3^+^ LPMCs express different ORAI channel subunits with the hypothesis that higher ORAI3 expression in CD3^−^ LPMCs renders them more resistant to BTP2 treatment than T cells. By analyzing scRNA‐seq data sets of intestinal LPMCs obtained from healthy individuals (Smillie *et al*, 2019), we found that the majority of CD3^−^ LPMCs including macrophages, dendritic cells (DC), B cells, and plasma cells have similar expression ratios of ORAI3/ORAI1 and ORAI3/ORAI2 compared to CD3^+^ LPMCs (Fig EV3). Reduced susceptibility to SOCE inhibition of myeloid cells, B cells, and NK cells compared to intestinal T cells therefore is, in our opinion, not likely to be due to differential expression of ORAI subunits.

### SOCE inhibition does not affect the homeostasis of primary murine and human intestinal epithelial cells

**Figure 6:**
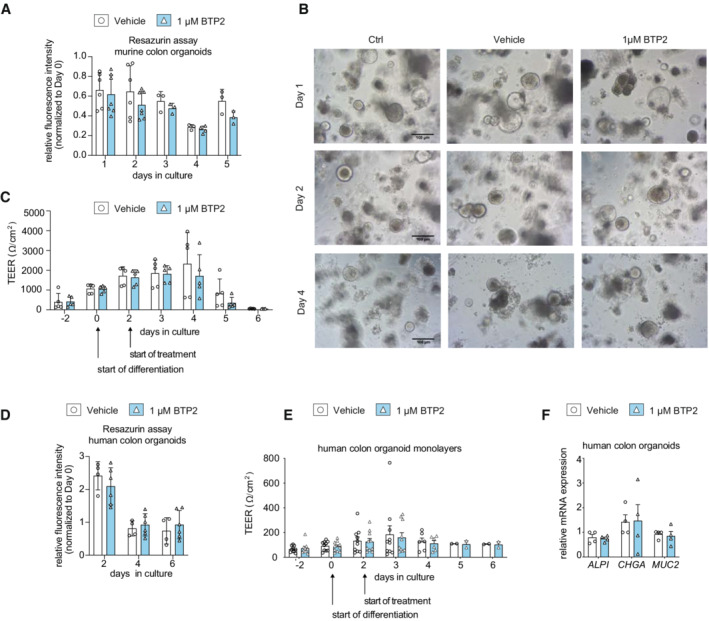
Inhibition of SOCE does not impair the viability, differentiation, or function of intestinal epithelial cells A, BAdult stem cell–derived intestinal epithelial spheroids of wild‐type C57BL/6 mice were cultured for 4 days in Wnt‐free medium to induce differentiation. Initial viability was determined on day 0. DMSO control (vehicle, white bars) or 1 μM BTP2 (blue bars) were added to the culture medium and viability was measured every 24 h by resazurin viability assay and microscopy. Scale bar depicts 100 μm.CTransepithelial electrical resistance (TEER) was measured on differentiated murine colon monolayers in the presence or absence of 1 μM BTP2.DHuman colonic spheroids were generated from primary human epithelial crypts obtained from colon resectates of two CD patients and cultured for 6 days in Wnt‐free medium to induce differentiation into colonic organoids. Initial viability was determined on day 0. DMSO control or 1 μM BTP2 were added to the culture medium and viability was measured on days 2, 4, and 6.ETEER of differentiated human colon monolayers was analyzed in the presence or absence of 1 μM BTP2.FRelative mRNA expression of markers for enterocytes (*ALPI*), goblet cells (*MUC2*), and entero‐endocrine cells (*CHGA*) was quantified by RT‐PCR in differentiated human colon monolayers after addition of 1 μM BTP2. Data information: Data bars in (A–D) represent the mean ± SD, while (E, F) represent the mean ± SEM of least two independent experiments with 1–4 replicates. Statistical significance was calculated using a paired *t*‐test with a Holm–Sidak correction for multiple comparisons. Source data are available online for this figure.

**Figure EV4:**
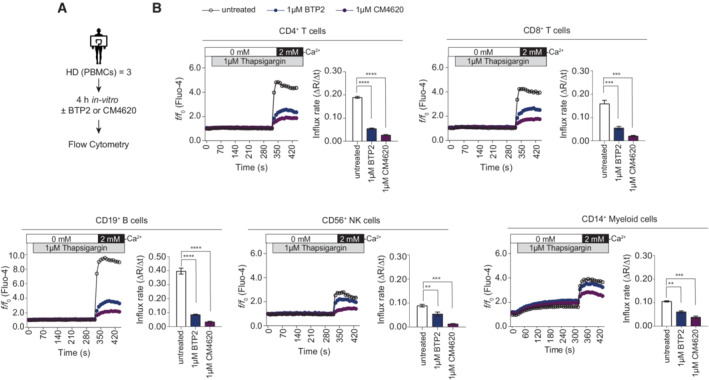
Similar effects of CRAC channel inhibition with CM4620 and BTP2 on Ca^2+^ influx in human immune cell subsets AExperimental design for Ca^2+^ influx measurements in PBMCs of three healthy donors (HD) by flow cytometry.BCa^2+^ influx rates were measured in CD4^+^ or CD8^+^ T cells, CD19^+^ B cells, CD56^+^ NK cells and CD14^+^ myeloid cells following pre‐incubation with 1 μM BTP2 or 1 μM CM4620 for 4 h. Cells were stimulated with thapsigargin in Ca^2+^ free buffer followed by addition of 2 mM Ca^2+^ Ringer solution. Bar graphs show the mean values of influx rates after addition of 2 mM Ca^2+^ from one experiment (samples of *n* = 3 HD run in technical triplicates). Statistical significance was calculated using repeated measures (RM) one‐way ANOVA test. ****P < 0.0001; ***P < 0.001; **P < 0.01; *P < 0.05. Source data are available online for this figure.

**Figure EV5:**
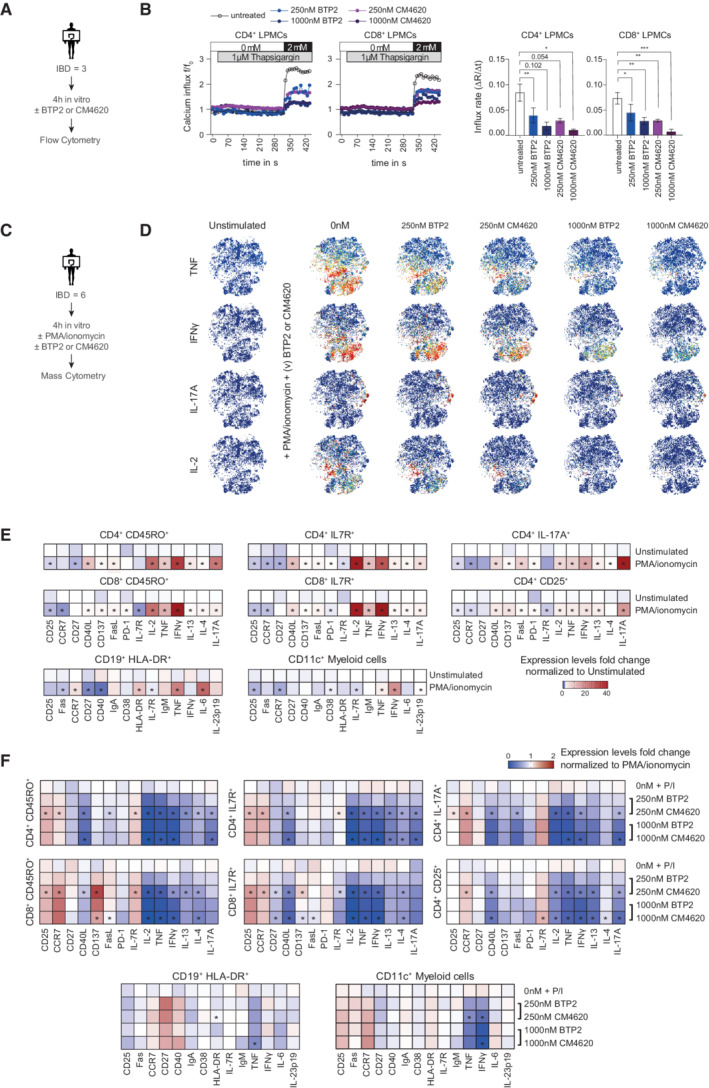
Suppression of cytokine production in LPMCs of IBD patients by CM4620 AExperimental setup for Ca^2+^ influx measurements of LPMCs from three IBD patients by flow cytometry.BHuman lamina propria CD4^+^ and CD8^+^ T cells were pretreated with 250 nM or 1,000 nM BTP2 or CM4620 for 4 h *in vitro* and added to the cell suspension until acquisition. Cells were stimulated with 1 μM thapsigargin (TG) in Ca^2+^ free Ringer solution followed by the readdition of 2 mM Ca^2+^ Ringer solution. Bar graphs show the mean ± SEM of Ca^2+^ influx rates after the addition of 2 mM Ca^2+^ Ringer solution from one experiment (*n* = 3 IBD patients). Statistical significance was calculated with repeated measures (RM) one‐way ANOVA test. ****P < 0.0001; ***P < 0.001; **P < 0.01; *P < 0.05.C–FMass cytometry analysis of LPMCs from six IBD patients. (C) Experimental design. (D) viSNE plots of CD45^+^CD3^+^ LPMCs stimulated with PMA/ionomycin in the presence of various (v) concentrations (250 or 1,000 nM) of BTP2 or CM4620 with unstimulated samples serving as controls. Colors indicate the expression level of IL‐2, IL‐17, IFNγ, and TNFα (blue: low, red: high) and are representative of one CD patient. (E) Heatmaps representing the median fold change of cytokines and cell surface molecules on CD45^+^CD3^+^ or CD45^+^CD3^−^ LPMCs that were stimulated with PMA/ionomycin for 4 h *in vitro*. Data are normalized to unstimulated samples. (F) Heatmaps representing the median fold change of cytokine and cell surface marker expression in CD45^+^CD3^+^ and CD45^+^CD3^−^ LPMCs stimulated with PMA/ionomycin for 4 h in the presence or absence of 250 nM or 1,000 nM BTP2 or 250 nM or 1,000 nM CM4620. Data were normalized to samples treated with PMA/ionomycin alone. Statistical significance in (E, F) was calculated using a one‐tailed paired Wilcoxon matched‐pairs signed rank test. *P < 0.05. Source data are available online for this figure.

After establishing that suppression of SOCE in mouse T cells and human LPMC attenuates intestinal inflammation (Figs 1, 2, EV1, 3, 4, 5), we investigated how SOCE inhibition affects intestinal epithelial cells (IECs). Although IECs are essential for intestinal barrier function, limited information is available about the physiological role of CRAC channels in IEC function (Timmons *et al*, 2012). To investigate how SOCE inhibition affects the survival, differentiation, and function of IEC, we isolated colonic crypts from C57BL/6 wild‐type mice, which were cultured *ex vivo* into colonic organoids or 2D organoids monolayers. Treatment of murine IEC‐derived organoids with 1 μM BTP2 did not significantly impact their viability (Fig 6A and B). Moreover, the transepithelial resistance of primary murine colonic epithelial cells that were grown in 2D layers did not change after treatment with 1 μM BTP2 compared to DMSO‐treated controls (Fig 6C). To validate these results in human IEC, we isolated primary human epithelial crypts from surgical colon specimen of CD patients and cultured them into organoids and 2D monolayers. Treatment of human IEC organoids with 1 μM BTP2 did not affect their viability (Fig 6D). Similar to our findings in murine IEC, BTP2 did not alter the transepithelial resistance of human IEC grown in 2D layers (Fig 6E). Likewise, the differentiation of human IEC was not influenced by BTP2 treatment as no significant differences in the mRNA expression of IEC differentiation markers ALPI, MUC2, and CHGA were observed compared to DMSO‐treated controls (Fig 6F). Taken together, these data indicate that the same concentrations of 1 μM BTP2 that are sufficient to inhibit the activation and pro‐inflammatory functions of LPMCs do not interfere with the differentiation, viability, and barrier function of IEC *in vitro*.

### Pharmacological inhibition of SOCE ameliorates IBD and suppresses proinflammatory cytokine production by T cells

**Figure 7:**
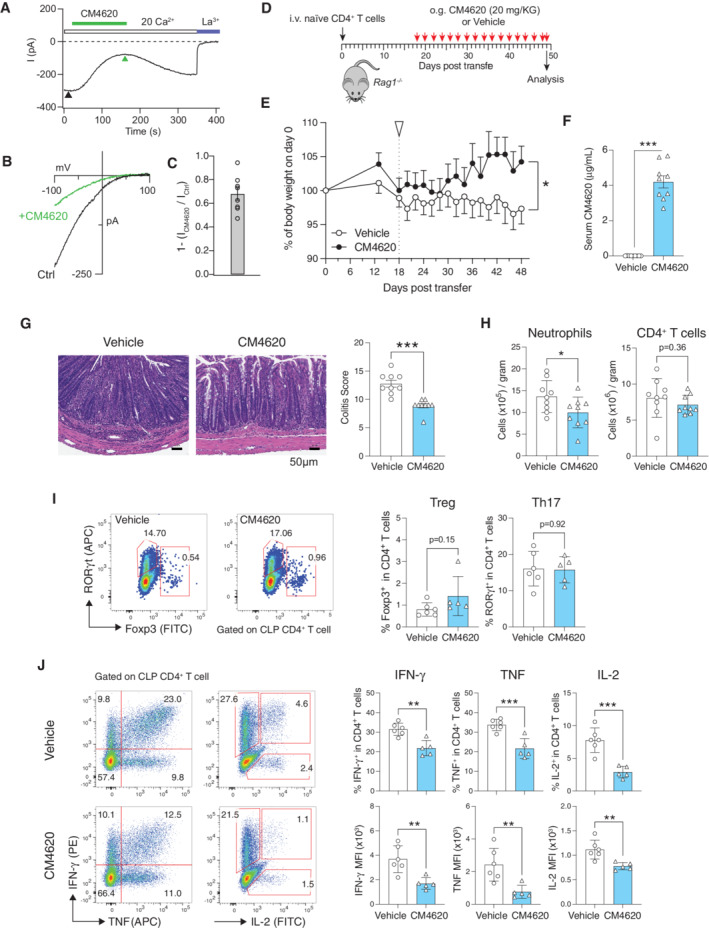
Systemic CRAC channel inhibition alleviates colon inflammation in mice A–CAcute administration of the CRAC channel blocker CM4620 inhibits CRAC currents (I_crac_). (A) Time course of peak amplitudes of I_CRAC_ before and after application of 3 μM CM4620 to HEK293 cells transfected with ORAI1 and STIM1. (B) Current–voltage (I–V) relationship of I_CRAC_ before and after CM4620 treatment. I–Vs were obtained at the time points indicated by the arrowheads in (A). (C) Fractional blockade of I_CRAC_ by CM4620 was measured by comparing current amplitudes before and after CM4620 administration (arrowheads) using the formula 1 − (I_CM4620_/I_Ctrl_). Data are from eight cells per conditions and shown as the mean ± SEM.DIBD was induced by i.v. injection of 5 × 10^5^ naïve CD4^+^ T cells into *Rag1* ^*−/−*^ host mice followed by oral gavage of mice with 20 mg/kg CM4620 or vehicle (SDD) every other day from days 18–49.ERelative weight loss of mice treated with vehicle or CM4620; the start of treatment is indicated by the white arrow. Data are the mean ± SEM of nine mice per cohort, statistical analysis by unpaired Student's *t*‐test, *P < 0.05.FConcentrations of CM4620 in the serum of mice at day 49, data are the mean ± SEM. Statistical analysis by unpaired Student's *t*‐test, ***P < 0.001.GRepresentative H&E staining of the distal colon of *Rag1* ^*−/−*^ mice treated with vehicle or CM4620. Colitis scores of nine mice per cohort. Each symbol represents one mouse. Statistical analysis by Mann–Whitney *U* test Data are the mean ± SEM, ***P < 0.001.HNumber of CD11b^+^Gr‐1^+^ neutrophils and CD4^+^ T cells (normalized to grams of tissue) in the colon lamina propria (CLP) of mice on day 49 quantified by flow cytometry. Each dot represents one mouse. Data are the mean ± SEM of nine mice per cohort.IFrequencies of RORγt^+^ Th17 cells and Foxp3^+^ Treg cells in the CLP of mice treated with CM4620 or vehicle. Each dot represents one mouse. Data are the mean ± SEM of six control and five CM4620 treated mice.JFrequencies of IFN‐γ, TNF‐α, and IL‐2 producing CD4^+^ T cells isolated from the CLP, restimulated *ex vivo* with PMA and ionomycin for 4 h (without addition of CM4620 during stimulation *in vitro*) and analyzed by flow cytometry. Bar graphs represent the percentages of IFN‐γ^+^, TNF‐α^+^, IL‐2^+^ cells in CD4^+^ cells (top row) and mean fluorescent intensity (MFI) of IFN‐γ, TNF‐α, IL‐2 on CD4^+^ cells (bottom row). Each dot represents one mouse. Data are the mean ± SEM of six control and five CM4620 treated mice. Data information: Statistical analyses in panels (H–J) by an unpaired Student's *t*‐test: ***P < 0.001 **P < 0.01 *P < 0.05. Source data are available online for this figure.

Because of the potent suppressive effects of SOCE inhibition with BTP2 on the expression of proinflammatory cytokines and activation markers by human LPMCs and the reduced ability of *Orai1* and *Stim1*‐deficient T cells to induce IBD in mice, we investigated if systemic treatment of mice with a selective CRAC channel inhibitor alleviates intestinal inflammation in mice. In contrast to the adoptive transfer of *Orai1* and *Stim1*‐deficient T cells into *Rag1* ^*−/−*^ mice, in which T cells lack SOCE from the start of the experiment, injection of mice with a CRAC channel inhibitor starting 18 days after T cell transfer assesses whether SOCE inhibition is able to attenuate the progression of established disease. For these experiments we used the CRAC channel blocker CM4620 that, like BTP2, selectively inhibits CRAC channels (I_CRAC_) (Zitt *et al*, 2004; Waldron *et al*, 2019). CM4620 has been used in a phase 2 clinical trial for COVID‐19 pneumonia (NCT04345614), in which it was reported to be safe and well‐tolerated with strong signals in both time to recovery and all‐cause mortality (Bruen *et al*, 2022) and in an ongoing phase 1/2 clinical trial for acute pancreatitis (NCT04195347). To confirm inhibition of CRAC channels by CM4620, we overexpressed STIM1 and ORAI1 in HEK293 cells to enhance I_CRAC_ as reported (Peinelt *et al*, 2006). Acute treatment of cells with 3 μM CM4620 resulted in strong (~ 70%) inhibition of I_CRAC_ without apparent changes to the biophysical properties of the channel (Fig 7A–C). We also compared the effects of CM4620 on SOCE in human PBMCs with those of BTP2 used in earlier experiments (Figs 2, EV1, 3, 4, 5). To this end, we treated human PBMCs from three healthy donors with 1 μM CM4620, 1 μM BTP2 or DMSO as a control for 4 h *in vitro*. CM4620 inhibited SOCE in CD4^+^ and CD8^+^ T cells, NK cells, B cells, and myeloid cells to a similar or slightly greater extent as BTP2 (Fig EV4). Moreover, we found that 250 nM and 1 μM CM4620 suppressed SOCE in intestinal CD4^+^ and CD8^+^ T cells isolated from the LP of IBD patients to a comparable or greater degree as equimolar concentrations of BTP2 (Fig EV5A and B). Accordingly, the production of pro‐inflammatory cytokines by T cells, B cells, ILCs, and myeloid cells isolated from the LP of IBD patients was strongly suppressed by both CM4620 and BTP2 in a dose‐dependent manner (Fig EV5C–F). These findings confirm our previous mass cytometry data using BTP2 and indicate that CM4620 is a selective as a CRAC channel blocker that is at least as/ or more potent compared to BTP2.

To investigate the effects of systemic CM4620 treatment on IBD progression and severity, we injected lymphopenic *Rag1* ^*−/−*^ mice with naïve CD4^+^ T cells from wildtype mice followed by gavage of host mice with CM4620 or vehicle (Fig 7D). *Rag1* ^*−/−*^ mice treated with vehicle alone had progressive weight loss, whereas CM4620 treatment resulted in moderate weight gain (Fig 7E). Serum concentrations of CM4620 were confirmed by LC–MS at the end of the treatment period and ranged from 2.70–5.67 μg/ml (Fig 7F). Systemic treatment with CM4620 significantly ameliorated intestinal inflammation compared to controls (Fig 7G). We observed significantly lower numbers of neutrophils in the colon lamina propria (CLP) of CM4620‐treated mice, whereas numbers of CD4^+^ T cells were not reduced (Fig 7H). Further characterization of CD4^+^ T cell subsets in the LP revealed that treatment with CM4620 did not result in decreased frequencies of RORγt^+^ Th17 cells and Foxp3^+^ Treg cells compared to control mice receiving vehicle only (Fig 7I). We next tested the function of CD4^+^ T cells isolated from the CLP and re‐stimulated them *ex vivo* with PMA/ionomycin for 4 h in the absence of CM4620. The frequencies of CD4^+^ T cells producing IFNγ, TNF, and IL‐2 and the cytokine expression levels in the CLP of mice treated with CM4620 were significantly reduced compared to vehicle‐treated mice (Fig 7J). Impaired IL‐2 production was also observed after re‐stimulation of CLP T cells with anti‐CD3/CD28 for 24 h (Appendix Fig S10). Reduced production of IFN‐γ, TNF, and IL‐2 is consistent with the suppressive effects of SOCE inhibition on CD4^+^ T cells isolated from the LP of CD and UC patients (Fig 4D). Not all cytokines were affected by SOCE inhibition because the frequencies of IL‐17A^+^IFN‐γ^+^ and IL‐10 producing CD4^+^ T cells from the CLP of CM4620‐treated host mice were normal after *ex vivo* restimulation (Appendix Fig S10A and B). Collectively, our data demonstrate that SOCE inhibition with CM4620 after the onset of IBD is well‐tolerated by mice and effective in attenuating intestinal inflammation. The effects of CM4620 are mediated, at least in part, by the suppression of pro‐inflammatory cytokine production, whereas the frequencies of Treg cells in the CLP and IL‐10 production by CLP T cells are preserved.

## Discussion

We here characterize the immune cell composition in the colon LP of patients with UC and CD by mass cytometry and show that SOCE regulates the function of immune cells driving IBD pathology. We also demonstrate that inhibition of SOCE, by genetic deletion of CRAC channel genes or pharmacological inhibition, attenuates intestinal inflammation in mouse models of colitis in a dose‐dependent manner. By contrast, SOCE inhibition does not affect the function and differentiation of primary murine and human intestinal epithelial cells *in vitro*. Together, our data demonstrate that SOCE is a driver of pro‐inflammatory immune cell function in IBD and suggest that inhibition of SOCE may represent a new approach for the treatment of CD and UC patients.

We used mass cytometry to characterize the immune cell composition in the colonic LP of therapy‐refractory UC and CD patients compared with non‐inflamed controls. Shared hallmarks of altered T cell composition in both IBD patient cohorts were an increased abundance of CD4^+^CD45RO^+^ effector CD4^+^ T cells and naïve CD8^+^ T cells, whereas the frequencies of CD4^+^CD45RO^+^IL‐7R^+^ effector memory T cells and CD8^+^ effector T cells were decreased. The overall frequencies of Treg cells were increased in the colonic LP of both UC and CD patients, with a significantly larger percentage of Treg cells producing TNF and IL‐17A. These findings are in line with published scRNA‐Seq data that show a higher expression of inflammatory cytokines in Treg cells of IBD patients (Mitsialis *et al*, 2020), supporting the notion that intestinal inflammation in IBD is driven at least in part by an imbalance of effector versus regulatory T cells and altered Treg cell properties such as the production of inflammatory cytokines. Surprisingly, IL‐4 and IL‐13 producing Th2 cells were significantly reduced in both UC and CD, suggesting that Th2 cells may not be the main source of these cytokines in IBD, reminiscent of a predominant production of IL‐4 and IL‐13 by NKT cells in allergen‐induced airway hyperreactivity (Akbari *et al*, 2003). In the B cell compartment, we observed a significant increase in the production of IL‐6 and a significant reduction of CD38^+^IgA^+^ cells in both UC and CD patients. The depletion of B cells producing IgA, which is required for the opsonization and neutralization of pathogenic bacteria in the gut, is likely to contribute to IBD in both UC and CD patients, a conclusion that is also supported by the increased frequency of IBD‐like inflammatory enteropathies in patients with common variable immunodeficiency and IgA deficiency (Shulzhenko *et al*, 2018; Rizvi *et al*, 2020).

Besides these commonalities between both IBD patient cohorts, UC and CD patients also featured several entity‐specific differences in their LPMC composition: The colonic LP of UC patients, but not that of CD patients, was infiltrated with PD‐1^+^CD4^+^ and PD‐1^+^CD8^+^ effector T cells, which points to a chronic activation of these T cells, potentially due to translocation of intestinal antigens into the LP. Expression of the inhibitory receptor PD‐1 on antigen‐activated T cells in UC patients might represent an attempt to maintain immune tolerance and to limit excessive T cell responses. This conclusion is supported by a significantly higher rate of adverse gastrointestinal effects including diarrhea and colon perforation in 41% of IBD patients receiving checkpoint inhibitors compared to 11% in non‐IBD patients (Abu‐Sbeih *et al*, 2020). Our finding of increased frequencies of PD‐1^+^ T cells in the CLP of UC patients provides a potential explanation for this clinical observation and cautions against the use of checkpoint inhibitors such as nivolumab or pembrolizumab in IBD patients. Also consistent with the idea of a partially exhausted T cell compartment in UC patients due to chronic GI inflammation is our finding that the production of IFNγ and IL‐6 and the expression of CD103 (ITGAE) (Mokrani *et al*, 2014) were reduced in effector and effector memory CD4^+^ T cells of UC patients compared to T cells of non‐inflamed controls and CD patients. In comparison to UC patients, the immune compartment in the LP of CD patients was characterized by higher frequencies of different clusters of CD38^+^ B cells expressing IgM or IgA as well as IFNγ‐producing NK cells and IL7R‐expressing CD4^+^ and CD8^+^ memory T cells.

The production of inflammatory cytokines by immune cells in the LP is an important pathogenic driver of intestinal disease in UC and CD. In CD, IFNγ and IL‐17/IL‐22 generated by Th1 and Th17 cells, respectively, were described early on as cytokines potentially involved in disease pathogenesis, whereas the development of UC has conventionally been linked to high levels of IL‐4 and IL‐13 produced by T cells and NKT cells (Strober & Fuss, 2011). It is noteworthy that our mass cytometry data did not find a predominance of IL‐4 and IL‐13 in the LP of UC patients, but we instead mainly detected cells producing IFNγ, TNF, and IL‐6 that are known to be present in both forms of IBD (Strober & Fuss, 2011). Intriguingly, we did not observe significant differences in the expression levels of these pro‐inflammatory cytokines on a single‐cell basis in effector or memory T cells isolated from surgical colon specimen of CD and UC patients when compared to non‐inflamed controls, but we did note a higher frequency of cells producing IFNγ and TNF in CD and UC patients. Only Treg cells isolated from inflamed lesions of CD and UC patients showed significantly increased expression levels of IL‐17 and TNF relative to Treg cells from non‐inflamed control patients. It is possible, however, that differences in the levels of cytokine production by LPMCs from inflamed and non‐inflamed regions were masked by the strong stimulus (PMA plus ionomycin) we used to induce cytokine production.

The expression of the inflammatory cytokines such as IFNγ, TNF, and IL‐17A is regulated by Ca^2+^ dependent transcription factors such as NFAT (Vaeth & Feske, 2018b). Given the important role of these cytokines in IBD pathogenesis, we investigated how inhibition of SOCE as the main Ca^2+^ influx pathway after antigen receptor stimulation of immune cells affects cytokine production. Using the CRAC channel inhibitors BTP2 and CM4620, we here demonstrate that SOCE is required for the production of many inflammatory cytokines including IL‐2, IL‐4, IL‐17, TNF, and IFNγ by T cells and, although to a lesser degree, B cells and myeloid cells. Increasing concentrations of BTP2 and CM4620 resulted in a dose‐dependent suppression of cytokine production in CD4^+^ and CD8^+^ effector and effector memory T cells, Th17 cells, as well as Treg cells isolated from the LP of IBD patients. In human LP T cells, the most Ca^2+^ dependent cytokines were IL‐2 and IFNγ, followed by TNF and IL‐17, whereas the Th2 cytokines IL‐4 and IL‐13 were less sensitive to BTP2 or CM4620‐mediated SOCE inhibition. When we compared the effects of BTP2 on cytokine production by a variety of colonic T cell subsets, we observed significantly reduced frequencies of IL‐2, IL‐4, IL‐13, IL‐17, TNF and IFNγ producing T cell subsets in both CD and UC patients. The suppressive effects of SOCE inhibition on cytokine expression in T cells of IBD patients are consistent with a report of decreased production of IFNγ, IL‐2, and IL‐17A by LPMCs of IBD patients that had been stimulated *ex vivo* in the presence of the SOCE inhibitor Synta 66 (Di Sabatino *et al*, 2009). Significant suppression of cytokine production by BTP2 was also observed for TNF, IFNγ, and IL‐2 in Treg and NK cells of CD patients, and for TNF, IL‐2, and IL‐17A in ILCs of UC patients. BTP2 also blocked the production of TNF, IL‐2, and IL‐6 by B cells of both UC and CD patients, whereas the effects of SOCE inhibition in myeloid cells were limited to reduced IFNγ production in CD patients. These broad effects of SOCE inhibition on the production of pro‐inflammatory cytokines by T cells, and to a somewhat lesser degree also B cells, ILCs and myeloid cells, of UC and CD patients emphasize the important role of SOCE in the transcriptional regulation of these cytokines and the potential of CRAC channel blockers for the treatment of IBD.

To investigate whether the suppressive effect of SOCE inhibition on the *ex vivo* production of pro‐inflammatory cytokines by LPMCs of UC and CD patients translates into attenuated IBD severity *in vivo*, we used a murine T cell transfer model of IBD in combination with either genetic or pharmacological inhibition of CRAC channels. Transfer of naïve CD4^+^ T cells from *Stim1*‐deficient mice and, to a lesser degree, *Orai1*‐deficient mice attenuated IBD severity, whereas deletion of *Stim2* had no significant protective effect. The degree to which IBD was attenuated correlated with the degree of reduced SOCE in *Stim1*, *Orai1*, and *Stim2*‐deficient CD4^+^ T cells and the extent of their impaired IFNγ, IL‐17A, and TNF production. The production of IFNγ and IL‐17A was almost abolished in *Orai1*‐deficient T cells despite substantial residual SOCE, demonstrating that incomplete suppression of SOCE is sufficient to strongly attenuate inflammatory cytokine production. Nonetheless, *Orai1*‐deficient T cells retain some residual function because they are able to cause moderate weight loss and intestinal inflammation when transferred to lymphopenic mice. Consistent with reduced pro‐inflammatory cytokine production by SOCE‐deficient T cells isolated from mice with IBD, we also observed that genetic or pharmacologic inhibition of SOCE suppressed the production of IFNγ and IL‐17A in murine Th1 and T17 cells, respectively, *in vitro* in a dose‐dependent manner. By contrast, the expression Foxp3 and CTLA4, which are regulators of Treg cell differentiation and function (Shevach, 2009), were less or not affected by SOCE inhibition. These findings are consistent with previous studies showing that the suppressive function of nTreg and iTreg cells is preserved in mice with conditional deletion of *Orai1* (Jin *et al*, 2013; Kaufmann *et al*, 2016) or *Stim1* (Desvignes *et al*, 2015) in T cells. Taken together, these data indicate that effector Th1 and Th17 cells require strong Ca^2+^ influx for the production of pro‐inflammatory cytokines, whereas moderate SOCE is sufficient for the function of tolerogenic Treg cells. The concept of different SOCE thresholds in pro‐inflammatory effector and regulatory T cells may extent to human immune cells. We observed that the production of IFNγ, IL‐2, and other cytokines was reduced by >50% in CD4^+^ effector and memory T cells of CD and UC patients by sub‐micromolar concentrations of BTP2, whereas 1 μM BTP2 was required to achieve >50% reduction of IL‐17A and TNF production by Treg cells. Moreover, cytokine production by B cells and myeloid cells was not or only moderately affected by SOCE inhibition even with high concentrations of BTP2. Our data suggest that different immune cell subsets have distinct Ca^2+^ optimums for key functions. Cell types such as effector T cells and ILCs that promote inflammation appear to depend on strong SOCE for the production of inflammatory cytokines, whereas tolerogenic Treg cells require less SOCE for their differentiation and function. Understanding the molecular mechanisms controlling these differential SOCE requirements in pro‐ versus anti‐inflammatory T cell subsets requires further study. Nonetheless, our data suggest the existence of a therapeutic window for SOCE inhibition to suppress the function of pro‐inflammatory effector T cells without compromising the tolerogenic function of Treg cells.

To further investigate whether pharmacologic inhibition of CRAC channel function is an effective and safe approach to IBD treatment, we tested the effects of systemic application of the CRAC channel inhibitor CM4620 in a murine adoptive T cell transfer model of IBD. At serum concentrations equivalent to the IC_50_ for inhibition of human T cell function *in vitro*, we observed reduced weight loss, neutrophil inflammation of the LP, and production of IFNγ and TNF by LP T cells, whereas the frequencies of Treg cells and the amount of secreted IL‐10 were not altered in the LP of animals after blockade of SOCE. These results indicate that SOCE inhibition attenuates IBD severity even when treatment is started after disease initiation and administered systemically. This is significant given the fact that SOCE is the source of Ca^2+^ signals in many non‐excitable cell types, including intestinal epithelial cells. To exclude that CRAC channel inhibition has adverse effects on IEC function and the intestinal barrier, thereby exacerbating IBD, we treated primary murine and human colonic epithelial cells grown in organoid cultures with the selective CRAC channel inhibitor BTP2 *in vitro*. Exposure to 1 μM BTP2 over 4 days did not affect the survival and differentiation of IECs or trans‐epithelial resistance, suggesting that SOCE inhibition does not significantly impair IEC and intestinal barrier function *in vitro*. We cannot rule out that SOCE inhibition may be required for the differentiation or function of rare epithelial cell subsets such as tuft cells, goblet cells, or neuroendocrine cells *in vivo*, which play important roles in mucosal immune homeostasis, chemo‐sensing, and tissue regeneration (Birchenough *et al*, 2015; Schneider *et al*, 2019) which were not investigated in this study. Possible side effects of CRAC channel inhibitors on IEC and intestinal barrier function may manifest only after long‐term application, which would be required for the treatment of IBD. Arguing against such a role of SOCE is the fact that patients with loss‐of‐function mutations in *ORAI1* or *STIM1* genes and knock‐out or knock‐in mice lacking functional ORAI1 channels do not show signs of intestinal inflammation under steady‐state conditions (Gwack *et al*, 2008; McCarl *et al*, 2010; Vaeth & Feske, 2018a). Moreover, a recent study reported that the conditional deletion of *Stim1* in IEC had no effect on the differentiation of IECs in *Stim1* ^*fl/fl*^ *Villin‐Cre* mice and did not result in spontaneous intestinal inflammation (Liang *et al*, 2022), suggesting that SOCE in IEC is not required for intestinal barrier function under steady‐state conditions. Another possible side effect of SOCE inhibition could be the development of colorectal cancer (CRC), and patients with UC and CD involving the colon are at higher risk than the general population to develop CRC (Nebbia *et al*, 2020). A recent study reported that calcineurin and NFAT, which are activated by SOCE, contribute to the development of intestinal epithelial tumors by promoting the survival and proliferation of cancer stem cells (Peuker *et al*, 2016). Inhibition of SOCE may therefore provide several benefits to IBD patients by not only attenuating GI inflammation but also by improving mucus production by goblet cells via reducing ER stress–induced apoptosis of goblet cells (Liang *et al*, 2022) and by suppressing the development of CRC. It is noteworthy that although calcineurin inhibition with cyclosporin A and tacrolimus has been used with some success for the treatment of UC (Harbord *et al*, 2017), Crohn's disease patients often do not efficiently respond to calcineurin inhibitors (Feagan *et al*, 1994). By contrast, inhibition of SOCE not only suppresses calcineurin‐NFAT signaling but also blocks additional signaling processes including the PI3K‐AKT–mTOR pathway, NF‐kB signaling, and mitochondrial metabolism (Vaeth *et al*, 2017; Berry *et al*, 2018; Kaufmann *et al*, 2019). Pharmacologic blockade of SOCE may therefore have beneficial effects in IBD patients who are resistant to cyclosporin A treatment. The suitability of SOCE as new drug target in IBD patients is further supported by the fact that SOCE was reported to be increased in intestinal lymphocytes of IBD patients (Schwarz *et al*, 2004), which is confirmed by our own observations in this study (Appendix Fig S11), and the increased expression of STIM1 in CD45^+^ LPMCs in inflamed intestinal tissues of IBD patients (Liang *et al*, 2022).

The intravenous formulation of CM4620, Auxora, has been administered in a Phase 2 trial of patients with severe COVID‐19 pneumonia (NCT04345614) where is was reported to be safe and well tolerated with strong signals in both time to recovery and all cause mortality (Bruen *et al*, 2022). Auxora was also reported to be safe and well tolerated in a Phase 2 trial (NCT03401190) of patients with acute pancreatitis and systemic inflammatory response syndrome (SIRS) (Bruen *et al*, 2021. Phase 2 trials of Auxora are ongoing in patients with critical COVID‐19 pneumonia (NCT04661540) and acute pancreatitis and accompanying SIRS (NCT04681066), as well as a Phase 1/2 trial in children and young adults with asparaginase associated pancreatitis (NCT04195347). These data suggest that SOCE inhibition is well‐tolerated and effective in patients with severe pulmonary and pancreatic inflammation and they are in line with data reported here. In mice, CM4620 was well‐tolerated and reduced IBD severity when administered systemically after disease onset. We also found that CM4620 blocks SOCE in human LPMCs in a dose‐dependent manner, resulting in a significant reduction of TNF, IFNγ, IL‐2, IL‐4, IL‐13, IL‐17A, and CD40L production by human CD4^+^ and CD8^+^ T cells, decreased production of TNF by intestinal B cells, as well as IFNγ by myeloid cells. It is important to note that CRAC channel inhibitors not only suppress cytokine production in LPMCs of IBD patients but also and to a similar extend in LPMCs obtained from non‐IBD patients. Because complete suppression of SOCE in human patients with loss of function mutations in *ORAI1* and *STIM1* genes and mice with abolished SOCE in T cells is prone to severe infections and autoimmunity because of impaired functions of effector T cells and Treg cells, respectively, therapeutic CRAC channel inhibition for IBD would have to be confined to a therapeutic window. The efficacy and safety of long‐term, systemic CRAC channel inhibition in chronic inflammatory diseases such as CD and UC remains to be studied.

## Materials and Methods

### Ethical regulations

Written informed consent was obtained from all patients and healthy donors including the consent to publish clinical information potentially identifying individuals. All experiments involving human material were approved by the institutional review board of the Charité‐ Universitätsmedizin Berlin and were conducted in accordance to the principles set out in the WMA Declaration of Helsinki and the Department of Health and Human Services Belmont Report. All animal experiments were performed in accordance to institutional guidelines for animal welfare approved by the Institutional Animal Care and Use Committee at New York University Grossman School of Medicine.

### Mice

*Stim1* ^*fl/fl*^ *Cd4Cre*, *Stim2* ^*fl/fl*^ *Cd4Cre*, *Stim1* ^*fl/fl*^ *Stim2* ^*fl/fl*^ *Cd4Cre* (Oh‐Hora *et al*, 2008), and *Orai1* ^*fl/fl*^ *Cd4Cre* mice (Kaufmann *et al*, 2016) have been described previously. *Rag1* ^*−/−*^ mice were purchased from The Jackson Laboratory (stock number 002216). Animals were used between 6 and 12 weeks of age. For experiments shown in Fig 1, only female mice were used as donor and recipient mice. For experiments shown in Fig 7, both female and male mice were included in the studies and no mice were excluded. All mice were maintained under specific pathogen‐free conditions and kept under a 12 h light cycle with *ad libitum* access to water and food.

### Isolation of human lamina propria mononuclear cells

Surgical colon resectates were obtained from 13 UC and 11 CD patients undergoing colectomy, while non‐inflamed specimen of six patients with colorectal cancer served as non‐inflamed controls (Appendix Table S1). The number of patients included per group for CyTOF were based on our experience with similar explorative mass cytometry experiments (Ziegler *et al*, 2019). Colonic mucosa was detached using sterile forceps and washed in 1 mM 1,4 Dithiothret (DTT) (Roth) dissolved in Hanks' Balanced Salt solution (HBSS) w/o Ca^2+^ and Mg^2+^ (ThermoFisher Scientific), in shaking conditions at RT for 15 min. Lamina propria was cleaned from any left submucosal tissue, cut in pieces and washed three times in 1 mM ethylenediaminetetraacetic acid (EDTA) (Sigma‐Aldrich) under constant shaking for 15 min at 37°C in order to detach epithelial cells. Afterward, EDTA was carefully washed out using HBSS w/o Ca^2+^ and Mg^2+^ and tissue was subsequently incubated in 0.15 mg/ml collagenase A (ROCHE) for 16 h at 37°C under constant shaking. The supernatant was then filtered through a 100‐μm cell strainer (ThermoFisher Scientific) and washed three times in HBSS w/o Ca^2+^ and Mg^2+^. Cells were separated through a Percoll gradient (GE Healthcare) centrifugation for 30 min at 1,500 rpm at 4°C. Lympho‐monocytes were collected at the interface of 40–60% Percoll. Cells were washed twice and stimulated *ex‐vivo* for 4 h in RPMI 1640 (ThermoFisher Scientific) supplemented with 10% fetal bovine serum (FBS; Sigma) and 1% Penicillin–Streptomycin (ThermoFisher Scientific) containing 20 ng/ml phorbol 12‐myristate 13‐acetate (PMA; Sigma‐Aldrich) and 1 μg/ml ionomycin (Sigma‐Aldrich) in the presence or absence of 0–1,000 nM BTP2 (Sigma Aldrich). Brefeldin A (10 μg/ml; Sigma‐Aldrich) was added for 2 h, while 25KU Benzonase Nuclease (Sigma) was added (1:10,000) 15 min prior to harvesting. For mass cytometry, cells were fixed in Smart Tube buffer (SMART TUBE Inc.) supplemented with 20% BSA (ThermoFisher Scientific) and subsequently stored at −80°C until antibody staining.

### Calcium influx measurements in murine T cells

96‐well imaging plates (Fisher) were coated with 0.01% poly‐L‐lysine (w/v) (Sigma‐Aldrich) for 2 h and then washed with water. Cells were labeled with 2 μM Fura‐2 AM (Life Technologies) for 30 min in cell culture medium and attached for 10 min to the plates. Intracellular Ca^2+^ measurements were analyzed using a Flexstation 3 fluorescence plate reader (Molecular Devices) at 340 and 380 nm excitation wavelengths. Cells were stimulated with 0.3 μM ionomycin (EMD Millipore) in 1 mM Ca^2+^ Ringer solution (155 mM NaCl, 4.5 mM KCl, 3 mM MgCl_2_, 10 mM D‐glucose, 5 mM Na HEPES) to induce SOCE. Fura‐2 fluorescence emission ratios (F340/380) were collected at 510 nm every 5 s. Ca^2+^ signals were quantified by analyzing the peak value of F340/380 ratios using the GraphPad Prism 9.0 software.

### Calcium influx measurements in human LPMCs and PBMCs

Heparinized blood was obtained from healthy donors, and PBMCs were isolated by density gradient centrifugation using Biocoll (Merck). Alternatively, surgical colon specimens were obtained from IBD patients undergoing colectomy and cells were isolated according to the isolation of lamina propria mononuclear cells protocol described above. Isolated PBMCs or LPMCs were cultured for 4 h (Figs 2B, EV4 and EV5) or overnight (Appendix Fig S2A) at 37°C, 5% CO_2_ in the presence or absence of 15–1,000 nM BTP‐2 (Sigma Aldrich) or 250–1,000 nM CM4620 (Sigma Aldrich) dissolved in dimethyl sulfoxide (DMSO) (Sigma). Subsequently, cells were loaded with the calcium sensing dye Fluo‐4 AM/DMSO (2 μg/ml; Life Technology) for 30 min on ice protected from light, washed with PBS/5% BSA (ThermoFisher Scientific) and afterward stained for 15 min on ice with anti‐human CD3 (APC, OKT1, eBioscience) or CD3 (Viogreen, REA613, Miltenyi Biotec), CD4 (BV510, RPA‐T4, Biolegend), CD8 (APC‐Cy7, SK1, Biolegend), CD19 (APC‐Cy7, HIB‐19, Biolegend), CD14 (APC, 63D3, Biolegend), CD16 (Pe‐Cy7, 3G8, BD), and CD56 (APC, TULY56, eBioscience). BTP2 and CM4620 were added to single‐cell suspensions at every step of the staining procedure until flow cytometric acquisition. To measure baseline intracellular Ca^2+^ ([Ca^2+]^i), cells were washed and re‐suspended in 0 mM Ca^2+^ Ringer solution (155 mM NaCl, 4.5 mM KCl, 3 mM MgCl_2_, 10 mM D‐glucose, 5 mM Na‐HEPES) and acquired for 30 s using a Canto II flow cytometer (BD Bioscience). Subsequently, cells were stimulated with 1 μM thapsigargin (EMD Millipore, Billerica, MA) in Ca^2+^ free buffer followed by addition of Ca^2+^ containing Ringer solution (2 mM CaCl_2_) after 300 s to the cells (final [Ca^2+^] 1 mM). For analysis of Ca^2+^ levels, the mean fluorescence intensity (MFI) of Fluo‐4 (*f*) was normalized to the MFI average detected during the 30 s baseline measurement (*f*_0_), and the resulting ratio *f/f*_0_ was plotted against a time (*t*) axis. Graphs were plotted using Prism 9 software.

### Annexin V and 7AAD staining of human LPMCs

Colon resectates were obtained from three IBD patients undergoing colectomy, and cells were isolated according to the isolation of lamina propria mononuclear cells protocol described above. Cells were stimulated *ex vivo* for 4 h in RPMI 1640 (ThermoFisher Scientific) supplemented with 10% fetal bovine serum (FBS; Sigma) and 1% Penicillin–Streptomycin (ThermoFisher Scientific) containing 20 ng/ml phorbol 12‐myristate 13‐acetate (PMA; Sigma‐Aldrich) and 1 μg/ml ionomycin (Sigma‐Aldrich) in the presence or absence of 1 μM BTP2 (Sigma Aldrich). Brefeldin A (10 μg/ml; Sigma‐Aldrich) was added 2 h prior to harvesting. Cells were stained according to the Pacific Blue™ Annexin V Apoptosis Detection Kit with 7‐AAD (Biolegend). Samples were measured using a Canto II flow cytometer (BD Bioscience). Data were analyzed using the FlowJo software package V10.1 (FlowJo, LLC).

### Mass cytometry staining and acquisition

Cells were thawed and barcoded for 30 min at RT by using the Cell‐ID 20‐plex Pd Barcoding Kit (Fluidigm) ± CD45‐89Y staining in order to pool more samples in one batch/run (max 40 samples). Individual samples were washed twice with cell staining buffer (Fluidigm) and pooled together before staining. Anti‐human antibodies were purchased either pre‐conjugated to metal isotopes or conjugated in‐house using the conjugation MaxPar X8 kit (Fluidigm) according to the manufacturer's protocol (Appendix Table S2). For surface staining, cells were incubated with antibodies for 30 min at 4°C, then washed twice with cell staining buffer and incubated for 60 min at 4°C in fixation/permeabilization buffer according to the manufacturer's protocol (Fix/Perm Buffer, eBioscience). Cells were then washed twice using permeabilization buffer (eBioscience) and stained with antibody cocktails against intracellular molecules for 1 h at 4°C. Cells were subsequently washed twice with permeabilization buffer and incubated overnight in 2% methanol‐free formaldehyde solution (ThermoFisher). Cells were washed and re‐suspended in 1 ml iridium intercalator solution (Fluidigm) for 1 h at RT. Finally, cells were washed twice with cell staining buffer and then twice with Maxpar Water (Fluidigm) for CyTOF measurements. Cells were analyzed using a CyTOF2 upgraded to Helios specifications, with software version 6.5.236, as previously described (Böttcher *et al*, 2019). Compensation beads staining: OneComp eBeads™ Compensation Beads (Invitrogen) were stained individually with each of the antibodies included in our panel (Appendix Table S2) for bead‐based compensation of mass cytometry. For each channel assessed in the panel described above, one drop of OneComp eBeads was loaded in a well of a v‐bottom 96‐well plate (Corning) and individually stained with 1 μg of the corresponding metal‐labeled antibody for 30 min at room temperature. After staining, individual beads were washed three times in staining buffer and afterward pooled in a single tube and washed once in PBS (ThermoFisher Scientific). Beads were then fixed in 1.6% methanol‐free formaldehyde solution (ThermoFisher) for 1 h at room temperature. After fixation, beads were washed twice in staining buffer and twice in Maxpar water. Beads were re‐suspended in 300 μL of Maxpar water previous CyTOF acquisition.

### Data analysis of mass cytometry data

The Cytobank software package (www.cytobank.org) was used for initial manual gating on single cells and for de‐barcoding of pooled samples. FCS files of de‐barcoded single‐cells were exported and further compensated for signal spillover using the R Software (3.6.0, Bioconductor 9) and by applying the *CATALYST* package and arcsinh transformation (scale factor 5) prior to data analysis. Compensated files were then gated on CD45^+^, CD45^+^CD3^+^ or CD45^+^CD3^−^ cells, and t‐SNE maps of each pre‐gated cell population were generated according to the expression of markers listed in Appendix Tables S3–S6 by using the Cytobank Software. Subsequently, FCS files containing the t‐SNE embedding as additional two parameters were exported from Cytobank and the clustering analysis was performed using the *FlowSOM/ConsensusClusterPlus* package on R (3.6.0, Bioconductor 9) (Appendix Fig S7). Cell clusters of LPMCs were identified after visual inspection of t‐SNE plots and functional interpretation of heat maps generated by *FlowSOM/ConsensusClusterPlus*. Statistical significance of differential abundant clusters (DA) among the three disease groups (UC, CD, and Non‐inflamed) was performed using a generalized linear mixed‐effects model (GLMM) available through the R package *diffcyt* (used all defaults with analysis_type = “DA”, method_DA = “diffcyt‐DA‐GLMM”, min‐cells = 3). False discovery rate (FDR) was adjusted at 10% using the Benjamini–Hochberg (BH) procedure for multiple hypothesis testing, as previously described (Bottcher *et al*, 2019). To test differences in protein expression among disease groups multiple *t* tests with FDR adjusted at 1% using the Benjamini, Krieger and Yekutieli procedure were applied, while the effects of SOCE‐inhibitors BTP2 and CM4620 on LPMCs isolated from UC or CD patients were calculated using a one‐tailed paired Wilcoxon matched‐pairs signed‐rank test.

### Data analysis of publicly available scRNA‐seq data

Pre‐processed scRNA‐seq data of LPMCs derived from 34 colon biopsies of 10 healthy donors published by Smillie *et al* (2019) were downloaded from the Broad Institute Single Cell Portal (SCP259). Feature expression measurements of individual cells were normalized by the total expression, multiplied by a scale factor of 10.000 and results log‐transformed using the Seurat v4.1.1 package (Hao *et al*, 2021) in R v4.1.3. Non‐zero normalized expression levels of cells were extracted from the feature expression matrix preserving original cell type annotation and tSNE coordinates. Differences in cell‐type and disease‐state specific expression levels were tested for significance using nonparametric Kruskal–Wallis rank sum tests followed by pairwise Wilcoxon rank sum tests corrected for multiple testing using Benjamini–Hochberg (Benjamini *et al*, 2001). Differential gene expression analysis was performed globally using the *Seurat FindMarkers* function (Satija *et al*, 2015); log_2_‐fold changes of features were adjusted for cellular detection rates and tested for significance using the *MAST* package hurdle model (Finak *et al*, 2015).

#### Human and murine epithelial cell acquisition

Intestinal tissue was obtained from colon resectates of two CD patients and colon crypts were isolated from non‐inflamed areas of the ascending colon. Intestinal tissue was washed and cut into smaller pieces (approx. 3 mm) after removing underlying muscle layers with surgical scissors. For murine samples, *C57BL/6* mice between 8 and 15 weeks were euthanized, and the colonic part of the gut was removed.

#### Crypt Isolation and culture

Establishment and maintenance of murine and human spheroid cultures from the colon epithelium were obtained as described previously (Sato *et al*, 2011; Miyoshi & Stappenbeck, 2013; van de Wetering *et al*, 2015). In brief, colons from C57B/6 wild‐type mice were opened longitudinally, cut into small pieces, and washed with cold phosphate‐buffered saline (PBS; Gibco; Life Technologies, Carlsbad, USA). Human intestinal mucosa was isolated by removing the muscle and fat tissue from colon resectates. The tissue was then cut into small (1–2 mm) stripes and washed with cold PBS. Intestinal fragments were washed repeatedly with cold PBS until the supernatant was clear. The tissue was then incubated with 10 mM EDTA cold chelation buffer (PBS supplemented with 54.9 mM D‐sorbitol, 43.4 mM sucrose, and 1 mM 1,4‐dithiothreitol (DTT; Carl Roth, Karlsruhe, Germany) and an antibiotic cocktail containing ciprofloxacin (10 μg/ml; Fresenius Kabi, Bad Homburg, Germany), fluconazol (1 μg/ml; B. Braun, Melsungen, Germany), primocin (100 μg/ml; InvivoGen, San Diego, USA) and gentamycin (50 μg/ml, Ratiofarm, Ulm, Germany)) for 45 min on ice with gentle shaking. EDTA‐containing supernatant was removed, 10 ml chelation buffer (without DTT and antibiotics) was added and crypts were released from the tissue by vortexing four times for 30 s. Supernatant of every vortexing step was pooled and washed (300 *g*, 4°C, 5 min) two times with 10 ml washing medium (advanced DMEM/F‐12; Gibco, Life Technologies, Carlsbad, USA) supplemented with 2 mM L‐glutamine, 50 units/ml penicillin, and 50 μg/ml streptomycin, 10 mM HEPES (Gibco). Crypts were transferred to a 1.5‐ml microtube followed by seeding.

Isolated intestinal crypts were counted, embedded in Matrigel (Corning, NY, USA; growth factor reduced, phenol red‐free, LDEV‐free) on ice, and seeded into 24‐well plates (500 crypts in 50 μl Matrigel per well). Matrigel was polymerized for 15 min at 37°C and overlaid with 500 μl of spheroid culture medium (50% L‐WRN‐CM2 [conditioned medium produced using the cell line L‐WRN CRL‐3276, ATCC, Manassas, USA]) in advanced DMEM/F‐12 supplemented with 20% fetal bovine serum, 2 mM L‐glutamine, 50 units/ml penicillin, and 50 μg/ml streptomycin (Merck Millipore, Burlington, USA) (and additional supplements specific for mouse and human [Mouse: 50 ng/ml recombinant murine EGF (Peprotech, Hamburg, Germany). Human: 50 ng/ml recombinant human EGF (Peprotech), 10 mM nicotinamide (Sigma, St. Louis, USA), 0.5 μM A83‐01, 10 μM SB202190, 10 nM human gastrin I] (all from Sigma)). 10 μM Y‐27632 (Abmole Bioscience, Houston, USA) was added to the culture medium for the first 2 days of culture after isolation or passaging. Fresh culture medium was added every other day, and spheroids were passaged once a week at a 1:6 splitting ratio. For passaging, Matrigel was scraped off to collect organoids in a 15‐ml conical tube. Organoids were digested into single cells in 1 ml TrypLE (Gibco) for 5 min at 37°C, embedded in Matrigel and seeded into a 24‐well plate.

#### Resazurin viability assay

Spheroids were passaged, embedded in Matrigel and seeded into 96‐well plates at a density of approx. 100 organoids in 8 μl Matrigel per well. Organoids were cultured in spheroid culture medium for 5 days. 10 μM Y‐27632 was added for the first 2 days of culture. To induce differentiation, WRN‐CM, nicotinamide, and SB202190 were withdrawn from the medium and 100 ng/ml recombinant noggin (Peprotech), 500 ng/ml recombinant human R‐Spondin (R&D Systems, Wiesbaden, Germany) and 5 μM DAPT (Selleckchem, Houston, USA) were added as described previously (Sato *et al*, 2011). Initial organoid viability was assessed with resazurin sodium salt (Sigma) once before changing to differentiation medium. Resazurin was added to every well at a working concentration of 0.25 μg/ml, and the culture was incubated in the dark at 37°C for 1 h. Subsequently, the culture medium was transferred to a black flat clear‐bottom 96‐well plate and fluorescence was measured at 560 nm excitation and 590 nm emission with a SpectraMax Gemini EM Microplate Reader (Molecular Devices, San Jose, USA). Wells with organoid culture were washed with PBS, and fresh medium was added. Culture was continued at 37°C, and viability was measured with resazurin as described above on the indicated days of culture. The medium was supplemented with 1 μM BTP2 starting from day 2 of differentiation.

#### Establishment of 2D‐monolayers and treatment with BTP2 and TNFα and IFNγ

Organoid‐derived monolayers were generated based on published protocols (Wang *et al*, 2019; Holthaus *et al*, 2020). Briefly, the upper compartment of a 0.6 cm^2^ trans‐well filter (Corning; PCF; 0.4 μm pores) was coated with 150 μl Matrigel mixed 1:10 with Advanced DMEM/F‐12 after chilling the plate at −20°C for 30 min and incubated for 16 h at 4°C. Before seeding, coating solution was removed, and the plate was warmed at 37°C for 30 min. After 5 days of culture, spheroids were digested into single cells and seeded at approx. 0.3 × 10^6^ cells per filter in 400 μl pre‐warmed (37°C) spheroid culture medium supplemented with ROCK1 inhibitor Y‐27632 for the first 2 days of culture. Trans‐epithelial electrical resistance (TEER) was measured with an ohmmeter (Millicell® ERS‐2 Volt‐Ohm Meter; Merck Millipore). Organoids were cultured until a stable TEER was reached, differentiation was induced as described above and BTP2 (1 μM), TNF‐α (10 ng/ml), and IFNγ (10 ng/ml) were added as indicated.

#### *In vitro* activation and differentiation of murine T cells with genetic ablation of SOCE signaling components

Naïve T cells were isolated from spleens and LNs of *Stim1* ^*fl/fl*^ *Cd4Cre*, *Stim2* ^*fl/fl*^ *Cd4Cre*, *Stim1* ^*fl/fl*^ *Stim 2* ^*fl/fl*^ *Cd4 Cre*, or *Orai1* ^*fl/fl*^ *Cd4Cre* mice and stimulated with 1 μg/ml plate‐bound anti‐CD3 (clone 2C11 obtained from BioXCell) and 1 μg/ml anti‐CD28 (clone 37.51,obtained from BioXCell) for 3 days in the presence of the following cytokine cocktails for differentiation into T helper (Th) cells. Th1: 10 ng/ml IL‐12 (PeproTech) and 2 μg/ml anti‐IL‐4 (eBioscience), Th17: 20 ng/ml IL‐6 (PeproTech), 0.5 ng/ml human TGF‐β1 (PeproTech), 2 μg/ml anti‐IL‐4 and 2 μg/ml anti‐IFNγ (eBioscience), or iTreg: 2.5 ng/ml TGF‐β1 (PeproTech) in IMDM medium (Cellgro) containing 2 mM L‐glutamine, 50 μM 2‐Mercaptoethanol, 100 U/ml penicillin, 100 μg/ml streptomycin and 10% FCS.

#### *In vitro* activation and differentiation of murine CD4 ^+^ T cells in the presence of increasing dosages of BTP2

CD4^+^ T cells were isolated from spleens and LNs of WT mice using the MagniSort Mouse CD4^+^ T cell enrichment kit (Invitrogen, Cat 8804‐6821‐74) following the manufacturer's instructions. Cells were stimulated with 1 μg/ml anti‐CD3 (BioXCell, clone 2C11) and 1 μg/ml anti‐CD28 (BioXCell, clone 37.51) in a flat plate pre‐coated 20 μg/ml rabbit anti‐hamster IgG (Invitrogen, Cat A18891), cells were then cultured for 3 days in the presence of the following cytokine cocktails, Th1: 10 ng/ml IL‐12 (PeproTech, 210‐12) and 5 μg/ml anti‐mouse IL‐4 (BioXCell, clone 11B11), Th17: 20 ng/ml IL‐6 (R&D), 0.5 ng/ml human TGFβ1 (PeproTech, 100–21), 5 μg/ml anti‐IL‐4 and 5 μg/ml anti‐mouse IFN‐γ (BioXCell, clone XMG1.2), or iTreg: 1.0 ng/ml TGF‐β1 (PeproTech) + 50 U/ml human recombinant IL‐2 (PeproTech, 200‐02), and 5 μg/ml anti‐IL‐4/IFN‐γ in RPIM1640 (ThermoFisher Scientific) containing 2 mM L‐glutamine, 50 μM 2‐Mercaptoethanol, 100 U/ml penicillin, 100 μg/ml streptomycin, and 10% FCS. Varying concentrations of the CRAC channel inhibitor BTP2 (Sigma Aldrich, Y4895) were reconstituted in DMSO and added to cell cultures at the initiation of differentiation. For cytokine detection, cells were stimulated for 4 h with 20 nM phorbol 12‐myristate 13‐acetate (PMA, Calbiochem, 524,400) and 1 μM ionomycin (Invitrogen, I‐24222) in the presence of 5 μM Brefeldin A (eBioscience, 00‐4506‐51) and processed for subsequent flow cytometric analyses as detailed below.

#### RT‐PCR of SOCE signaling components in murine T cells

RNA was isolated from differentiated CD4^+^ T helper cells using the RNeasy Micro Kit (Qiagen). cDNA was synthesized using the iScript II Kit (Bio‐Rad), followed by qRT‐PCR using the Platinum SYBR Green qPCR SuperMix (Invitrogen) and measured with the Opticon 2 thermocycler (Bio‐Rad). The following PCR conditions were used: 95°C for 10 min, followed by 40 cycles (94°C for 45 s, 58°C for 30 s and 72°C for 30 s) of amplification. To quantify the transcript levels, CT values were normalized to Hprt1 and expression was calculated using the 2^−^^ΔΔCT^ method. The following primers for mouse were used: Stim1 forward ATTCGGCAAAACTCTGCTTC and reverse GGCCAGAGTCTCAGCCATAG, Stim2 forward TCGAAGTGGACGAGAGTGATG and reverse TTTCCACTGTTTCCACAAATCC, Orai1 forward AGACTGCCTGATCGGATGGC and reverse TTGTCCCCGAGCCATTTCCT, Orai2 forward GCAGCTACCTGGAACTCGTC and reverse GTTGTGGATGTTGCTCACCG, Orai3 forward CAGTCAGCACTCTCTGCGG and reverse TGGCCACCATGGCGAAG and Hprt1 forward AGCCTAAGATGAGCGCAAGT and reverse TTACTAGGCAGATGGCCACA.

#### T cell transfer colitis

Naïve CD4^+^ T cells were isolated from *Stim1* ^*fl/fl*^ *Cd4Cre*, *Stim2* ^*fl/fl*^ *Cd4 Cre*, *Stim1* ^*fl/fl*^ *Stim 2* ^*fl/fl*^ *Cd4 Cre*, or *Orai1* ^*fl/fl*^ *Cd4Cre* mice by flow cytometric cell sorting for CD4^+^ CD62L^+^ CD25^−^ CD45RB^hi^ using a SONY SY3200 cell sorter. 5 × 10^5^ naive T cells were injected intra‐peritoneally into recipient *Rag1* ^*−/−*^ mice, which were monitored weekly for body weight loss over a period of 8 weeks. Colon sections of host mice were prepared at 8 weeks after T cell transfer, fixed with 4% paraformaldehyde, embedded in paraffin and stained with hematoxylin and eosin (H&E) using standard protocols.

#### Treatment of mice with CM4620

Naïve CD4^+^CD44^low^CD62L^+^CD25^−^ T cells were enriched by cell sorting and injected i.v. into sex‐matched *Rag1* ^*−/−*^ mice (5x10^5^ cells per mouse). Eighteen days after T cell transfer, mice were either treated with the selective CRAC channel inhibitor CM4620 (kindly provided by CalciMedica, La Jolla, CA) or treated with vehicle control (Waldron *et al*, 2019). The CM4620 compound was reconstituted in a vehicle composed of 0.5% methylcellulose (w/w) with a viscosity of 400 cP (Sigma Aldrich, M0262) and 1% w/w Tween 80 (Company, P1754) in ddH2O. The active form of CM4620 was administered by oral gavage at 20 mg per kg bodyweight (25% loading) plus 75 mg/kg bodyweight of hypromellose acetate succinate (HPMCAS) bead carrier formulated in vehicle every other day until the end point. Mice were sacrificed 7 weeks after T cell transfer. CM4620 concentrations in mouse plasma were determined using a high sensitivity method developed by BioAgilytix, San Diego (formerly MicroConstants). The method uses HPLC with MS/MS detection and is suitable for measuring concentrations ranging from 1 to 1,000 ng/ml using 25.0 μl of mouse plasma extracted using a liquid–liquid approach.

#### Scoring of histology sections

Colon samples of distal and proximal colon from *Rag1 ^−/−^* mice injected with naive CD4^+^ T cells were fixed with 4% of paraformaldehyde and embedded in paraffin. Samples were cut into 5 μm sections and stained with hematoxylin and eosin or alcian blue. Distal colon histology sections were scored for inflammation by a pathologist in a blinded fashion using the following grading system (modified from Ostanin *et al* 2009): 1. Degree of inflammation in lamina propria (score 0–3); 2. Goblet cell loss (score 0–2); 3. Abnormal crypts (score 0–3); 4. Presence of crypt abscesses (score 0–1); 5. Mucosal erosion and ulceration (score 0–1); 6. Submucosal spread to transmural involvement (score 0–3); 7. Neutrophil infiltration evaluated at 40x magnification (score 0–4). The total histopathological score for each sample was calculated as the sum of scores for all seven parameters.

#### Isolation of murine lamina propria leukocytes

Colon tissue of mice was opened longitudinally by a curved scissor and cut into pieces around 0.5 to 1 cm, colon bits were predigested with pre‐digestion buffer (5 mM EDTA and 1 mM DTT in HBSS buffer without calcium) for 15 min. Colon bits were then washed, further minced, and digested with digestion buffer (1 mg/ml collagenase IV and 0.1 mg/ml DNase‐I in HBSS with 5% FBS) in 37C shaker at a speed of 225 rpm for 45 min. Cells were then isolated using a 40/75% discontinuous Percoll gradient, the middle layer was collected, washed, and re‐suspended in RPMI1640 with 2% of fetal bovine serum. Cells were counted using hemocytometer.

#### Flow cytometric analyses of murine T cells

For surface antigen staining, cells isolated from the LP and mLN were prepared in PBS containing 2% of FBS and 2 mM EDTA. Cells were stained with fluorescently labeled antibodies for 20 min on ice in the dark. For intracellular cytokine detection, leukocytes were cultured in RPMI1640 plus 10% FBS and stimulated with 20 nM phorbol 12‐myristate 13‐acetate (PMA) (PMA, Calbiochem, 524,400) and 0.5 μM ionomycin (Invitrogen, I‐24222) in the presence of 5 μM brefeldin A (eBioscience, 00‐4506‐51) for 4 h. Cells were washed in PBS 2% containing FBS and incubated with anti‐CD16/CD32 antibodies (2.4G2, BioXcell) for 15 min. Surface marker staining was performed by incubating cells with fluorescently labeled antibodies for 20 min on ice in the dark. For intracellular cytokine staining, cells were fixed with IC Fixation Buffer (eBioscience, Cat: 00‐8222‐49) for 30 min and permeabilizated using Permeabilization Buffer (eBioscience, Cat: 00‐8333‐56). Cells samples were analyzed on a BD LSR Fortessa cell analyzer (BD Biosciences) and data were analyzed using FlowJo software version 10.6.2. (BD). A list of all fluorescent conjugated antibodies can be found in Appendix Table S7.

#### Cytometric bead array

For measuring cytokine production by T cells by cytometric bead array (CBA), colon lamina propria derived total lymphocytes were stimulated with 0.5 μg/ml anti‐CD3 (clone 145‐2C11, BioXcell) for 24 h. Cell supernatants were collected and cytokine levels were measured using the mouse Th1/Th2/Th17 CBA Kit (BD Biosciences, 560485). Cytokine levels in the serum of mice were measured using the same CBA kit following the manufacturer's instructions. Positive (PE bright capture beads) and negative (capture beads only) controls were analyzed accordingly.

#### Cells

HEK293 cells were purchased from Invitrogen and performance tested for viability and screened for viruses, mycoplasma, and sterility by the vendor. Cells were maintained in suspension at 37°C with 5% CO_2_ in CD293 medium (Thermo Fisher Scientific 10‐090‐CV) supplemented with 4 mM GlutaMAX (Thermo Fisher Scientific 35050‐061). For electrophysiology, cells were plated onto poly‐L‐lysine coated coverslips one day before transfection and grown in medium containing DMEM/F12 (Corning:10‐090‐CV), 10% fetal bovine serum (Corning 35‐011‐CV), 2 mM glutamine, 50 U/ml penicillin and 50 μg/ml streptomycin.

#### Transfections

HEK293 cells were transfected with Lipofectamine 2000 (Thermo Fisher Scientific), with WT Orai1‐YFP (100 ng) and mCherry STIM1 (500 ng) in 12‐mm coverslip. mCherry‐STIM1 was a kind gift of Dr. R. Lewis (Stanford University, USA). Cells were used for electrophysiology 24–48 h after transfection.

#### Electrophysiology

The standard extracellular Ringer's solution used for electrophysiological experiments contained 130 mM NaCl, 4.5 mM KCl, 20 mM CaCl_2_, 10 mM tetraethylammonium chloride (TEA‐Cl), 10 mM D‐glucose, and 5 mM HEPES (pH 7.4 with NaOH). The internal solution contained: 135 mM Cs aspartate, 8 mM MgCl_2_, 8 mM Cs‐BAPTA, and 10 mM HEPES (pH 7.2 with CsOH). Currents were recorded in the standard whole‐cell configuration at room temperature on an Axopatch 200B amplifier (Molecular Devices) interfaced to an ITC‐18 input/output board (Instrutech). Routines developed by R. S. Lewis (Stanford) on the Igor Pro software (Wavemetrics) were employed for stimulation, data acquisition, and analysis. Data are corrected for the liquid junction potential of the pipette solution relative to Ringer's in the bath (−10 mV). The holding potential was +30 mV. The standard voltage stimulus consisted of a 100‐ms step to −100 mV followed by a 100‐ms ramp from −100 to +100 mV applied at 1 s intervals. I_CRAC_ was typically activated by passive depletion of ER Ca^2+^ stores by intracellular dialysis of 8 mM BAPTA. All currents were acquired at 5 kHz and low pass filtered with a 1 kHz Bessel filter built into the amplifier. All data were corrected for leak currents collected in 100–200 μM LaCl_3_. Analysis of current amplitudes was typically performed by measuring the peak currents during the −100 mV pulse. Fractional blockade of current was quantified as: blockade = 1 − (I_CM4620_/I_Ctrl_), where I_C4620_ is the ORAI1 current in the presence of CM4620 (3 μM), and I_Ctrl_ is the ORAI1 current prior to application of the blocker.

### Statistical analyses

The GraphPad Prism 9.0 software, the R Software (3.6.0, Bioconductor 9) or the R package *diffcyt* (used all defaults with analysis_type = “DA,” method_DA = “diffcyt‐DA‐GLMM,” min‐cells = 3) were used to calculate statistics as indicated. All statistical tests and exact *P*‐values are summarized in Appendix Tables S8–S22; *P* values < 0.05 were considered significant.

## Author contributions

**Marilena Letizia:** Conceptualization; data curation; formal analysis; validation; investigation; visualization; methodology; writing – original draft; project administration; writing – review and editing. **Yin‐Hu Wang:** Conceptualization; data curation; formal analysis; validation; investigation; visualization; methodology; writing – review and editing. **Ulrike Kaufmann:** Conceptualization; data curation; formal analysis; validation; investigation; visualization; project administration. **Lorenz Gerbeth:** Data curation; formal analysis; investigation; visualization. **Annegret Sand:** Data curation; formal analysis; investigation. **Max Brunkhorst:** Formal analysis; validation; investigation. **Patrick Weidner:** Formal analysis; visualization; methodology. **Jörn Felix Ziegler:** Formal analysis; investigation. **Chotima Böttcher:** Formal analysis; supervision; methodology. **Stephan Schlickeiser:** Formal analysis; supervision. **Camilla Fernández:** Formal analysis. **Megumi Yamashita:** Formal analysis; investigation; visualization; methodology. **Kenneth Stauderman:** Formal analysis; investigation. **Katherine Sun:** Formal analysis. **Désirée Kunkel:** Conceptualization; methodology. **Murali Prakriya:** Conceptualization; supervision; investigation. **Ashley Sanders:** Formal analysis; supervision; methodology. **Britta Siegmund:** Supervision. **Stefan Feske:** Conceptualization; supervision; funding acquisition; validation; investigation; visualization; writing – original draft; project administration; writing – review and editing. **Carl Weidinger:** Conceptualization; supervision; funding acquisition; investigation; visualization; methodology; writing – original draft; project administration; writing – review and editing.

In addition to the CRediT author contributions listed above, the contributions in detail are:

ML, Y‐HW, UK, LG, AS, MB, JFZ, MY and DK performed experiments. ML, UK, Y‐HW, LG, AS, MB, CF, CB, SS, KS, KatS, DK, PW, MY, MP, AshS, SF and CW designed experiments, analyzed and interpreted the data. BS contributed to clinical sample acquisition and helped with data interpretation. ML, Y‐HW, SF and CW wrote the paper.

## Disclosure and competing interests statement

SF is a scientific co‐founder of CalciMedica. KS is a co‐founder and CSO of CalciMedica. BS has served as consultant for Abbvie, Arena, BMS, Boehringer, Celgene, Falk, Galapagos, Janssen, Lilly, Pfizer, Prometheus and Takeda and received speaker's fees from Abbvie, CED Service GmbH, Falk, Ferring, Janssen, Novartis, Pfizer, Takeda [payments were made to the institution]. None of the other authors declare competing interests.

**The paper explained**

**Problem**

The incidence of inflammatory bowel disease (IBD) is on the rise worldwide, with a significant proportion of patients not adequately responding to the currently available immune suppressive therapies. This constitutes a major clinical challenge in need of new treatment modalities to improve patient care. Moreover, the immune responses and signaling cascades controlling immune cell function in patients with therapy‐refractory IBD remain incompletely understood.

**Results**

Using mass and flow cytometry, we observed that the pharmacologic inhibition of store‐operated calcium entry (SOCE) impairs the production of inflammatory cytokines in human colonic T cells, ILCs, B cells, and myeloid cells obtained from patients with Crohn's disease (CD) and ulcerative colitis (UC) in a dose‐dependent manner. Moreover, conditional deletion of the SOCE genes *Stim1*, *Stim2*, and *Orai1* in T cells as well as systemic pharmacologic inhibition of SOCE attenuates IBD severity in a murine T cell transfer model of colitis. By contrast, SOCE inhibition does not impair epithelial barrier function.

**Impact**

Our data suggest that pharmacologic inhibition of SOCE may be a new therapeutic strategy for treating IBD because SOCE inhibition suppresses the function of human and mouse intestinal immune cells without impairing the function of intestinal epithelial cells.

## Supporting information



Appendix

Expanded View Figures PDF

Source Data for Expanded View and Appendix

Source Data for Figure 1

Source Data for Figure 2

Source Data for Figure 3

Source Data for Figure 4

Source Data for Figure 5

Source Data for Figure 6

Source Data for Figure 7

## Data Availability

This study does not contain data that requires deposition in a public database.
