# Supplementary material for: Store‐operated calcium entry controls innate and adaptive immune cell function in inflammatory bowel disease
Source: EMBO Mol Med. 2022 Aug 2;14(9):e15687. doi: 10.15252/emmm.202215687 (PMC9449601; doi:10.15252/emmm.202215687)
Supplement: Supplementary file 1 — Appendix [file EMMM-14-e15687-s009.pdf]

Appendix

Table of content

Appendix Figure S1-S11

Appendix Table S1-S22

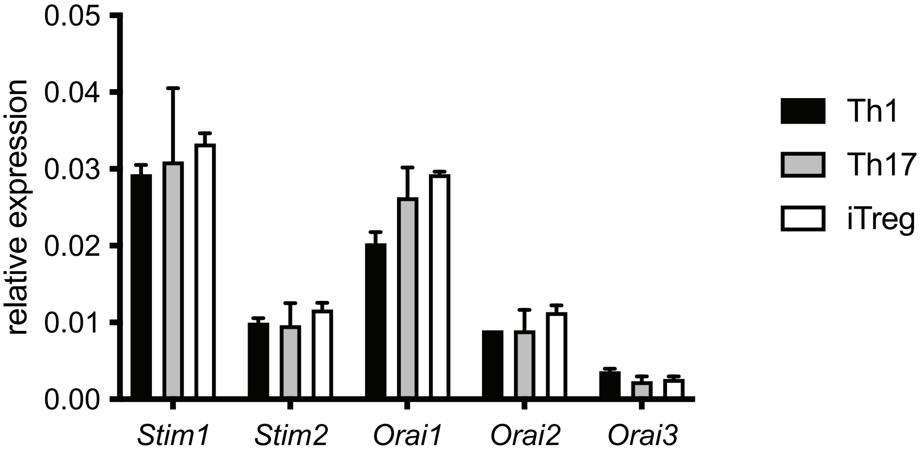

Appendix Figure S1. Expression levels of *Stim1*, *Stim2, Orai1*, *Orai2* and *Orai3* mRNA in different T helper cell subsets. Naïve $CD4^+$ T cells were isolated from spleen and LNs of WT mice and differentiated into Th1, Th17 and iTreg cells for 3 days. mRNA was isolated and analyzed by quantitative real-time PCR. Bar graphs show mean ± SEM from three mice. Statistical analysis by two-way ANOVA. No statistically significant differences were detected.

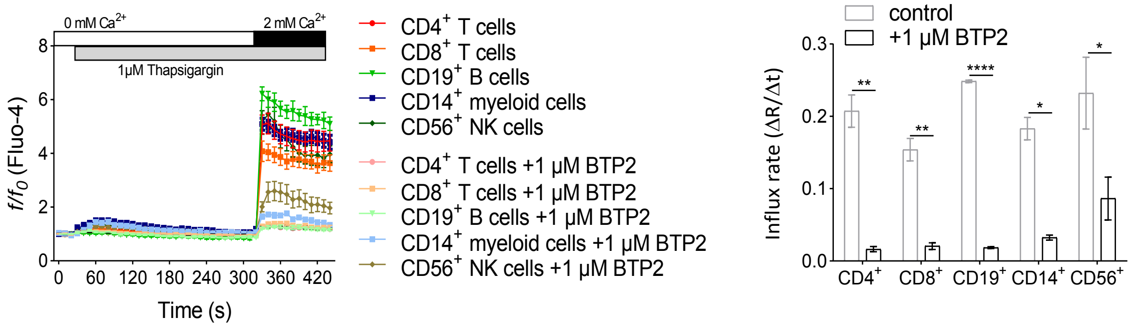

Appendix Figure S2A. Ca^{2+} influx analyses were performed by flow cytometry to assess Ca^{2+} influx in PBMCs isolated from healthy donors (n = 3, in duplicate), with or without 1 µM BTP2 treatment. Statistic was calculated with a paired t test corrected by using the two-stage linear step-up procedure of Benjamini, Krieger and Yekutieli, with Q = 1%. *p-value < 0.05, **p-value < 0.01, ***p-value < 0.001, ****p-value < 0.0001. Bars denote the mean values of influx rate and error bars represent the standard error of mean (SEM).

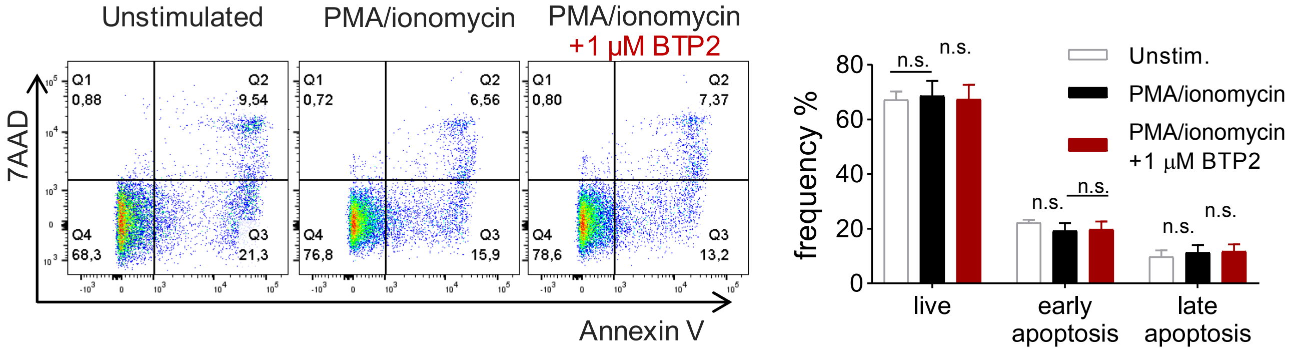

Appendix Figure S2B. LPMCs were isolated from 3 IBD patients and cellular viability in the presence or absence of PMA/Ionomycin (4 h *in vitro*) or additional BTP2 was assessed by quantifying frequencies of early apoptotic (Annexin V^+ $7AAD^-$) and late apoptotic cells (Annexin V^+ $7AAD^+$). Statistics were calculated using a paired Wilcoxon matched-pairs signed rank test, *p < 0.05. Error bars represent the standard error of mean (SEM).

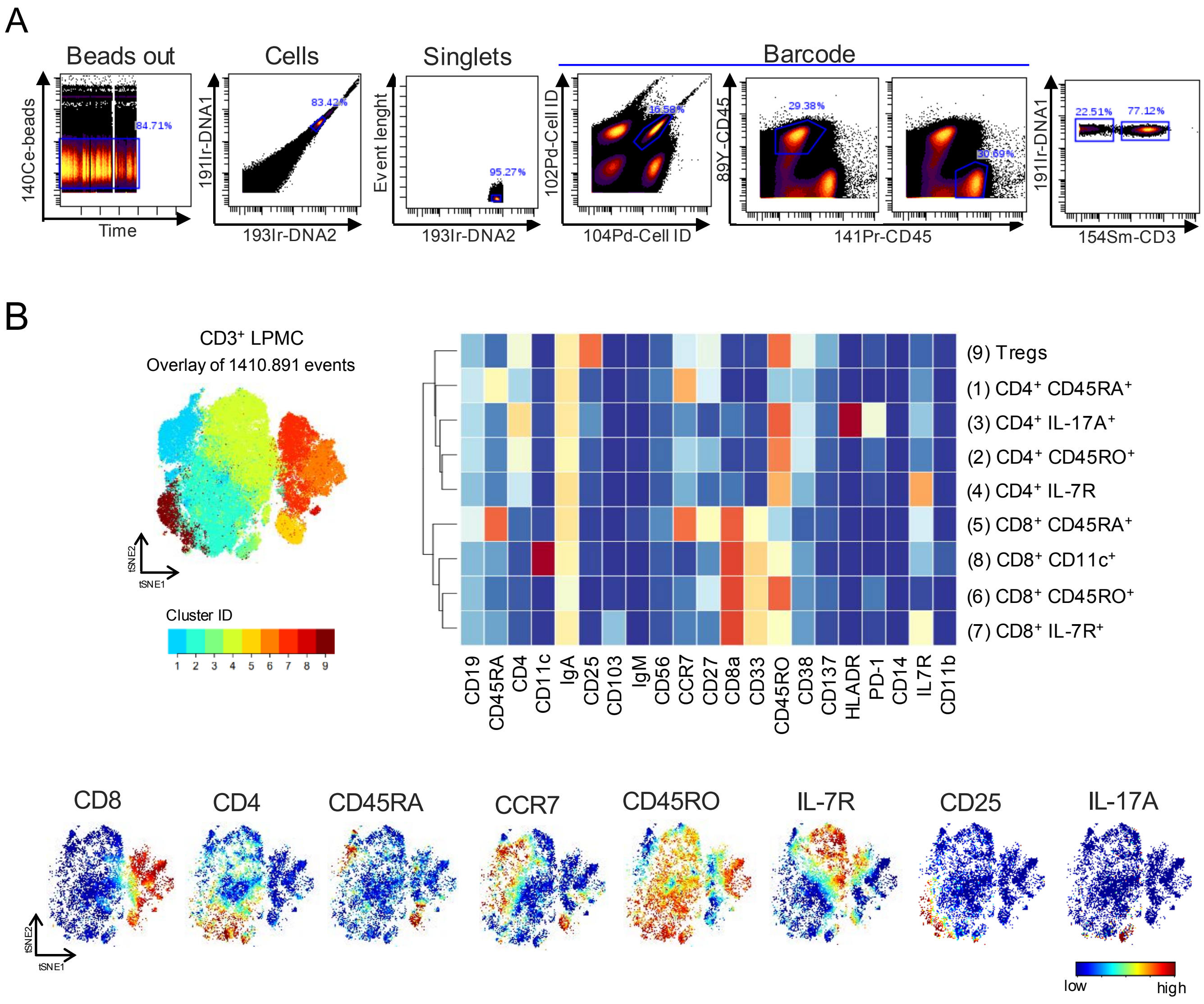

Appendix Figure S3. (**A**) Gating strategy preceding analyses using the t-distributed stochastic linear embedding (t-SNE) algorithm and *FlowSOM/ConsensusClusterPlus* self-organizing map. Cells were cleaned from calibration beads and doublets and de-barcoded according to the Cell-ID 20-plex Pd Barcoding Kit and 89Y-CD45 staining. (**B**) FlowSOM plot of merged FCS files from samples treated with PMA/ionomycin ± 1μM BTP2 (CD: n =5). Colors indicate 9 defined clusters among $CD45^{+}CD3^{+}$ LPMCs. Heatmap clusters show the expression levels of the 21 markers used for cluster analysis. viSNE plots display representative LPMCs isolated from one CD patient colored by marker expression levels (blue: low, red: high).

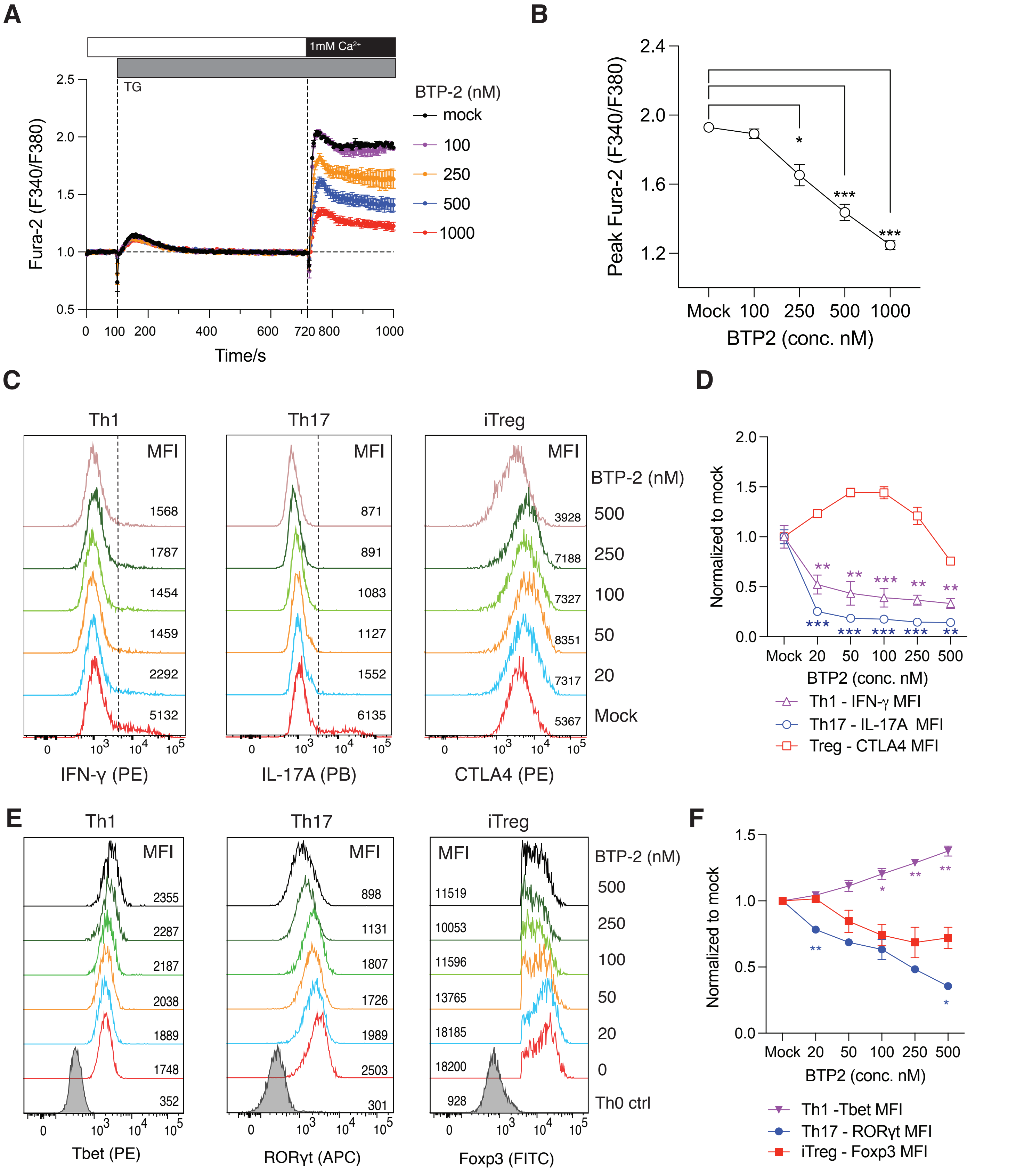

Appendix Figure S4. Effects of CRAC channel inhibitor BTP-2 on the function of Th1, Th17 and iTreg cells. (**A**) Analysis of SOCE in CD4$^+$ T cells cultured in vitro for 3 days under Th0 condition; cells were then treated acutely with BTP-2 for 30 min before calcium measurement. SOCE (peak F340/380) was quantified in (**B**); shown are means±SEM of cells from two mice measured in triplicates. (**C**) Analysis of IFNγ, IL-17A and CTLA-4 expression in Th1, Th17 and iTreg cells with CRAC inhibitor BTP-2 treatment. Naïve T cells were cultured under Th1 and Th17 condition for 3 days in the presence of gradient BTP-2, cells were restimulated with PMA/Ionomycin for 4 hours to detect cytokine production by flow cytometry. CLTA4 in iTreg cells was detected by intracellular staining without PMA/Ionomycin stimulation. (**D**) Quantification of normalized MFI of IFNγ, IL-17A and CTLA-4 by Th1, Th17, iTreg respectively. (**E+F**) Quantification of normalized MFI of Tbet, RORγt and Foxp3 levels in Th1, Th17 and iTreg in the presence of gradient BTP-2 treatment. Statistical analysis by unpaired student's t-test was performed either between Th1 and iTreg or Th17 and iTreg: ***p<0.001 **p<0.01 *p<0.05. Statistical analysis by unpaired student's t-test: ***p<0.001 **p<0.01 *p<0.05. Data in C-F are the mean ± SEM of 3 mice.

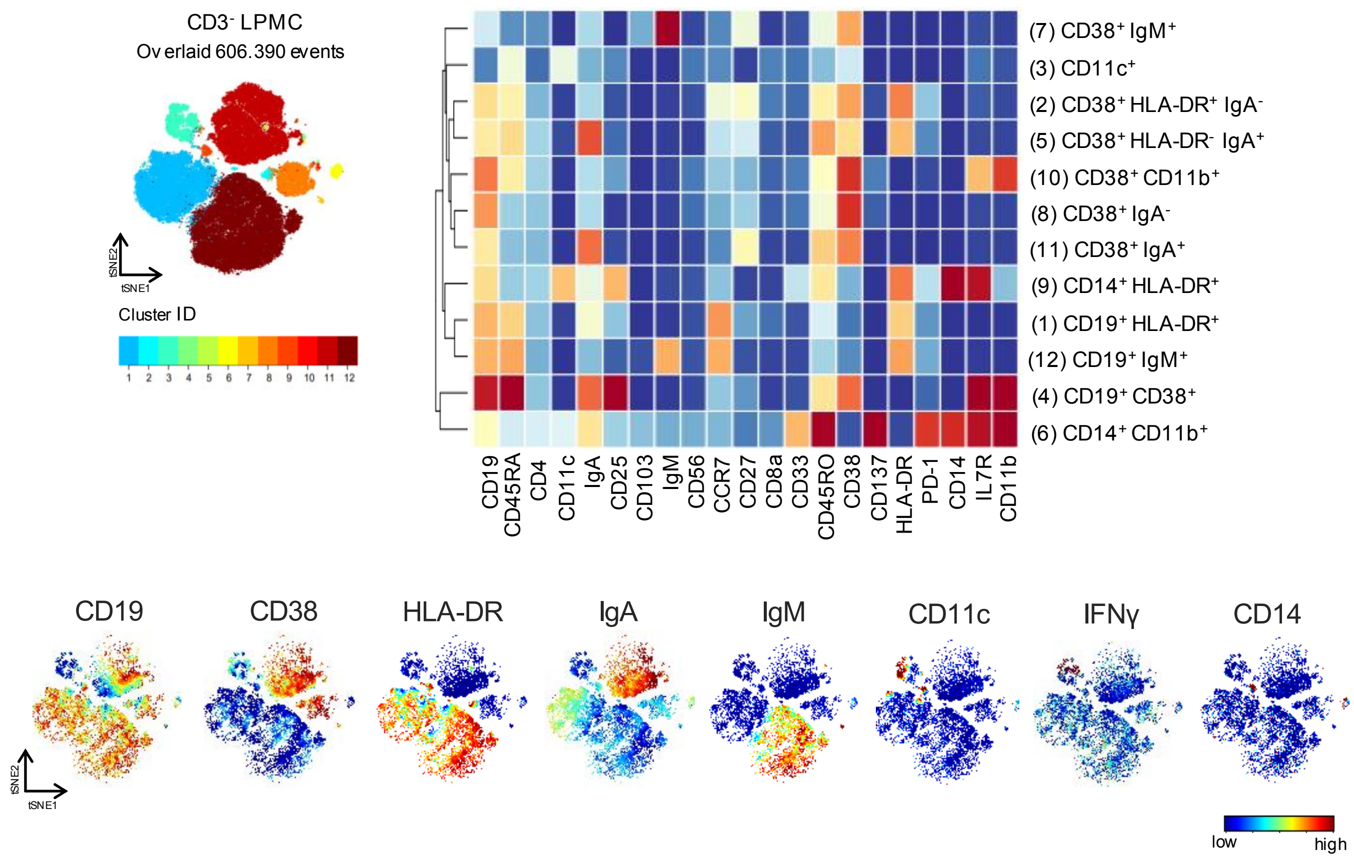

Appendix Figure S5. FlowSOM plot of merged FCS files from samples treated with PMA/ ionomycin ± 1µM BTP2 (CD: n =5). Colors indicate 12 defined clusters among CD45⁺CD3⁻ LPMCs. Heatmap clusters show the expression levels of the 21 markers used for cluster analysis. viSNE plots represent LPMCs isolated from one CD patient colored by marker expression levels (blue: low, red: high).

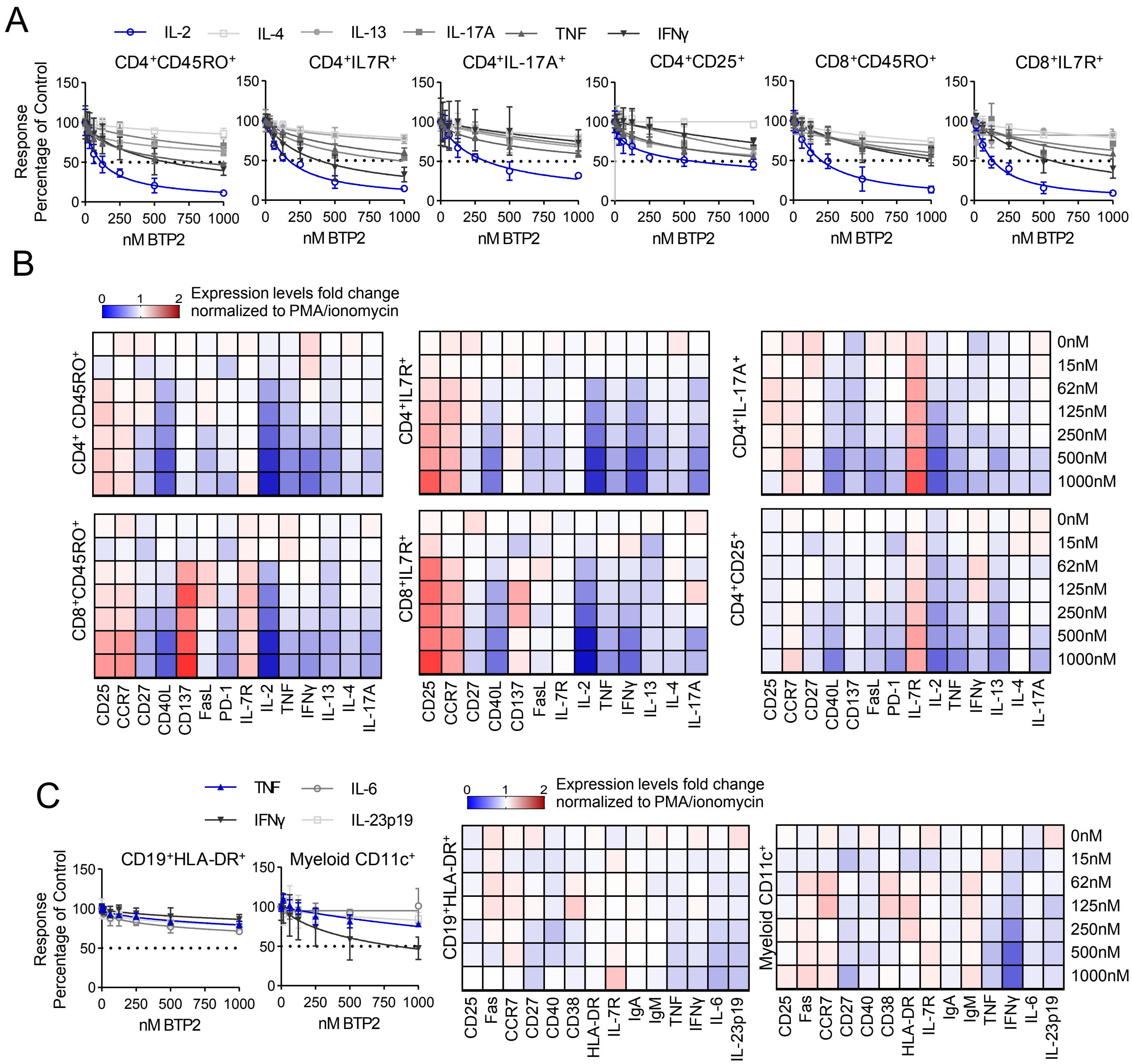

Appendix Figure S6. Dose-dependent suppression of SOCE inhibits the pro-inflammatory cytokine production by lamina propria T cells of UC patients (UC: n=3). (**A**) Dose-response curves reflecting the normalized production of cytokines in response to treatment with increasing dosages (15-1000nM) of BTP2 in CD45^{+}CD3^{+} T cells after 4 h of *ex vivo* stimulation with PMA/ionomycin (UC: n=3). The dose response was normalized to control samples treated with PMA/ionomycin. (**B**) Heatmaps representing the median fold change of cytokines and functional markers expression in CD45^{+}CD3^{+} LPMCs (UC: n=3) activated 4 h *ex vivo* with PMA/ionomycin ± 15-1000nM BTP2, normalized to samples treated with PMA/ionomycin. (**C**) On the left, dose-response curve graphs reflecting the normalized production of cytokines in response to treatment with increasing dosages (15-1000nM) of BTP2 in CD45^{+}CD3^{-}cells after 4 h of *ex vivo* stimulation with PMA/ionomycin (UC: n=3). The dose response was normalized to control samples treated with PMA/ionomycin. Heatmaps representing the median fold change of cytokines and functional markers expression in CD45^{+}CD3^{-} LPMCs (UC: n=3) after 4 h of *ex vivo* stimulaton with PMA/ionomycin ± 15-1000nM BTP2, normalized to samples treated with PMA/ionomycin.

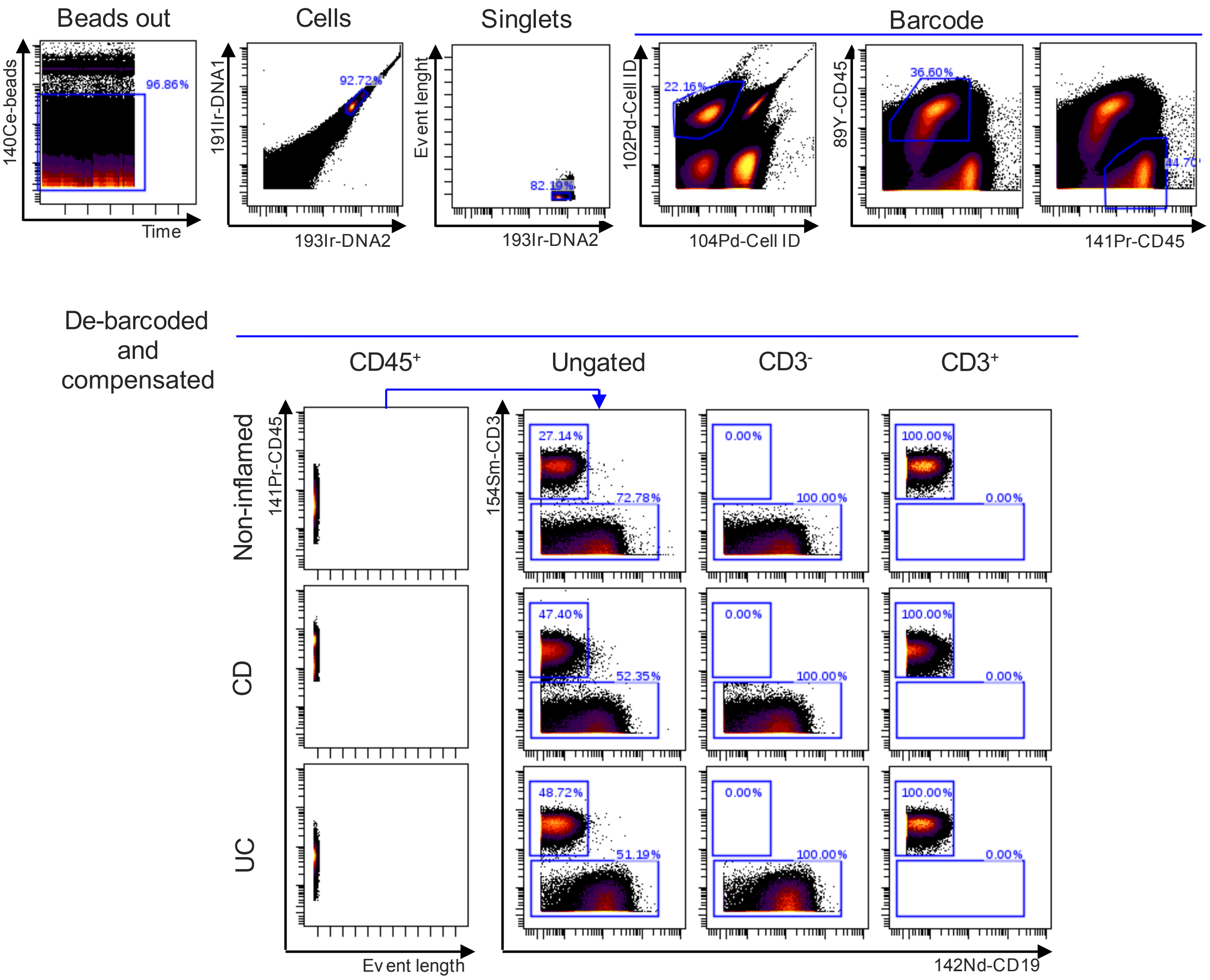

Appendix Figure S7. Gating strategy preceding analyses using the t-distributed stochastic linear embedding (t-SNE) algorithm and *FlowSOM/ConsensusClusterPlus* self-organizing map. After exclusion of beads and doublets, de-barcoding and compensation, cells were gated on $CD45^{+}$ or $CD45^{+}CD3^{+}$ and $CD45^{+}CD3^{-}$ for further deep immune-characterization.

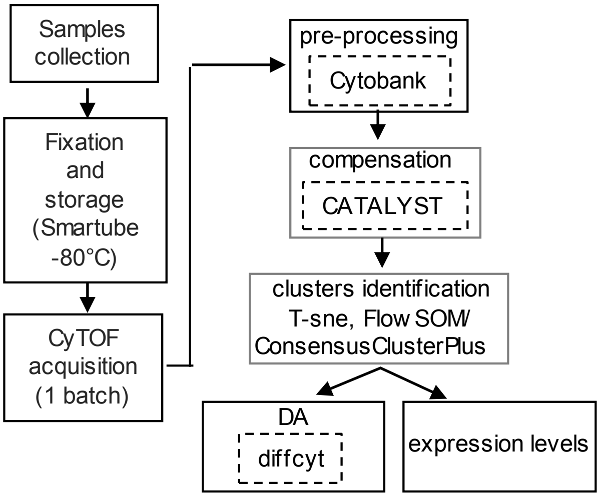

Appendix Figure S8. Experimental design and analysis workflow applied to CyTOF data.

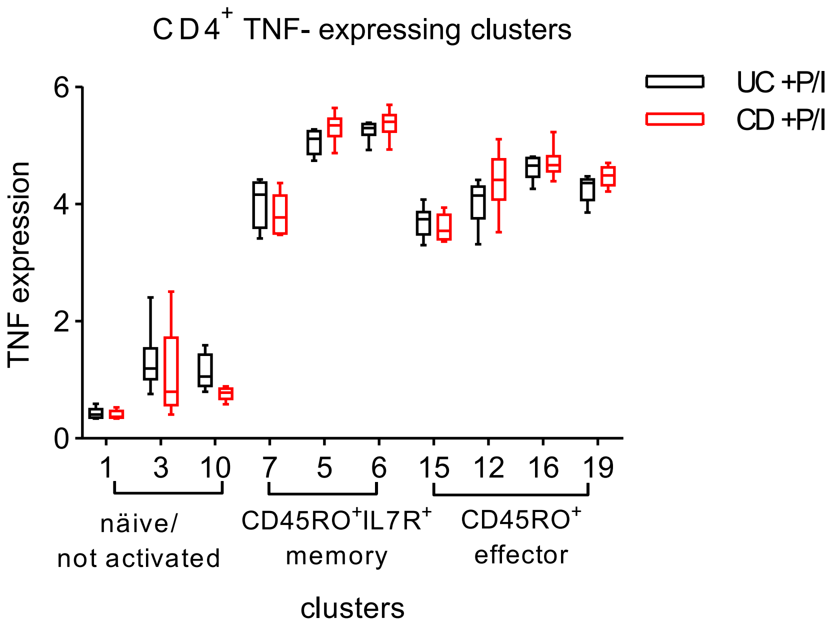

Appendix Figure S9. TNFα expression among $CD4^+$ näive/not activated (clusters 1,3 and 10), memory (clusters 7,5 and 6) or effector T cells (clusters 15,12,16 and 19) identified by the *FlowSOM/ConsensusClusterPlus* analysis performed on $CD3^+CD45^+$ LPMCs isolated from UC and CD patients and activated for 4 h *in vitro* with PMA/ionomycin (**Figure 4**). Boxplots showing mean expression levels (arbitrary unit) of TNF in each cell cluster. Boxes extend from the 25th to 75th percentiles. Whisker plots show the min (smallest) and max (largest) values. The line in the box denotes the median.

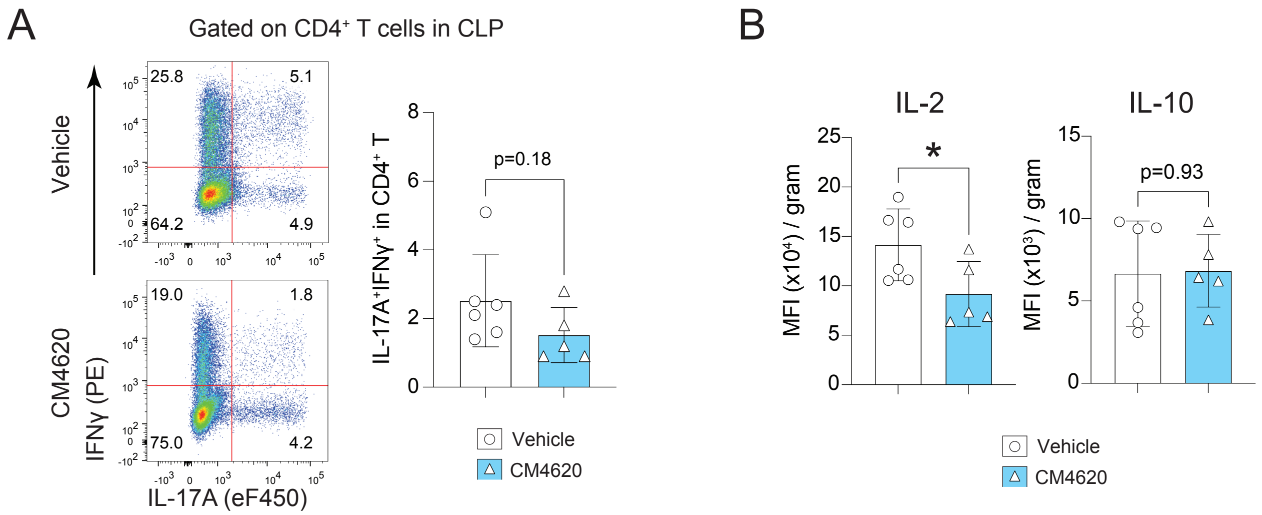

Appendix Figure S10. (**A**) Flow cytometric analysis of IFNγ and IL-17 producing CD4⁺ T cells obtained from the colon lamina propria of colitic mice after treatment with vehicle or the SOCE inhibitor CM4620. Cells were restimulated for 4 hours with PMA/Ionomycin. (**B**) Cytometric bead analyses (CBA) of IL-2 and IL-10 in the supernatant of colon lamina propria derived lymphocytes stimulated with 0.5ug/mL anti-CD3 for 24 hours. Each dot represents one mouse. Statistical analysis by unpaired student's t-test: ***$p<0.001$ **$p<0.01$ *$p<0.05$.

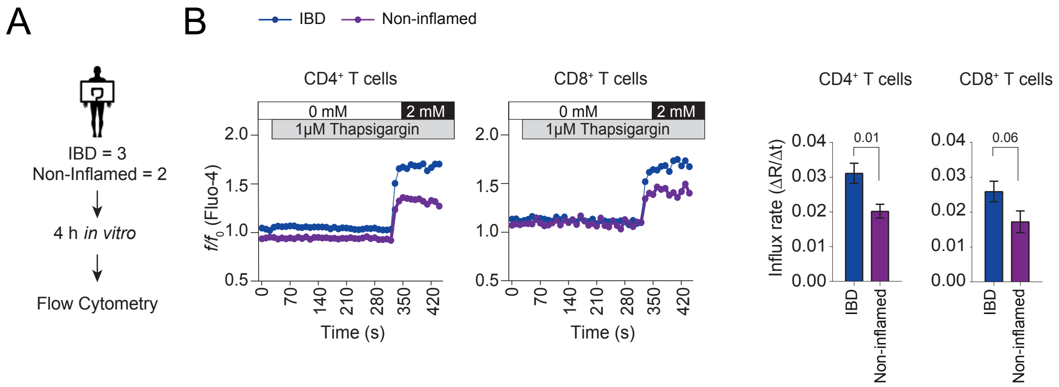

Appendix Figure S11. Enhanced SOCE in IBD and increased expression of SOCE signature modules in IBD compared to Non-inflamed controls. (**A**) Experimental setup for Ca^{2+} influx assays on LPMCs (IBD: n = 3, Non-inflamed n= 2) by Flow cytometry. All samples have been acquired in quadruplicate. (**B**) Dot plots on the right show Ca^{2+} influx rates in human lamina propria $CD4^{+}$ and $CD8^{+}$ T cells kept 4 h *in-vitro* and stimulated with the sarco-endoplasmic reticulum Ca ATPase (SERCA) inhibitor thapsigargin (TG) followed by the addition of 2mM Ca ringer solution. Bar plots on the right indicate the mean Ca^{2+} Influx rate after the addition of 2mM Ca ringer solution. Error bars indicate the standard error mean (SEM). Statistics were calculated using an unpaired t test, exact p-values are shown.

Total patients	30
Sex	
Males	14
Male %	46.67%
Females	16
Female %	53.33%
Age	
Mean	47.1
Median	44
Range min	19
Range max	91
Disease/control	
Crohn's disease	11
percentage	36.67%
Ulcerative colitis	13
percentage	43.33%
Control	6
percentage	20.00%
Patients on anti-inflammatory medications at inclusion	12
percentage	40.00%
Mean leucocyte count per nl at inclusion	8.18
Range min	4.28
Range max	17.57

Appendix Table S1: Patients‘ characteristics at inclusion.

Metal	target	Isotype	Clone	Company	dilution
89Y	CD45	IgG1	HI30	Fluidigm	1:100
141Pr	CD45	IgG1	HI30	Fluidigm	1:100
142Nd	CD19	IgG1	HIB19	Fluidigm	1:100
143Nd	CD45RA	Mouse IgG2b	HI100	Fluidigm	1:100
144Nd	IL-4	Rat IgG1	MP4-25D2	Fluidigm	1:100
145Nd	CD4	Mouse IgG1	RPA-T4	Fluidigm	1:50
146Nd	TNF	IgG1	Mab11	Fluidigm	1:100
147Sm	CD11c	Mouse IgG1	Bu15	Fluidigm	1:200
148Nd	IgA	Goat	Polyclonal	Fluidigm	1:100
149Sm	CD25	Mouse IgG1	2A3	Fluidigm	1:100
150 Nd	CD86	IgG2b	IT2.2	Fluidigm	1:100
151Eu	CD103	Mouse IgG1	Ber-ACT8	Fluidigm	1:100
152Sm	Fas	Mouse IgG1	DX2	Fluidigm	1:200
153Eu	IgM	Mouse IgG1	MHM- 88	Biolegend	1:200
154Sm	CD3	Mouse IgG1	UCTH1	Fluidigm	1:100
155Gd	CD56	IgG1	B159	Fluidigm	1:100
156Gd	IL- 6	IgG1	MQ2-13AS	Fluidigm	1:100
158Gd	IFNγ	Mouse IgG1	B27	Fluidigm	1:400
159Tb	CCR7	IgG2a	G043H7	Fluidigm	1:200
160Gd	CD27	Mouse IgG1, k	2EA	Biolegend	1:200
161Dy	IL-23p19	IgG2b	23dcdp	Fluidigm	1:100
162Dy	CD8	Mouse IgG1	RPA-T8	Fluidigm	1:100
163Dy	CD33	Mouse IgG1	WM53	Fluidigm	1:100
164Dy	CD45RO	Mouse IgG2a	UCHL1	Fluidigm	1:100
165Ho	CD40	Mouse IgG1	5C3	Fluidigm	1:100
166Er	IL- 2	Rat IgG2a	MQ117H12	Fluidigm	1:100
167Er	CD38	Mouse IgG1	HIT2	Fluidigm	1:200
168Er	CD40L	Mouse IgG1	24-31	Fluidigm	1:100
169Tm	IL-13	Rat IgG1	JES105A2	Fluidigm	1:100
170Er	CD137	Mouse IgG1, k	4B4-1	Biolegend	1:100
171Yb	FasL	Mouse IgG1, k	NOK-1	Biolegend	1:100
172Yb	IL-17A	Mouse IgG1	BL168	Fluidigm	1:100
173Yb	HLA-DR	Mouse IgG2a	L243	Fluidigm	1:200
174Yb	PD-1	Mouse IgG1	EH12.2H7	Fluidigm	1:200
175Lu	CD14	Mouse IgG2a	M5E2	Fluidigm	1:50
176Yb	IL-7R	Mouse IgG1	A019D5	Fluidigm	1:50
209Bi	CD11b	IgG1	ICRF44	Fluidigm	1:100

Appendix Table S2. Antibodies used for Mass Cytometry staining**.**

Metal	target
142Nd	CD19
143Nd	CD45RA
145Nd	CD4
147Sm	CD11c
148Nd	IgA
149Sm	CD25
151Eu	CD103
153Eu	IgM
155Gd	CD56
159Tb	CCR7
160Gd	CD27
162Dy	CD8
163Dy	CD33
164Dy	CD45RO
167Er	CD38
170Er	CD137
173Yb	HLA-DR
175Lu	CD14
176Yb	IL- 7R
209Bi	CD11b

Appendix Table S3. Markers used for viSNE clustering of $CD45^{+}CD3^{+}$ and $CD45^{+}CD3^{-}$ LPMCs described in **Figure 2D-G.**

Metal	target
142Nd	CD19
143Nd	CD45RA
145Nd	CD4
147Sm	CD11c
148Nd	IgA
149Sm	CD25
151Eu	CD103
153Eu	IgM
154Sm	CD3
155Gd	CD56
159Tb	CCR7
160Gd	CD27
162Dy	CD8
163Dy	CD33
164Dy	CD45RO
167Er	CD38
170Er	CD137
173Yb	HLA- DR
175Lu	CD14
176Yb	IL-7R
209Bi	CD11b

Appendix Table S4. Markers used for viSNE clustering of $CD45^{+}$ LPMCs described in **Figure 3.**

Metal	target
143Nd	CD45RA
144Nd	IL-4
145Nd	CD4
146Nd	TNF
149Sm	CD25
151Eu	CD103
152Sm	Fas
156Gd	IL-6
158Gd	IFNg
159Tb	CCR7
160Gd	CD27
161Dy	IL- 23p19
162Dy	CD8
164Dy	CD45RO
165Ho	CD40
166Er	IL- 2
167Er	CD38
168Er	CD40L
169Tm	IL-13
170Er	CD137
171Yb	FasL
172Yb	IL-17A
174Yb	PD-1
176Yb	IL-7R

Appendix Table S5. Markers used for viSNE clustering of $CD45^{+}CD3^{+}$ LPMCs described in **Figure 4.**

Metal	target
142Nd	CD19
143Nd	CD45RA
144Nd	IL-4
145Nd	CD4
146Nd	TNF
147Sm	CD11c
148Nd	IgA
149Sm	CD25
150 Nd	CD86
151Eu	CD103
152Sm	Fas
153Eu	IgM
155Gd	CD56
156Gd	IL-6
158Gd	IFNγ
159Tb	CCR7
160Gd	CD27
161Dy	IL-23p19
162Dy	CD8
163Dy	CD33
164Dy	CD45RO
165Ho	CD40
166Er	IL-2
167Er	CD38
168Er	CD40L
169Tm	IL-13
170Er	CD137
171Yb	FasL
172Yb	IL-17A
173Yb	HLA-DR
174Yb	PD-1
175Lu	CD14
176Yb	IL-7R
209Bi	CD11b

Appendix Table S6. Markers used for viSNE clustering of $CD45^{+}CD3^{-}$ LPMCs described in **Figure 5.**

Antigen	Manufacturer	Clone#	Conjugation	Conc. mg/mL	Dilution
CD11b	eBioscience	M1/70	PE-Cy7	0.2	1:500
CD4	Biolegend	GK1.5	APC-Cy7	0.2	1:500
CD44	eBioscience	IM7	FITC	0.5	1:500
CD62L	Biolegend	Mel-14	PerCP-Cy5.5	0.2	1:200
IFN-γ	Biolegend	XMG1.2	PE	0.2	1:100
IL-17A	eBioscience	eBio17B7	eFluor 450	0.2	1:100
IL-2	eBioscience	JES6-5H4	FITC	0.5	1:200
TNF	eBioscience	MP6-XT22	APC	0.2	1:100
Ly6G	Biolegend	1A8	Alexa-Fluor647	0.5	1:500
CD25	Biolegend	PC61	PE	0.2	1:200
Foxp3	eBioscience	FJK-16s	PE	0.2	1:100
RORγt	eBioscience	B2D	APC	0.2	1:100
CTLA4	Biolegend	UC10-4B9	PE	0.2	1:200

Appendix Table S7. Antibodies used for Flow Cytometry stainings of murineT cell experiments

Appendix Table S8. (**A-D**) Statistical summary and p-values of Figure 1.

A. Statistical summary and p-values Figure 1A

One-way ANOVA test

Dunnett's multiple comparisons test	Summary	Adjusted p-value
22 WT Th1 vs. 6 S2 Th1	*	0.0445
22 WT Th1 vs. 10 O1 Th1	****	<0.0001
22 WT Th1 vs. 14 S1 Th1	****	<0.0001

Dunnett's multiple comparisons test	Summary	Adjusted p-value
23 WT Th17 vs. 7 S2 Th17	ns	0.2498
23 WT Th17 vs. 11 O1 Th17	****	<0.0001
23 WT Th17 vs. 15 S1 Th17	****	<0.0001

Dunnett's multiple comparisons test	Summary	Adjusted p-value
24 WT iTreg vs. 8 S2 iTreg	ns	0.3695
24 WT iTreg vs. 12 O1 iTreg	***	0.0008
24 WT iTreg vs. 16 S1 iTreg	****	<0.0001

B. Statistical summary and p-values Figure 1B, (body weight of colitis mice)

Two-way ANOVA test

Uncorrected Fisher's LSD	Summary	Individual p-value
Wildtype vs. *Stim1$^{fl/fl}$ Cd4-Cre*	****	<0.0001
Wildtype vs. *Stim2$^{fl/fl}$ Cd4-Cre*	ns	0.4404
Wildtype vs. *Orai1$^{fl/fl}$ Cd4-Cre*	*	0.0407

C. Statistical summary and p-values Figure 1C, (colitis histology score)

Mann-Whitney test

	p-value
WT vs Orai1-/-	0.1429
WT vs Stim1-/-	0.0238

D. Statistical summary and p-values Figure 1E, (frequencies of IFN-g, IL-17A, TNF and Foxp3 CD4$^+$ T cells in mLNs)

One-way ANOVA test for IFN-g comparisons

Uncorrected Fisher's LSD	Summary	Individual p-value
Wildtype vs. *Stim1$^{fl/fl}$ Cd4-Cre*	***	0.0007
Wildtype vs. *Orai1$^{fl/fl}$ Cd4-Cre*	**	0.0045
Wildtype vs. *Stim2$^{fl/fl}$ Cd4-Cre*	ns	0.1834

One-way ANOVA test for IL-17A comparisons

Uncorrected Fisher's LSD	Summary	Individual p-value
Wildtype vs. *Stim1$^{fl/fl}$ Cd4-Cre*	***	0.0007
Wildtype vs. *Orai1$^{fl/fl}$ Cd4-Cre*	**	0.001
Wildtype vs. *Stim2$^{fl/fl}$ Cd4-Cre*	ns	0.1949

One-way ANOVA test for TNF comparisons

Uncorrected Fisher's LSD	Summary	Individual p-value
Wildtype vs. *Stim1$^{fl/fl}$ Cd4-Cre*	**	0.0031
Wildtype vs. *Orai1$^{fl/fl}$ Cd4-Cre*	**	0.0042
Wildtype vs. *Stim2$^{fl/fl}$ Cd4-Cre*	ns	0.7386

One-way ANOVA test for Foxp3 comparisons

Uncorrected Fisher's LSD	Summary	Individual p-value
Wildtype vs. *Stim1$^{fl/fl}$ Cd4-Cre*	***	0.0002
Wildtype vs. *Stim2$^{fl/fl}$ Cd4-Cre*	*	0.0247
Wildtype vs. *Orai1$^{fl/fl}$ Cd4-Cre*	***	0.0008

Appendix Table S9. (**A-C**) Statistical Summary and p-values of Figure 2.

A. Statistical Summary and p-values Figure 2B, (calcium Influx Rate in LPMCs),
RM one-way ANOVA test, corrected for multiple comparison with Two-stage linear step-up procedure of Benjamini, Krieger and Yekutieli, (Q = 0.05).

CD4 T cells (LPMC)	Individual p-value
0nM vs. 15nM	0.0019
0nM vs. 62nM	0.0002
0nM vs. 125nM	<0.0001
0nM vs. 250nM	<0.0001
0nM vs. 500nM	<0.0001
0nM vs. 1000nM	<0.0001

CD8 T cells (LPMC)	Individual p-value
0nM vs. 15nM	0.1118
0nM vs. 62nM	0.0002
0nM vs. 125nM	<0.0001
0nM vs. 250nM	<0.0001
0nM vs. 500nM	<0.0001
0nM vs. 1000nM	<0.0001

B. Statistical Summary and p-values Figure 2E, (expression levels fold change normalized to PMA/ionomycin)
One-tailed Wilcoxon matched-pairs signed rank test comparing expression fold change of P/I + (15-1000) nM BTP2 with P/I + 0 nM BTP2.
Tables indicate individual p-values.

CD4+ CD45RO+	0nM vs. 15nM	0nM vs. 62nM	0nM vs. 125nM	0nM vs. 250nM	0nM vs. 500nM	0nM vs. 1000nM
CD25	0.3125	0.2188	0.0625	0.0313	0.0313	0.0313
CCR7	0.0625	0.2188	0.0625	0.0313	0.0313	0.0313
CD27	0.4063	0.4063	0.5	0.3125	0.3125	0.0313
CD40L	0.2188	0.0625	0.0625	0.0313	0.0313	0.0313
CD137	0.3125	0.2188	0.1563	0.0625	0.938	0.0625
FasL	0.0938	0.3125	0.0625	0.0313	0.0313	0.0313
PD-1	0.1563	0.5	0.3125	0.2188	0.0313	0.4063
IL-7R	0.2188	0.2188	0.2188	0.0938	0.2188	0.3125
IL-2	0.0313	0.0313	0.0313	0.0313	0.0313	0.0313
TNF	0.2188	0.0625	0.0313	0.0313	0.0313	0.0313
IFNγ	0.5	0.2188	0.0313	0.0313	0.0313	0.0313
IL-13	0.0938	0.0938	0.1563	0.0313	0.0313	0.0313
IL-4	0.0938	0.5	0.5	0.3125	0.1563	0.0313
IL-17A	0.4063	0.2188	0.0313	0.0313	0.0313	0.0313

CD4+ IL7R+	0nM vs. 15nM	0nM vs. 62nM	0nM vs. 125nM	0nM vs. 250nM	0nM vs. 500nM	0nM vs. 1000nM
CD25	0.5	0.0313	0.0313	0.0313	0.0313	0.0313
CCR7	0.1563	0.0313	0.1563	0.0313	0.0313	0.0313
CD27	0.4063	0.3125	0.2188	0.2188	0.0625	0.3125
CD40L	0.5	0.1563	0.0625	0.0313	0.0313	0.0313
CD137	0.2188	0.0313	0.2188	0.0938	0.0625	0.0625
FasL	0.0938	0.938	0.0313	0.0313	0.0625	0.0625
PD-1	0.2188	0.4063	0.2188	0.0938	0.0313	0.0625
IL-7R	0.3125	0.1563	0.3125	0.4063	0.3125	0.4063
IL-2	0.0313	0.0313	0.0313	0.0313	0.0313	0.0313
TNF	0.5	0.0625	0.0313	0.0313	0.0313	0.0313
IFNγ	0.5	0.0313	0.0313	0.0313	0.0313	0.0313
IL-13	0.3125	0.5	0.0625	0.0625	0.0625	0.0625
IL-4	0.2188	0.5	0.0313	0.0625	0.0625	0.0313
IL-17A	0.2188	0.0313	0.0313	0.0313	0.0313	0.0313

CD4+ IL17A+	0nM vs. 15nM	0nM vs. 62nM	0nM vs. 125nM	0nM vs. 250nM	0nM vs. 500nM	0nM vs. 1000nM
CD25	0.4063	0.3125	0.4063	0.1563	0.0313	0.0625
CCR7	0.0938	0.0625	0.2188	0.0938	0.0625	0.0625
CD27	0.4063	0.3125	0.4063	0.3125	0.3125	0.5
CD40L	0.3125	0.2188	0.0625	0.0313	0.0313	0.0313
CD137	0.3125	0.5	0.4063	0.1563	0.4063	0.3125

FasL	0.4063	0.5	0.0625	0.0313	0.0625	0.0313
PD-1	0.5	0.5	0.4063	0.0938	0.0625	0.0313
IL-7R	0.0313	0.2188	0.1563	0.0313	0.0313	0.0313
IL-2	0.1563	0.0938	0.0313	0.0313	0.0313	0.0313
TNF	0.2188	0.0313	0.0938	0.0313	0.0313	0.0313
IFNγ	0.4063	0.2188	0.0313	0.0313	0.0313	0.0313
IL-13	0.0313	0.5	0.0938	0.0938	0.0938	0.0625
IL-4	0.0938	0.4063	0.0938	0.4063	0.0313	0.0625
IL-17A	0.0938	0.3125	0.0625	0.0313	0.0313	0.0313

CD8+ CD45RO+	0nM vs. 15nM	0nM vs. 62nM	0nM vs. 125nM	0nM vs. 250nM	0nM vs. 500nM	0nM vs. 1000nM
CD25	0.4063	0.0313	0.0313	0.0625	0.0313	0.0313
CCR7	0.0625	0.0313	0.0938	0.0313	0.0313	0.0313
CD27	0.5	0.4063	0.2188	0.3125	0.2188	0.0938
CD40L	0.4063	0.4063	0.0938	0.0313	0.0313	0.0313
CD137	0.2188	0.0313	0.0313	0.0313	0.0313	0.0313
FasL	0.3125	0.3125	0.5	0.4063	0.5	0.0938
PD-1	0.2188	0.2188	0.4063	0.3125	0.1563	0.0313
IL-7R	0.0938	0.5	0.5	0.0625	0.0313	0.0313
IL-2	0.1563	0.0313	0.0313	0.0313	0.0313	0.0313
TNF	0.5	0.0313	0.0625	0.0313	0.0313	0.0313
IFNγ	0.0938	0.4063	0.0625	0.0313	0.0313	0.0313
IL-13	0.4063	0.3125	0.1563	0.0625	0.1563	0.0313
IL-4	0.1563	0.4063	0.1563	0.0313	0.0313	0.0313
IL-17A	0.2188	0.0938	0.5	0.1563	0.0313	0.0313

CD8+ IL7R+	0nM vs. 15nM	0nM vs. 62nM	0nM vs. 125nM	0nM vs. 250nM	0nM vs. 500nM	0nM vs. 1000nM
CD25	0.0313	0.0313	0.1563	0.0313	0.0313	0.0313
CCR7	0.0625	0.0625	0.3125	0.0313	0.0313	0.0313
CD27	0.0938	0.0938	0.2188	0.0938	0.0625	0.0313
CD40L	0.4063	0.4063	0.1563	0.0313	0.0313	0.0313
CD137	0.0313	0.0313	0.0313	0.0313	0.0313	0.0313
FasL	0.5	0.5	0.2188	0.5	0.3125	0.1563
PD-1	0.3125	0.3125	0.2188	0.0313	0.0313	0.0313
IL-7R	0.4063	0.4063	0.5	0.0625	0.0313	0.0313
IL-2	0.0313	0.0313	0.0313	0.0313	0.0313	0.0313
TNF	0.0313	0.0313	0.0313	0.0313	0.0313	0.0313
IFNγ	0.0938	0.0938	0.0313	0.0313	0.0313	0.0313
IL-13	0.4063	0.4063	0.5	0.1563	0.5	0.3125
IL-4	0.3125	0.3125	0.1563	0.3125	0.0938	0.0313
IL-17A	0.5	0.5	0.3125	0.0938	0.0313	0.0313

CD4+ CD25+	0nM vs. 15nM	0nM vs. 62nM	0nM vs. 125nM	0nM vs. 250nM	0nM vs. 500nM	0nM vs. 1000nM
CD25	0.5	0.3125	0.5	0.5	0.5	0.3125
CCR7	0.0625	0.0313	0.0625	0.0313	0.0313	0.0313
CD27	0.2188	0.0313	0.2188	0.3125	0.3125	0.4063
CD40L	0.3125	0.0313	0.0313	0.0313	0.0313	0.0313
CD137	0.4063	0.5163	0.3125	0.2188	0.1563	0.2188
FasL	0.3125	0.1563	0.2188	0.1563	0.0625	0.0313
PD-1	0.0938	0.3125	0.2188	0.0625	0.0313	0.0313
IL-7R	0.5	0.2188	0.2188	0.5	0.2188	0.5163
IL-2	0.4063	0.1563	0.0938	0.0313	0.0313	0.0313
TNF	0.0313	0.0313	0.0313	0.0313	0.0313	0.0313
IFNγ	0.0313	0.3125	0.0313	0.0313	0.0313	0.0313
IL-13	0.0923	0.0625	0.1563	0.0938	0.0938	0.0938
IL-4	0.1563	0.4063	0.5	0.5	0.3125	0.3125
IL-17A	0.2188	0.0313	0.2188	0.0313	0.0313	0.0313

C. Statistical Summary and p-values Figure 2G, (expression levels fold change normalized to PMA/ionomycin)

One-tailed Wilcoxon matched-pairs signed rank test comparing expression fold change of P/I + (15-1000) nM BTP2 with P/I + 0 nM BTP2. Tables indicate individual p-values.

CD19+ HLA-DR+	0nM vs. 15nM	0nM vs. 62nM	0nM vs. 125nM	0nM vs. 250nM	0nM vs. 500nM	0nM vs. 1000nM
CD25	0.0625	0.0313	0.2188	0.0313	0.0313	0.0313
Fas	0.2188	0.0625	0.4063	0.0938	0.0938	0.0938
CCR7	0.0313	0.0313	0.0313	0.0313	0.0313	0.0313
CD27	0.4063	0.3125	0.4063	0.3125	0.3125	0.0313
CD40	0.0313	0.0625	0.2188	0.0938	0.0625	0.0313
CD38	0.3125	0.4063	0.4063	0.4063	0.3125	0.2188
HLA-DR	0.4063	0.0625	0.4063	0.1563	0.1563	0.1563
IL-7R	0.3125	0.0625	0.1563	0.4063	0.3125	0.5
IgA	0.1563	0.1563	0.2188	0.2188	0.2188	0.0625
IgM	0.3125	0.2188	0.1563	0.0313	0.3125	0.5
TNF	0.4063	0.0313	0.0625	0.0313	0.0313	0.0313
IFNγ	0.1563	0.4063	0.1563	0.1563	0.1563	0.0313
IL-6	0.0625	0.0313	0.1563	0.0938	0.0938	0.0313
IL-23p19	0.0313	0.1563	0.0938	0.0938	0.0625	0.0625

CD11c+ Myeloid	0nM vs. 15nM	0nM vs. 62nM	0nM vs. 125nM	0nM vs. 250nM	0nM vs. 500nM	0nM vs. 1000nM
CD25	0.2188	0.3125	0.4063	0.0313	0.0313	0.0313
Fas	0.3125	0.0313	0.4063	0.0313	0.0938	0.0625
CCR7	0.0313	0.0313	0.1563	0.0313	0.0313	0.0313
CD27	0.5	0.5	0.0938	0.3125	0.0313	0.2188
CD40	0.4063	0.5	0.1563	0.5	0.3125	0.5
CD38	0.0938	0.0625	0.3125	0.313	0.0313	0.0313
HLA-DR	0.1563	0.3125	0.5	0.3125	0.4063	0.4063
IL-7R	0.4063	0.4063	0.4063	0.3125	0.5	0.4063
IgA	0.2188	0.3125	0.3125	0.3125	0.3125	0.3125
IgM	0.1563	0.5	0.5	0.2188	0.4063	0.4063
TNF	0.5	0.5	0.3125	0.0625	0.0313	0.0313
IFNγ	0.0313	0.0313	0.0625	0.0313	0.0313	0.0313
IL-6	0.4063	0.5	0.2188	0.0938	0.0313	0.0313
IL-23p19	0.3125	0.3125	0.3125	0.2188	0.4063	0.5

Appendix Table S10. (**A-B**) Statistical summary and p-values of Figure 3.

A. Statistical summary and p-values Figure 3E, (frequency of CD45^{+} LPMCs in UC, CD and non-inflamed)

EdgeR statistical framework with negative binomial GLM and a false discovery rate adjusted to 10% Benjiamini-Hochberg

Non-Inflamed + P/I compared to UC + P/I		
Cluster_id	p_val	p_adj
CD4+CD45RA+	0.0695	0.116
CD8+CD45RA+	0.00172	0.00388
CD4+CD45RO+	4.70E-06	2.35E-05
CD8+CD45RO+	0.0151	0.0302
CD4+CD45RO+IL7R+	4.23E-05	0.000141
Tregs	2.55E-07	2.55E-06
C11b+CD11c+	0.582	0.672
CD11c+CD38+	0.173	0.247
CD14+HLA-DR+	0.853	0.853
NKcells	0.639	0.672
CD19+CD38+IL7R+	0.406	0.515
CD19+HLA-DR+IgM-	0.613	0.672
CD19low	4.43E-06	2.35E-05
CD38+HLA-DR+IgA-	6.56E-11	1.31E-09
CD38+HLA-DR+IgA+	0.412	0.515
CD38+IgM+	2.06E-05	8.25E-05
CD38+	0.0379	0.069
CD38+IgA+	0.000381	0.00109
CD38+IgM-	0.00175	0.00388
CD19+HLA-DR+IgM+	0.109	0.168

Non-Inflamed + P/I compared to CD + P/I		
Cluster_id	p_val	p_adj
CD4+CD45RA+	0.147	0.419
CD8+CD45RA+	0.00161	0.0108
CD4+CD45RO+	0.0016	0.0108
CD8+CD45RO+	0.751	0.835
CD4+CD45RO+IL7R+	0.323	0.496
Tregs	6.18E-05	0.00124
C11b+CD11c+	0.285	0.488
CD11c+CD38+	0.618	0.727
CD14+HLA-DR+	0.97	0.97
NKcells	0.124	0.412
CD19+CD38+IL7R+	0.172	0.431
CD19+HLA-DR+IgM-	0.0761	0.304
CD19low	0.0685	0.304
CD38+HLA-DR+IgA-	0.58	0.727
CD38+HLA-DR+IgA+	0.593	0.727
CD38+IgM+	0.246	0.488
CD38+	0.293	0.488
CD38+IgA+	0.208	0.461
CD38+IgM-	0.58	0.727
CD19+HLA-DR+IgM+	0.887	0.934

UC + P/I compared to CD + P/I		
cluster_id	p_val	p_adj
CD4+CD45RA+	0.685	0.761
CD8+CD45RA+	0.983	0.983
CD4+CD45RO+	0.112	0.249
CD8+CD45RO+	0.0182	0.0521
CD4+CD45RO+IL7R+	0.000517	0.00357
Tregs	0.201	0.334
C11b+CD11c+	0.567	0.668
CD11c+CD38+	0.346	0.462
CD14+HLA-DR+	0.871	0.916
NKcells	0.237	0.361
CD19+CD38+IL7R+	0.559	0.668
CD19+HLA-DR+IgM-	0.157	0.286
CD19low	0.00199	0.00993
CD38+HLA-DR+IgA-	1.21E-10	2.43E-09
CD38+HLA-DR+IgA+	0.134	0.268
CD38+IgM+	0.000536	0.00357
CD38+	0.253	0.361
CD38+IgA+	0.0104	0.0346
CD38+IgM-	0.00413	0.0165
CD19+HLA-DR+IgM+	0.0513	0.128

B. Statistical summary and p-values Figure 3F, (expression levels fold change normalized to non-inflamed samples)

Unpaired t test corrected with FDR adjustment at 1% using Benjamini, Krieger and Yekutieli procedure. Tables indicate adjusted p-values.

UC + P/I/Non-inflamed vs Non-inflamed/Non-inflamed.										
Cluster_id	1	2	3	4	5	6	7	8	9	10
CD27	0.4902	0.9073	0.1502	0.5882	0.5039	0.0871	0.6580	0.9920	0.7536	0.5507
CD25	0.2160	0.6100	0.8648	0.8676	0.4572	0.0007	0.0307	0.1383	0.0226	0.1755
IL-7R	0.9664	0.3471	0.4746	0.5170	0.3716	0.1493	0.1106	0.6883	0.6893	0.1209
CD40	0.4301	0.0027	0.5498	0.9481	0.6518	0.1389	0.1344	0.8771	0.1249	0.8888
CD40L	0.7809	0.9400	0.0699	0.7155	0.4011	0.0221	0.4948	0.8271	0.3745	0.0726
CD137	0.4228	0.4970	0.3754	0.7035	0.7853	0.1637	0.8862	0.5650	0.6293	0.9213
FasL	0.3353	0.4713	0.1708	0.3952	0.8545	0.9171	0.7505	0.9565	0.8462	0.5462
Fas	0.9547	0.8835	0.0004	0.1688	0.0005	0.5711	0.0024	0.1597	0.0726	0.1599
HLA-DR	0.3495	0.0001	0.5350	0.0789	0.7621	0.4788	0.0000	0.1666	0.5875	0.8261

PD-1	0.6370	0.1649	0.2383	0.9269	0.1829	0.3679	0.0222	0.1201	0.5025	0.6880
CD86	0.7258	0.8284	0.5008	0.0324	0.3733	0.0121	0.0760	0.1230	0.4716	0.8798
CD103	0.6643	0.6884	0.0946	0.4168	0.8017	0.8414	0.0287	0.9232	0.9580	0.8004
TNF	0.3853	0.9043	0.7358	0.9156	0.1435	0.0500	0.0633	0.9284	0.0280	0.9922
IFNγ	0.2883	0.6971	0.0120	0.3751	0.3560	0.2435	0.8123	0.4474	0.8792	0.0911
IL-2	0.2301	0.8835	0.1361	0.1163	0.0159	0.0397	0.4491	0.9605	0.9894	0.3589
IL-4	0.3186	0.9824	0.3089	0.5748	0.7465	0.5306	0.8461	0.1121	0.4133	0.6523
IL-6	0.0024	0.8308	0.0000	0.3587	0.0000	0.0000	0.0060	0.7359	0.2833	0.4130
IL-17A	0.5146	0.5859	0.1316	0.3469	0.5538	0.3825	0.3699	0.6859	0.8692	0.4906
IL-13	0.5695	0.4207	0.2313	0.5038	0.9448	0.9421	0.9453	0.9410	0.5845	0.9319
IL-23p19	0.7369	0.4950	0.4305	0.0545	0.6351	0.9407	0.9081	0.6224	0.3424	0.1825
UC + P/I/Non-inflamed vs Non-inflamed/Non-inflamed										
Cluster_id	11	12	13	14	15	16	17	18	19	20
CD27	0.4922	0.6830	0.8809	0.0584	0.0003	0.0484	0.0000	0.0000	0.1241	0.1177
CD25	0.0206	0.0532	0.3840	0.5477	0.4345	0.0583	0.5538	0.1393	0.4772	0.7789
IL-7R	0.9714	0.6452	0.8760	0.7855	0.4117	0.4760	0.4190	0.0578	0.7307	0.2477
CD40	0.4626	0.9517	0.5906	0.6542	0.6896	0.7998	0.6024	0.9496	0.7013	0.6903
CD40L	0.8183	0.4887	0.7734	0.7990	0.6218	0.1577	0.4410	0.7484	0.6765	0.9724
CD137	0.8380	0.9168	0.7283	0.7908	0.6362	0.3155	0.9330	0.0023	0.8896	0.5743
FasL	0.6233	0.9041	0.8830	0.8136	0.2407	0.1089	0.3531	0.7897	0.5501	0.3970
Fas	0.1018	0.2233	0.8905	0.6301	0.4048	0.7703	0.5881	0.4000	0.5489	0.8548
HLA-DR	0.0072	0.0490	0.2673	0.0863	0.8528	0.9471	0.2319	0.0275	0.0306	0.7059
PD-1	0.0595	0.0071	0.7580	0.0000	0.0000	0.0000	0.0460	0.0000	0.5043	0.0000
CD86	0.2293	0.4727	0.7075	0.6769	0.7637	0.4500	0.9677	0.8105	0.3052	0.7259
CD103	0.7424	0.5818	0.7505	0.3080	0.0755	0.0080	0.1924	0.1884	0.7345	0.9820
TNF	0.3148	0.3456	0.5233	0.9964	0.8411	0.9019	0.0964	0.7464	0.0706	0.0000
IFNγ	0.8474	0.6028	0.4652	0.9658	0.0323	0.0218	0.0516	0.9686	0.1411	0.1042
IL-2	0.7098	0.3840	0.8397	0.2773	0.5774	0.8367	0.0855	0.1951	0.0988	0.4769
IL-4	0.1202	0.1169	0.3505	0.3829	0.3656	0.2558	0.4589	0.6721	0.5971	0.1701
IL-6	0.0756	0.6144	0.2411	0.8781	0.1940	0.0339	0.9392	0.6399	0.3781	0.6559
IL-17A	0.8913	0.9949	0.8809	0.9414	0.7246	0.9915	0.7216	0.0099	0.4046	0.0000
IL-13	0.7098	0.9887	0.5886	0.7604	0.2219	0.1151	0.8493	0.6840	0.7488	0.5985
IL-23p19	0.5056	0.6206	0.5721	0.9656	0.8059	0.9480	0.8852	0.7310	0.8149	0.9963

CD + P/I/Non-inflamed vs Non-inflamed/Non-inflamed										
Cluster_id	1	2	3	4	5	6	7	8	9	10
CD27	0.0456	0.1772	0.8428	0.3942	0.165514	0.000011	0.676287	0.992015	0.8214	0.9392
CD25	0.7869	0.2076	0.5038	0.2628	0.652584	0.859094	0.061251	0.138256	0.0504	0.1736
IL-7R	0.5921	0.8042	0.9433	0.7659	0.862739	0.838884	0.550785	0.688341	0.9134	0.2251
CD40	0.2439	0.2154	0.0499	0.5468	0.345286	0.255353	0.550622	0.87708	0.6432	0.3165
CD40L	0.3697	0.0007	0.0889	0.7253	0.381685	0.594594	0.752087	0.827142	0.9186	0.2007
CD137	0.1075	0.6489	0.0583	0.5595	0.701448	0.871841	0.107433	0.564972	0.4404	0.1057
FasL	0.2241	0.5518	0.2395	0.1733	0.630999	0.970839	0.620724	0.956458	0.7706	0.8965
Fas	0.1838	0.6466	0.0679	0.2446	0.787755	0.155874	0.004704	0.159726	0.9581	0.5055
HLA-DR	0.3288	0.7046	0.6637	0.8862	0.900377	0.98206	0.066342	0.166551	0.9885	0.9553
PD-1	0.1538	0.5939	0.3009	0.9481	0.617853	0.9188	0.659834	0.120144	0.8657	0.9545
CD86	0.4831	0.4451	0.5622	0.8582	0.240593	0.712114	0.162164	0.123026	0.5081	0.727
CD103	0.1682	0.3465	0.4713	0.239	0.306854	0.877993	0.399629	0.923198	0.001	0.1104
TNF	0.2685	0.2153	0.8716	0.7855	0.828529	0.102389	0.042554	0.928385	0.5515	0.8552
IFNγ	0.0901	0.9312	0.0463	0.6241	0.507336	0.72558	0.967209	0.447413	0.7825	0.1841
IL-2	0.4328	0.0061	0.4127	0.6684	0.385956	0.469758	0.000503	0.960516	0.7388	0.2679
IL-4	0.1689	0.6515	0.4517	0.7715	0.134212	0.002216	0.875446	0.112148	0.8742	0.9313
IL-6	0.4529	0.3849	0.0063	0.994	2.4E-09	0.239486	2.16E-06	0.735904	0.1166	0.6057
IL-17A	0.1785	0.8788	0.1649	0.3943	0.833509	0.891776	0.43766	0.68589	0.6721	0.1271
IL-13	0.5349	0.111	0.1279	0.8904	0.933968	0.776834	0.860382	0.940976	0.538	0.9269
IL-23p19	0.3012	0.4914	0.9516	0.0163	0.641375	0.008443	0.663567	0.622386	0.5544	0.3631
CD + P/I/Non-inflamed vs Non-inflamed/Non-inflamed										
Cluster_id	11	12	13	14	15	16	17	18	19	20
CD27	0.0689	0.852	0.0193	0.014	0.109	0.3746	0.0032	0.033066	0.89131	0.162565
CD25	0.107	0.0005	< 0.0001	0.4009	0.4401	0.1075	0.0792	0.426319	0.633633	0.438779
IL-7R	0.3501	0.6584	0.1517	0.4869	0.7971	0.612	0.5981	0.113542	0.206855	0.047464
CD40	0.9347	0.7243	0.2389	0.6649	0.8501	0.7479	0.4066	0.992941	0.438923	0.752206
CD40L	0.9234	0.7592	0.9218	0.5938	0.2333	0.1241	0.9839	0.118336	0.753463	0.123606
CD137	0.6831	0.8363	0.6356	0.9707	0.9412	0.5189	0.98	0.055543	0.463485	0.814132

FasL	0.6926	0.9115	0.9784	0.4504	0.578	0.5235	0.4888	0.510294	0.11697	0.765646
Fas	0.3814	0.9573	0.3966	0.4519	0.7721	0.7739	0.3393	0.846654	0.676637	0.982818
HLA-DR	0.4814	0.9498	0.4783	0.7818	0.8475	0.9951	0.0317	0.823154	0.24378	0.14365
PD-1	0.6217	0.7637	0.3683	0.4312	0.406	0.43	0.8352	0.000138	0.332391	0.083044
CD86	0.5411	0.738	0.5706	0.4461	0.704	0.7661	0.435	0.828404	0.149871	0.949512
CD103	0.6963	0.0656	0.5507	0.0907	0.5545	0.365	0.2084	0.280444	0.316539	0.736402
TNF	0.4293	0.7815	0.0049	0.5908	0.657	0.4162	0.4694	0.532189	0.000243	3.65E-05
IFNγ	0.5939	0.7995	0.9059	0.6158	0.2805	0.7417	0.232	0.707476	0.02753	0.265431
IL-2	0.7777	0.6345	0.6906	0.105	0.6593	0.285	0.5357	0.549127	0.000001	5.25E-05
IL-4	0.2735	0.6821	0.0084	0.0068	0.6693	0.7757	0.15	0.65138	0.026004	0.205619
IL-6	0.1309	0.8379	0.1387	0.7672	0.0491	0.0386	0.8896	0.036529	0.342005	0.787927
IL-17A	0.9952	0.5072	0.9242	0.6557	0.0886	0.155	0.585	0.049947	0.31399	7.23E-09
IL-13	0.8501	0.7126	0.8391	0.5734	0.2228	0.1111	0.6269	0.767436	0.579215	0.486469
IL-23p19	0.5799	0.8978	0.7091	0.4812	0.5361	0.284	0.5412	0.786612	0.790353	0.791805

(CD + P/I)/(CD + P/I) vs (CD + P/I)/(UC + P/I)										
Cluster_id	1	2	3	4	5	6	7	8	9	10
CD27	0.309228	0.314398	0.253747	0.576072	0.544059	3.5E-14	0.994609	0.22771	0.696996	0.760231
CD25	0.00123	0.044747	0.678659	0.199018	0.492984	7.57E-09	0.845249	0.103591	0.419432	0.843723
IL-7R	0.571406	2.28E-05	0.222365	0.867367	0.21222	0.448859	0.277185	0.332172	0.534888	0.988062
CD40	0.076611	0.000755	0.324609	0.636133	0.661436	0.992682	0.317389	0.852212	0.837081	0.314159
CD40L	0.158866	0.001878	0.183595	0.643166	0.824353	0.053935	0.377721	0.31875	0.56238	0.839396
CD137	0.759828	0.000689	0.562557	0.376718	0.413733	0.240183	0.000413	0.851072	0.664634	0.166872
FasL	0.518284	0.047412	0.334396	0.682125	0.761756	0.965073	0.08008	0.452915	0.593762	0.726035
Fas	0.181824	0.440022	0.176915	0.066257	0.004066	0.040936	0.950223	0.089569	0.052918	0.766994
HLA-DR	0.668639	0.000904	0.662389	0.04331	0.783842	0.63181	0.03214	0.031261	0.256417	0.921963
PD-1	0.491579	0.088585	0.466108	0.736664	0.310555	0.596986	0.080024	0.011693	0.871875	0.718921
CD86	0.167747	0.136249	0.080655	3.42E-05	0.875941	0.009938	0.740199	0.038936	0.303998	0.834717
CD103	0.095682	0.935884	0.00649	0.972121	0.407293	0.998684	0.200796	0.23381	0.364239	0.193556
TNF	0.592792	0.096947	0.519724	0.801631	0.000087	7.7E-06	0.773123	0.390113	0.297054	0.867239
IFNγ	0.837802	0.17654	5.7E-06	0.128345	0.461822	0.497666	0.745376	0.000221	0.674682	0.993729
IL-2	1.79E-06	0.000689	0.057067	0.626559	<0.000001	0.000728	4.56E-05	0.605801	0.426766	0.671183
IL-4	0.611708	0.613114	0.435641	0.872789	0.209234	0.017283	0.477267	0.054438	0.368532	0.724964
IL-6	0.011662	0.703229	0.120903	0.044761	0.293914	0.122711	0.045256	0.452095	0.266832	0.21297
IL-17A	0.869577	0.343992	0.328844	0.298343	0.590434	0.630108	0.911868	0.762841	0.327725	0.307801
IL-13	0.673731	0.455273	0.967677	0.626666	0.9991	0.729316	0.633954	0.607373	0.159819	0.891074
IL-23p19	0.711823	0.104776	0.227232	0.23767	0.14505	0.010177	0.273065	0.304535	0.245438	0.858666
(CD + P/I)/(CD + P/I) vs (CD + P/I)/(UC + P/I)										
Cluster_id	11	12	13	14	15	16	17	18	19	20
CD27	0.417332	0.471685	0.454935	0.6126	0.150369	0.422462	0.270895	0.021338	0.89131	0.524258
CD25	0.185763	0.26526	0.525234	0.861354	0.852424	0.777223	0.270313	0.307546	0.633633	0.587013
IL-7R	0.247183	0.267498	0.439665	0.91941	0.306357	0.771742	0.70528	0.000916	0.206855	0.331961
CD40	0.157853	0.758068	0.936562	0.776221	0.721292	0.962066	0.74434	0.909052	0.438923	0.19593
CD40L	0.593696	0.727225	0.742224	0.590811	0.643615	0.991023	0.204498	0.21817	0.753463	0.024646
CD137	0.433647	0.915409	0.434089	0.778201	0.547947	0.597829	0.935391	0.15948	0.463485	0.117758
FasL	0.736292	0.99207	0.858309	0.849047	0.154907	0.109998	0.568768	0.172849	0.11697	0.353113
Fas	0.006953	0.348677	0.736886	0.255764	0.294808	0.598508	0.638817	0.197435	0.676637	0.728202
HLA-DR	0.001237	0.156798	0.348486	0.089575	0.694962	0.95208	0.166785	0.021379	0.24378	0.129971
PD-1	0.010044	0.064766	0.281585	<0.000001	0.007531	0.0293	0.058257	0.00315	0.332391	0.00137
CD86	0.301737	0.345544	0.869768	0.428774	0.418515	0.211693	0.325565	0.47096	0.149871	0.427107
CD103	0.921603	0.002589	0.506543	0.778799	0.000474	0.000005	0.685061	0.116731	0.316539	0.666726
TNF	0.524164	0.298284	0.646125	0.784732	0.473721	0.536973	0.012974	0.146688	0.000243	0.340414
IFNγ	0.320021	0.737629	0.362576	0.82852	6.56E-06	0.000035	0.011683	0.700582	0.02753	0.319694
IL-2	0.289494	0.731071	0.655041	0.65684	0.182149	0.450022	0.003494	0.012486	3.12E-07	0.000154
IL-4	0.319384	0.383634	0.883286	0.710633	0.328249	0.238075	0.556088	0.145103	0.026004	0.593269
IL-6	0.375481	0.777304	0.571305	0.745801	2.02E-08	<0.000001	0.786439	0.000101	0.342005	0.182789
IL-17A	0.82693	0.480409	0.88341	0.755887	0.279042	0.189214	0.854037	0.288738	0.31399	0.091236
IL-13	0.648022	0.680418	0.535054	0.977933	0.583936	0.878962	0.384212	0.709543	0.579215	0.978953
IL-23p19	0.673095	0.555769	0.37982	0.691342	0.333194	0.279905	0.612665	0.780548	0.790353	0.716588

Appendix Table S11. (**A-C**) Statistical summary and p-values of Figure 4.

A. Statistical summary and p-values Figure 4A, (frequency of CD45^{+}CD3^{+} LPMCs in UC, CD and non-inflamed)

EdgeR statistical framework with negative binomial GLM and a false discovery rate adjusted to 10% Benjiamini-Hochberg

Non-inflamed + P/I compared to UC + P/I		
cluster_id	p_val	p_adj
(18) CD8^{+}PD1^{+}	1.68E-06	1.68E-05
(4) CD8^{+}CD45RA^{+}IFNγ^{+}	0.000432	0.00144
(17) CD8^{+}	0.284	0.365
(11) CD4^{+}IL-17A^{+}	4.30E-05	0.000215
(14) CD4^{+}PD1^{+}	0.00127	0.00362
(20) Tregs	1.36E-05	9.04E-05
(15) CD4^{+}TNF^{+}	0.000157	0.000627
(7) CD4^{+}IL7R^{high}TNF^{+}	0.0823	0.137
(10) CD4^{+}	0.292	0.365
(3) CD4^{+}CD45RA^{+}IL-2^{+}	0.789	0.831
(1) CD4^{+}CD45RA^{+}CD40L^{-}	0.039	0.0781
(2) CD4^{+}CD45RA^{+}CD40L^{+}	0.125	0.179
(6) CD4^{+}IL7R^{high}IL-2^{+}TNF^{+}IL-17A^{+}	0.0296	0.0658
(12) CD4^{+}IL-2^{+}TNF^{+}	0.324	0.381
(13) CD4^{+}IL-4^{+}IL-13^{+}	6.40E-10	1.28E-08
(8) CD8^{+}IL7R^{high}IL-2^{+}TNF^{+}	0.113	0.174
(9) CD8^{+}IL7R^{high}IL-2^{+}TNF^{+}IFNγ^{+}	0.0611	0.111
(19) CD8^{+}TNF^{+}IFNγ^{+}	0.503	0.559
(5) CD4^{+}IL7R^{high}IL-2^{+}TNF^{+}IFNγ^{+}	0.00576	0.0144
(16)CD4^{+}TNF^{+}IFNγ^{+}	0.955	0.955

Non-inflamed + P/I compared to CD + P/I		
cluster_id	p_val	p_adj
(18) CD8^{+}PD1^{+}	0.0336	0.134
(4) CD8^{+}CD45RA^{+}IFNγ^{+}	0.00249	0.024
(17) CD8^{+}	0.471	0.607
(11) CD4^{+}IL-17A^{+}	0.0118	0.0589
(14) CD4^{+}PD1^{+}	0.374	0.575
(20) Tregs	0.0036	0.024
(15) CD4^{+}TNF^{+}	0.0455	0.152
(7) CD4^{+}IL7R^{high}TNF^{+}	0.312	0.52
(10) CD4^{+}	0.289	0.52
(3) CD4^{+}CD45RA^{+}IL-2^{+}	0.721	0.759
(1) CD4^{+}CD45RA^{+}CD40L^{-}	0.304	0.52
(2) CD4^{+}CD45RA^{+}CD40L^{+}	0.101	0.288
(6) CD4^{+}IL7R^{high}IL-2^{+}TNF^{+}IL-17A^{+}	0.697	0.759
(12) CD4^{+}IL-2^{+}TNF^{+}	0.242	0.52
(13) CD4^{+}IL-4^{+}IL-13^{+}	0.000161	0.00322
(8) CD8^{+}IL7R^{high}IL-2^{+}TNF^{+}	0.464	0.607
(9) CD8^{+}IL7R^{high}IL-2^{+}TNF^{+}IFNγ^{+}	0.77	0.77
(19) CD8^{+}TNF^{+}IFNγ^{+}	0.211	0.52
(5) CD4^{+}IL7R^{high}IL-2^{+}TNF^{+}IFNγ^{+}	0.691	0.759
(16)CD4^{+}TNF^{+}IFNγ^{+}	0.486	0.607

UC + P/I compared to CD + P/I		
cluster_id	p_val	p_adj
(18) CD8^{+}PD1^{+}	0.00268	0.0351
(4) CD8^{+}CD45RA^{+}IFNγ^{+}	0.58	0.773
(17) CD8^{+}	0.695	0.869
(11) CD4^{+}IL-17A^{+}	0.0791	0.176
(14) CD4^{+}PD1^{+}	0.00914	0.0351
(20) Tregs	0.108	0.217
(15) CD4^{+}TNF^{+}	0.0469	0.134
(7) CD4^{+}IL7R^{high}TNF^{+}	0.416	0.64
(10) CD4^{+}	0.994	0.994
(3) CD4^{+}CD45RA^{+}IL-2^{+}	0.92	0.968
(1) CD4^{+}CD45RA^{+}CD40L^{-}	0.248	0.45
(2) CD4^{+}CD45RA^{+}CD40L^{+}	0.905	0.968
(6) CD4^{+}IL7R^{high}IL-2^{+}TNF^{+}IL-17A^{+}	0.00417	0.0351
(12) CD4^{+}IL-2^{+}TNF^{+}	0.837	0.968
(13) CD4^{+}IL-4^{+}IL-13^{+}	0.00584	0.0351
(8) CD8^{+}IL7R^{high}IL-2^{+}TNF^{+}	0.0105	0.0351
(9) CD8^{+}IL7R^{high}IL-2^{+}TNF^{+}IFNγ^{+}	0.0772	0.176
(19) CD8^{+}TNF^{+}IFNγ^{+}	0.515	0.736
(5) CD4^{+}IL7R^{high}IL-2^{+}TNF^{+}IFNγ^{+}	0.00825	0.0351
(16)CD4^{+}TNF^{+}IFNγ^{+}	0.4	0.64

B. Statistical summary and p-values Figure 4B, (frequency of CD45^{+}CD3^{+} LPMCs in UC and CD samples ± 1µM BTP2)

One-tailed Wilcoxon matched-pairs signed rank test

UC + P/I compared to UC + P/I + BTP2	
cluster_id	p_val
(1) CD4^{+}CD45RA^{+}CD40L^{-}	0.0313
(2) CD4^{+}CD45RA^{+}CD40L^{+}	0.3125
(3) CD4^{+}CD45RA^{+}IL-2^{+}	0.0313
(4) CD8^{+}CD45RA^{+}IFNγ^{+}	0.0313

CD + P/I compared to CD + P/I + BTP2	
cluster_id	p_val
(1) CD4^{+}CD45RA^{+}CD40L^{-}	0.0313
(2) CD4^{+}CD45RA^{+}CD40L^{+}	0.0625
(3) CD4^{+}CD45RA^{+}IL-2^{+}	0.0313
(4) CD8^{+}CD45RA^{+}IFNγ^{+}	0.1563

(10) CD4$^+$	0.0313
(17) CD8$^+$	0.0313
(12) CD4$^+$IL-2$^+$TNF$^+$	0.0313
(15) CD4$^+$TNF$^+$	0.0625
(16)CD4$^+$TNF$^+$IFNγ$^+$	0.0938
(19) CD8$^+$TNF$^+$IFNγ$^+$	0.0938
(13) CD4$^+$IL-4$^+$IL-13$^+$	0.0313
(11) CD4$^+$IL-17A$^+$	0.0625
(20) Tregs	0.0625
(5) CD4$^+$IL7R^{high}IL-2$^+$TNF$^+$IFNγ$^+$	0.0313
(6) CD4$^+$IL7R^{high}IL-2$^+$TNF$^+$IL-17A$^+$	0.0313
(7) CD4$^+$IL7R^{high}TNF$^+$	0.0313
(8) CD8$^+$IL7R^{high}IL-2$^+$TNF$^+$	0.0625
(9) CD8$^+$IL7R^{high}IL-2$^+$TNF$^+$IFNγ$^+$	0.0313
(14) CD4$^+$PD1$^+$	0.0313
(18) CD8$^+$PD1$^+$	0.0313

(10) CD4$^+$	0.0313
(17) CD8$^+$	0.0313
(12) CD4$^+$IL-2$^+$TNF$^+$	0.0313
(15) CD4$^+$TNF$^+$	0.8438
(16)CD4$^+$TNF$^+$IFNγ$^+$	> 0.0999
(19) CD8$^+$TNF$^+$IFNγ$^+$	0.4375
(13) CD4$^+$IL-4$^+$IL-13$^+$	0.0313
(11) CD4$^+$IL-17A$^+$	0.0938
(20) Tregs	0.0313
(5) CD4$^+$IL7R^{high}IL-2$^+$TNF$^+$IFNγ$^+$	0.0313
(6) CD4$^+$IL7R^{high}IL-2$^+$TNF$^+$IL-17A$^+$	0.0313
(7) CD4$^+$IL7R^{high}TNF$^+$	0.0313
(8) CD8$^+$IL7R^{high}IL-2$^+$TNF$^+$	0.0313
(9) CD8$^+$IL7R^{high}IL-2$^+$TNF$^+$IFNγ$^+$	0.0313
(14) CD4$^+$PD1$^+$	0.0313
(18) CD8$^+$PD1$^+$	0.0313

C. Statistical summary and p-values Figure 4D, (expression levels fold change normalized to samples treated only with P/I)

One-tailed Wilcoxon matched-pairs signed rank test. Tables indicate individual p-values.

UC + P/I + BTP2 vs UC + P/I										
Cluster_id	1	2	3	4	5	6	7	8	9	10
CD45RA	0.1563	0.0313	0.0313	0.2188	0.0625	0.4375	0.8438	0.8438	0.0625	0.6875
CD45RO	0.8438	0.0313	0.4375	0.2188	0.6875	0.5625	0.4375	0.0938	0.0313	0.1563
CCR7	0.0625	0.0313	0.6875	0.3125	0.8438	0.6875	0.0938	0.0313	0.0625	0.0313
CD27	0.0313	0.1563	0.3125	0.3125	0.3125	0.6875	0.0313	0.8438	0.5625	0.3125
IL-7R	0.0313	0.6875	>0.999	0.8438	>0.999	0.0313	0.0938	0.1563	0.5625	0.0938
CD25	0.0625	0.0313	0.5625	0.0625	0.8438	0.4375	0.0313	0.6875	>0.999	0.0938
CD137	0.0313	0.0313	0.5625	0.0625	0.4375	0.5625	0.5625	>0.999	0.0625	0.2188
CD86	0.1563	0.4375	>0.999	0.5625	0.3125	0.4375	0.5625	0.1563	0.0938	0.6875
CD40	0.5625	0.3125	0.4375	0.6865	0.5625	0.5625	0.0625	0.0938	0.8438	>0.999
CD40L	0.3125	0.0313	0.0313	0.0313	0.0313	0.0938	0.1563	0.4375	0.3125	0.5625
HLA-DR	0.2188	0.8438	0.3125	0.3125	0.4375	>0.999	0.3125	0.4375	0.8438	0.0313
Fas	0.0625	0.6875	0.4375	0.3125	>0.999	0.8438	>0.999	0.4375	>0.999	0.2188
FasL	0.0313	0.0313	0.1563	0.5163	0.0313	0.0313	0.2188	>0.999	0.8438	>0.999
PD-1	0.0313	0.3125	0.8438	0.5625	0.3125	0.0625	0.3125	>0.999	0.2188	0.8438
TNF	0.4375	0.0313	0.0625	0.6875	0.0313	0.0313	0.0625	0.0313	0.0313	0.0313
IFNγ	0.0313	0.1563	0.5625	0.0938	0.0313	0.0313	0.4375	0.1563	0.0313	0.4375
IL-2	0.0938	0.0313	0.0313	0.0313	0.0625	0.0313	0.0313	0.0313	0.0938	0.0313
IL-4	0.6845	0.5625	>0.999	0.5625	0.0625	0.5625	0.6875	0.0625	0.0313	0.4375
IL-13	0.3125	0.3125	0.5625	0.1563	0.0625	>0.999	0.8438	0.1563	0.8438	>0.999
IL-17A	0.4375	0.8438	0.8438	0.8438	0.4375	0.0313	0.0313	0.0938	0.1563	0.8438
IL-6	>0.999	0.3125	0.5625	0.8438	0.8438	0.1563	0.8438	0.4375	0.2188	0.4375
IL-23p19	0.6875	0.0313	0.8438	0.3125	0.6875	>0.999	0.2188	0.5625	>0.999	0.0938
UC + P/I + BTP2 vs UC + P/I										
Cluster_id	11	12	13	14	15	16	17	18	19	20
CD45RA	0.3125	0.3125	0.1563	0.6875	0.6875	0.8438	>0.999	0.4375	0.0313	0.3125
CD45RO	>0.999	0.8438	0.0625	0.8438	0.6875	0.8438	0.4375	0.3125	0.4375	0.8438
CCR7	>0.999	0.0313	0.0625	0.4375	0.0313	0.1563	0.0625	0.2188	0.0625	0.3125
CD27	0.0313	0.0313	0.0625	0.8438	0.1563	0.4375	0.1563	0.0313	0.5625	0.0313
IL-7R	0.0313	0.0313	0.0625	0.1563	0.0313	0.0625	0.0938	>0.999	0.0313	0.0938
CD25	0.1563	0.0313	0.0625	0.0313	0.0313	>0.999	0.1563	0.8438	0.0625	0.5625
CD137	0.0625	0.2188	0.3125	0.8438	0.1563	0.0313	0.0313	0.0938	0.0313	0.0313
CD86	0.5625	0.1563	0.3125	0.6875	0.4375	0.1563	0.2188	0.6875	0.4375	0.3125
CD40	0.5625	0.6875	0.0313	0.6875	0.3125	>0.999	0.6875	>0.999	0.8438	0.6875
CD40L	0.0313	0.0625	0.0625	0.1563	0.0938	0.1563	0.4375	0.2188	0.1563	0.0313
HLA-DR	0.0625	0.8438	0.0625	0.8438	0.8438	0.6875	>0.999	>0.999	0.6875	0.4375
Fas	0.6875	0.4375	0.0625	0.1563	0.0313	0.0625	0.0625	>0.999	0.8438	0.8438
FasL	0.0313	0.0938	0.0625	0.2188	0.4375	0.1563	>0.999	>0.999	0.3125	0.4375
PD-1	0.0625	0.0313	0.0313	0.0313	0.0313	0.0313	0.6875	0.3125	0.2188	0.0313
TNF	0.0313	0.0313	0.0313	0.0313	0.0313	0.0313	0.0625	0.6875	0.0313	0.0313

IFNγ	0.5163	0.5625	0.1250	0.0313	0.8438	0.0313	0.0625	0.0625	0.0313	0.3125
IL-2	0.0625	0.0313	0.0313	0.0313	0.0313	0.1563	0.0625	0.0625	0.0625	0.2188
IL-4	0.0625	0.0313	0.0313	0.5625	0.1563	0.0313	>0.999	>0.999	0.0313	0.2188
IL-13	0.3125	0.8438	0.0625	>0.999	0.1563	0.1563	0.3125	0.4375	0.4375	0.2188
IL-17A	0.0313	0.4375	0.0625	0.6875	0.6875	0.0938	0.6875	0.6875	0.0313	0.1563
IL-6	0.0313	0.0313	0.0313	0.4375	0.0313	0.8438	0.2188	>0.999	>0.999	0.098
IL-23p19	0.8438	0.0313	0.0625	0.6875	0.0313	0.6875	0.4375	0.4375	0.4375	0.4375

CD + P/I + BTP2 vs CD + P/I										
Cluster_id	1	2	3	4	5	6	7	8	9	10
CD45RA	0.5625	0.0313	0.0313	0.0938	0.3125	0.8438	0.4375	>0.999	0.1563	0.3125
CD45RO	>0.999	0.0313	0.5625	0.1563	0.3125	0.6875	0.1563	0.1563	0.0625	0.0313
CCR7	0.0313	0.0313	0.4375	0.6875	0.0938	0.3125	0.6875	0.8438	0.4375	0.0313
CD27	0.0625	0.0313	0.6875	0.0938	0.4375	0.1563	0.8438	>0.999	0.0313	0.2188
IL-7R	0.0625	0.5625	0.0313	0.4375	0.0938	0.4375	0.0625	0.0625	0.6875	0.0313
CD25	0.0625	0.0313	0.8438	0.0313	0.3125	>0.999	0.0313	0.8438	0.8438	0.2188
CD137	0.0625	0.8438	0.8438	0.8438	0.0313	0.0625	0.5625	0.4375	0.3125	0.0313
CD86	0.6875	>0.999	0.5625	0.8438	0.3125	0.5625	0.8438	0.8438	>0.999	>0.999
CD40	0.5625	0.6875	0.4375	0.0313	0.4375	0.2188	0.4375	0.8438	0.0938	>0.999
CD40L	0.0938	0.0313	0.0313	0.0313	0.1563	0.1563	0.5625	>0.999	0.8438	0.4375
HLA-DR	0.0625	0.8438	0.8438	0.0625	0.5625	0.5625	0.8438	>0.999	0.8438	0.0313
Fas	0.8438	0.0313	0.4375	0.0313	0.0313	0.0625	0.5625	0.8438	0.4375	0.4375
FasL	0.8438	0.0313	0.1563	0.0938	0.0313	0.0313	0.6875	0.0313	0.8438	0.0938
PD-1	0.3125	0.1563	0.3125	>0.999	0.0313	0.8438	0.6875	0.6875	0.3125	0.3125
TNF	0.0938	0.0313	0.0313	0.3125	0.0625	0.0313	0.4375	0.0313	0.4375	0.5625
IFNγ	0.1563	0.0625	0.8438	0.5625	0.1563	0.0938	0.0313	0.2188	0.0938	0.0313
IL-2	0.0625	0.0625	0.0313	0.0625	0.0938	0.0313	0.0313	0.0313	0.4375	0.0313
IL-4	>0.999	0.3125	>0.999	>0.999	0.2188	0.0625	0.6875	0.0313	0.0938	>0.999
IL-13	0.0625	>0.999	0.5625	0.6875	0.1563	0.5625	0.4375	0.6875	0.5625	0.2188
IL-17A	0.6875	0.5625	0.3125	0.0625	0.0313	0.4375	0.0313	0.0625	>0.999	0.6875
IL-6	0.4375	0.8438	0.125	>0.999	0.4375	0.3125	0.4375	0.3125	0.6875	0.4375
IL-23p19	0.3125	0.3125	>0.999	0.4375	0.0625	0.3125	0.3125	0.0313	0.0313	0.0313
CD + P/I + BTP2 vs CD + P/I										
Cluster_id	11	12	13	14	15	16	17	18	19	20
CD45RA	0.5625	0.2188	0.3125	0.8438	0.2188	0.1563	0.2188	>0.999	0.0313	0.1563
CD45RO	0.8438	0.8438	0.2188	0.4375	0.1563	0.8438	0.0313	0.0625	0.0313	0.6875
CCR7	0.6875	0.5625	0.4375	0.0313	0.0313	0.1563	0.0625	0.625	0.0313	0.0313
CD27	0.3125	0.1563	0.8438	0.3125	0.0938	0.0313	0.2188	0.8125	0.1563	0.0938
IL-7R	0.0313	0.0313	>0.999	0.0313	0.0313	0.0313	0.0313	0.125	0.0313	0.3125
CD25	0.8438	0.0625	0.5625	0.0313	>0.999	0.0313	0.0313	0.3125	0.0625	0.0625
CD137	0.1563	0.4375	0.8438	>0.999	0.0313	0.4375	0.1563	0.3125	0.0313	0.0313
CD86	0.8438	0.1563	>0.999	0.8438	0.8438	0.3125	>0.999	0.625	>0.999	0.5625
CD40	0.8438	0.4375	0.8438	>0.999	0.5625	0.4375	0.0625	0.125	0.0625	0.1563
CD40L	0.0625	0.0313	0.0313	0.0313	0.0313	0.5625	>0.999	0.3125	0.2188	0.0313
HLA-DR	0.0313	0.4375	0.4375	0.0625	>0.999	0.3125	0.3125	0.4375	0.2188	0.0625
Fas	0.2188	0.0938	0.8438	0.1563	0.4375	0.8438	0.1563	0.0625	0.1563	0.0938
FasL	0.0313	0.4375	>0.999	0.1563	0.0625	0.8438	0.0938	0.8125	0.5625	0.6875
PD-1	0.0313	0.2188	>0.999	0.0625	0.0313	0.0313	0.8438	0.4375	0.2188	0.0313
TNF	0.0313	0.0313	0.2188	0.1563	0.1563	0.0625	0.4375	0.8125	0.0313	0.0313
IFNγ	0.0313	0.3125	0.5625	0.6875	0.8438	0.0313	0.0313	0.4375	0.0313	0.0313
IL-2	0.0313	0.0313	0.1563	0.0313	0.0625	0.0313	0.0313	0.4375	0.0313	0.0313
IL-4	0.2188	0.0313	0.3125	0.5625	0.0313	0.0313	>0.999	0.625	0.0938	0.6875
IL-13	0.8438	0.4375	0.8438	>0.999	0.4375	>0.999	>0.999	0.3125	0.3125	0.4375
IL-17A	0.0313	0.0625	0.8438	0.4375	0.8438	0.0313	0.0938	>0.999	0.8438	0.6875
IL-6	0.0313	0.0313	>0.999	0.2188	0.0313	0.0625	0.1563	>0.999	0.6875	0.6875
IL-23p19	0.0313	0.0625	>0.999	0.3125	0.0313	0.0313	0.0313	0.125	0.0313	0.2188

Appendix Table S12. (**A-C**) Statistical summary and p-values of Figure 5.

A. Statistical summary and p-values Figure 5A, (frequency of $CD45^{+}CD3^{-}$ LPMCs in UC, CD and non-inflamed)

EdgeR statistical framework with negative binomial GLM and a false discovery rate adjusted to 10% Benjiamini-Hochberg

Non-inflamed + P/I compared to UC + P/I		
cluster_id	p_val	p_adj
(20) $CD11c^{+}CD38^{+}$	0.057	0.120
(13) $CD19^{+}CD38^{+}IL7R^{+}$	0.390	0.443
(5) $CD19^{+}HLA\text{-}DR^{+}IgM^{-}CD40^{high}$	0.132	0.209
(4) $CD19^{+}HLA\text{-}DR^{+}IgM^{-}CD40^{low}$	0.396	0.443
(6) $CD19^{+}HLA\text{-}DR^{+}IgM^{+}$	0.080	0.138
(15) ILC3	0.027	0.069
(2) $CD14^{+}HLA\text{-}DR^{+}$	0.029	0.069
(3) $CD14^{+}HLA\text{-}DR^{+}IL\text{-}6^{+}$	NA	NA
(12) $CD38^{+}IgM^{+}$	0.000	0.000
(11) $CD38^{+}IgM^{-}$	0.000	0.000
(7) $CD38^{+}HLA\text{-}DR^{+}IgA^{-}$	0.003	0.010
(10) $CD38^{+}HLA\text{-}DR^{+}IgA^{+}$	0.146	0.214
(8) $CD38^{+}IgA^{+}$	0.000	0.000
(9) $CD38^{+}IgA^{+}CD11b^{+}$	0.066	0.124
(17) NK $IFN\gamma^{+}$	0.246	0.311
(18) Nkcells	0.179	0.242
(16) ILCs	0.916	0.916
(1) $CD11b^{+}CD11c^{+}$	0.567	0.599
(14) $CD19^{low}IgA^{+}$	0.024	0.069
(19) $CD19^{low}IgA^{-}$	0.002	0.007

Non-inflamed + P/I compared to CD + P/I		
cluster_id	p_val	p_adj
(20) $CD11c^{+}CD38^{+}$	0.18	0.457
(13) $CD19^{+}CD38^{+}IL7R^{+}$	0.242	0.459
(5) $CD19^{+}HLA\text{-}DR^{+}IgM^{-}CD40^{high}$	0.267	0.462
(4) $CD19^{+}HLA\text{-}DR^{+}IgM^{-}CD40^{low}$	0.0148	0.14
(6) $CD19^{+}HLA\text{-}DR^{+}IgM^{+}$	0.836	0.87
(15) ILC3	0.00239	0.0454
(2) $CD14^{+}HLA\text{-}DR^{+}$	0.805	0.87
(3) $CD14^{+}HLA\text{-}DR^{+}IL\text{-}6^{+}$	NA	NA
(12) $CD38^{+}IgM^{+}$	0.333	0.487
(11) $CD38^{+}IgM^{-}$	0.159	0.457
(7) $CD38^{+}HLA\text{-}DR^{+}IgA^{-}$	0.51	0.646
(10) $CD38^{+}HLA\text{-}DR^{+}IgA^{+}$	0.87	0.87
(8) $CD38^{+}IgA^{+}$	0.217	0.457
(9) $CD38^{+}IgA^{+}CD11b^{+}$	0.199	0.457
(17) NK $IFN\gamma^{+}$	0.334	0.487
(18) Nkcells	0.0576	0.274
(16) ILCs	0.0358	0.226
(1) $CD11b^{+}CD11c^{+}$	0.114	0.432
(14) $CD19^{low}IgA^{+}$	0.756	0.87
(19) $CD19^{low}IgA^{-}$	0.359	0.487

UC + P/I compared to CD + P/I		
cluster_id	p_val	p_adj
(20) $CD11c^{+}CD38^{+}$	0.557	0.588
(13) $CD19^{+}CD38^{+}IL7R^{+}$	0.733	0.733
(5) $CD19^{+}HLA\text{-}DR^{+}IgM^{-}CD40^{high}$	0.00366	0.0174
(4) $CD19^{+}HLA\text{-}DR^{+}IgM^{-}CD40^{low}$	0.0763	0.121
(6) $CD19^{+}HLA\text{-}DR^{+}IgM^{+}$	0.0846	0.124
(15) ILC3	0.368	0.466
(2) $CD14^{+}HLA\text{-}DR^{+}$	0.0339	0.0643
(3) $CD14^{+}HLA\text{-}DR^{+}IL\text{-}6^{+}$	NA	NA
(12) $CD38^{+}IgM^{+}$	1.12E-05	0.000107
(11) $CD38^{+}IgM^{-}$	1.45E-10	2.76E-09
(7) $CD38^{+}HLA\text{-}DR^{+}IgA^{-}$	0.00837	0.0318
(10) $CD38^{+}HLA\text{-}DR^{+}IgA^{+}$	0.0763	0.121
(8) $CD38^{+}IgA^{+}$	0.000393	0.00249
(9) $CD38^{+}IgA^{+}CD11b^{+}$	0.526	0.588
(17) NK $IFN\gamma^{+}$	0.0188	0.0446
(18) Nkcells	0.538	0.588
(16) ILCs	0.0145	0.0394
(1) $CD11b^{+}CD11c^{+}$	0.264	0.358
(14) $CD19^{low}IgA^{+}$	0.0333	0.0643
(19) $CD19^{low}IgA^{-}$	0.0122	0.0385

B. Statistical summary and p-values Figure 5B, (frequency of $CD45^{+}CD3^{-}$ LPMCs in UC and CD samples ± 1µM BTP2)

One-tailed Wilcoxon matched-pairs signed rank test

UC + P/I compared to UC + P/I + BTP2	
cluster_id	p_val
(4) $CD19^{+}HLA\text{-}DR^{+}IgM^{-}$ $CD40^{low}$	0.1563
(5) $CD19^{+}HLA\text{-}DR^{+}IgM^{-}$ $CD40^{high}$	0.3125
(6) $CD19^{+}HLA\text{-}DR^{+}IgM^{+}$	0.6875
(19) $CD19^{low}IgA^{-}$	0.2188

CD + P/I compared to CD + P/I + BTP2	
cluster_id	p_val
(4) $CD19^{+}HLA\text{-}DR^{+}IgM^{-}$ $CD40^{low}$	0.2188
(5) $CD19^{+}HLA\text{-}DR^{+}IgM^{-}$ $CD40^{high}$	0.0313
(6) $CD19^{+}HLA\text{-}DR^{+}IgM^{+}$	0.6875
(19) $CD19^{low}IgA^{-}$	0.5625

(13) $CD19^{+}CD38^{+}IL7R^{+}$	0.8438
(14) $CD19^{low}IgA^{+}$	>0.999
(7) $CD38^{+}HLA\text{-}DR^{+}IgA^{-}$	0.5625
(9)$CD38^{+}IgA^{+}CD11b^{+}$	0.625
(10) $CD38^{+}HLA\text{-}DR^{+}IgA^{+}$	0.6875
(12)$CD38^{+}IgM^{+}$	0.4375
(8)$CD38^{+}IgA^{+}$	>0.999
(11)$CD38^{+}IgM^{-}$	0.5625
(15)ILC3	>0.999
(16)ILCs	0.8438
(17)NK $IFN\gamma^{+}$	0.1563
(18)NKcells	0.5625
(1) $CD11b^{+}CD11c^{+}$	0.6875
(2) $CD14^{+}HLA\text{-}DR^{+}$	>0.999
(3) $CD14^{+}IL\text{-}6^{+}$	0.4375
(20) $CD11c^{+}CD38^{+}$	0.5625

(13) $CD19^{+}CD38^{+}IL7R^{+}$	>0.999
(14) $CD19^{low}IgA^{+}$	0.8438
(7) $CD38^{+}HLA\text{-}DR^{+}IgA^{-}$	0.5625
(9)$CD38^{+}IgA^{+}CD11b^{+}$	0.8438
(10) $CD38^{+}HLA\text{-}DR^{+}IgA^{+}$	0.8438
(12)$CD38^{+}IgM^{+}$	0.8438
(8)$CD38^{+}IgA^{+}$	>0.999
(11)$CD38^{+}IgM^{-}$	0.3125
(15)ILC3	0.4375
(16)ILCs	0.8438
(17)NK $IFN\gamma^{+}$	0.0313
(18)NKcells	>0.999
(1) $CD11b^{+}CD11c^{+}$	0.1563
(2) $CD14^{+}HLA\text{-}DR^{+}$	0.1563
(3) $CD14^{+}IL\text{-}6^{+}$	>0.999
(20) $CD11c^{+}CD38^{+}$	0.5625

C. Statistical summary and p-values Figure 5D, (expression levels fold change normalized to samples treated only with P/I)

One-tailed Wilcoxon matched-pairs signed rank test. Tables indicate p-values.

UC + P/I + BTP2 vs UC + P/I										
Cluster_id	1	2	3	4	5	6	7	8	9	10
CD45RA	>0.999	0.0625	0.5625	0.6875	0.6875	0.3125	0.8438	0.5625	0.4375	0.8438
CD45RO	0.4375	>0.999	0.8438	0.4375	0.8438	0.3125	0.2188	0.4375	0.4375	0.3125
CCR7	0.3125	0.8438	0.4375	0.0313	0.5625	0.0313	>0.999	>0.999	0.625	0.8438
CD27	0.0938	0.5625	0.0625	0.8438	>0.999	0.1563	0.6875	0.8438	0.4375	0.3125
IL-7R	0.6875	0.6875	0.8438	0.5625	0.4375	0.4375	>0.999	0.8438	0.1875	>0.999
CD25	0.4375	0.5625	0.6875	0.2188	0.0313	0.0313	0.3125	>0.999	0.0625	>0.999
CD137	0.1563	0.6875	>0.999	0.8438	0.4375	0.8438	0.0625	0.1563	0.625	0.3125
CD86	0.3125	0.2188	0.3125	0.6875	0.4375	0.5625	>0.999	>0.999	0.0625	0.8438
CD40	0.3125	>0.999	0.625	0.5625	0.4375	0.6875	0.6875	>0.999	>0.999	0..0625
CD40L	0.3125	0.4375	0.4875	>0.999	>0.999	>0.999	0.8438	0.3125	0.625	0.2188
HLA-DR	0.8438	0.6875	>0.999	0.6875	0.1563	0.8438	>0.999	>0.999	>0.999	0.5625
Fas	0.8438	0.5625	0.4375	0.2188	0.8438	0.6875	0.5625	>0.999	>0.999	>0.999
FasL	0.3125	0.3125	0.5625	0.8438	0.8438	0.5625	0.6875	0.2188	0.0625	>0.999
PD-1	0.2188	0.6875	>0.999	0.6875	0.5625	>0.999	0.8438	0.6875	>0.999	0.4375
TNF	0.3125	0.2188	0.2188	0.0313	0.1563	0.0313	>0.999	0.3125	0.1875	0.5625
IFNγ	0.1563	0.8438	0.4375	>0.999	0.2188	0.1563	0.0625	>0.999	0.0625	>0.999
IL-2	>0.999	0.215	0.5625	0.3125	0.1563	0.4375	0.5625	0.4375	>0.999	0.6875
IL-4	0.8438	0.3125	0.3125	0.8438	0.8438	>0.999	0.4375	0.1563	0.1875	>0.999
IL-13	0.6875	0.3125	0.3125	0.5625	0.5625	0.8438	0.4375	0.5625	0.625	0.4375
IL-17A	0.8438	0.8438	0.6875	0.4375	0.2188	0.6875	0.2188	0.0625	0.4375	0.8438
IL-6	>0.999	0.3125	>0.999	0.0313	0.0313	0.0313	0.5625	>0.999	0.625	0.6875
IL-23p19	0.8438	0.8125	0.6875	0.5625	0.5625	0.4375	0.2188	0.4375	0.8125	0.3125
UC + P/I + BTP2 vs UC + P/I										
Cluster_id	11	12	13	14	15	16	17	18	19	20
CD45RA	0.8438	>0.999	0.625	0.4375	0.2188	0.6875	0.6875	0.6875	0.8438	0.8438
CD45RO	0.3125	0.8125	0.4375	0.1563	0.1563	0.4375	0.4375	0.0625	0.5625	0.4375
CCR7	>0.999	>0.999	0.625	0.6875	0.0313	0.4375	0.4375	>0.999	0.2188	0.6875
CD27	0.3125	0.625	0.4375	0.4375	0.0313	0.0938	0.8438	0.4375	0.0938	0.4375
IL-7R	0.4375	0.8125	0.8125	0.3125	>0.999	>0.999	0.8438	0.8438	0.4375	0.4375
CD25	0.6875	>0.999	>0.999	0.0625	0.0938	0.3125	0.8438	>0.999	0.4375	0.5625
CD137	0.8438	0.8125	0.3125	0.3125	0.0313	0.2188	>0.999	0.2188	0.8438	0.8125
CD86	0.6875	0.3125	0.625	0.6875	0.0625	0.8438	0.8438	0.8438	0.4375	>0.999
CD40	0.6875	0.8125	0.3125	0.5625	0.8438	0.6875	0.125	0.5625	0.5625	0.875
CD40L	0.5625	0.8125	0.8125	0.8125	0.4375	0.0313	0.5625	0.2188	0.0625	>0.999
HLA-DR	0.4375	0.4375	0.3125	0.2188	0.3125	0.0938	0.4375	0.1563	>0.999	0.6875
Fas	>0.999	0.8125	0.1875	>0.999	>0.999	0.0313	0.0625	>0.999	>0.999	0.5625
FasL	0.6875	0.8125	0.8125	0.0938	0.8438	0.0313	0.0313	0.8438	0.2188	0.4375
PD-1	0.8438	>0.999	0.625	0.6875	0.0625	0.8438	>0.999	0.2188	0.5625	>0.999
TNF	0.6875	>0.999	0.625	>0.999	0.6875	0.0313	0.3125	0.2188	0.5625	>0.999
IFNγ	0.5625	0.25	0.625	>0.999	0.6875	>0.999	0.4375	0.2188	0.8438	0.625

IL-2	0.8438	0.8125	0.625	0.0938	0.8438	0.0313	0.2188	0.0938	>0.999	0.8125
IL-4	0.8438	>0.999	0.3125	0.5625	>0.999	0.5625	0.2188	0.2188	0.3125	0.3125
IL-13	0.6875	>0.999	0.4375	0.2188	>0.999	0.3125	0.3125	0.6875	0.6875	0.0938
IL-17A	0.4375	0.1875	0.4375	0.6875	0.8438	0.0313	0.5625	0.3125	0.1563	0.625
IL-6	0.8438	0.4375	0.4375	0.8125	0.8438	0.1563	0.8438	0.8438	0.8438	0.8125
IL-23p19	0.4375	0.3125	0.3125	0.6875	0.2188	0.3125	>0.999	0.6875	0.2188	0.3125

CD + P/I + BTP2 vs CD + P/I										
Cluster_id	1	2	3	4	5	6	7	8	9	10
CD45RA	0.4375	0.0625	>0.999	0.0625	0.0313	0.25	0.156	0.8438	>0.999	0.0938
CD45RO	0.6875	>0.999	0.2188	0.5625	0.3125	0.25	0.156	0.6875	0.3125	0.5625
CCR7	0.6875	0.8438	0.4375	0.0313	0.0625	0.5	0.688	0.6875	0.8125	0.6875
CD27	0.0313	0.5625	0.75	0.3125	0.4375	0.5	0.688	>0.999	0.625	0.0625
IL-7R	>0.999	0.6875	0.6875	>0.999	0.4375	>0.999	0.313	0.2188	>0.999	0.8438
CD25	0.4375	0.5625	0.0625	0.0313	0.0313	>0.999	0.313	0.3125	>0.999	0.3125
CD137	>0.999	0.6875	0.625	0.5625	0.0313	0.75	0.156	0.5625	0.8125	0.1875
CD86	0.2188	0.2188	0.5625	0.4375	0.0313	0.25	0.156	0.1563	0.1875	0.1563
CD40	0.5625	>0.999	0.125	0.0313	0.0313	0.25	0.438	0.6875	0.125	>0.999
CD40L	0.4375	0.4375	0.5	0.3125	>0.999	0.5	>0.999	0.4375	0.0625	0.4375
HLA-DR	0.3125	0.6875	0.3125	0.3127	0.8438	0.25	0.563	>0.999	0.1875	0.5625
Fas	0.8438	0.5625	0.3125	0.2188	0.6875	0.5	0.563	0.8438	0.625	0.6875
FasL	0.5625	0.3125	0.8125	0.5625	0.4375	0.25	0.219	0.8438	0.0625	0.8438
PD-1	0.3125	0.6875	0.2188	>0.999	0.3125	0.25	0.438	0.5625	>0.999	0.6875
TNF	0.5625	0.2188	0.4375	0.0313	0.0313	0.25	0.688	>0.999	0.3125	0.1563
IFNγ	0.0313	0.0313	0.375	>0.999	>0.999	0.75	0.031	0.0625	0.625	0.8125
IL-2	0.0313	0.125	0.8432	0.0313	0.0313	0.25	0.813	0.8438	0.125	0.6875
IL-4	0.8438	0.3125	>0.999	0.4375	0.2188	0.25	0.031	0.8438	0.8125	0.0625
IL-13	0.8438	0.8438	>0.999	>0.999	0.8438	0.75	0.844	>0.999	0.3125	0.625
IL-17A	>0.999	0.8438	0.4375	0.3127	>0.999	0.25	0.844	0.3125	0.625	0.8438
IL-6	0.5625	0.3125	>0.999	0.0313	0.0313	0.25	0.844	>0.999	0.0625	0.4375
IL-23p19	0.6875	0.8125	0.625	0.6875	0.1563	0.25	>0.999	0.6875	0.8125	>0.999

CD + P/I + BTP2 vs CD + P/I										
Cluster_id	11	12	13	14	15	16	17	18	19	20
CD45RA	0.6875	0.6875	0.6875	0.0313	0.0625	>0.999	0.8438	0.5625	0.6875	>0.999
CD45RO	0.4375	0.6875	0.4375	0.5625	0.8438	0.1563	>0.999	0.4375	0.0313	0.0313
CCR7	0.3125	0.2188	0.8438	0.2188	0.4375	0.0625	0.5625	0.0625	0.8438	0.6875
CD27	0.8438	0.8438	0.0625	0.1563	0.4375	0.8438	0.4375	0.3125	>0.999	0.2188
IL-7R	0.5625	0.0313	0.6875	0.0625	0.4375	0.6875	0.4375	>0.999	0.8438	0.5625
CD25	0.3125	0.4375	0.3125	0.4375	0.5625	0.0313	0.3125	0.0625	0.5625	0.6875
CD137	0.3175	0.2188	0.4375	>0.999	>0.999	0.8438	0.8438	0.1563	0.0938	>0.999
CD86	>0.999	0.5625	>0.999	0.6875	>0.999	0.2188	0.3125	0.4375	0.0313	>0.999
CD40	>0.999	0.0938	0.5625	0.0938	0.6875	0.2188	0.4375	>0.999	0.0313	0.4375
CD40L	0.2188	0.3125	0.6875	0.8438	0.21188	0.0313	0.2188	0.1563	0.5625	>0.999
HLA-DR	0.6875	0.4375	0.3125	0.8438	0.2188	0.3125	0.5625	0.5625	>0.999	0.1563
Fas	0.4375	0.5625	0.3125	0.8438	0.5625	0.8438	0.8438	0.0313	0.8438	>0.999
FasL	0.6875	0.8438	0.4375	0.6875	>0.999	0.4375	0.4375	>0.999	0.0313	0.3125
PD-1	0.8438	0.5625	0.4375	0.4375	0.5625	0.8438	0.2188	0.6875	0.8438	>0.999
TNF	0.8438	0.0938	0.4375	>0.999	0.0313	0.2188	0.0938	0.0313	0.0313	0.6875
IFNγ	0.4375	0.8438	0.4375	0.5625	0.4375	0.1563	0.0313	0.0313	0.0313	0.0625
IL-2	0.3125	0.8438	0.2188	0.8438	0.8438	0.0313	0.0313	0.0313	0.2188	>0.999
IL-4	0.8438	0.3125	0.4375	0.8438	0.5625	>0.999	0.5625	0.2188	0.4375	0.5625
IL-13	0.4375	0.4375	>0.999	0.2188	0.4375	0.2188	>0.999	>0.999	>0.999	>0.999
IL-17A	0.4375	0.8438	0.8125	>0.999	0.0938	0.1563	>0.999	>0.999	0.0313	>0.999
IL-6	>0.999	0.8438	0.6875	0.5625	0.1563	0.0313	>0.999	0.5625	0.0313	0.6875
IL-23p19	0.5625	0.2188	0.4375	0.0938	0.5625	0.8438	0.4375	0.4375	0.6875	0.2188

Appendix Table S13. (**A-E**) Statistical summary and p-values of Figure 6.

A. Statistical summary and p-values Figure 6A,

(Resazurin assay murine organoids)

Multiple t-tests with Holm-Sidak Correction

Comparison	Day of Culture	adj p-value
Ctrl vs BTP2	1	0.6932
Ctrl vs BTP2	2	0.6932
Ctrl vs BTP2	3	0.6932
Ctrl vs BTP2	4	0.6932
Ctrl vs BTP2	5	0.4119

B. Statistical summary and p-values Figure 6C,

(TEER measurements murine organoids)

Multiple t-tests with Holm-Sidak Correction

Comparison	Days in Culture	adj p-value
Ctrl vs BTP2	-2	0.9979
Ctrl vs BTP2	0	0.9979
Ctrl vs BTP2	2	0.9979
Ctrl vs BTP2	3	0.9979
Ctrl vs BTP2	4	0.9829
Ctrl vs BTP2	5	0.8192
Ctrl vs BTP2	6	0.9934

C. Statistical summary and p-values Figure 6D,

(Resazurin assay human organoids)

Multiple t-tests with Holm-Sidak Correction

Comparison	Days in Culture	adj p-value
Ctrl vs BTP2	2	0.7451
Ctrl vs BTP2	4	0.7558
Ctrl vs BTP2	6	0.7558

D. Statistical summary and p-values Figure 6E,

(TEER measurements human organoid monolayers)

Multiple t-tests with Holm-Sidak Correction

Comparison	Days in Culture	adj p-value
Ctrl vs BTP2	-2	0.9998
Ctrl vs BTP2	0	0.9998
Ctrl vs BTP2	2	0.9998
Ctrl vs BTP2	3	0.9998
Ctrl vs BTP2	4	0.9998
Ctrl vs BTP2	5	0.9998
Ctrl vs BTP2	6	0.9998

E. Statistical summary and p-values Figure 6F,

(Relative mRNA expression)

Multiple t-tests with Holm-Sidak Correction

Comparison	Gene	adj p-value
Ctrl vs BTP2	ALPI	0.9600
Ctrl vs BTP2	CHGA	0.9600
Ctrl vs BTP2	MUC2	0.9488

Appendix Table S14. (**A-F**) Statistical summary and p-value of Figure 7.

A. Statistical summary and p-values Figure 7E,

(Weight curve of the mice treated with vehicle or CM4620)
Unpaired T test, equal variance

	Individual p-value
day 48 Vehicle vs days 48 CM4620	0.048300314

B. Statistical summary and p-values Figure 7F,

(CM4620 levels in serum ng/mL.)
Unpaired T test, equal variance

	Individual p-value
Vehicle vs CM4620 serum CRACi level	3.55827E-10

C. Statistical summary and p-values Figure 7G,

(Histological score of colitis)
Mann Whitney test

	Individual p-value
vehicle vs CM4620	0.0005

D. Statistical summary and p-values Figure 7H,

(Cellularity of Neutrophil or CD4 T cells)
Equal variance unpaired T.test

Normalized CD11b+Gr-1+ Neutrophil#	Individual p-value
vehicle vs CM4620 with pooled Exp1 and Exp2	0.039206576

Normalized CD4+ T cell#	Individual p-value
vehicle vs CM4620 with pooled Exp1 and Exp2	0.517624931

E. Statistical summary and p-values Figure 7I,

(Quantification of Foxp3+ and Rorgt+ cells)
Equal variance unpaired T.test

Foxp3% in CD4 T cell	Individual p-value
vehicle vs CM4620	0.149701766

Rorg% in CD4 T cell	Individual p-value
vehicle vs CM4620	0.915087936

F. Statistical summary and p-values Figure 7J,

(Cytokine production by CLP derived CD4 T cells)
Equal variance unpaired T.test

IFNg%	Individual p-value
vehicle vs CM4620	0.001464702

TNF%	Individual p-value
vehicle vs CM4620	0.000769115

IL2%	Individual p-value
vehicle vs CM4620	0.000476664

Appendix Table S15. Statistical summary and p-values of Expanded View Figure 4.

Statistical summary and p-values Figure EV4B,

(Calcium influx rate in PBMCs)

RM one-way ANOVA, uncorrected Fisher's LSD

CD4 T cells (PBMC)	Individual p-value
untreated vs. BTP2	<0.0001
untreated vs. CM4620	<0.0001

CD8 T cells (PBMC)	Individual p-value
untreated vs. BTP2	0.0007
untreated vs. CM4620	0.0002

CD19 B cells (PBMC)	Individual p-value
untreated vs. BTP2	<0.0001
untreated vs. CM4620	<0.0001

$CD56^{+}$ NK cells (PBMC)	Individual p-value
untreated vs. BTP2	0.0048
untreated vs. CM4620	0.0002

$CD14^{+}$ Myeloid cells (PBMC)	Individual p-value
untreated vs. BTP2	0.0016
untreated vs. CM4620	0.0003

Appendix Table 16. (**A-C**) Statistical summary and p-values of Expanded View Figure 5.

A. Statistical summary and p-values Figure EV5B,

(Calcium influx rate in LPMCs)

RM one-way ANOVA, uncorrected Fisher's LSD

CD4 T cells (LPMC)	Individual p-value
untreated vs. 1000 BTP2	0.1073
untreated vs. 1000 CM4620	0.037
untreated vs. 250 BTP2	0.0024
untreated vs. 250 CM4620	0.0549

CD8 T cells (LPMC)	Individual p-value
untreated vs. 1000 BTP2	0.002
untreated vs. 1000 CM4620	0.0002
untreated vs. 250 BTP2	0.0222
untreated vs. 250 CM4620	0.0022

B. Statistical summary and p-values Figure EV5E,

(Expression levels fold change normalized to Unstimulated samples)

One-tailed Wilcoxon matched-pairs signed rank test comparing expression fold change of P/I/unstim. vs Unstim/unstim

The table indicates individual p-values.

P/I/unstim. vs Unstim/unstim	CD4+ CD45RO+	CD4+ IL7R+	CD4+ IL-17A+	CD8+ CD45RO+	CD8+ IL-7R+	Tregs+	CD19+ HLA-DR+	CD11c+
CD25	0.0313	0.0156	0.0469	0.0156	0.0469	0.0156	0.2813	0.0156
CCR7	0.1094	0.0156	0.0156	0.0156	0.0156	0.0313	0.0156	0.1094
CD27	0.0313	0.0313	0.2188	0.1094	0.2188	0.0781	0.0156	0.0156
CD40L	0.0156	0.0156	0.0156	0.0156	0.0156	0.0156	0.0156	0.5
CD137	0.0156	0.0156	0.0156	0.0156	0.0156	0.0156	0.0156	0.5
FasL	0.0156	0.0156	0.0313	0.0156	0.0313	0.0156	0.0469	0.0313
PD-1	0.0789	0.0156	0.0313	0.0156	0.0313	0.0156	0.0469	0.5
IL-7R	0.2188	0.0156	0.1563	0.0156	0.1563	0.0156	0.0156	0.0156
IL-2	0.0156	0.0156	0.0156	0.0156	0.0156	0.0156	0.1563	0.1563
TNF	0.0156	0.0156	0.0156	0.0156	0.0156	0.0156	0.4219	0.3438
IFNγ	0.0156	0.0156	0.0156	0.0156	0.0156	0.0156	0.0156	0.0156
IL-13	0.0156	0.0156	0.0313	0.0156	0.0156	0.0156	0.5	0.0156
IL-4	0.0156	0.0156	0.0156	0.0156	0.0313	0.2813	0.0313	0.1563
IL-17A	0.0156	0.0156	0.0156	0.0156	0.0156	0.0156	0.0781	0.0156

C. Statistical summary and p-values Figure EV5F,

(Expression levels fold change normalized to PMA/ionomycin)

One-tailed Wilcoxon matched-pairs signed rank test. Tables indicate individual p-values.

CD4+ CD45RO+	250nM BTP2 vs. 250nM CM4620	1μM BTP2 vs. 1μM CM4620
CD25	0.0156	0.1563
CCR7	0.0313	0.5
CD27	0.4219	0.3438
CD40L	0.0156	0.0313
CD137	0.2813	0.2813
FasL	0.0156	0.0781
PD-1	0.4219	0.4219
IL-7R	0.0156	0.4219
IL-2	0.0156	0.0156
TNF	0.0156	0.0156
IFNγ	0.0156	0.0156

CD4+ IL-7R+	250nM BTP2 vs. 250nM CM4620	1μM BTP2 vs. 1μM CM4620
CD25	0.0156	0.1094
CCR7	0.0313	0.4219
CD27	0.4219	0.2813
CD40L	0.0156	0.0313
CD137	0.0781	0.0781
FasL	0.5	0.0781
PD-1	0.4219	0.4219
IL-7R	0.0313	0.0156
IL-2	0.0156	0.0156
TNF	0.0156	0.0156
IFNγ	0.0156	0.0156

CD4+ IL-17A+	250nM BTP2 vs. 250nM CM4620	1μM BTP2 vs. 1μM CM4620
CD25	0.0313	0.5
CCR7	0.0156	0.3438
CD27	0.1563	0.2188
CD40L	0.0156	0.1563
CD137	0.4219	0.4219
FasL	0.0156	0.1094
PD-1	0.1094	0.3438
IL-7R	0.4219	0.5
IL-2	0.0156	0.1094
TNF	0.0156	0.0156
IFNγ	0.0156	0.0156

IL-13	0.0156	0.1563
IL-4	0.0156	0.781
IL-17A	0.0156	0.0156

IL-13	0.0313	0.0156
IL-4	0.0469	0.0469
IL-17A	0.0156	0.0156

IL-13	0.2813	0.5
IL-4	0.1563	0.2813
IL-17A	0.0156	0.0156

CD8+ CD45RO+	250nM BTP2 vs. 250nM CM4620	1µM BTP2 vs. 1µM CM4620
CD25	0.0156	0.5
CCR7	0.0156	0.0781
CD27	0.4219	0.3438
CD40L	0.0313	0.1563
CD137	0.0156	0.0313
FasL	0.1563	0.0469
PD-1	0.3438	0.1094
IL-7R	0.0156	0.2188
IL-2	0.0156	0.0156
TNF	0.0156	0.0156
IFNγ	0.0156	0.0156
IL-13	0.0313	0.2813
IL-4	0.0156	0.0313
IL-17A	0.1563	0.2188

CD8+ IL-7R+	250nM BTP2 vs. 250nM CM4620	1µM BTP2 vs. 1µM CM4620
CD25	0.0313	0.3438
CCR7	0.0156	0.2813
CD27	0.0469	0.0313
CD40L	0.0156	0.0469
CD137	0.0313	0.0156
FasL	0.4219	0.0156
PD-1	0.0469	0.0156
IL-7R	0.1563	0.4219
IL-2	0.0156	0.0469
TNF	0.0156	0.0313
IFNγ	0.0156	0.0156
IL-13	0.1094	0.4219
IL-4	0.0313	0.0156
IL-17A	0.3438	0.2813

Tregs	250nM BTP2 vs. 250nM CM4620	1µM BTP2 vs. 1µM CM4620
CD25	0.2813	0.3438
CCR7	0.0313	0.3438
CD27	0.5	0.2188
CD40L	0.0156	0.0313
CD137	0.2813	0.0156
FasL	0.0781	0.4219
PD-1	0.5	0.0789
IL-7R	0.0781	0.0156
IL-2	0.0156	0.0313
TNF	0.0156	0.0313
IFNγ	0.0156	0.0313
IL-13	0.0156	0.0469
IL-4	0.5	0.0313
IL-17A	0.0156	0.0156

CD19+ HL-DR+	250nM BTP2 vs. 250nM CM4620	1µM BTP2 vs. 1µM CM4620
CD25	0.2188	0.5
Fas	0.1563	0.2188
CCR7	0.0781	0.3438
CD27	0.4219	0.5
CD40	0.0781	0.4219
CD38	0.1563	0.1094
HLA-DR	0.0156	0.1094
IL-7R	0.1094	0.2813
IgA	0.1563	0.4219
IgM	0.5	0.1094
TNF	0.2813	0.0469
IFNγ	0.5	0.0781
IL-6	0.1094	0.0781
IL-23p19	0.2188	0.2188

CD11c+	250nM BTP2 vs. 250nM CM4620	1µM BTP2 vs. 1µM CM4620
CD25	0.0781	0.1094
Fas	0.5	0.1563
CCR7	0.1094	0.5
CD27	0.3438	0.5
CD40	0.0781	0.5
CD38	0.1094	0.2813
HLA-DR	0.3438	0.4219
IL-7R	0.2188	0.2813
IgA	0.2188	0.5
IgM	0.4219	0.0781
TNF	0.0469	0.1094
IFNγ	0.0313	0.0156
IL-6	0.2813	0.3438
IL-23p19	0.1563	0.2813

Appendix Tables S17-S22. Statistical Summary and p-values of Appendix.

S17. Statistical summary and p-values Appendix Figure S1

(Expression levels of Stim1, Stim2, Orai1, Orai2 and Orai3 in T helper cell subsets)
Two-way ANOVA test

Tukey's multiple comparisons test	Summary	Adjusted p-value
Stim1		
Th1 vs. Th17	ns	0.915
Th1 vs. iTreg	ns	0.6043
Th17 vs. iTreg	ns	0.8407
Stim2		
Th1 vs. Th17	ns	0.9964
Th1 vs. iTreg	ns	0.915
Th17 vs. iTreg	ns	0.8801
Orai1		
Th1 vs. Th17	ns	0.3302
Th1 vs. iTreg	ns	0.0928
Th17 vs. iTreg	ns	0.7515
Orai2		
Th1 vs. Th17	ns	>0.9999
Th1 vs. iTreg	ns	0.8407
Th17 vs. iTreg	ns	0.8407
Orai3		
Th1 vs. Th17	ns	0.9447
Th1 vs. iTreg	ns	0.9685
Th17 vs. iTreg	ns	0.9964

S18. Statistical summary and p-values Appendix Figure S2 (A-B)

A. Appendix Fig. S2A, (calcium influx rate in PBMCs)

Paired t test with FDR adjustment at 1% using Benjamini, Krieger and Yekutieli procedure

PBMCs	p-value
$CD4^+$ T cells cnt. vs $CD4^+$ T cells + BTP2	0.009841
$CD8^+$ T cells vs $CD8^+$ T cells + BTP2	0.006798
$CD19^+$ B cells vs $CD19^+$ B cells + BTP2	0.00001
monocytes vs monocytes + BTP2	0.012634
NK cells vs NK cells + BTP2	0.035858

B. Appendix Fig. S2B, (apoptosis assay)

Wilcoxon matched-pairs signed rank test

LPMCs	p-value
Unstim vs PMA/Iono	>0.999
PMA/Iono vs PMA/Iono + BTP2	>0.999

S19. Statistical summary and p-values Appendix Figure S4 (A-C)

A. Appendix Fig. S4B, (peak quantification). Unpaired T test, equal variance

Peak Fura-2 ratio (800-1100s)	p-value
Mock vs 100	0.26160
Mock vs 250	0.01114
Mock vs 500	0.00047
Mock vs 1000	0.00001

B. Appendix Fig. S4D,

(Cytokines MFI ± BTP2 normalize to mean of mock condition)
Unpaired T test, equal variance

Cytokines MFI ± BTP2		p-value
Mock vs 20	Th1(IFNg) vs Treg(CTLA4)	1.0000
Mock vs 50	Th1(IFNg) vs Treg(CTLA4)	0.0044
Mock vs 100	Th1(IFNg) vs Treg(CTLA4)	0.0044
Mock vs 250	Th1(IFNg) vs Treg(CTLA4)	0.0006
Mock vs 500	Th1(IFNg) vs Treg(CTLA4)	0.0062

Cytokines MFI ± BTP2		p-value
Mock vs 20	Th17(IL17A) vs Treg(CTLA4)	1

Mock vs 50	Th17(IL17A) vs Treg(CTLA4)	0.0003964
Mock vs 100	Th17(IL17A) vs Treg(CTLA4)	0.00062301
Mock vs 250	Th17(IL17A) vs Treg(CTLA4)	6.9354E-06
Mock vs 500	Th17(IL17A) vs Treg(CTLA4)	0.00209937

C. Appendix Fig. S4F,

(Markers MFI ± BTP2 normalize to mean of mock condition)
Unpaired T test, equal variance

Markers MFI ± BTP2		p-value
Mock vs 20	Th1 vs Treg	0.747
Mock vs 50	Th1 vs Treg	0.000
Mock vs 100	Th1 vs Treg	0.000
Mock vs 250	Th1 vs Treg	0.001
Mock vs 500	Th1 vs Treg	0.006

Markers MFI ± BTP2		p-value
Mock vs 20	Th17 vs Treg	0.04483731
Mock vs 50	Th17 vs Treg	0.00541864
Mock vs 100	Th17 vs Treg	0.17023393
Mock vs 250	Th17 vs Treg	0.17158405
Mock vs 500	Th17 vs Treg	0.01948784

S20. Statistical summary and p-values Appendix Figure S9

(Expression levels of TNF in T cell clusters)
Wilcoxon matched-pairs signed rank test

TNF expression	p-value
cluster 7 vs 1 in UC + P/I	0.0156
cluster 7 vs 3 in UC + P/I	0.0156
cluster 7 vs 10 in UC + P/I	0.0156
cluster 7 vs 5 in UC + P/I	0.0156
cluster 7 vs 6 in UC + P/I	0.0156
cluster 7 vs 15 in UC + P/I	0.1094
cluster 7 vs 12 in UC + P/I	0.5
cluster 7 vs 16 in UC + P/I	0.0156
cluster 7 vs 19 in UC + P/I	0.2188

TNF expression	p-value
cluster 7 vs 1 in CD + P/I	0.0156
cluster 7 vs 3 in CD + P/I	0.0313
cluster 7 vs 10 in CD + P/I	0.0156
cluster 7 vs 5 in CD + P/I	0.0156
cluster 7 vs 6 in CD + P/I	0.0156
cluster 7 vs 15 in CD + P/I	0.1563
cluster 7 vs 12 in CD + P/I	0.0469
cluster 7 vs 16 in CD + P/I	0.0156
cluster 7 vs 19 in CD + P/I	0.0156

S21. Statistical summary and p-values Appendix Figure S10 (A-B)

A. Appendix Fig. S10A, (IFNγ and IL-17A producing cell % in CD4)

Unpaired T test, equal variance

IFNg+ IL-17A+ in CD4 T cells %	p-value
Compare vehicle vs CM4620	0.180

B. Appendix Fig. S10B, (cytokines /gram colon tissue)

Unpaired T test, equal variance

IL-2 Level / gram colon tissue	p-value
Compare vehicle vs CM4620	0.043

IL-10 Level / gram colon tissue	p-value
Compare vehicle vs CM4620	0.926

S22. Statistical summary and p-values Appendix Figure S11

(Calcium Influx Rate in $CD4^+$ and $CD8^+$ LPMCs)
Unpaired T test, equal variance

CD4+ LPMCs	p-value
IBD vs Inflamed	0.0124

CD8+ LPMCs	p-value
IBD vs Inflamed	0.0667
