# Supplementary material for: Store‐operated calcium entry controls innate and adaptive immune cell function in inflammatory bowel disease
Source: EMBO Mol Med. 2022 Aug 2;14(9):e15687. doi: 10.15252/emmm.202215687 (PMC9449601; doi:10.15252/emmm.202215687)
Supplement: Supplementary file 2 — Expanded View Figures PDF [file EMMM-14-e15687-s010.pdf]

Expanded View Figures

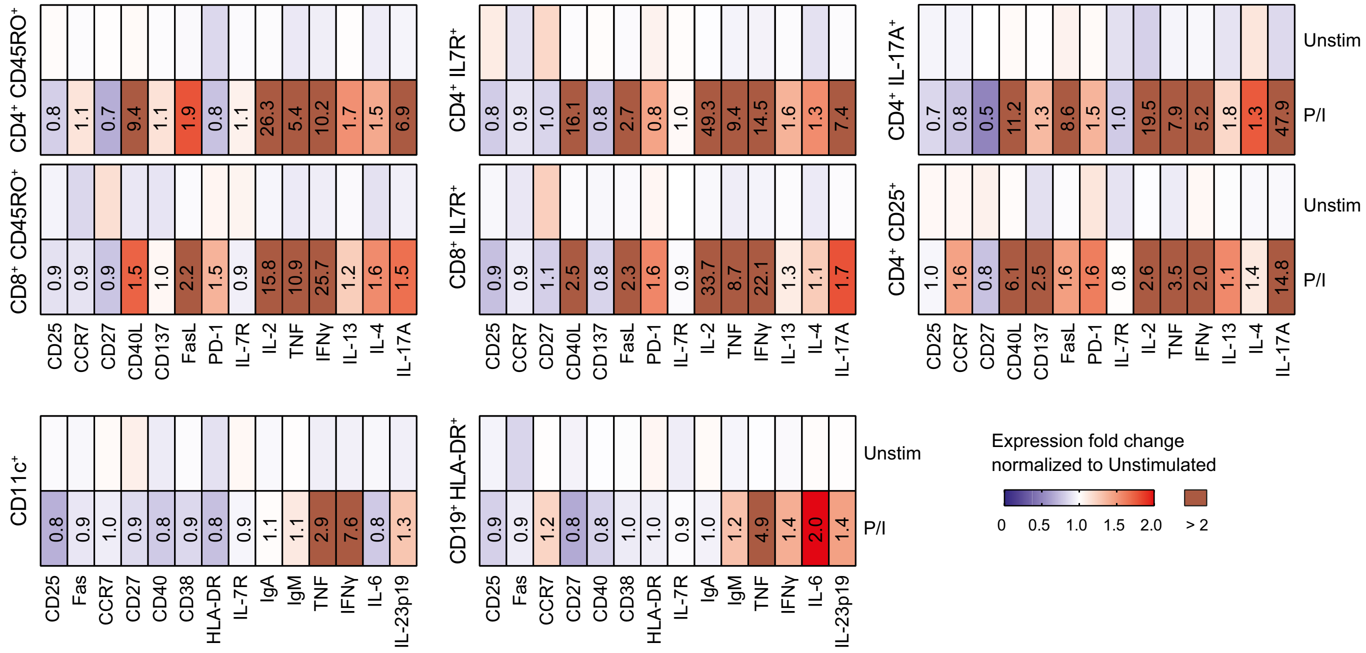

Figure EV1. Effects of PMA/ionomycin stimulation on protein expression of human lamina propria (LP) immune cells.

Heatmaps representing the median fold change (FC) of cytokine and surface marker expression in $CD45^{+}CD3^{+}$ T cells (top row) and $CD45^{+}CD3^{-}$ immune cells (bottom row) isolated from colon lamina propria of five patients with Crohn's disease. Cells were treated with 20 ng/ml PMA and 1 μg/m (P/I) or DMSO (unstimulated) for 4 h *in vitro*, and protein expression was measured by mass cytometry (CyTOF). FC values are relative to unstimulated samples.

Source data are available online for this figure.

© 2022 The Authors

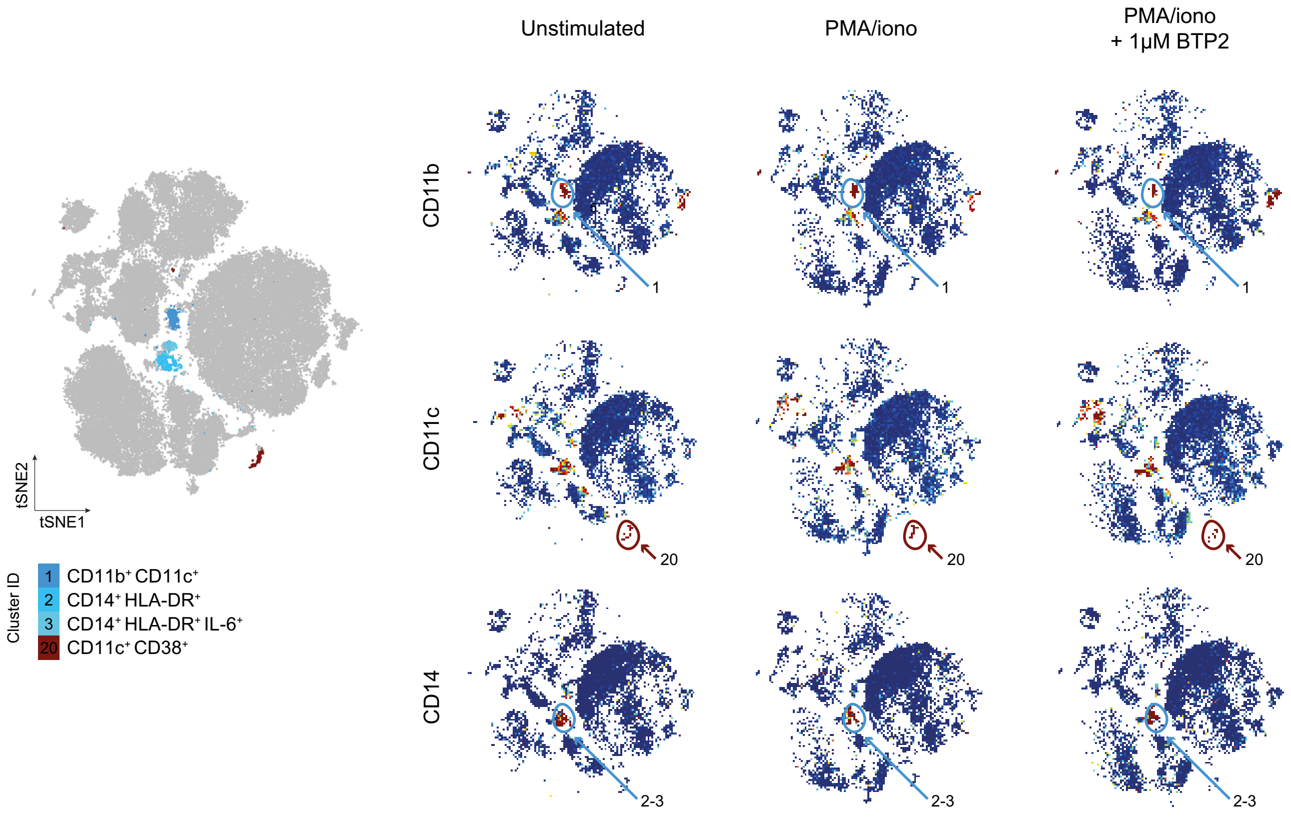

Figure EV2. Four clusters of myeloid cells in the colon lamina propria (CLP) of IBD patients and effects of SOCE inhibition.

(Left) FlowSOM plot of merged FCS files from unstimulated IBD samples and samples treated with PMA/ionomycin ± 1 μM BTP2 (non-inflamed: n = 4, CD: n = 6, UC: n = 6). Colors indicate myeloid cell clusters within $CD45^{+}CD3^{-}$ LPMCs. (Right) viSNE plots of one exemplary CD patient. Colors indicate expression levels of cell surface markers (blue: low; red: high) in cells left unstimulated, stimulated with PMA/ionomycin or stimulated in the presence of 1 μM BTP2.

© 2022 The Authors

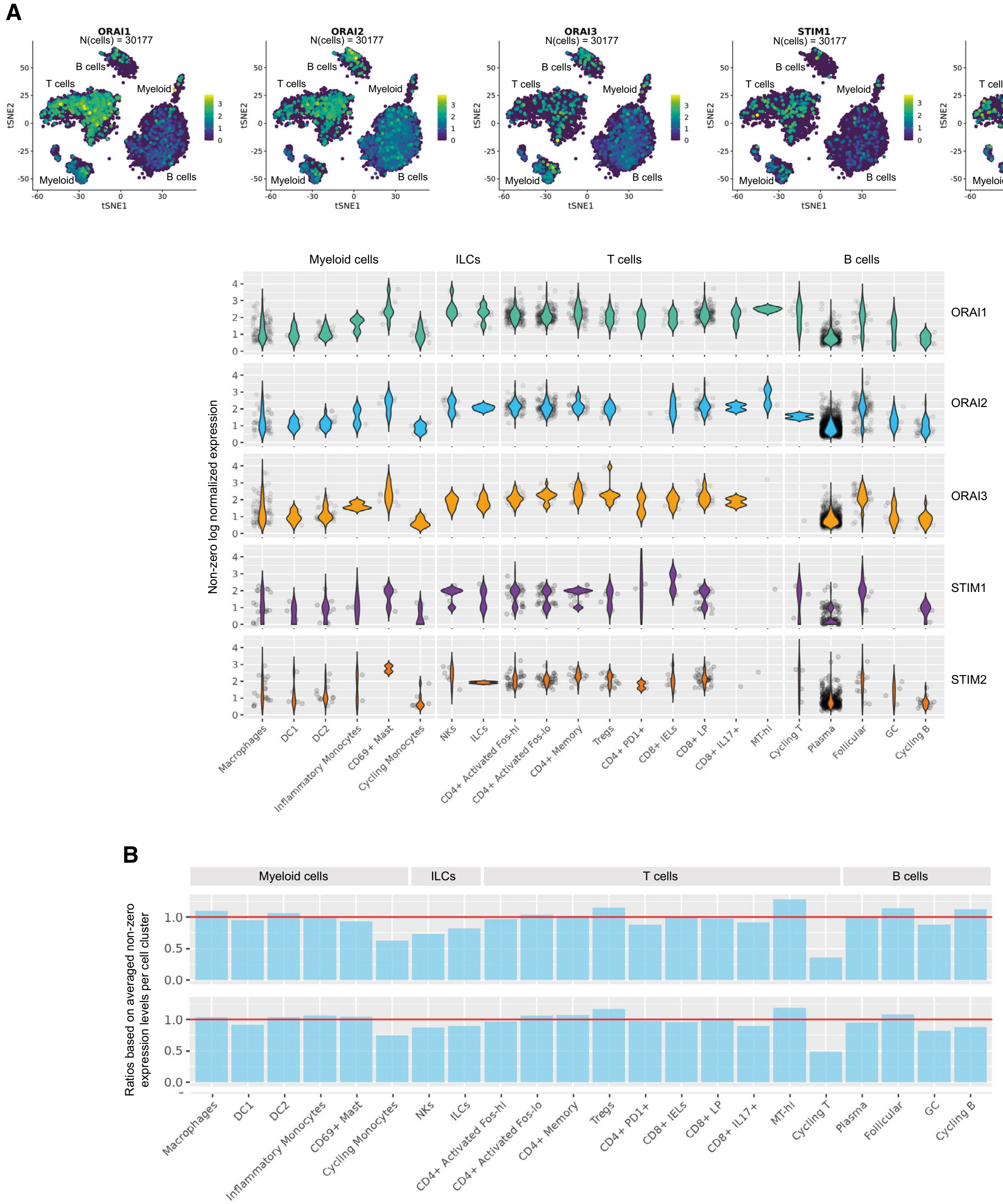

Figure EV3. mRNA expression of ORAI and STIM genes in lamina propria mononuclear cells (LPMC) of healthy donors.

A viSNE plots and violin plots showing mRNA expression levels of ORAI1, ORAI2, ORAI3, STIM1 and STIM2 in subsets of LPMC. Expression data were reanalyzed from Smillie *et al* (2019).

B Bar graphs showing the ratios of mRNA expression for ORAI3/ORAI1 and ORAI3/ORAI2 in different subsets of LPMC. Expression data were reanalyzed from Smillie *et al* (2019).

© 2022 The Authors

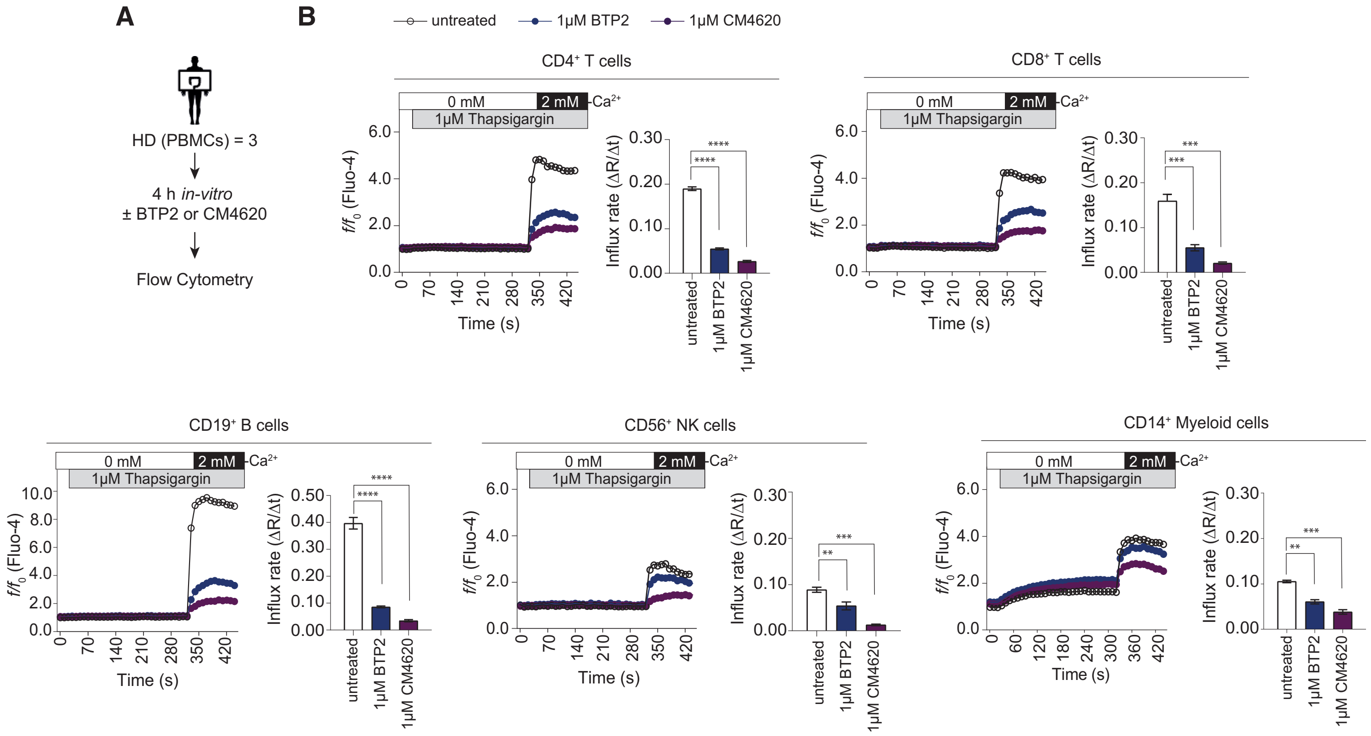

Figure EV4. Similar effects of CRAC channel inhibition with CM4620 and BTP2 on Ca^{2+} influx in human immune cell subsets.

A Experimental design for Ca^{2+} influx measurements in PBMCs of three healthy donors (HD) by flow cytometry.

B Ca^{2+} influx rates were measured in $CD4^+$ or $CD8^+$ T cells, $CD19^+$ B cells, $CD56^+$ NK cells and $CD14^+$ myeloid cells following pre-incubation with 1 µM BTP2 or 1 µM CM4620 for 4 h. Cells were stimulated with thapsigargin in Ca^{2+} free buffer followed by addition of 2 mM Ca^{2+} Ringer solution. Bar graphs show the mean values of influx rates after addition of 2 mM Ca^{2+} from one experiment (samples of n = 3 HD run in technical triplicates). Statistical significance was calculated using repeated measures (RM) one-way ANOVA test. ****$P < 0.0001$; ***$P < 0.001$; **$P < 0.01$; *$P < 0.05$.

Source data are available online for this figure.

Figure EV5. Suppression of cytokine production in LPMCs of IBD patients by CM4620.

A Experimental setup for Ca^{2+} influx measurements of LPMCs from three IBD patients by flow cytometry.

B Human lamina propria $CD4^+$ and $CD8^+$ T cells were pretreated with 250 nM or 1,000 nM BTP2 or CM4620 for 4 h *in vitro* and added to the cell suspension until acquisition. Cells were stimulated with 1 µM thapsigargin (TG) in Ca^{2+} free Ringer solution followed by the readdition of 2 mM Ca^{2+} Ringer solution. Bar graphs show the mean ± SEM of Ca^{2+} influx rates after the addition of 2 mM Ca^{2+} Ringer solution from one experiment (n = 3 IBD patients). Statistical significance was calculated with repeated measures (RM) one-way ANOVA test. ****$P < 0.0001$; ***$P < 0.001$; **$P < 0.01$; *$P < 0.05$.

C–F Mass cytometry analysis of LPMCs from six IBD patients. (C) Experimental design. (D) viSNE plots of $CD45^+CD3^+$ LPMCs stimulated with PMA/ionomycin in the presence of various (v) concentrations (250 or 1,000 nM) of BTP2 or CM4620 with unstimulated samples serving as controls. Colors indicate the expression level of IL-2, IL-17, IFNγ, and TNFα (blue: low, red: high) and are representative of one CD patient. (E) Heatmaps representing the median fold change of cytokines and cell surface molecules on $CD45^+CD3^+$ or $CD45^+CD3^-$ LPMCs that were stimulated with PMA/ionomycin for 4 h *in vitro*. Data are normalized to unstimulated samples. (F) Heatmaps representing the median fold change of cytokine and cell surface marker expression in $CD45^+CD3^+$ and $CD45^+CD3^-$ LPMCs stimulated with PMA/ionomycin for 4 h in the presence or absence of 250 nM or 1,000 nM BTP2 or 250 nM or 1,000 nM CM4620. Data were normalized to samples treated with PMA/ionomycin alone. Statistical significance in (E, F) was calculated using a one-tailed paired Wilcoxon matched-pairs signed rank test. *$P < 0.05$.

Source data are available online for this figure.

© 2022 The Authors

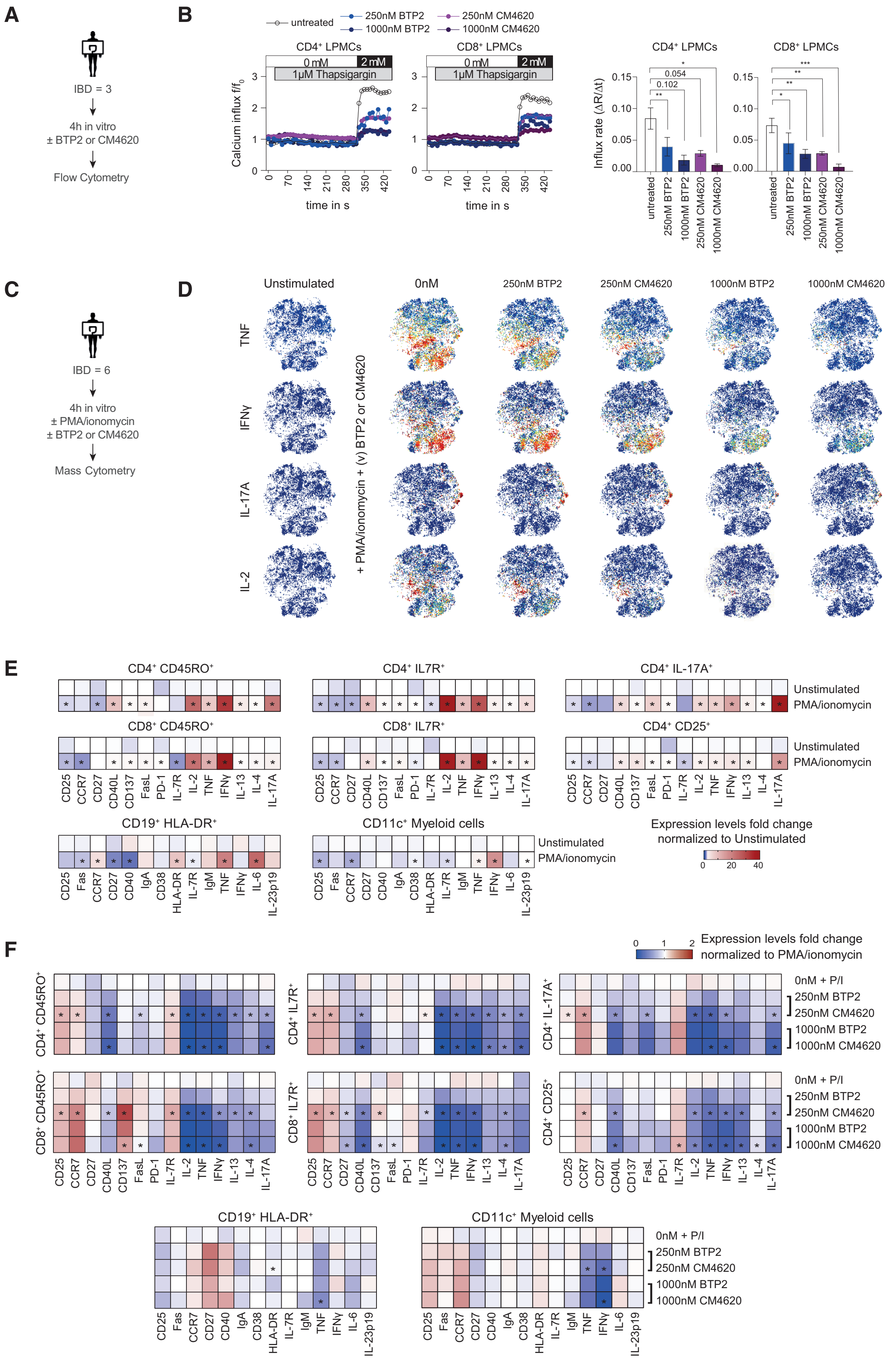

Figure EV5.

 © 2022 The Authors
