# Supplementary material for: Store‐operated calcium entry controls innate and adaptive immune cell function in inflammatory bowel disease
Source: EMBO Mol Med. 2022 Aug 2;14(9):e15687. doi: 10.15252/emmm.202215687 (PMC9449601; doi:10.15252/emmm.202215687)
Supplement: Supplementary file 11 [file EMMM-14-e15687-s006.pdf]

EMBO Molecular Medicine

Store-Operated Calcium Entry (SOCE) Controls Innate and Adaptive Immune Cell Function in IBD

Marilena Letizia, Yin-Hu Wang, Ulrike Kaufmann, Lorenz Gerbeth, Annegret Sand, Max Brunkhorst, Patrick Weidner, Jörn Ziegler, Chotima Böttcher, Stephan Schlickeiser, Camilla Zapata, Megumi Yamashita, Kenneth Stauderman, Katherine Sun, Desirée Kunkel, Murali Prakriya, Ashley Sanders, Britta Siegmund, Stefan Feske, and Carl Weidinger
DOI: 10.15252/emmm.202215687

Corresponding authors: Carl Weidinger (carl.weidinger@charite.de) , Stefan Feske (Stefan.Feske@nyulangone.org)

Review Timeline:

Submission Date:	7th Jan 22
Editorial Decision:	28th Jan 22
Revision Received:	16th May 22
Editorial Decision:	20th Jun 22
Revision Received:	3rd Jul 22
Accepted:	5th Jul 22

Editor: Lise Roth

Transaction Report:

(Note: With the exception of the correction of typographical or spelling errors that could be a source of ambiguity, letters and reports are not edited. Depending on transfer agreements, referee reports obtained elsewhere may or may not be included in this compilation. Referee reports are anonymous unless the Referee chooses to sign their reports.)

28th Jan 2022

Dear Dr. Weidinger,

Thank you for the submission of your manuscript to EMBO Molecular Medicine. We have now received feedback from the three reviewers who agreed to evaluate your manuscript. As you will see from the reports below, the referees acknowledge the interest of the study and are overall supporting publication of your work pending appropriate revisions.

Addressing the reviewers' concerns in full will be necessary for further considering the manuscript in our journal, and acceptance of the manuscript will entail a second round of review. EMBO Molecular Medicine encourages a single round of revision only and therefore, acceptance or rejection of the manuscript will depend on the completeness of your responses included in the next, final version of the manuscript. For this reason, and to save you from any frustrations in the end, I would strongly advise against returning an incomplete revision.

Revised manuscripts should be submitted within three months of a request for revision; they will otherwise be treated as new submissions, except under exceptional circumstances in which a short extension is obtained from the editor.

When submitting your revised manuscript, please carefully review the instructions that follow below. We perform an initial quality control of all revised manuscripts before re-review; failure to include requested items will delay the evaluation of your revision.

We require:

1) A .docx formatted version of the manuscript text (including legends for main figures, EV figures and tables). Please make sure that the changes are highlighted to be clearly visible.

2) Individual production quality figure files as .eps, .tif, .jpg (one file per figure). For guidance, download the 'Figure Guide PDF' (https://www.embopress.org/page/journal/17574684/authorguide#figureformat).

3) A .docx formatted letter INCLUDING the reviewers' reports and your detailed point-by-point responses to their comments. As part of the EMBO Press transparent editorial process, the point-by-point response is part of the Review Process File (RPF), which will be published alongside your paper.

4) A complete author checklist, which you can download from our author guidelines (https://www.embopress.org/page/journal/17574684/authorguide#submissionofrevisions). Please insert information in the checklist that is also reflected in the manuscript. The completed author checklist will also be part of the RPF.

5) Please note that all corresponding authors are required to supply an ORCID ID for their name upon submission of a revised manuscript.

6) It is mandatory to include a 'Data Availability' section after the Materials and Methods. Before submitting your revision, primary datasets produced in this study need to be deposited in an appropriate public database, and the accession numbers and database listed under 'Data Availability'. Please remember to provide a reviewer password if the datasets are not yet public (see https://www.embopress.org/page/journal/17574684/authorguide#dataavailability).
In case you have no data that requires deposition in a public database, please state so in this section. Note that the Data Availability Section is restricted to new primary data that are part of this study.

7) For data quantification: please specify the name of the statistical test used to generate error bars and P values, the number (n) of independent experiments (specify technical or biological replicates) underlying each data point and the test used to calculate p-values in each figure legend. The figure legends should contain a basic description of n, P and the test applied. Graphs must include a description of the bars and the error bars (s.d., s.e.m.). Please provide exact p values.

8) We would also encourage you to include the source data for figure panels that show essential data. Numerical data should be provided as individual .xls or .csv files (including a tab describing the data). For blots or microscopy, uncropped images should be submitted (using a zip archive if multiple images need to be supplied for one panel). Additional information on source data and instruction on how to label the files are available at
.

9) Our journal encourages inclusion of *data citations in the reference list* to directly cite datasets that were re-used and obtained from public databases. Data citations in the article text are distinct from normal bibliographical citations and should directly link to the database records from which the data can be accessed. In the main text, data citations are formatted as

follows: "Data ref: Smith et al, 2001" or "Data ref: NCBI Sequence Read Archive PRJNA342805, 2017". In the Reference list, data citations must be labeled with "[DATASET]". A data reference must provide the database name, accession number/identifiers and a resolvable link to the landing page from which the data can be accessed at the end of the reference. Further instructions are available at .

10) We replaced Supplementary Information with Expanded View (EV) Figures and Tables that are collapsible/expandable online. A maximum of 5 EV Figures can be typeset. EV Figures should be cited as 'Figure EV1, Figure EV2" etc... in the text and their respective legends should be included in the main text after the legends of regular figures.
- For the figures that you do NOT wish to display as Expanded View figures, they should be bundled together with their legends in a single PDF file called *Appendix*, which should start with a short Table of Content. Appendix figures should be referred to in the main text as: "Appendix Figure S1, Appendix Figure S2" etc.
- Additional Tables/Datasets should be labeled and referred to as Table EV1, Dataset EV1, etc. Legends have to be provided in a separate tab in case of .xls files. Alternatively, the legend can be supplied as a separate text file (README) and zipped together with the Table/Dataset file.
See detailed instructions here:
.

11) The paper explained: EMBO Molecular Medicine articles are accompanied by a summary of the articles to emphasize the major findings in the paper and their medical implications for the non-specialist reader. Please provide a draft summary of your article highlighting
- the medical issue you are addressing,
- the results obtained and
- their clinical impact.
This may be edited to ensure that readers understand the significance and context of the research. Please refer to any of our published articles for an example.

12) For more information: There is space at the end of each article to list relevant web links for further consultation by our readers. Could you identify some relevant ones and provide such information as well? Some examples are patient associations, relevant databases, OMIM/proteins/genes links, author's websites, etc...

13) Conflict of interest: We updated our journal's competing interests policy in January 2022 and request authors to consider both actual and perceived competing interests. Please review the policy https://www.embopress.org/competing-interests and update your competing interests if necessary.

14) Every published paper now includes a 'Synopsis' to further enhance discoverability. Synopses are displayed on the journal webpage and are freely accessible to all readers. They include a short stand first (maximum of 300 characters, including space) as well as 2-5 one-sentences bullet points that summarizes the paper. Please write the bullet points to summarize the key NEW findings. They should be designed to be complementary to the abstract - i.e. not repeat the same text. We encourage inclusion of key acronyms and quantitative information (maximum of 30 words / bullet point). Please use the passive voice. Please attach these in a separate file or send them by email, we will incorporate them accordingly.
Please also suggest a striking image or visual abstract to illustrate your article as a PNG file 550 px wide x 300-600 px high.

15) As part of the EMBO Publications transparent editorial process initiative (see our Editorial at http://embomolmed.embopress.org/content/2/9/329), EMBO Molecular Medicine will publish online a Review Process File (RPF) to accompany accepted manuscripts.
In the event of acceptance, this file will be published in conjunction with your paper and will include the anonymous referee reports, your point-by-point response and all pertinent correspondence relating to the manuscript. Let us know whether you agree with the publication of the RPF and as here, if you want to remove or not any figures from it prior to publication.
Please note that the Authors checklist will be published at the end of the RPF.

EMBO Molecular Medicine has a "scooping protection" policy, whereby similar findings that are published by others during review or revision are not a criterion for rejection. Should you decide to submit a revised version, I do ask that you get in touch after three months if you have not completed it, to update us on the status.

I look forward to receiving your revised manuscript.

With kind regards,

Lise Roth

Lise Roth, PhD
Editor

***** Reviewer's comments *****

Referee #1 (Remarks for Author):

This elegant study from Drs Feske and Weilinger's group had characterized the various immune cell subsets in the colonic lamina propria of therapy refractory IBD patients and investigated the effects of the SOCE inhibitors on intestinal immune cell populations from IBD patients. The authors used patient's samples as well as various KO mice along with the pharmacological approach to evaluate the role of SOCE in IBD. Overall, while the manuscript is potentially interesting, the data could be further strengthened by the following experiments.

Specific comments

1. The data presented in figure 1 shows that calcium entry was inhibited (due to knockdown of various calcium signaling genes) in various T cell populations. However, one of the questions still remains, if inhibition of these genes had any effect on the development of other T cell types or was it specific for Th1 and Th17. Perhaps looking at Th2, Th9, Th22, and Tfh might explain some of the results. In addition, perhaps a control for CD8+ cells (which should not be knocked down in these conditions) could also be used. Finally, instead of showing traces, could the PI separate the ER release and calcium entry, which might give a better picture as if both of them are altered or only SOCE is affected in these cells. Although the injection of Orai1-deficient CD4+ T cells did somewhat attenuated weight-loss, the data is very close to that observed with WT. However, STIM1 KO significantly gained weight, though the levels of cytokines between Orai1 and STIM1 KO were same, suggesting that perhaps other calcium channels (Orai2/3 or TRPC1) could also play a role, which needs to be evaluated/discussed.

2. The data presented in figure 2 is interesting, but only a fraction of cells showed IL-2 production, which was decreased by BTP2. The question that still remain is if these cells are different, or if the same cell produced TNFa, IFNg and other cytokines, which will again be interesting and could suggest clonal differences. Also, why was CD3 + LPMCs were used and not CD4+? Another interesting observation was that some of the cell surface markers (CD25, which is important for autoimmune disease) were actually up regulated in BTP2-treated cells. This begs the question if treated with BTP2 as suggested here, could this lead to other complications. Interestingly, a previous publication from Dr. Muallem's (Ahuja e al, 2017) group had suggested for caution in the use of Orai1 inhibitors for acute inflammatory diseases.

3. Again, the data presented in figure 3-5 are of high interest, which although showed that B cells were significantly expanded compared to non-inflamed controls. Also had significantly fewer CD4+CD45RO+IL7R+ and CD8+CD45RO+ effector T cells; however, one of the caveats of the data is that they only represent a snapshot of these patients and may not represent the true condition as the disease development is more dynamic. One way to solve this will be to perform adoptive transfer and evaluate these phenotypes at regular time points to have a better picture. However, I realize that such an elaborate experiment might not be feasible and just discussing the caveat may be enough.

4. Looking at NK cells is although good, another thing to consider is looking at macrophages which are the gate keepers of intestinal immune homeostasis and see if they have similar phenotypes that might also explain some of the data. Also, most of the data presented uses BTP2, but for the clinical findings they have used CM460 and perhaps showing that it has similar effects on T, B cells as observed with BTP2 might be good. One data that has been puzzling is that BTP2 did not showed any effect on intestinal epithelial cells, which is odd as previous studies have shown that epithelial cells need calcium for its proliferation/survival.

In summary, the authors report interesting findings, but additional experiments/ explanations are needed to prove their point.

Referee #2 (Remarks for Author):

In their study, Marilena and colleagues investigated immune cells including CD4+ and CD8+ effector T cells, T regulatory cells and innate lymphoid cells from the lamia propria of patients suffering from ulcerative colitis and Crohn's disease. UC and CD are characterized by elevated presence of these cells and elevated cytokine production. The authors investigated altered immune cell composition in the lamina propria of patients with ulcerative colitis and Crohn's disease and compared it with immune cells from non-inflamed tissue using CyTOF. They also compared expression markers/cytokines dependent on BTP2 (SOCE blocker). They in depth investigated CD45+CD3+ T cells and CD45+CD3- non T-cells (20 subsets each, multiple lineage/expression markes/cytokines were checked for classification) of patients/controls with BTP2. In these cells interleukine production depends on SOCE and pharmacological block of SOCE by BTP2 resulted in reduced production of several key cytokines including

interleukines, TNFα and IFNγ in human colonic T cells and innate lymphoid cells, reduced the production of IL-6 by several of patient's B cell subsets and reduced production of IFNγ by myeloid cells, without affecting intestinal epithelial cells. Effects were more pronounced in T-cell subsets demonstrationg the significance for T cell activity in IBD. In transgenic mice, they can show that SOCE components (STIM1, STIM2, STIM1+STIM2, and Orai1 knockouts) distinctively contribute to SOCE and that the reduction of SOCE correlates with IL production, differentiation of T cells and severity of IBD in vivo also without affecting intestinal epithelial cells in mice. I am impressed by the comprehensive and detailed results linking each SOCE component KO to a Ca2+ phenotype and complete IL panels and T cell subsets have been analysed. They also demonstrate that a new selective CRAC channel blocker (CM4620, phase 2 clinical trials for COVID-19 associated pulmonary inflammation and acute pancreatitis (NCT04195347)) tackled IBD in mice via a reduction of T cell function. With their holistic approach, the authors here present a novel pharmaceutical strategy to treat IBD in patients.
This work is of high originality and significance in the field of STIM1/Orai channels, Ca2+ signaling, immune cell function, molecular medicine, biomedical research and IBD (ulcerative colitis and Crohn's disease) and will be of interest to a broad audience of readers. The study will push forward SOCE and IBD research and kick off other research fields that are associated with SOCE malfunction including several fields of cancer research (CRC, PCa, breast cancer...) and research in other immune-related fields (e.g. multiple sclerosis). The design of the study, the experiments and experimental settings (mass cytometry (CyTOF), Ca2+ imaging, transgenic mice, IBD patients) are highly suitable and of high quality. This is not a surprise as both labs (Feske and Weidinger) are known for their high quality, cutting edge research.
My overall recommendation is to publish this study as it reveals a new therapeutic approach in the treatment of IBD, findings from experiments (patients and mice) are conclusive, and a molecular mechanism for this novel pharmacological approach has been identified.
I have some minor points and I am also curious about few points:
• Page 5: Likewise, CD4+ T cells from Orai1R93W knock-in mice that express a channel-dead version of ORAI1 lacked SOCE almost completely, likely because of a dominant negative effect of the mutant ORAI1 protein on other ORAI paralogues (ORAI2, ORAI3) with which ORAI1 can form heteromeric channels 20, 22.
The dominant negative effect of OraiR91W on Orai2 and Orai3 has first been shown by Lis et al. current biology, 2007 und thus this is the right reference to cite.
• Do the authors have an idea what are the bases of the weight loss of Rag1-/- mice when Orai1 deficient T cells are transferred? (This may not be of importance for this study, I am just curious)
• Did the authors observe any sex based differences in their findings (in mice as they used sex matched Rag1-/- mice)?
• Fig. 2D In this experimental setting the authors activate SOCE artificially (ionomycin is a quite harsh treatment) and block it with BTP2 and measure IL-2 production. Did they perform the same experiments without artificial activation of CRAC channels? The CRAC channels should be activated during IL2 production per se and one should be able to see the BTP2 effect.
• Maybe I overlook it, but could the authors comment on why cytokine production was only inhibited 50% in CD4+CD25+IL7R- Treg cells (Fig.2E) or almost not affected in CD19+HLA-DR+. Do the authors have an idea, what are expression levels of Orai2/Orai3 in these cells (compared to other T cells). Orai3 is less inhibited by BTP2 than Orai1 (https://pubmed.ncbi.nlm.nih.gov/32896813/)?
• Why are some markers (CD25 in CD4+and CD8+) upregulated upon BTP2 treatment, 'a cell type dependent threshold' probably does not explain why the effect goes in the opposite direction: An upregulation of markers is observed.
• Fig. 7a: If biophysical properties of CRAC are unchanged, the current in DVF solution should be larger than in 20 mM Ca2+. Can the authors comment on this? Please also display the outward currents analyzed at +80 mV and/or the IV during La3+ and DVF application.

Referee #3 (Remarks for Author):

This study investigates the role of SOCE in the development and function of immune cells in IBD, and tests the therapeutic potential of a SOCE inhibitor in a mouse model of IBD. The authors examine the relationship of calcium signaling strength, T cell differentiation/polarization, function, and contribution to T cell transfer colitis using a collection of STIM/ORAI single and double mutant mice. This is then coupled with cytof-based analysis of development and calcium-dependent function of lamina propria immune cells from IBD patients. The authors conclude that SOCE regulates immune cell-driven pathology and identify SOCE inhibitors as potential therapeutic agent for IBD. In general the study is well written and synthesizes a large body of data clearly. There are however, some issues with experimental design and/or analysis requiring clarification in order to strengthen the study's conclusions.

Major points:
1. Figure 1 - SOCE has less effect of Tregs? Conditions used to evaluate Th1, Th17 is quite different from Treg. In the first case cells are subjected to two calcium-dependent activation events (differentiation on CD3/CD28 + cytokine cocktail followed by PMA/iono stim) vs one for Tregs (differentiation only). It seems that to directly compare the magnitude of effect of SOCE on T cell differentiation the assays should be comparable, ie looking at restimulated cytokine production in iTregs or relevant transcription factors pre-stim for Th1/Th17.

For Orai1f/fCD4Cre colitis transfer model - it was previously reported by the Feske lab that Orai1f/fCD4Cre T cells induce colitis equivalent to WT. Why the difference in phenotype here?

2. Figure 2 - This figure examines the impact of SOCE inhibition on calcium flux and cytokine production in LP T cells from IBD patients. In A and B, cells are incubated overnight (16h) in BTP2 prior to calcium studies. Why were the cells exposed to the inhibitor for so long? It is also not clear whether the BTP2 is also present during the calcium assay or just the overnight incubation? It is important to distinguish between longer term effect of the inhibitor on cell survival/health, vs the acute effect of BTP2 on SOCE. For this experiment the acute effect of BTP2 on SOCE seems more relevant. The experiment performed to assess the effect of BTP2 on cell viability (suppl Fig 2), only looks at a 4h time point, relevant to the experiments in 2C-G, but not the calcium experiments in 2A-B.
The calcium influx and effect of the inhibitor is compared somewhat indirectly to PBMC's from healthy controls (in Suppl. Figure 1). It is actually quite notable, but not mentioned in the text, that the magnitude and rate of calcium influx is markedly higher in healthy PBMC's. Whether this is due to the difference in cell population (PBMCs) vs healthy/IBD host is unclear, but is important to understand if/how SOCE is dysregulated in IBD. In order to better understand the subsequent experiments comparing T cell subsets, cytokine production and the effect of SOCE inhibition in IBD vs non-inflamed patients it would be helpful to directly compare calcium flux, and the effect of the inhibitor, in IBD vs non-inflamed LP cells.
The data presented in 2E-G only shows change in expression does not include the change in expression compared with unstimulated cells (except IL2 in 2D). Since direction of expression change with activation can be either up or down, and may differ between cell populations, it would be helpful to show this data (at least in the supplement), to best interpret the impact of BTP2.

3. Figure 3. These experiments aim to compare the frequencies and functional status (as determined by stimulated cytokine production and activation markers) in IBD vs control LPMC. Comparison of frequency of cell populations based on lineage and activation markers is shown in 3B-E, however the data shown are after 4h restimulation with P/I. Since the goal is to "define the UC and CD-specific immune cell composition", it seems that the more relevant data to show here are the populations in the unstimulated condition since much can change during stimulation including activation markers, subpopulation-specific cell survival etc. The basal cytokine signature would also be interesting to see, since these patients are presumably having a disease flare (necessitating bowel resection) and cytokine expression is likely already elevated in LPMC. How these markers and cytokine expression change with SOCE-dependent stimulation, and compare amongst non-inflamed, and IBD samples is a separate question, and also important.

In the text top of p10 - "with a particular involvement of CD38+ plasma cells producing IgA and IgM in CD not UC patients". Is this statement correct? It appears in the figure that 38+ IgA or IgM+ cells are altered in UC, not CD, compared to uninflamed.
4. Figure 4 and 5. Related to the comment on figure 3, fully interpreting the effect of BTP2 on stimulated cytokine production requires understanding how these markers changed from unstimulated to stimulated conditions. Also not shown is whether BTP2 differentially impacts activation marker/cytokine expression from noninflamed compared with IBD LPMCs. This would be important to know from a therapeutic perspective, particularly if considering longer term therapy - will SOCE inhibition inhibit function of healthy cells more or less than those in the inflammatory environment?
5. Figures 7. Experiments with a different SOCE inhibitor demonstrate the ability of a CRAC channel inhibitor to improve disease progression in a mouse model. Although the data with CM4620 is convincing, to connect these experiments with the rest of the study it would be better to include at least some direct comparison of BTP2 and CM4620, and data showing the impact of CM4620 on primary T cell SOCE or function since not all inhibitors function identically and may have different activity depending on the ORAI isoform combination in each cell type.
In Figure 7J it is not clear whether CM4620 is also used during the P/I restimulation. If not, what is the proposed mechanism for the decreased cytokine production in the extracted cells? Altered activation or priming state due to direct effect of the inhibitor or environment?

Minor Points:
- Figure 2G. Text says CD11c + myeloid cells, but not clear from the figure or figure legend which myeloid population/s is shown.
- In the text p. 7 "alternating requirements of cell-specific thresholds of SOCE signaling strength". It is unclear to me what "alternating requirements" refers to.

Point-by-Point Response to Reviewer Comments

Referee #1

This elegant study from Drs Feske and Weidinger's group had characterized the various immune cell subsets in the colonic lamina propria of therapy refractory IBD patients and investigated the effects of the SOCE inhibitors on intestinal immune cell populations from IBD patients. The authors used patient's samples as well as various KO mice along with the pharmacological approach to evaluate the role of SOCE in IBD. Overall, while the manuscript is potentially interesting, the data could be further strengthened by the following experiments.

Response: We thank the reviewer for the positive evaluation of our manuscript and the thoughtful comments.

Specific comments

1. The data presented in figure 1 shows that calcium entry was inhibited (due to knockdown of various calcium signaling genes) in various T cell populations. However, one of the questions still remains, if inhibition of these genes had any effect on the development of other T cell types or was it specific for Th1 and Th17. Perhaps looking at Th2, Th9, Th22, and Tfh might explain some of the results.

Response: We agree with the reviewer that it would be indeed very interesting to study the role of SOCE in the differentiation of Th9, Th22 and Tfh cells. However, in the T transfer model of colitis we used in this study, inflammation is mediated by Th17 and Th1 cells, and suppressed by Treg cells. This is why we have focused in our analysis shown in **Figure 1** on Th1, Th17 and Treg cells. While studying the role of SOCE in Th9, Th22 and Tfh cells would indeed be interesting, it is outside of the scope of this study.

In addition, perhaps a control for $CD8^+$ cells (which should not be knocked down in these conditions) could also be used.

Response: We thank the reviewer for the opportunity to comment on this issue. In the Cd4-Cre mice we used for our studies, *Stim1, Stim2* and *Orai1* genes are deleted in both $CD4^+$ and $CD8^+$ T cells because the CD4 promoter is expressed in $CD4^+CD8^+$ double-positive immature T cells during thymic development. For example, we showed that *Stim1*$^{fl/fl}$ *Cd4-Cre* mice have reduced SOCE in $CD4^+$ and $CD8^+$ T cells (*Desvignes et al.,* J Clin Invest 2015) and we have previously used *Stim1*$^{fl/fl}$ *Stim2*$^{fl/fl}$ *Cd4-Cre* mice to investigate the role of SOCE in $CD8^+$ T cell for antitumor immunity (Weidinger et al., Embo Mol Med 2013) and antiviral immunity (Shaw et al., J Clin Invest 2014).

Finally, instead of showing traces, could the PI separate the ER release and calcium entry, which might give a better picture as if both of them are altered or only SOCE is affected in these cells.

Response: We thank the reviewer for this comment. Because we stimulated T cells with ionomycin in the presence of 1 mM extracellular Ca^{2+}, we cannot distinguish Ca^{2+} release from the ER from extracellular Ca^{2+} entry in the these experiments. However, there is ample previous work in the literature from our own group and others showing that the Ca^{2+} content of the ER is not significantly altered in the absence in *Orai1* or *Stim1* deficient T cells (Gwack et al. 2008, Mol Cell Biol; Vaeth et al., Nature Communications 2017).

Although the injection of Orai1-deficient $CD4^+$ T cells did somewhat attenuated weight-loss, the data is very close to that observed with WT. However, STIM1 KO significantly gained weight, though the levels of cytokines between Orai1 and STIM1 KO were same, suggesting that perhaps other calcium channels (Orai2/3 or TRPC1) could also play a role, which needs to be evaluated/discussed.

Response: We thank the reviewer for this comment. Mice injected with *Stim1*-deficient T cells indeed gained some weight (which is due to the fact that recipient mice were young and still growing) whereas mice injected with *Orai1*-deficient T cells showed moderate weight loss. These data are consistent with the severity of colitis as analyzed by histology and quantified by the colitis score in **Figure 1C**. Mice injected with *Stim1*-deficient T cells had the lowest colitis score, where mice injected with *Orai1*-deficient T cells also had an attenuated (but less pronounced) reduction in colitis scores compared to recipients of wildtype T cells. We agree that proinflammatory cytokines (IL-17A, IFNγ, TNFα) were equally reduced in *Orai1*- and *Stim1*-deficient T cells after ex vivo restimulation with PMA/ionomycin, which is a very strong stimulus that bypasses early TCR signaling steps. During antigen-driven T cell activation by gut pathobionts *in vivo* that results in colitis, differences in cytokine production by *Orai1*- and *Stim1*-deficient T cells are likely to be more pronounced, thus explaining the differences in weight loss and colitis severity.

2. The data presented in figure 2 is interesting, but only a fraction of cells showed IL-2 production, which was decreased by BTP2. The question that still remain is if these cells are different, or if the same cell produced TNFα, IFNγ and other cytokines, which will again be interesting and could suggest clonal differences.

Response: We thank the reviewer for this comment and apologize for not explaining our results in more detail. Indeed, only a subset of $CD3^+$ T cells produces IL-2. As mentioned by the reviewer, several T cell subsets among $CD3^+$ LPMCs can produce multiple cytokines simultaneously. For instance, subsets of T cells producing IL-2 can also produce TNFα. By contrast, IL-17A producing T cells appear to be distinct from those producing IL-2. To clarify this point, we have attached viSNE plots showing the expression of the respective cytokines as **Reviewer Figure 1**, which summarize the expression of several cytokines in $CD45^+CD3^+$ LPMCs isolated from 5 CD patients following stimulation with PMA/ionomycin for 4h in the absence and presence of 15-1000 nM BTP2.

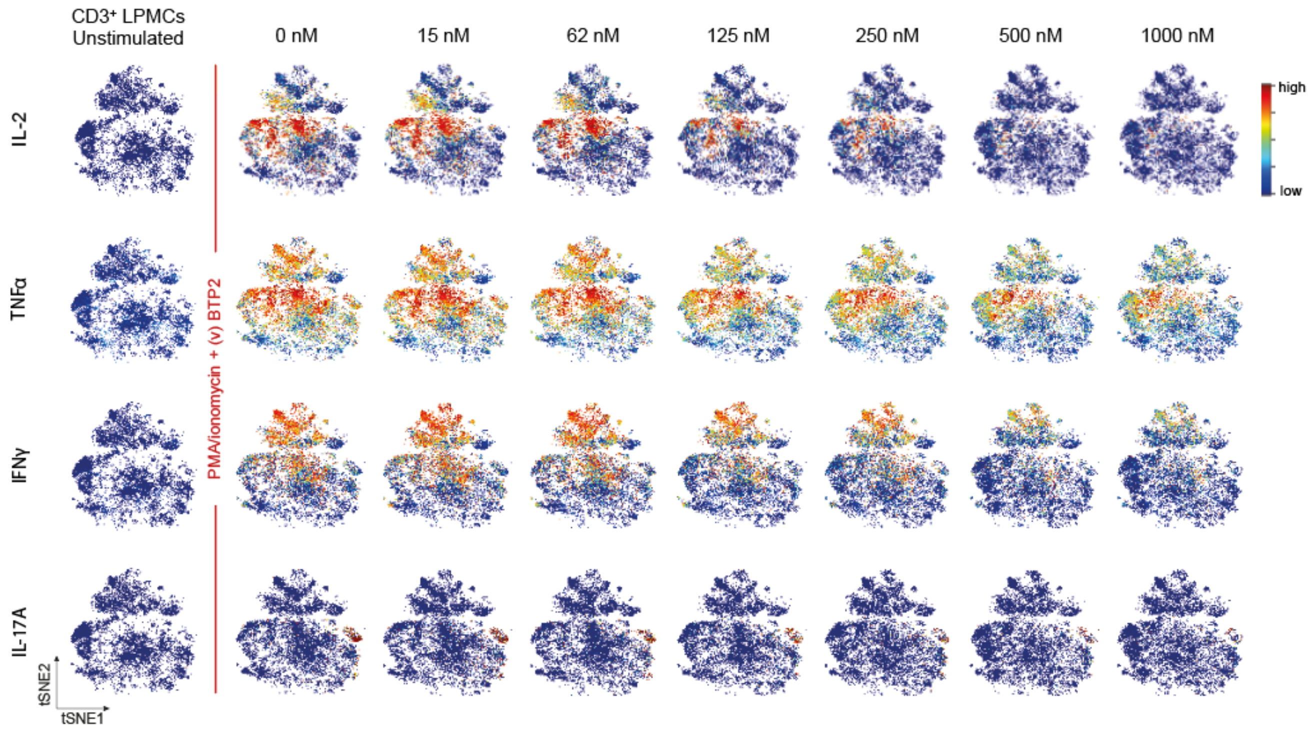

Reviewer Figure 1. Production of different pro-inflammatory cytokines by subsets of CD3$^+$ T cells in the lamina propria of CD patients and dose-dependent suppression of cytokine production by BTP2-mediated inhibition of SOCE. viSNE plots of CD45$^+$CD3$^+$ LPMCs from CD patients (*n* = 5) stimulated for 4h with PMA/ionomycin in the presence of 15-1000 nM BTP2. Unstimulated LPMC were used as control. viSNE plots are colored according to the expression level of the indicated cytokines (blue: low, red: high) and are representative of one CD patient.

Also, why was CD3$^+$ LPMCs were used and not CD4$^+$?

Response: We gated LPMC on CD3$^+$ T cells to capture all T cell populations in our subsequent analysis including CD4$^+$ and CD8$^+$ T cells, which can both produce proinflammatory cytokines.

Another interesting observation was that some of the cell surface markers (CD25, which is important for autoimmune disease) were actually up regulated in BTP2-treated cells. This begs the question if treated with BTP2 as suggested here, could this lead to other complications.

Response: We thank the reviewer for this interesting comment. CD25 is another name for the IL-2 receptor alpha chain, which together with beta and gamma chains forms the high-affinity IL-2 receptor. While CD25 is expressed on activated T cells and resting memory T cells in humans, it is most important function arguably is on Treg cells, for which CD25 often serves as a marker. In addition to serving as a Treg cell marker, CD25 expression is essential for the differentiation and proliferative fitness of Treg cells. Mice lacking CD25 suffer from massive systemic autoimmunity that can be reversed by the adoptive transfer of CD4$^+$CD25$^+$ T cells from wild-type mice. The increase in CD25 following BTP2 treatment mentioned by the reviewer was most prominent in CD4$^+$ IL-7R$^+$ T cells. Because IL-7R signaling in Treg cells supports the functional activity of effector Treg cells by increasing their IL-2 sensitivity (Schmaler et al. 2015, PNAS), we think that the increase in CD25 expression on CD4$^+$ T cells treated with BTP2 (**Figure 2D**) may be indicative of enhanced Treg cell function. As a consequence, we would expect an immunosuppressive effect of BTP2 treatment rather than "complications".

Interestingly, a previous publication from Dr. Muallem's (Ahuja e al, 2017) group had suggested for caution in the use of Orai1 inhibitors for acute inflammatory diseases.

Response: We thank the reviewer for this comment. The paper by Ahuja et al. (2017, Cell Metabolism) used mice with conditional deletion of *Orai1* specifically in pancreatic acinar cells, and found strong intestinal dysbiosis, bacteremia and early death of mice, which the authors attributed to reduced production of antimicrobial peptides by pancreatic acinar cells. It is therefore a valid concern that systemic treatment of patients or mice may result in intestinal inflammation. Several lines of empiric evidence, however, argue against this concern:

- Patients with loss-of-function mutations in ORAI1 or STIM1 suffer from immunodeficiency but not from IBD or intestinal dysbiosis.
- Systemic injection of wild-type mice with CRAC channel inhibitors for the treatment of EAE, a mouse model of multiple sclerosis did not show signs of intestinal inflammation (Kaufmann et al. 2016, J Immun) and treatment of mice with the CRAC-channel inhibitor CM4620 attenuated IBD severity rather than exacerbating it (**Figure 7** of our current study).
- Systemic treatment of mice with CRAC channel inhibitors strongly reduced the severity of pancreatitis in several animal models of the disease (Waldron et al. 2015, J Physiol; Wen et al. 2015, Gastroenterology).
- Systemic treatment of patients with the CRAC channel inhibitor CM4620 (Auxora) in two phase 2 clinical trials (for acute pancreatitis and COVID19 pneumonia) was not associated with apparent signs of IBD or dysbiosis (NCT03401190 and NCT04345614) but attenuated inflammation.

We therefore think that caution regarding the use of Orai1 inhibitors is not warranted because the vast majority of available data indicate that CRAC channel inhibition has anti-inflammatory effects, which is consistent with data in our current study.

3. Again, the data presented in figure 3-5 are of high interest, which although showed that B cells were significantly expanded compared to non-inflamed controls. Also had significantly fewer $CD4^+CD45RO^+IL7R^+$ and $CD8^+CD45RO^+$ effector T cells; however, one of the caveats of the data is that they only represent a snapshot of these patients and may not represent the true condition as the disease development is more dynamic. One way to solve this will be to perform adoptive transfer and evaluate these phenotypes at regular time points to have a better picture. However, I realize that such an elaborate experiment might not be feasible and just discussing the caveat may be enough.

Response: We agree with the reviewer that our results represent a snapshot of IBD disease activity in patients with CD and UC. It would indeed be very interesting to perform longitudinal studies comparing the composition and function of immune cells in the lamina propria of IBD patients over time. However, because CyTOF analyses of stimulated cells require a lot of cells, we can only work with resectates of IBD patients and not punch-biopsies obtained from endoscopy. The latter are suitable for longitudinal studies, but resectates of entire gut sections are not, for obvious reasons, as such patient material is very rare. Adoptive T cell transfer experiments in humans are not possible for experimental reasons (for instance, adoptive T cell transfer mouse models use immunodeficient recipient mice, typically $Rag^{-/-}$ mice that lack Treg cells, which is not possible in human patients) and ethical concerns. Because of these reasons, it is almost impossible to study the dynamics of IBD development in patients at the cellular level.

4. Looking at NK cells is although good, another thing to consider is looking at macrophages which are the gate keepers of intestinal immune homeostasis and see if they have similar phenotypes that might also explain some of the data.

Response: We thank the reviewer for raising this important point. We fully agree that myeloid cells, including monocytes, macrophages, and dendritic cells play an important role in intestinal immune homeostasis. We had indeed included an analysis of myeloid cells in the original **Figure 5**. We apologize if the description of the figure was not clear. We have now included viSNE plots into the new **Extended View Figure 2** showing the different myleoid cell clusters (see also **Reviewer Figure 2**). We now highlight 4 clusters of myeloid cells, such as CD11b$^+$ CD11c$^+$ dendritic cells (cluster 1), CD14$^+$ HLA-DR$^+$ monocytes (cluster 2), CD14$^+$ HLA-DR$^+$ IL-6$^+$ monocytes (cluster 3) and CD11c$^+$CD38$^+$ macrophages (cluster 20). Importantly, although we observed myeloid cell clusters in the lamina propria of IBD patients and non-inflamed controls, we could not detect

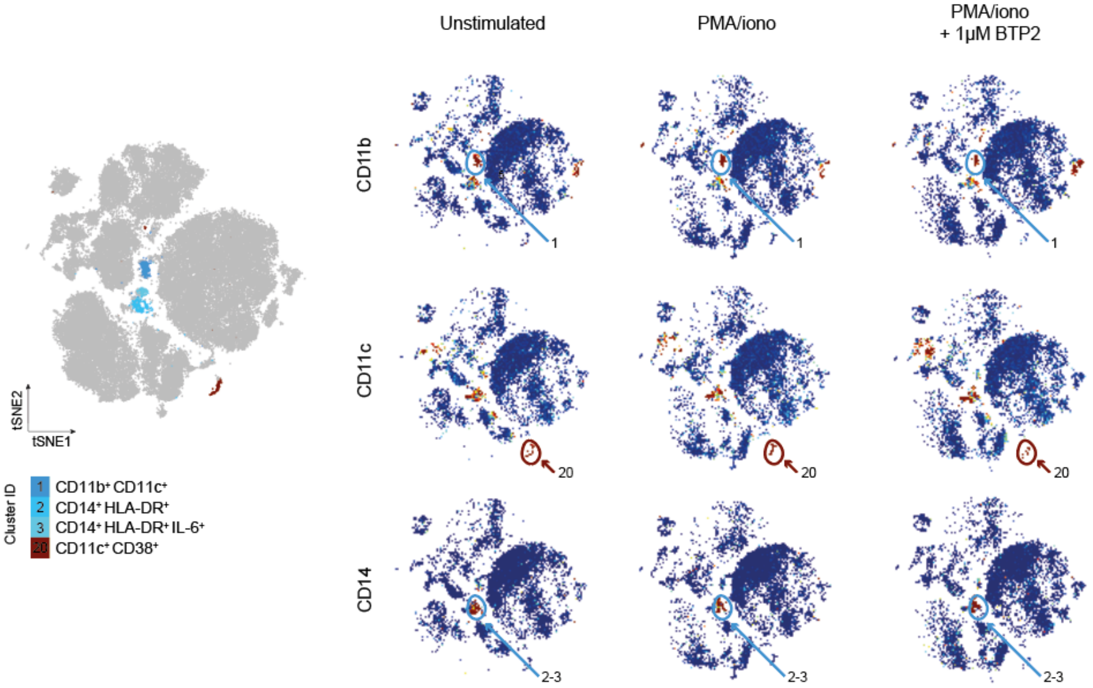

Reviewer Figure 2. Four clusters of myeloid cells in the colon lamina propria (CLP) of IBD patients and effects of SOCE inhibition. (Left) FlowSOM plot of merged FCS files from unstimulated IBD samples and samples treated with PMA/ionomycin ± 1 µM BTP2 (non-inflamed: n = 4, CD: n =6, UC: n =6). Colors indicate myeloid cell clusters within CD45$^+$CD3$^-$ LPMCs. (Right) viSNE plots of one exemplary CD patient. Colors indicate expression levels of cell surface markers (blue: low; red: high) in cells left unstimulated, stimulated with PMA/ionomycin or stimulated in the presence of 1 µM BTP2**.**

significant changes in the frequencies of these clusters after treatment the SOCE inhibitor BTP2 (**Reviewer Figure 2** and **Figure 5C**). We did, however, detect a significant reduction of IFNγ-production by CD11b$^+$ CD11c$^+$ dendritic cells (cluster 1) and CD14$^+$ HLA-DR$^+$ monocytes (cluster 2) after treatment of LPMCs isolated from CD but not UC patients with 1 µM BTP2 (**Figure 5 D**) indicating that SOCE contributes to pro-inflammatory cytokine production by myeloid cells in the LP. We have included the viSNE plots as a new **Extended View Figure 2** in the revised manuscript.

Also, most of the data presented uses BTP2, but for the clinical findings they have used CM460 and perhaps showing that it has similar effects on T, B cells as observed with BTP2 might be good.

Response: We thank the Reviewer for this clinically relevant suggestion. To show that BTP2 and CM4620 have similar effects on immune cell subsets, we have now isolated PBMCs from 3 healthy volunteers and measured Ca^{2+} influx by flow cytometry after treatment of cells for 4h with 1 µM BTP2 or 1 µM CM4620 (**Reviewer Figure 3**). 1 µM CM4620 blocked Ca^{2+} influx in $CD4^+$ or $CD8^+$ T, $CD19^+$ B cells, $CD56^+$ NK cells and $CD14^+$ myeloid cells similarly to 1 µM BTP2. In analogy to our previous experiments shown in **Figure 2,** we furthermore compared the functional impact of CM4620 on cytokine production by mass cytometry (CyTOF) in new additional experiments. Briefly, we observed a strong suppression of pro-inflammatory cytokines including INFγ, TNFα, IL-2, IL-6 and IL-17A in various lymphocyte subsets, highlighting that CM4620 acts as a potent inhibitor of lymphocyte function similar to BTP2. We have included these new Calcium-measurements and CyTOF experiments in the revised manuscript as **new Extended Figure 4+5** and included a description of our findings in the revised **Results** section on page 15 and the revised **Discussion** section on page 21-22**.** Please see also **Reviewer Figures 18 and 19** for a detailed description of our new CyTOF experiments.

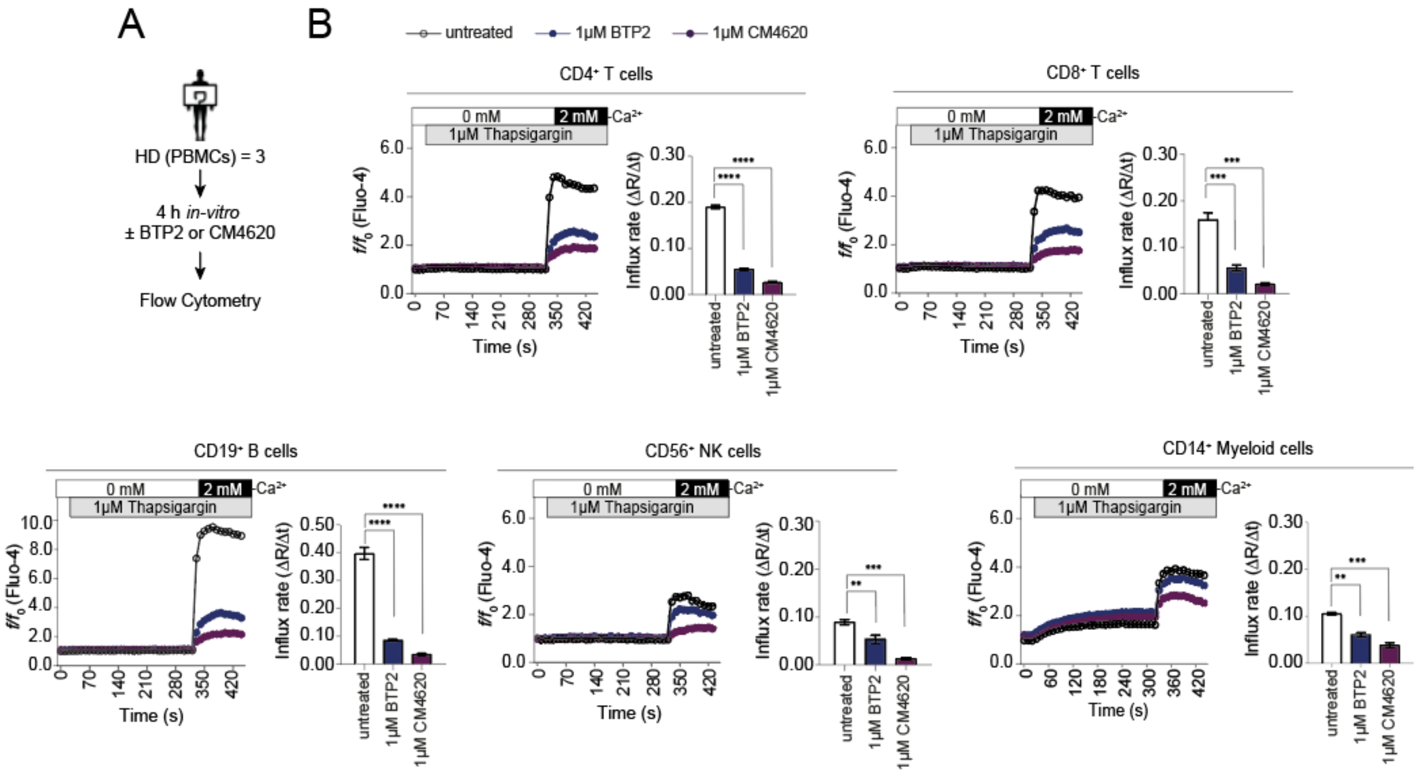

Reviewer Figure 3. Similar effects of CRAC channel inhibition with CM4620 and BTP2 on Ca2+ influx in human immune cell subsets. (A) Experimental design for Ca^{2+} influx measurements in PBMCs of three healthy donors (HD) by flow cytometry. (**B**) Ca^{2+} influx rates were measured in $CD4^+$ or $CD8^+$ T cells, $CD19^+$ B cells, $CD56^+$ NK cells and $CD14^+$ myeloid cells following pre-incubation with 1 µM BTP2 or 1 µM CM4620 for 4 h. Cells were stimulated with thapsigargin in Ca^{2+} free buffer followed by addition of 2 mM Ca^{2+} Ringer solution. Bar graphs show the mean values of influx rates after addition of 2 mM $Ca^{2+.}$ Statistical significance was calculated using repeated measures (RM) one-way ANOVA test. ****p<0.0001 ***p<0.001 **p<0.01 *p<0.05.

One data that has been puzzling is that BTP2 did not showed any effect on intestinal epithelial cells, which is odd as previous studies have shown that epithelial cells need calcium for its proliferation/survival.

Response: We thank the reviewer for this thoughtful comment. We were actually surprised to see that the proliferation of intestinal epithelial cells was preserved in the presence of BTP2. However, these findings are supported by several other lines of evidence:

- Patients with loss-of-function mutations in STIM1 or ORAI1, and *Orai1*$^{-/-}$ mice do not have intestinal inflammation that could be due to impaired intestinal barrier function.
- A paper by Liang et al. recently investigated the impact of *Stim1* deletion in intestinal epithelial cells (IEC) using *Stim1*$^{fl/fl}$ *Villin-Cre* mice (Liang et al, CMGH 2022, https://doi.org/10.1016/j.jcmgh.2022.03.007). The authors of that study observed normal intestinal development as well as preserved proliferation and differentiation of IEC under steady-state conditions.

We updated the Discussion section on **page 21** accordingly to include these findings and discuss the reviewer's critique.

Referee #2

In their study, Marilena and colleagues investigated immune cells including CD4$^+$ and CD8$^+$ effector T cells, T regulatory cells and innate lymphoid cells from the lamia propria of patients suffering from ulcerative colitis and Crohn's disease. UC and CD are characterized by elevated presence of these cells and elevated cytokine production. The authors investigated altered immune cell composition in the lamina propria of patients with ulcerative colitis and Crohn's disease and compared it with immune cells from non-inflamed tissue using CyTOF. They also compared expression markers/cytokines dependent on BTP2 (SOCE blocker). They in depth investigated CD45$^+$CD3$^+$ T cells and CD45$^+$CD3$^-$ non T-cells (20 subsets each, multiple lineage/expression markes/cytokines were checked for classification) of patients/controls with BTP2. In these cells interleukine production depends on SOCE and pharmacological block of SOCE by BTP2 resulted in reduced production of several key cytokines including interleukines, TNFα and IFNγ in human colonic T cells and innate lymphoid cells, reduced the production of IL-6 by several of patient's B cell subsets and reduced production of IFNγ by myeloid cells, without affecting intestinal epithelial cells. Effects were more pronounced in T-cell subsets demonstrationg the significance for T cell activity in IBD. In transgenic mice, they can show that SOCE components (*Stim1*, *Stim2*, *Stim1*+ *Stim2*, and *Orai1* knockouts) distinctively contribute to SOCE and that the reduction of SOCE correlates with IL production, differentiation of T cells and severity of IBD in vivo also without affecting intestinal epithelial cells in mice. I am impressed by the comprehensive and detailed results linking each SOCE component KO to a Ca^{2+} phenotype and complete IL panels and T cell subsets have been analysed. They also demonstrate that a new selective CRAC channel blocker (CM4620, phase 2 clinical trials for COVID-19 associated pulmonary inflammation and acute pancreatitis (NCT04195347)) tackled IBD in mice via a reduction of T cell function. With their holistic approach, the authors here present a novel pharmaceutical strategy to treat IBD in patients. This work is of high originality and significance in the field of STIM1/ORAI channels, Ca^{2+} signaling, immune cell function, molecular medicine, biomedical research and IBD (ulcerative colitis and Crohn's disease) and will be of interest to a broad audience of readers. The study will push forward SOCE and IBD research and kick off other research fields that are associated with SOCE malfunction including several fields of cancer research (CRC, PCa, breast cancer...) and research in other immune-related fields (e.g. multiple sclerosis). The design of the study, the experiments and experimental settings (mass cytometry (CyTOF), Ca^{2+} imaging, transgenic mice, IBD patients) are highly suitable and of high quality. This is not a surprise as both labs (Feske and Weidinger) are known for their high quality, cutting edge research. My overall recommendation is to publish this study as it reveals a new therapeutic approach in the treatment of IBD, findings from experiments (patients and mice) are conclusive, and a molecular mechanism for this novel pharmacological approach has been identified.

Response: We thank the reviewer for the positive feedback on our manuscript and will address the reviewer's comments below.

I have some minor points and I am also curious about few points:

(1) Page 5: Likewise, $CD4^{+}$ T cells from *Orai1*^{R93W} knock-in mice that express a channel-dead version of ORAI1 lacked SOCE almost completely, likely because of a dominant negative effect of the mutant ORAI1 protein on other ORAI paralogues (ORAI2, ORAI3) with which ORAI1 can form heteromeric channels 20, 22. The dominant negative effect of *OraiR91W* on *Orai2* and *Orai3* has first been shown by Lis et al. current biology, 2007 und thus this is the right reference to cite.

Response: Thank you for alerting us to this oversight. The reviewer is correct in saying that mutations in ORAI1 can function as dominant negatives on CRAC channel function. The study by Lis et al. (PMID: 17442569), however, showed this effect on ORAI2 and ORAI3 for the E106Q mutation of ORAI1 (not the R91W mutation). It was a study by Thompson et al. (PMID 19075015), which has shown that " incorporation of *R91W* mutant *Orai1* subunits in the CRAC channel pore affects the overall magnitude of its conductance". We have added this paper to the reference list and apologize for not having included tis reference before.

(2) Do the authors have an idea what are the bases of the weight loss of Rag1-/- mice when Orai1 deficient T cells are transferred? (This may not be of importance for this study, I am just curious)

Response: SOCE is not completely abolished in *Orai1*-deficient T cells (**Fig.1A**). And although cytokine production is strongly reduced, the defect is not as strong as in STIM1-deficient T cells (**Fig.1D-E**). This is also apparent in the fact that histological colitis scores (**Fig.1C**) are higher in *Rag1*$^{-/-}$ mice that have received *Orai1*-deficient T cells compared to mice that had received STIM1-deficient T cells. In response to the reviewer's comment and to a comment by reviewer #3 (see page 13), we had the colitis scores in *Rag1*$^{-/-}$ mice reanalyzed by a specialized GI pathologist using a more detailed scoring system (scores 1-17, described in Ostanin et al., PMC2643911) instead of the simplified scoring system we had used for the initial submission (scores 1-5). This reanalysis shows a moderate, but non-significant reduction in colitis scores in *Rag1*$^{-/-}$ mice that had received Orai1-deficient T cells compared to wildtype T cells. We conclude that *Orai1*-deficient T cells retain residual function, which results in weight loss of *Rag1*$^{-/-}$ mice. We added a statement to the revised ***Discussion*** section on page 19.

(3) Did the authors observe any sex based differences in their findings (in mice as they used sex matched *Rag1*$^{-/-}$ mice)?

Response: We thank the reviewer for this interesting question. We used only female animals as T cell donors and recipients for the T cell transfer experiments in **Fig.1** to avoid immune responses based on Y-chromosome encoded antigens. We can therefore not assess potential sex-based differences in our experiments. We have included a statement in the revised ***Methods*** section on **page 30** mentioning the use of female donor and recipient mice in these experiments.

(4) Fig. 2D In this experimental setting the authors activate SOCE artificially (ionomycin is a quite harsh treatment) and block it with BTP2 and measure IL-2 production. Did they perform the same experiments without artificial activation of CRAC channels? The CRAC channels should be activated during IL2 production per se and one should be able to see the BTP2 effect.

Response: We thank the reviewer for this comment and agree that PMA/ionomycin is a strong stimulus. As shown in the leftmost panel of **Fig.2D**, we could not detect any IL-2 production in unstimulated LPMCs by CyTOF. Moreover, as shown in the **Reviewer Figure 4** below, we were not able to detect other cytokines (TNFα, IFNγ, IL-17A) in un-stimulated cells either. We therefore had to activate LPMCs to induce cytokine production and analyze the effects of BTP2.

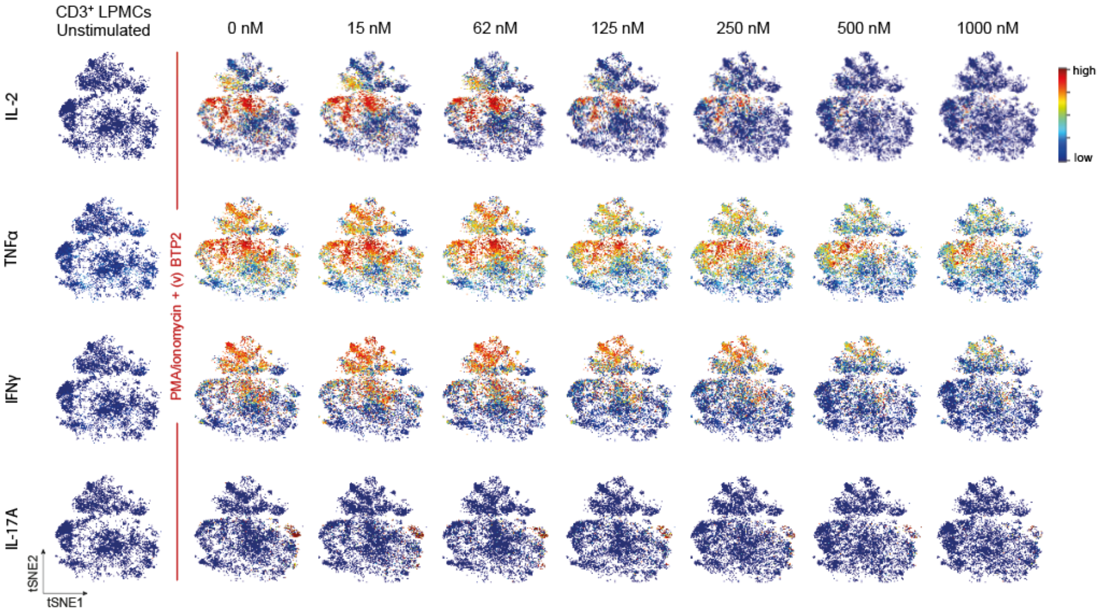

Reviewer Figure 4. Production of IL-2, TNFα, IFNγ and IL_17A by human lamina propria cells is only detectable after stimulation with ionomycin/PMA. No basal cytokine production is observed in unstimulated cells. viSNE plots of $CD45^+CD3^+$ LPMCs (CD: *n* = 5) stimulated 4h with PMA/ionomycin in the presence of 15 - 1000 nM BTP2, with unstimulated samples serving as controls. viSNE plots are colored according to the expression level of cytokines (blue: low, red: high) and are representative of one CD patient.

Maybe I overlook it, but could the authors comment on why cytokine production was only inhibited 50% in $CD4^+CD25^+IL7R^-$ Treg cells (Fig.2E) or almost not affected in $CD19^+$HLA-DR^+. Do the authors have an idea, what are expression levels of Orai2/Orai3 in these cells (compared to other T cells). Orai3 is less inhibited by BTP2 than Orai1 (https://pubmed.ncbi.nlm.nih.gov/32896813/)?

Response: We thank the reviewer for this interesting comment. We agree that a reduced responsiveness of $CD4^+CD25^+$ Treg cells (**Fig.2E**) and $CD19^+$HLA-DR^+ B cells (**Fig.2G**) to BTP2 could theoretically be due to an altered ORAI1 subunit composition of CRAC channels, for instance a higher ORAI3:ORAI1 ratio in myeloid cells, ILC, NK cells, Treg cells or B cells. To test this hypothesis, we have analyzed publicly available single cell RNA sequencing data set of human intestinal lamina propria mononuclear cells (*Smilie et al.,* Cell 2019) for the expression levels of ORAI1, 2 and 3 (as well as STIM1 and STIM2). The ORAI1, 2 and 3 expression levels however, only show very subtle differences between intestinal $CD4^+$ activated T cells (columns 1 and 2 in the T cell box) and Treg cells (column 4 in the T cell box) (**Reviewer Figure 5A**). Likewise, we observed by RT-PCR that murine Th1, Th17 and Treg express comparable levels of ORAI1-3 and STIM1-2 mRNA (**new Supplementary Figure 1**). With regard to the effects of BTP2 treatment on human B cells, we also found similar levels of ORAI1, 2 and 3, STIM1 and STIM2 mRNA expression in follicular B cells (characterized by high CD19, HLA-DR, and CCR7 expression similar to the $CD19^+$HLA-DR^+ we had identified by our CyTOF analyses) when compared to T cells (**Reviewer Figure 5A**). As suggested by the reviewer, we calculated ORAI3/ORAI1 and ORAI3/ORAI1 ratios for the different subclusters and observed comparable ratios for most of the cell clusters, including myeloid cells, NK cells, ILCs and T cells. Differences in ORAI1, 2 and 3 levels can therefore, in our opinion, not well explain why some of the cell clusters such as activated $CD4^+$ $CD45RO^+$ T cells respond more strongly to BTP2 treatment than $CD4^+$ $CD25^+$ Treg cells (**Fig.2E**), We added a short description of these findings together with the mentioned citation to the Results section on **page 13** and have included these data as **Extended View Figure 3.**

A

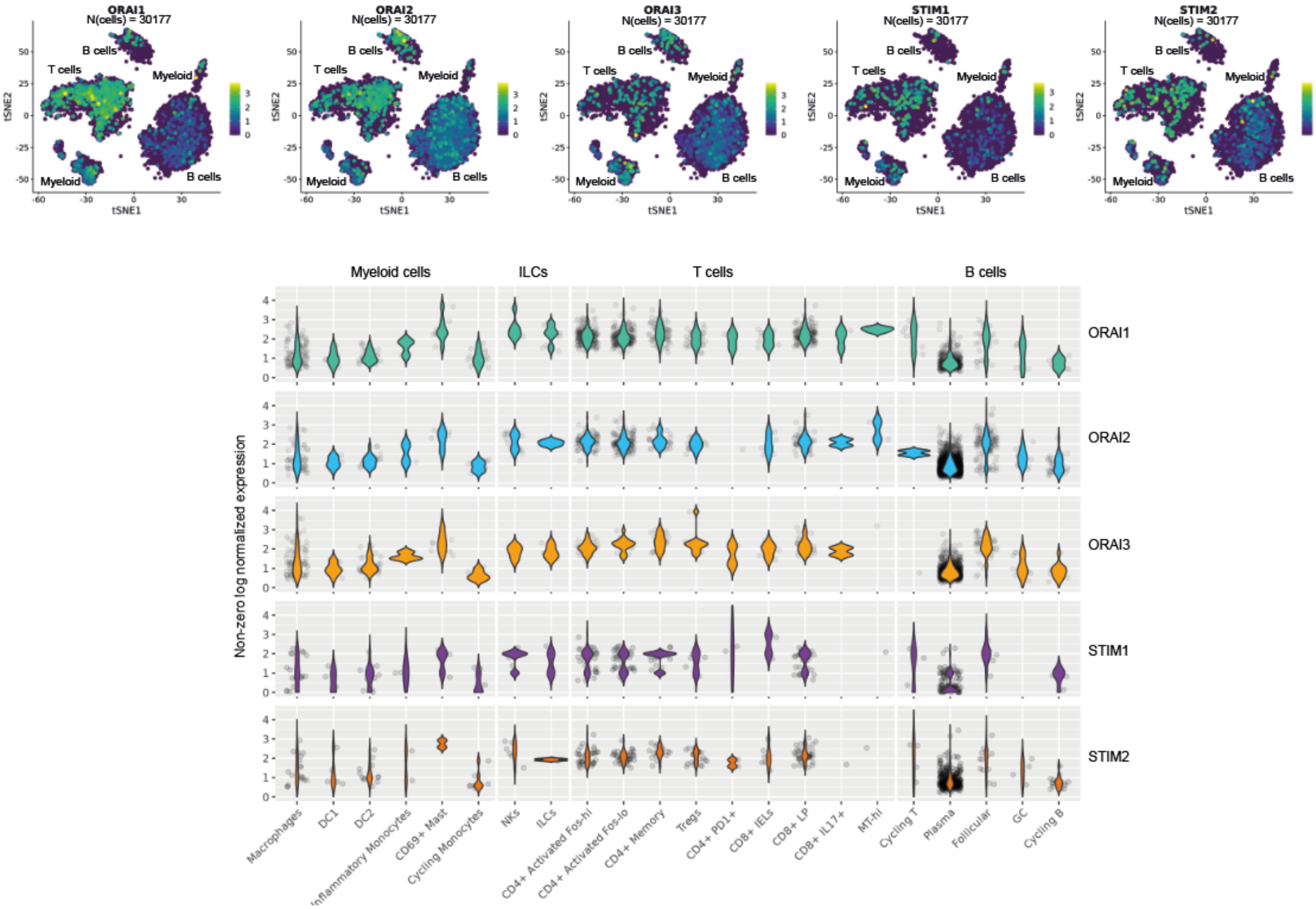

B

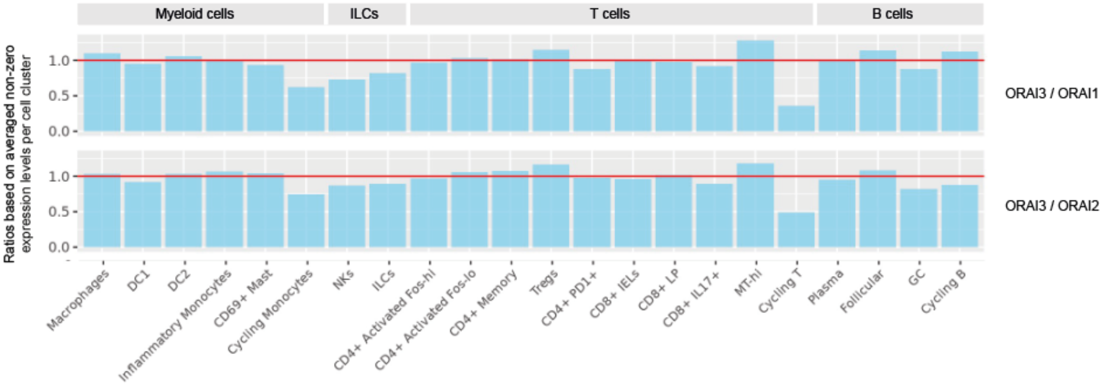

Reviewer Figure 5. mRNA expression of ORAI and STIM genes in lamina propria mononuclear cells (LPMC) of healthy donors. (**A**) ViSNE plots and violin plots showing mRNA expression levels of ORAI1, ORAI2, ORAI3, STIM1 and STIM2 in subsets of LPMC. (**B**) Bar graphs showing the ratios of average mRNA expression for ORAI3/ORAI1 and ORAI3/ORAI2 in different subsets of LPMC. Expression data in A and B were reanalyzed from Smilie et al. 2019, PMID: 31348891.

Why are some markers (CD25 in $CD4^+$ and $CD8^+$) upregulated upon BTP2 treatment, 'a cell type dependent threshold' probably does not explain why the effect goes in the opposite direction: An upregulation of markers is observed.

Response: We thank the reviewer for the interesting question. Inhibition of SOCE results in the suppression of many genes, especially cytokines, whose expression in most cases is positively regulated by SOCE as also shown in this study. For other genes, however, SOCE can either induce or repress expression, which we have shown in many previous papers using either patient T cells lacking SOCE (Feske et al. 2001, PMID 11276202, Fig.4) and T cells from knockout mice lacking SOCE (e.g. Kaufmann et al. 2019, PMID 30773462, Fig.2 and 3; or Kahlfuss et al. 2020, PMID 32609955, Fig.7 and 8). Moreover, we had previously shown that inhibition of SOCE by deletion of *Stim1* in T cells causes the upregulation of cytokine receptors IL-12R and IL-18R, and increased responsiveness of T cells to these cytokines (Desvignes et al. 2015, PMID 25938788). Some of these SOCE effects are likely mediated by Ca^{2+}-regulated transcriptional repressors such as DREAM (Carrion et al. 1999, PMID 10078534). It is therefore not surprising that inhibition of SOCE with BTP2 results in upregulation of some genes.

Fig. 7a: If biophysical properties of CRAC are unchanged, the current in DVF solution should be larger than in 20 mM Ca^{2+}. Can the authors comment on this? Please also display the outward currents analyzed at +80 mV and/or the IV during La^{3+} and DVF application.

Response: We thank the reviewer for this comment. In the example trace shown, the current in DVF solution is in fact larger than the Ca^{2+} current in 20 mM Ca^{2+}immediately prior to DVF solution application (it is >2 fold larger). However, in that experiment, DVF was applied after CM4620 and La^{3+} applications. From previous work, we know that blockade of CRAC current by both CM4620 and La^{3+} is difficult to reverse, and hence the DVF current is smaller than the original CRAC current collected prior to CM4620 and La^{3+} applications. In more recent unpublished work, we have also found that reversal of CRAC current by CM4620 is enhanced by repeated DVF solution applications, suggesting that the CM4620 block may be sensitive to the presence of extracellular Ca^{2+} chelators. We do not know the biophysical mechanisms of this effect, yet. However, because this issue is outside the scope of the current study, we have simplified the trace shown in the Figure and removed the DVF solution application. The **updated Figure 7A-C** now just shows inhibition of CRAC current by CM4620 in 20 mM Ca^{2+} without the DVF solution administration.

Referee #3

This study investigates the role of SOCE in the development and function of immune cells in IBD, and tests the therapeutic potential of a SOCE inhibitor in a mouse model of IBD. The authors examine the relationship of calcium signaling strength, T cell differentiation/polarization, function, and contribution to T cell transfer colitis using a collection of STIM/ORAI single and double mutant mice. This is then coupled with cytof-based analysis of development and calcium-dependent function of lamina propria immune cells from IBD patients. The authors conclude that SOCE regulates immune cell-driven pathology and identify SOCE inhibitors as potential therapeutic agent for IBD. In general the study is well written and synthesizes a large body of data clearly. There are however, some issues with experimental design and/or analysis requiring clarification in order to strengthen the study's conclusions.

Response: We thank the reviewer for the positive evaluation of our study and the critical and helpful comments, which we address one by one below.

Major points: 1. Figure 1 - SOCE has less effect of Tregs? Conditions used to evaluate Th1, Th17 is quite different from Treg. In the first case cells are subjected to two calcium-dependent activation events (differentiation on CD3/CD28 + cytokine cocktail followed by PMA/iono stim) vs one for Tregs (differentiation only). It seems that to directly compare the magnitude of effect of SOCE on T cell differentiation the assays should be comparable, ie looking at restimulated cytokine production in iTregs or relevant transcription factors pre-stim for Th1/Th17.

Response: We thank the reviewer for this comment. We agree that the readouts we had previously used to assess the effects of SOCE deletion on Th1, Th17 and Treg cells were inconsistent. Part of the reason was that Th1 and Th17 function is easy to measure (cytokines), but Treg function is more complicated to assess. To provide a better comparison of SOCE effects on Th1, Th17 and Treg cells, we have conducted new experiments in which we have measured the effects of SOCE inhibition on lineage-specific (i) transcription factors, and (ii) effector molecules in Th1, Th17 and Treg cells. To determine the effects of SOCE, we had to use the SOCE inhibitor BTP2 because we did not have all the mouse strains originally used in **Figure 1** available anymore. (Please note that BTP2 was also used for most of the CyTOF experiments shown in **Figures 2-5** and that it has comparable effects to another CRAC channel inhibitor, CM4620. We are therefore very confident that BTP2 is a specific inhibitor of SOCE and CRAC channels).

i) Effects of SOCE on T cell functional: Naïve $CD4^+$ T cells were differentiated into Th1 cells (in the presence of IL-12 and anti-IL-4 antibodies) and Th17 cells (IL-6, TGFβ, anti-IFNγ and anti-IL-4-antibodies) for 3 days in the presence of different concentrations of BTP2. T cells were restimulated with PMA/ionomycin for 4h, which resulted in progressively stronger inhibition of SOCE (**Reviewer Figure 6A,B**) and IFNγ production in Th1 cells and IL-17A in Th17 cells with higher concentrations of BTP2 (**Reviewer Figure 6C,D**). The IC_{50} of BTP2 inhibition was ~30 nM for IFNγ production by Th1 cells and ~ 25 nM for IL-17A production by Th17 cells. In parallel, we differentiated naïve $CD4^+$ T cells into iTreg cells (in the presence of TGFβ) for 3 days and analyzed the expression of the effector molecule CTLA-4, which suppresses the activation of effector T cells (Shevach 2009, PMID 19464986). We observed a biphasic expression of CTLA-4 in iTreg cells, i.e. upregulation at low concentrations of BTP2 and moderate downregulation at higher BTP2 concentrations (**Reviewer Figure 6C,D**). These results suggest that CTLA4 expression is not significantly reduced by SOCE inhibition. We were not able to directly measure the suppressive capacity of iTreg cells in co-culture experiments with effector T cells (Teff) because the presence of BTP2 in the in vitro co-culture system would inhibit both Treg and Teff cells. We have, however, previously published in two independent studies that the suppressive function of *Orai1*-deficient and *Stim1*-deficient Treg cells is preserved (Desvignes et al. 2015, PMID: 25938788. Suppl. Figure 6. Kaufmann et al. 2016, PMID 26673135. Figure 2). These data are reproduced in **Reviewer Figure 7**.

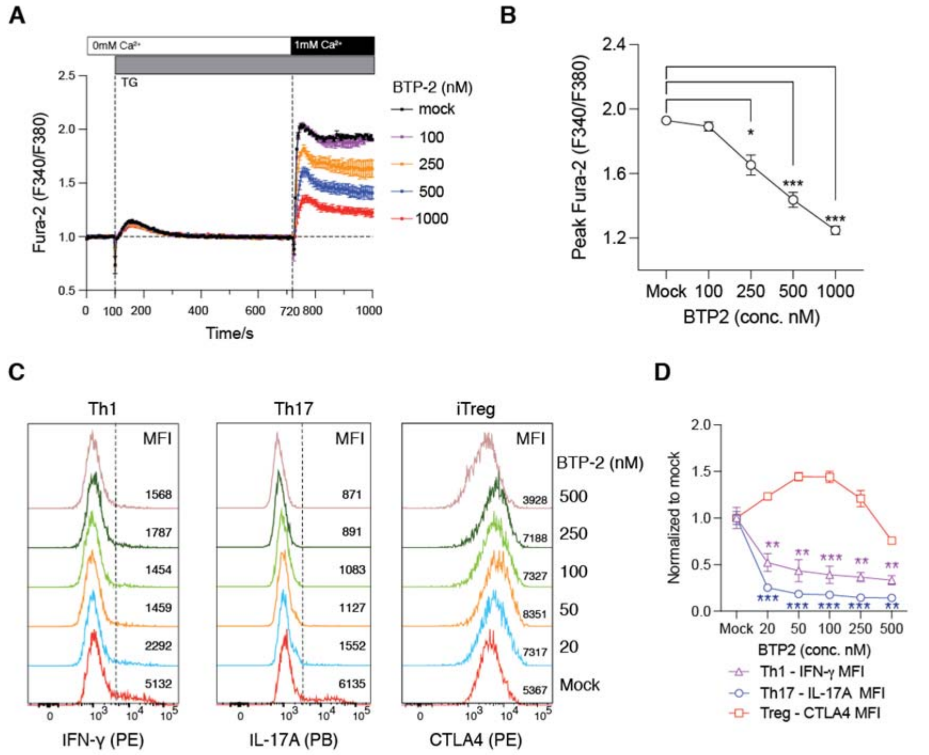

Reviewer Figure 6. Effects of the CRAC channel inhibitor BTP-2 on the function of murine Th1, Th17 and iTreg cells (**A**) Analysis of SOCE in $CD4^+$ T cells cultured *in vitro* for 3 days under Th0 condition; cells were then treated acutely with BTP-2 for 30 min before calcium measurement. SOCE (peak F340/380) was quantified in (**B**); shown are means±SEM of cells from two mice measured in triplicates. (**C**) Analysis of IFNγ, IL-17A and CTLA-4 expression in Th1, Th17 and iTreg cells with CRAC inhibitor BTP-2 treatment. Naïve T cells were cultured under Th1 and Th17 condition for 3 days in the presence of gradient BTP-2, cells were restimulated with PMA/Ionomycin for 4 hours to detect cytokine production by flow cytometry. CLTA4 in iTreg cells was detected by intracellular staining without PMA/Ionomycin stimulation. (**D**) Quantification of normalized MFI of IFNγ, IL-17A and CTLA-4 by Th1, Th17, iTreg respectively. Statistical analysis by unpaired student's t-test was performed either between Th1 and iTreg or Th17 and iTreg: ***p<0.001 **p<0.01 *p<0.05. (C-F) shown are means±SEM of data from three mice.

.

Figure for reviewers removed

(ii) Effects of SOCE on T cell differentiation: CD4$^+$ T cells were differentiated into Th1, Th17 and iTreg cells for 3 days in the presence of different concentrations of BTP2 as described above and analyzed for the expression of the lineage defining transcription factors T-bet, RORγt and Foxp3. Increasing concentrations of BTP2 resulted in a progressive decrease in the expression of RORγt in Th17-polarized cells and Foxp3 in iTreg-polarized cells with an IC_{50} of BTP2 inhibition of ~ 79 nM (Th17) and ~ 63 nM (iTreg) (**Reviewer Figure 8A,B**). Surprisingly, increasing concentrations of BTP2 resulted in a progressive enhancement of T-bet expression in Th1-polarized cells. We investigated the cause of this enhancement and found that BTP2 treatment results in increased expression of IL-12 receptor (IL-12R) (**Reviewer Figure 8C**). Because CD4$^+$ T cells are polarized into Th1 cells in the presence of IL-12 and because IL-12/IL-12R signaling is known to regulate T-bet expression (e.g. PMID: 16220539), BTP2 treatment makes T cells more responsive to T-bet expression and Th1 polarization. We had previously observed a similar up-regulation of IL-12Rb1 expression in T cells of a patient with ORAI1 loss-of-function mutation (Desvignes et al. 2015, PMID: 25938788, Suppl. Figure 2) (**Reviewer Figure 9**). These data are consistent with the known role of TCR signaling in suppressing IL-12R expression (Schulz et al. 2009, PMID: 19409816).

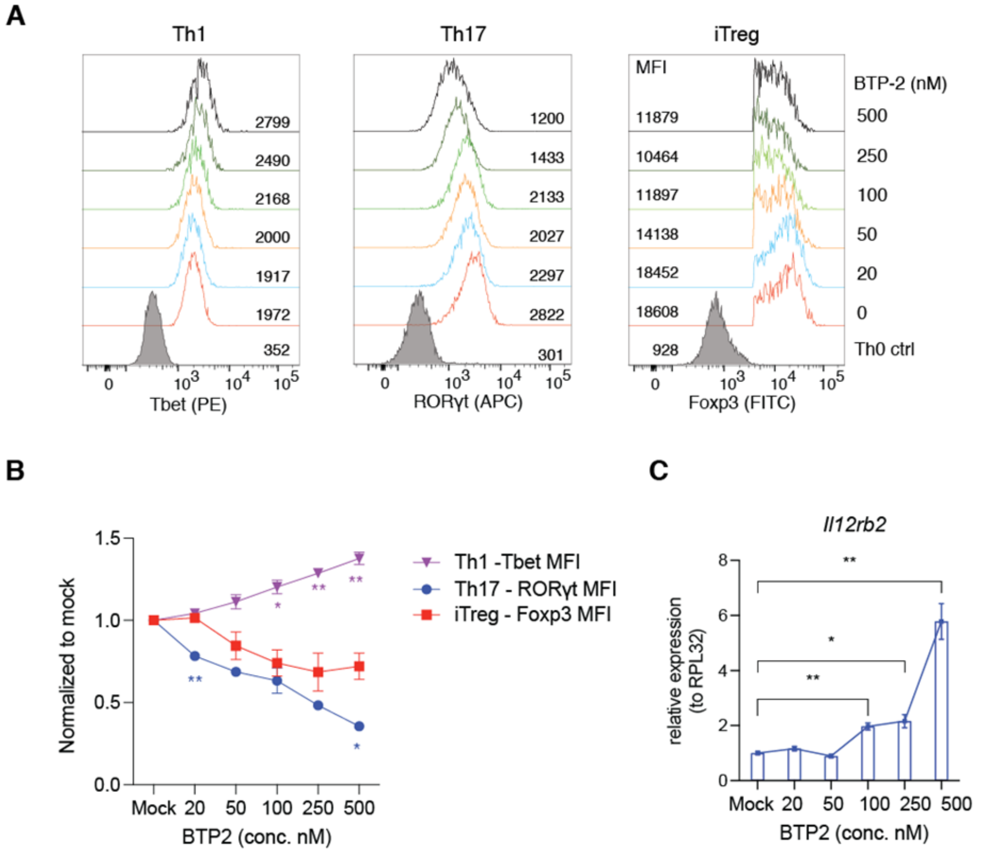

Reviewer Figure 8. Effects of BTP-2 on the differentiation of murine Th1, Th17 and iTreg cells. (A and B) Tbet, RORgt and Foxp3 levels on Th1, Th17 and iTreg in the presence of gradient BTP-2 treatment. Normalized mean fluorescence intensities (MFI) in (**A**) were quantified in (**B**). Statistical analysis by unpaired student's t-test was performed either between Th1 and iTreg or Th17 and iTreg: ***p<0.001 **p<0.01 *p<0.05. (**C**) IL12Rb2 mRNA levels were detected by qPCR in CD4$^+$ T cells under Th1 polarization condition for 24 hours with BTP-2 treatment. Statistical analysis by unpaired student's t-test: ***p<0.001 **p<0.01 *p<0.05. Data in A-B are the mean ± SEM of 3 mice per cohort.

From these new experiments and our previously published data we conclude that inhibition of SOCE (i) gradually suppresses the function of Th1 and Th17 cells (cytokine production) but not that of iTreg cells, and (ii) suppresses the differentiation of Th17 and iTreg cells, whereas T-bet expression is enhanced (at least under standard in vitro polarization conditions in the presence of exogenous IL-12). We have added some of these data as **New Supplementary Figure 4** to the manuscript and added a discussion of these findings.

Figure for reviewers removed

For Orai1f/fCD4Cre colitis transfer model - it was previously reported by the Feske lab that Orai1f/fCD4Cre T cells induce colitis equivalent to WT. Why the difference in phenotype here?

Response: We thank the reviewer for the opportunity to discuss the apparent difference between this study and Vaeth et al. 2017 (PMID 28294127) with regard to the colitis severity induced by the adoptive transfer of *Orai1*$^{fl/fl}$ *Cd4Cre* T cells. We think that slight differences in the experimental protocols used for these studies account for the differences. In this study, we adoptively transferred $5x10^5$ FACS-sorted $CD4^+$ $CD25^-$ $CD45RB^{high}$ $CD62L^+$ T cells, whereas in Vaeth et al., we transferred $5x10^5$ $CD4^+$ $CD25^-$ $CD62L^+$ T cells. In the current study, we transferred T cells from female donor mice into female *Rag1*$^{-/-}$ recipients, whereas in Vaeth et al. we performed both male to male and female to female transfers. Moreover, mice were housed in different animal facilities for this study and the experiments reported in Vaeth et al. Given the importance of the gut microbiome composition for colitis severity, it is likely that these differences account for the protection from weight loss reported here compared to no protection reported in Vaeth et al.

Weight loss, however, is not a good readout for IBD severity. When comparing the histological colitis scores of mice that had received *Orai1*-deficient T cells, we noted that *Orai1*$^{fl/fl}$ *CD4Cre* T cells induced less severe colitis in both studies, although this difference was moderate and did not reach statistical significance. (Please note that we had the histology slides of the current study re-analyzed by a GI pathologist in a blinded manner using a more detailed scoring system (1-17) instead of the more basic scoring system (1-5) shown in the previous version of the manuscript, new **Figure 1C**). Looking at the histology pictures in Figure 8C of Vaeth et al., please note for instance that the presence of goblet cells in recipient mice is preserved after transfer of *Orai1*$^{fl/fl}$ *CD4Cre* T cells compared to wildtype T cells, which is an important parameter of colitis severity. Moreover, *Orai1*$^{fl/fl}$ *CD4Cre* T cells also induced less colon shortening than wildtype T cells in Vaeth et al, which is another parameter of colitis severity. Overall, we think that the partial reduction of SOCE in *Orai1*$^{fl/fl}$ *CD4Cre* T cells is close to the threshold required for pathogenic T cell function in IBD and slight differences in experimental conditions may induce more or less severe colitis. In support of this interpretation is the fact that more profound suppression of SOCE in *Orai1*$^{fl/fl}$*Orai2*$^{fl/fl}$ *CD4Cre* T cells (Vaeth et al) or *Stim1*$^{fl/fl}$ *Cd4Cre* T cells (this study) results in almost complete protection from colitis.

After looking at the 2. Figure 2 - This figure examines the impact of SOCE inhibition on calcium flux and cytokine production in LP T cells from IBD patients. In A and B, cells are incubated overnight (16h) in BTP2 prior to calcium studies. Why were the cells exposed to the inhibitor for so long? It is also not clear whether the BTP2 is also present during the calcium assay or just the overnight incubation? It is important to distinguish between longer term effect of the inhibitor on cell survival/health, vs the acute effect of BTP2 on SOCE. For this experiment the acute effect of BTP2 on SOCE seems more relevant. The experiment performed to assess the effect of BTP2 on cell viability (suppl Fig 2), only looks at a 4h time point, relevant to the experiments in 2C-G, but not the calcium experiments in 2A-B.

Response: We thank the Reviewer for this valuable comment. As suggested, we have now examined the acute impact of BTP2 on SOCE inhibition in LP T cells from IBD patients after 4h of treatment in new additional experiments. As shown in **Reviewer Figure 10**. in-vitro exposure of CD4+ and CD8+ LPMCs to BTP2 for 4h causes a dose-dependent inhibition of SOCE and suppresses Ca^{2+} influx almost completely at a concentration of 1μM. For clarity reasons, we have now replaced the calcium traces shown in **Figure 2 A-B** to demonstrate the acute effects of BTP-2 treatment and to be consistent with our CyTOF experiments.

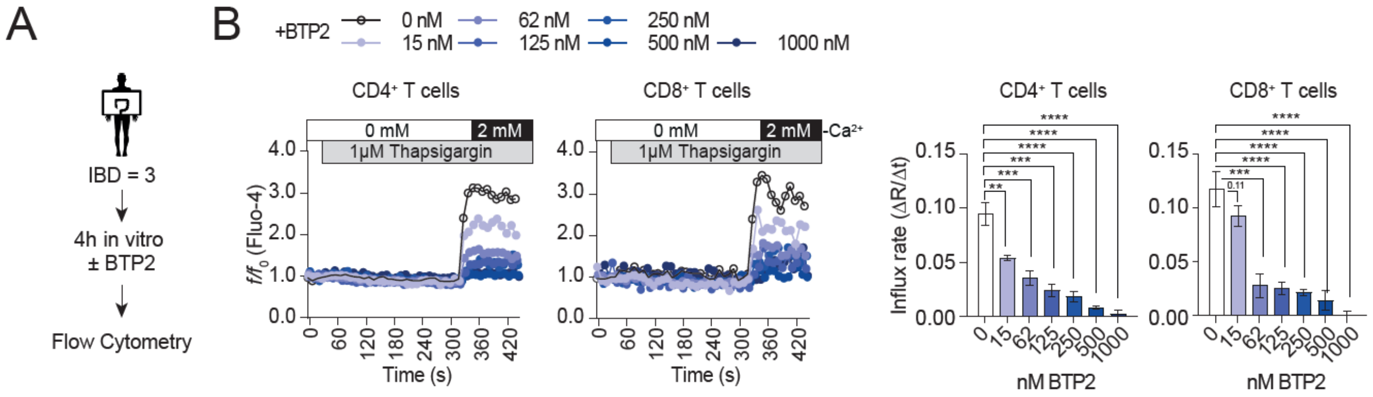

Reviewer Figure 10. Gradual inhibition of Store-operated Calcium Entry (SOCE) reduces Ca^{2+} influx rate of human lamina propria cells after 4h of treatment with BTP-2. (**A**) Experimental setup of Ca^{2+} influx assays in LPMCs (IBD: *n* = 3) by flow cytometry. (**B**) Traces show Ca^{2+} influx in human lamina propria CD4+ and CD8+ T cells (IBD: *n* = 3) following pre-incubation of cells with 15 - 1000 nM BTP2 for 4h until the time of data acquisition. Cells were stimulated with 1 μM thapsigargin (TG) in Ca^{2+} free buffer followed by the addition of 2mM Ca^{2+} Ringer buffer. Bar graphs indicate the mean Ca^{2+} Influx rate after the addition of 2 mM Ca^{2+} Ringer buffer. Error bars indicate standard error mean (SEM). Statistical significance was calculated using repeated measures (RM) one-way ANOVA test. ****p<0.0001 ***p<0.001 **p<0.01 *p<0.05.

In addition, we have also performed new additional calcium measurements in PBMCs of healthy donors comparing the effects of 4h treatment with 1μM BTP-2 and 1 μM CM4620 in T cells, NK cells, B cells and myeloid cells (Please see **Reviewer Figure 3** for details). These new data demonstrate that 4h treatment with CRAC-Channel inhibitors significantly suppresses SOCE in all cell subsets investigated. As mentioned above we have included these data into the new **Extended View Figure 4**.

The calcium influx and effect of the inhibitor is compared somewhat indirectly to PBMC's from healthy controls (in Suppl. Figure 1). It is actually quite notable, but not mentioned in the text, that the magnitude and rate of calcium influx is markedly higher in healthy PBMC's. Whether this is due to the difference in cell population (PBMCs) vs healthy/IBD host is unclear, but is important to understand if/how SOCE is dysregulated in IBD. In order to better understand the subsequent experiments comparing T cell subsets, cytokine production and the effect of SOCE inhibition in IBD vs non-inflamed patients it would be helpful to directly compare calcium flux, and the effect of the inhibitor, in IBD vs non-inflamed LP cells.

Response: We thank the reviewer for this interesting comment. PBMCs indeed have a higher magnitude and rate of Ca^{2+} influx than LPMCs. Increased Ca^{2+} influx in PBMC compared to LPMC could be a preparation artefact, or a real biological differences. The first explanation is supported by the fact that PBMCs are freshly isolated from peripheral blood, whereas LPMCs are isolated from patients undergoing colon resection. Unlike peripheral blood, resectates take longer from the operation room to reach the laboratory. In addition, LPMCs undergo a longer isolation procedure than PBMCs, including overnight digestion of colon tissue. The second explanation is supported by a study by Schwartz et al. (PMID 14585840), which had shown that Ca^{2+} signals in lamina propria T lymphocytes from non-inflamed tissue were reduced compared with Ca^{2+} signals of blood T lymphocytes. To respond to the reviewer's suggestion to compare Ca^{2+} influx of intestinal T cells of IBD-patients vs non-IBD, we analyzed Ca^{2+} influx rates in T cells isolated from the lamina propria of three IBD patients and two non-IBD controls (**Reviewer Figure 11**). We detected a higher Ca^{2+} influx in CD4+ and CD8+ T cells isolated from inflamed tissues of IBD patients compared to non-inflamed samples, suggesting an

association between SOCE levels and IBD. These results should, however, be interpreted with caution due to the low numbers of samples analyzed (which was due to the low cases of elective surgeries in our clinic during the winter months and last wave of the Covid19 pandemic). In general, colon resectates from individuals without inflammation/IBD are very scarce and we were unable to obtain more samples during the 3 months revision period.

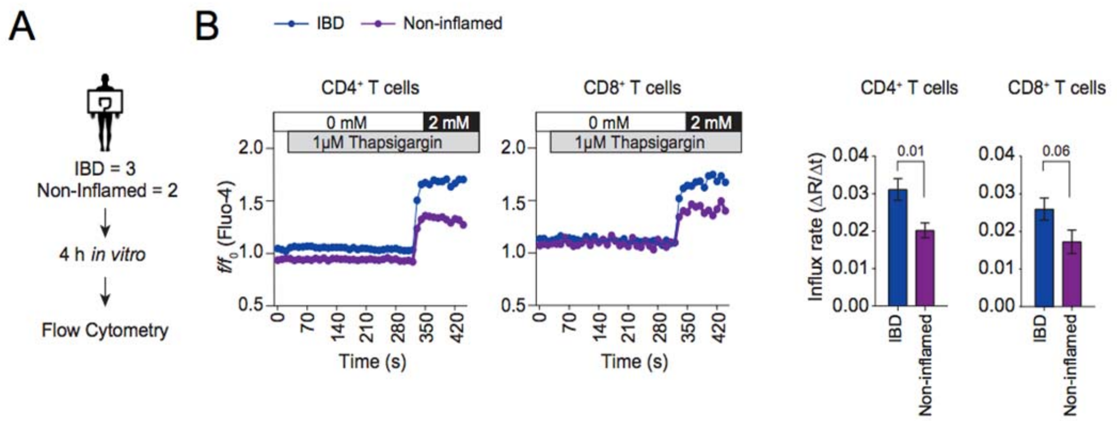

Reviewer Figure 11. Enhanced SOCE in IBD and increased expression of SOCE signature modules in IBD compared to Non-inflamed controls. (**A**) Experimental setup for Ca^{2+} influx assays on LPMCs (IBD: *n* = 3, Non-inflamed n= 2) by Flow cytometry. All samples have been acquired in quadruplicate. (**B**) Dot plots on the right show Ca^{2+} influx rates in human lamina propria $CD4^+$ and $CD8^+$ T cells kept 4 h in-vitro and stimulated with the sarco-endoplasmic reticulum Ca^{2+} ATPase (SERCA) inhibitor thapsigargin (TG) followed by the addition of 2mM Ca^{2+} ringer solution. Bar plots on the right indicate the mean Ca^{2+} Influx rate after the addition of 2mM Ca^{2+} ringer solution. Error bars indicate the standard error mean (SEM). Statistic was calculated with unpaired test and exact p-values are shown.

Supporting our observation of increased SOCE in LP T cells from IBD patients, Schwartz et al. (PMID 14585840), had previously reported that T cells isolated from inflamed mucosa of IBD patients have a higher Ca^{2+} signals compared to intestinal T cells isolated from non-inflamed mucosa. Likewise, Liang and colleagues have very recently reported that STIM1 protein expression is significantly higher in $CD45^+$ LPMCs isolated from inflamed mucosa of IBD patients, also supporting the concept that SOCE might be enhanced during IBD (Liang et al, CMGH 2022, https://doi.org/10.1016/j.jcmgh.2022.03.007).

To better understand how SOCE is regulated in LP T cells of IBD patients, we additionally determined the mRNA levels of *ORAI1, 2, 3 and STIM1, 2* in T cells isolated from IBD patients in comparison to healthy donors by reanalyzing publicly available scRNA data sets of LPMCs (*Smilie et al.* 2019, PMID: 31348891). As indicated in **Reviewer Figure 12**, ORAI1 and STIM1 mRNA levels were significantly higher in T cells of IBD patients. The fold-changes in expression, however, were small and it is unclear if they can explain the increase in SOCE. We have included a short description of the findings of Schwartz et al. and Liang et al. in the revised *Introduction* (page 5) and *Discussion (page 21)* and included the Ca^{2+} data from LP T cells of IBD and non-IBD patients as new **Supplementary Figure 11.**

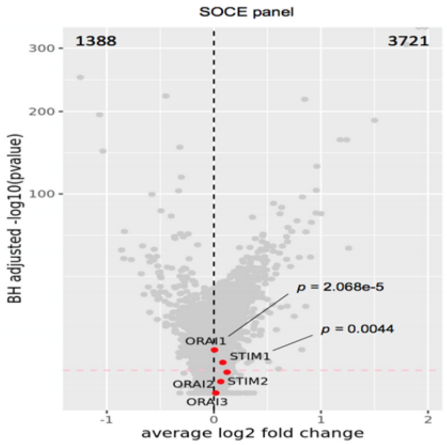

Reviewer Figure 12. Analysis of SOCE-signaling components in LP T cells of IBD patients vs non-inflamed healthy donors in publicly available scRNA data sets. Volcano plot showing LPMC-derived T cell specific gene expression changes comparing inflamed UC patients (n=7) with non-inflamed healthy donors (n=10). Differential expression analysis of genes was calculated using Seurat testing for significance applying the MAST hurdle model (sign. up-regulated in UC: 3721 genes; sign. down-regulated in UC: 1388). The SOCE signaling components ORAI1-3 are indicated in with red dots (ORAI1 p = 2.068e-5, log2 FC0.006) and STIM1-2 (STIM1 p=0.0044, log2 FC 0.084) were significantly upregulated in T cells of inflamed UC patients.

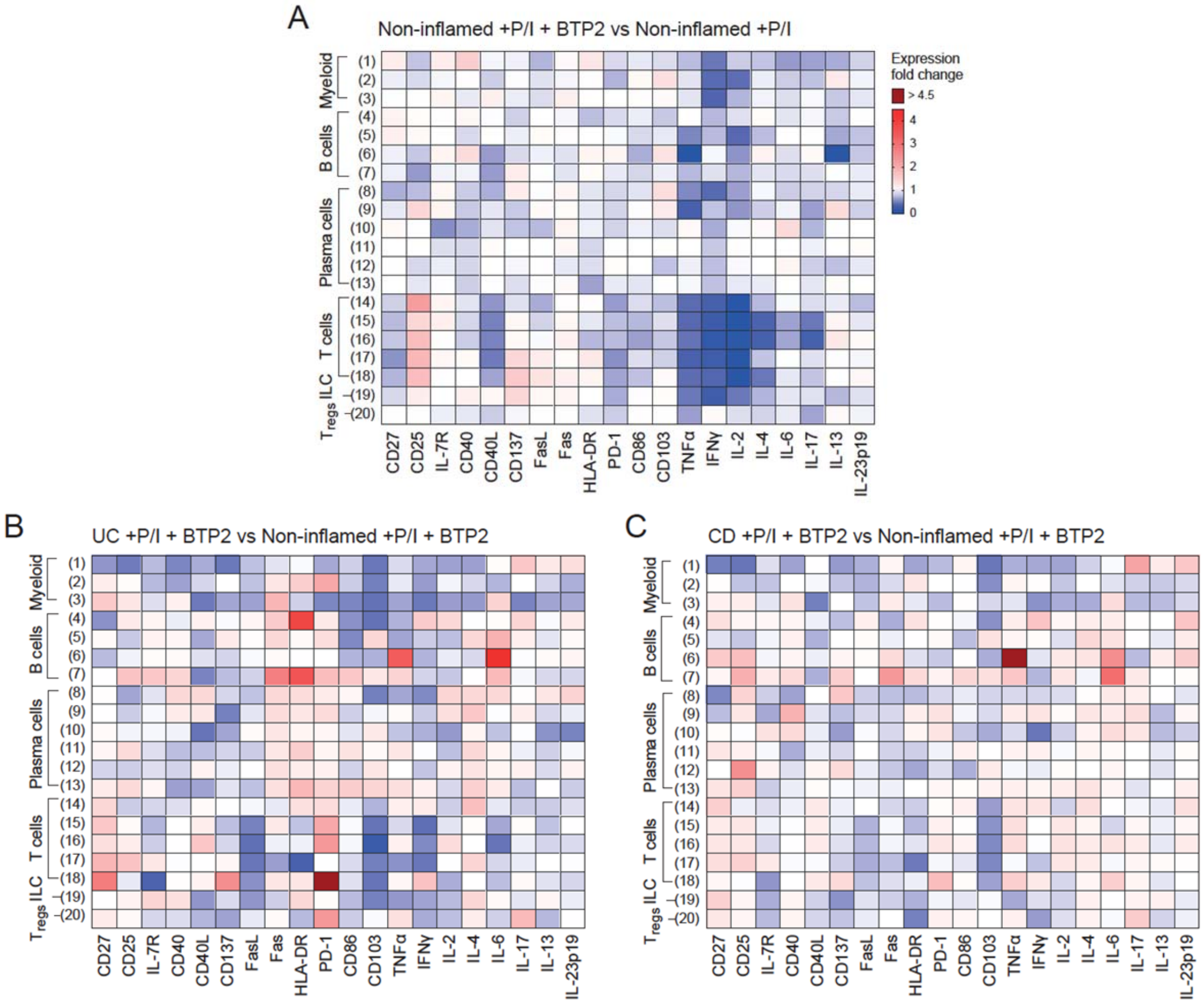

Reviewer Figure 13. Effects of BTP-2 on the function LPMCs from non-inflamed colon. (**A**) Heatmap representing the median fold change of cytokines and functional markers expression in $CD45^+$ LPMCs (HD: *n* = 4) activated 4 h *ex vivo* with PMA/ionomycin+ 1μM BTP2, normalized to samples treated with PMA/ionomycin alone. (**B+C**) Heatmap representing the median fold change of cytokines and functional markers in LPMCs of UC (*n* = 6) or CD patients (*n* = 6) treated with ionomycin/PMA+BTP-2 compared to LPMCs of non-inflamed controls (*n* = 4) treated with ionomycin/PMA+BTP2.

Regarding the reviewer's suggestion to compare the functional effects of BTP2 on T cells of IBD patients and non-IBD controls, we have performed additional analyses of our previous CyTOF experiments. As expected, because SOCE plays a pivotal role in T cells activation, we observed a downregulation of various proinflammatory cytokines in non-inflamed control specimens after BTP2 treatment (**Reviewer Figure 13A**). To directly compare the effects of BTP2 on IBD and non-inflamed samples, we also performed additional analyses comparing gene expression levels in LPMC from IBD patients (either CD or UC) and non-IBD controls after stimulation with P+I and treatment with BTP2 (**Reviewer Figure 13 B,C**). These comparisons revealed that BTP2 treated LPMCs of IBD patients still retained higher expression levels of several proinflammatory cytokines compared to non-OBD controls, for instance higher TNFα and IL-6 levels in B cells.

The data presented in **2E-G** only shows change in expression does not include the change in expression compared with unstimulated cells (except IL2 in 2D). Since direction of expression change with activation can be either up or down, and may differ between cell populations, it would be helpful to show this data (at least in the supplement), to best interpret the impact of BTP2.

Response: We thank the Reviewer for this comment. We have analyzed the effect of PMA/ionomycin treatment on $CD4^+$, $CD8^+$ T, $CD19^+HLA\text{-}DR^+$ and CD11c myeloid clusters compared to unstimulated cells as described in **Figure 2.** We included the results of this analysis as **new Extended View Figure 1** and as **Reviewer Figure 14** below.

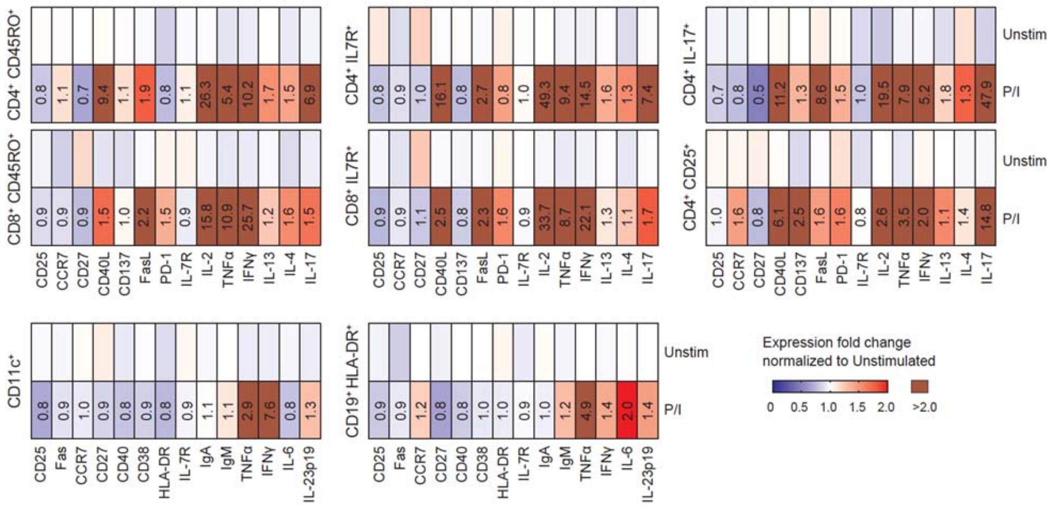

Reviewer Figure 14. Effects of PMA/ionomycin stimulation on protein expression of human lamina propria (LP) immune cells. Heatmaps representing the median fold change (FC) of cytokine and surface marker expression in $CD45^+CD3^+$ T cells (top row) and $CD45^+CD3^-$ immune cells (bottom row) isolated from colon lamina propria of five patients with Crohn's disease. Cells were treated with 20 ng/ml PMA and 1 µg/m (P/I) or DMSO (unstimulated) for 4h in vitro and protein expression was measured by mass cytometry (CyTOF). FC values are relative to unstimulated samples.

3. Figure 3. These experiments aim to compare the frequencies and functional status (as determined by stimulated cytokine production and activation markers) in IBD vs control LPMC. Comparison of frequency of cell populations based on lineage and activation markers is shown in 3B-E, however the data shown are after 4h restimulation with P/I. Since the goal is to "define the UC and CD-specific immune cell composition", it seems that the more relevant data to show here are the populations in the unstimulated condition since much can change during stimulation including activation markers, subpopulation-specific cell survival etc.

The basal cytokine signature would also be interesting to see, since these patients are presumably having a disease flare (necessitating bowel resection) and cytokine expression is likely already elevated in LPMC. How these markers and cytokine expression change with SOCE-dependent stimulation, and compare amongst non-inflamed, and IBD samples is a separate question, and also important.

Response: We thank the Reviewer for this thoughtful comment. We agree that an increase in "basal cytokine signature" in non-stimulated LPMC of IBD patients would be an interesting observation. We therefore compared the frequencies of cytokine producing LPMC in non-inflamed controls, UC and CD patients. However, as shown below in **Reviewer Figure 15**, we were unable to detect increases in basal levels of cytokines in unstimulated IBD or non-inflamed tissues by Mass cytometry. Therefore, we decided to focus on PMA/ionomycin-activated samples to analyze the properties of LP immune cell subsets in IBD vs non-IBD controls. Other technologies such as bulk or scRNA-sequencing of inflamed intestinal tissue may be better suited to study small increases in basal cytokine production and have in fact been used in several publications to identify the cytokine networks in IBD (e.g. Mitsialis et al. 2020, Gastroenterology, PMID: 324285070; Smilie et al. 2019, PMID: 31348891; Friedrich et al. 2021,Nature Medicine PMID: 34675383).

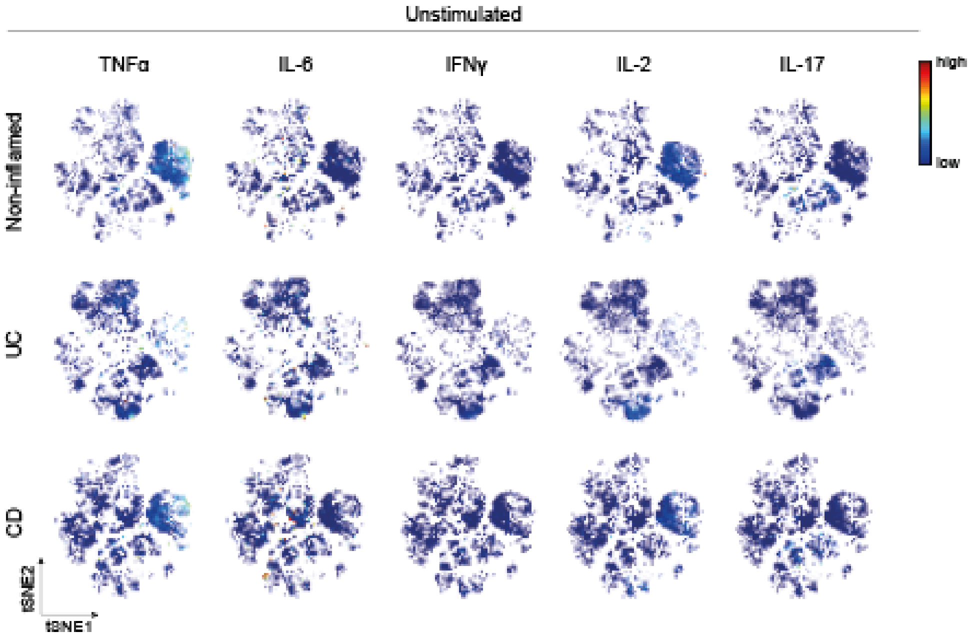

Reviewer Figure 15. Missing Cytokine production without ex vivo stimulation with ionomycin/PMA viSNE plots of one exemplary *ex-vivo* unstimulated Non-inflamed control, UC and CD patient colored by cytokine expression levels (blue: low, red: high).

To illustrate this point, we show in **Reviewer Figure 16** preliminary data from a collaborative research effort together with the IBD center in Erlangen, Germany, which aims at defining the molecular and cellular characteristics associated with therapy refractory disease in IBD patients using bulk RNA-sequencing of colon tissue and CyTOF analyses of PBMC of IBD patients. Preliminary analyses of RNA-sequencing data of colon resectates from a cohort of 61 patients show elevated mRNA levels of several pro-inflammatory cytokines such as IL17A, TNFα and IL-2 in UC and CD patients compared to non-IBD controls (**Reviewer Figure 16**). Since the molecular characterization of our cohort is ongoing and these results are unpublished, these data can unfortunately only be shown as Reviewer Figure at this point in time. We hope, however, that the data shown in Reviewer Figures 15 and 16 answer the referee's question.

Figure for reviewers removed

In the text top of p10 - "with a particular involvement of $CD38^+$ plasma cells producing IgA and IgM in CD not UC patients". Is this statement correct? It appears in the figure that 38+ IgA or IgM+ cells are altered in UC, not CD, compared to uninflamed.

Response: We thank the reviewer for his/her careful treading of our manuscript. We have corrected the sentence accordingly in the revised manuscript.

4. Figure 4 and 5. Related to the comment on figure 3, fully interpreting the effect of BTP2 on stimulated cytokine production requires understanding how these markers changed from unstimulated to stimulated conditions.

Response: We thank the Reviewer for this comment. We agree that to fully interpret the effect of SOCE inhibition on cytokine production and markers expression of samples treated with PMA/ionomycin + BTP2, we first must understand how the basal stimulation by PMA/ionomycin alone affects LPMCs. Therefore, we have included **Reviewer Figure 14** above**,** showing the global effect of PMA/ionomycin treatment on human lamina

propria cells. In addition, we have repeated the analysis of PMA/ionomycin effects on lamina propria immune cells subsets in an additional dataset of 6 IBD patients displayed in **Reviewer Figure 19** below**.** We hope that these data are sufficiently supporting the Reviewer's concern. Regrettably, we cannot show a detailed analysis of the effect of PMA/ionomycin stimulation on $CD45^+CD3^+$ and $CD45^+CD3^-$ LPMCs subsets, respectively described in **Figures 4 and 5**, because when taking into account cytokines and activation markers expression in the viSNE algorithm, the resulting cell clusters deriving from unstimulated or stimulated samples spatially separate in independent cell subsets. Therefore it is not possible to extract the unstimulated expression levels from clusters defined by cytokines (**Reviewer Figure 17**).

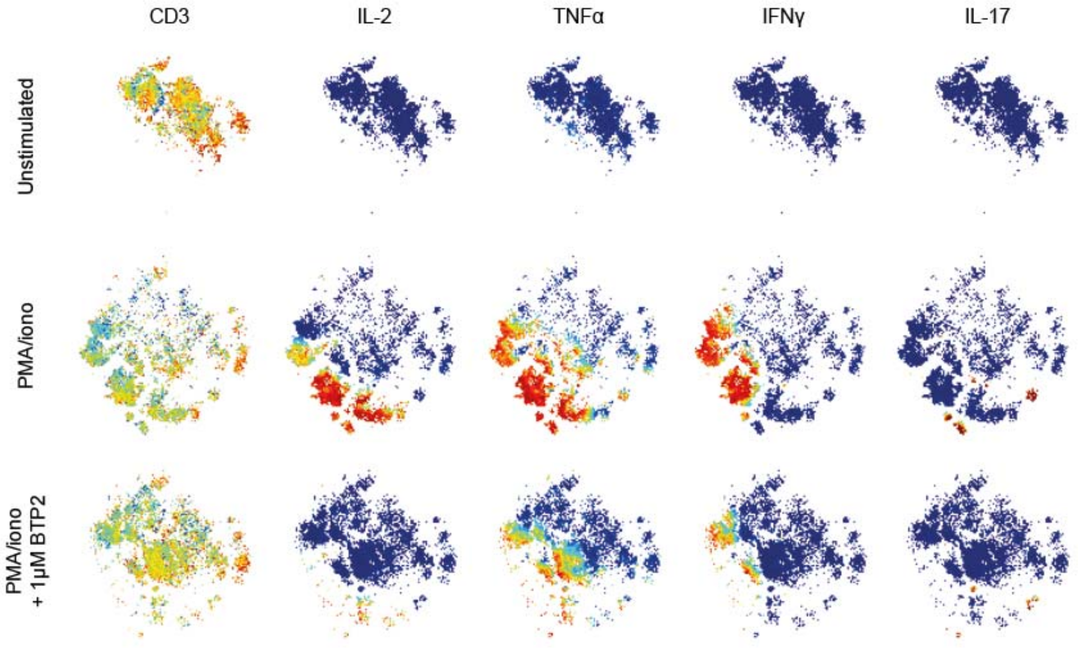

Reviewer Figure 17 viSNE plots of $CD45^+CD3^+$ LPMCs stimulated 4h with PMA/ionomycin in the presence of 1 μM BTP2, with un-stimulated samples serving as controls. viSNE plots are colored according to the expression level of cytokines (blue: low, red: high) and are representative of one CD patient.

Also not shown is whether BTP2 differentially impacts activation marker/cytokine expression from non-inflamed compared with IBD LPMCs. This would be important to know from a therapeutic perspective, particularly if considering longer term therapy - will SOCE inhibition inhibit function of healthy cells more or less than those in the inflammatory environment?

Response: We thank the reviewer for raising such an important point. As highlighted above we have compared the effects of BTP2 treatment on LPMCs of non-inflamed and inflamed tissue in **Reviewer Figure 14**. While SOCE inhibition may also affect the function of "healthy cells", we have not observed obvious adverse affects in mice treated with the SOCE inhibitor CM4620 for 30 days (**Figure 7D-J**). At the same time, IBD severity was significantly ameliorated. As mentioned in the Discussion section, CM4620 is currently being tested in clinical trials for its therapeutic effects on acute pancreatitis (NCT04195347) and COVID-19 pneumonia (NCT04345614). These studies suggest that SOCE inhibition is both well tolerated and effective in patients with severe pancreatic and pulmonary inflammation. Therefore, we do believe that despite potential side effects, SOCE inhibition is worth exploring further for its therapeutic effects in IBD. However, additional studies are required to investigate how the long-term treatment of patients with CRAC-channel inhibitors affects immunity against infections and tumors in patients and whether it would be safe for patients to continue SOCE-inhibitors after reaching clinical remission. We have included a paragraph addressing potential side effects of a chronic inhibition of CRAC-channels in the revised ***Discussion*** section on **page 22**.

5. Figures 7. Experiments with a different SOCE inhibitor demonstrate the ability of a CRAC channel inhibitor to improve disease progression in a mouse model. Although the data with CM4620 is convincing, to connect

these experiments with the rest of the study it would be better to include at least some direct comparison of BTP2 and CM4620, and data showing the impact of CM4620 on primary T cell SOCE or function since not all inhibitors function identically and may have different activity depending on the ORAI isoform combination in each cell type.

Response: We thank the Reviewer for this suggestion. We have performed additional experiments comparing the effects of BTP-2 and CM4620. As shown in **Reviewer Figure 18**, LPMCs from surgical colon resections of IBD patients were isolated and Ca^{2+} influx was assessed by flow cytometry after 4 h *ex vivo* incubation with two concentrations (250 and 1000nM) of the CRAC channel inhibitors BTP2 and CM4620 (**Reviewer Figure 18A**). Treatment with CM4620 caused a dose-dependent inhibition of SOCE in lamina propria $CD4^+$ and $CD8^+$ T cells similarly to BTP2 (**Reviewer Figure 18B**). Moreover, as shown above in **Reviewer Figure 3**, 1 µM CM4620 and 1 µM BTP2 both suppressed Ca^{2+} influx in $CD4^+$ and $CD8^+$ T cells, $CD19^+$ B cells, $CD56^+$ NK cells and $CD14^+$ monocytes. These findings indicate that BTP2 and CM4620 are potent inhibitors of SOCE.

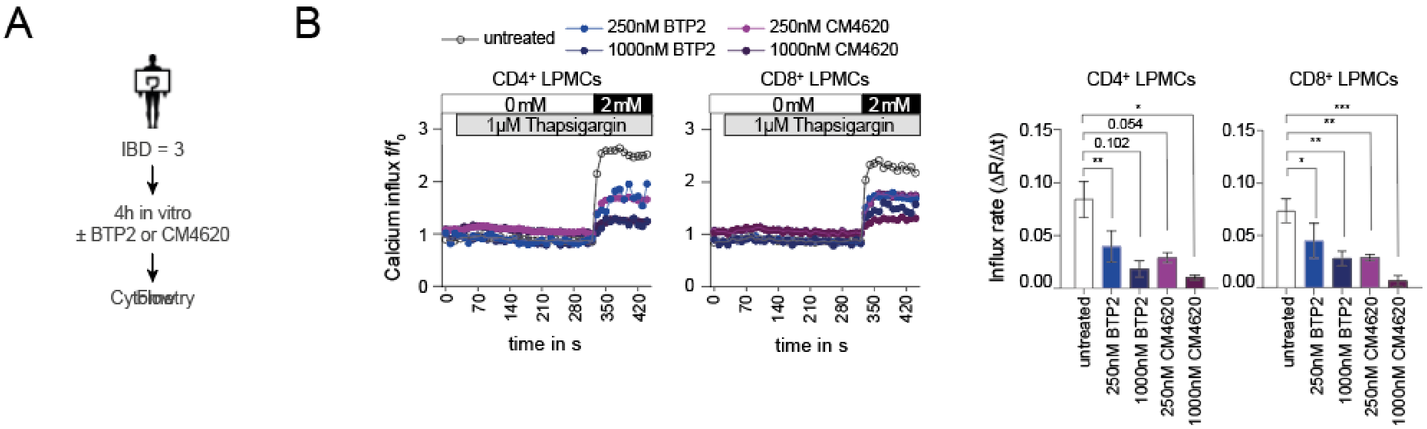

Reviewer Figure 18. CM4620 inhibits SOCE equally potent as BTP2 in LPMCs. (**A**) Experimental setup for Ca^{2+} influx measurements of LPMCs from 3 IBD patients by flow cytometry. (B) Human lamina propria $CD4^+$ and $CD8^+$ T cells were pretreated with 250 nM or 1000 nM BTP2 or CM4620 for 4 h in vitro and added to the cell suspension until acquisition. Cells were stimulated with 1 µM thapsigargin (TG) in Ca^{2+} free Ringer solution followed by the readdition of 2 mM Ca^{2+} Ringer solution. Bar graphs show the mean ± SEM of Ca^{2+} influx rates after the addition of 2 mM Ca^{2+} Ringer solution. Statistical significance was calculated with repeated measures (RM) one-way ANOVA test. ****p<0.0001; ***p<0.001; **p<0.01; *p<0.05.

To compare the effects of BTP2 or CM4620 on the function of immune cell subsets (including T, B, NK and myeloid cells), we analyzed the expression of cytokines and activation markers by LPMCs stimulated with PMA/ionomycin for 4 h and samples treated with either BTP2 (250 nM or 1000 nM) or CM4620 (250 nM or 1000 nM) by mass cytometry (**Reviewer Figure 19A**). As expected, stimulation of LPMCs with PMA/ionomycin resulted in an increased expression of cytokines such as IL-2, IL-17, IFNγ and TNFα in $CD45^+CD3^+$ LPMCs (**Reviewer Figure 19 B,C**), which were gradually inhibited by the addition of BTP2 or CM4620 (**Reviewer Figure 19D**). Importantly, the dose-dependent effects of BTP2 and CM4620 on gene expression were comparable. The Ca^{2+} influx data shown in **Reviewer Figure 18** and the new CyTOF data shown in **Reviewer Figure 19** have been included into the manuscript as new **Extended View Figure 5.**

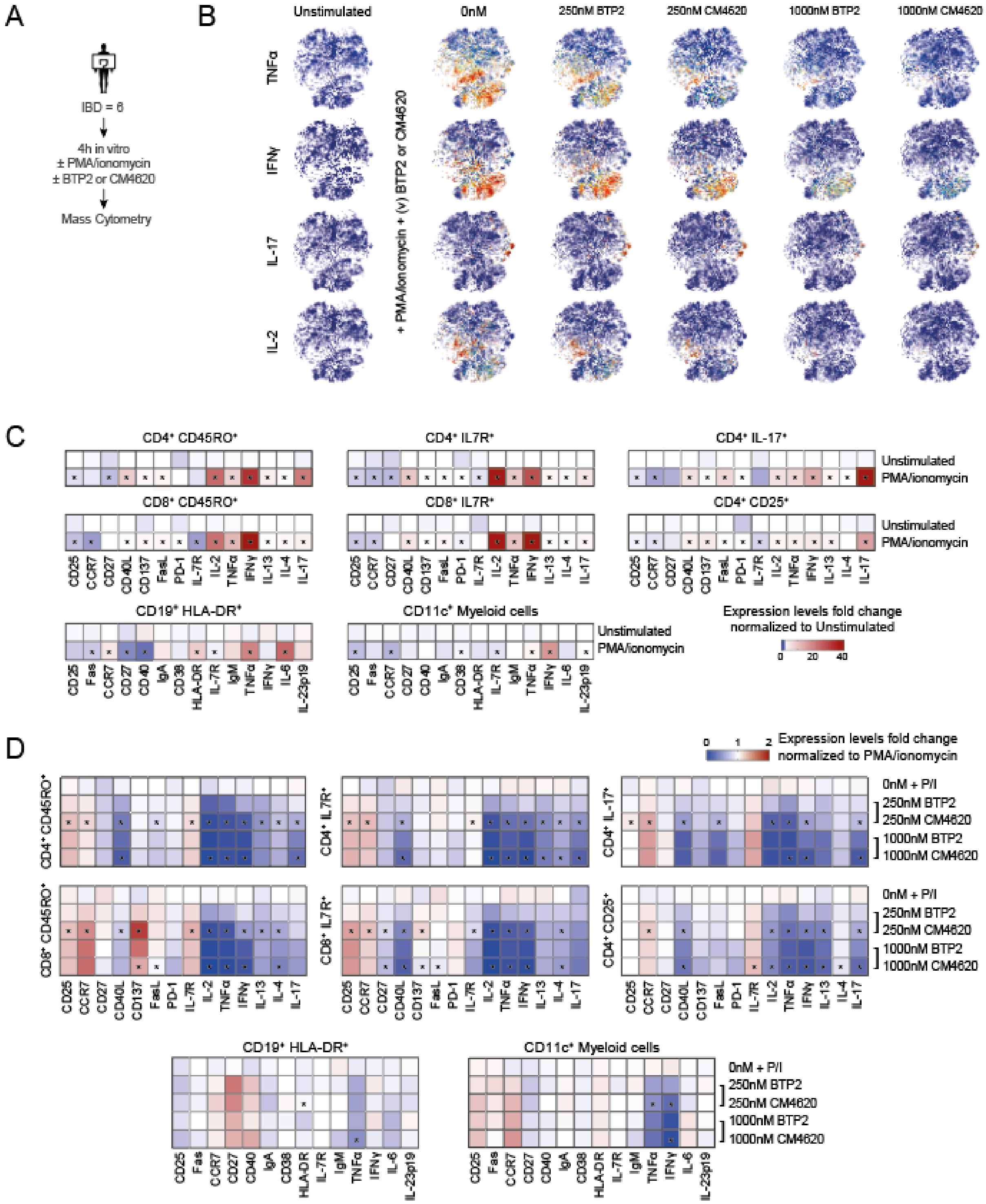

Reviewer Figure 19. Functional suppression of cytokine production by CM4620 in LPMCs of IBD patients. Mass cytometry analysis of LPMCs from 6 IBD patients. (**A**) Experimental design. (**B**) viSNE plots of $CD45^+CD3^+$ LPMCs stimulated with PMA/ionomycin in the presence of 250 or 1000 nM BTP2 or CM4620 with unstimulated samples as controls. Colors indicate expression level of IL-2, IL-17, IFNγ and TNFα (blue: low, red: high) and are representative of one CD patient. (**C**) Heatmaps representing the median fold change of cytokines and cell surface molecules on $CD45^+CD3^+$ or $CD45^+CD3^-$ LPMCs that were stimulated with PMA/ionomycin for 4 h in vitro. Data are normalized to unstimulated samples. (**D**) Heatmaps representing the median fold change of cytokine and cell surface marker expression in $CD45^+CD3^+$ and $CD45^+CD3^-$ LPMCs stimulated with PMA/ionomycin for 4 h in the presence or absence of 250 nM or 1000 nM BTP2 or 250 nM or 1000 nM CM4620. Data were normalized to samples treated with PMA/ionomycin alone. Statistical significance in **C, D** was calculated using a one-tailed paired Wilcoxon matched-pairs signed rank test. *$p < 0.05$.

In **Figure 7J** it is not clear whether CM4620 is also used during the P/I restimulation. If not, what is the proposed mechanism for the decreased cytokine production in the extracted cells? Altered activation or priming state due to direct effect of the inhibitor or environment?

Response: We apologize for not specifying the stimulation conditions in the legend to **Figure 7J,** which we have corrected in the revised ***Figure legend*** of **Figure 7J** on page 45 of the manuscript. $CD4^+$ T cells from the CLP of mice were stimulated in the absence of CM4620. Regarding the question " what is the proposed mechanism for the decreased cytokine production in the extracted cells", we speculate that CM4620 has a slow off-rate from its target ORAI1. We have not measured the precise off-rate of CM4620, but based on electrophysiology data it appears to be relatively long, similar to BTP2. We observed only a slight recovery of CRAC channel block over 15 minutes of washing out the compound. In patients with acute pancreatitis, the effects of a single infusion of CM4620 in an *ex vivo* whole blood assay of CRAC channel dependent IL-2 release, the inhibitory effects of CM4620 reversed 24-48 hours after the end of the infusion, indicating that the drug effect lingers for several hours after infusion. It is likely in our opinion that CM4620 stays engaged with ORAI1 and inhibits SOCE in the extracted cells.

Minor Points:

- Figure 2G. Text says CD11c + myeloid cells, but not clear from the figure or figure legend which myeloid population/s is shown.

Response: We apologize for the misleading description. The text is referring to cell cluster 3 of $CD45^+CD3^-$ LPMCs as indicated in the viSNE plots and heatmaps in **Supplementary Figure 5** in the Appendix. We have included an according explanation on page 8 of the revised results section.

In the text p. 7 "alternating requirements of cell-specific thresholds of SOCE signaling strength". It is unclear to me what "alternating requirements" refers to.

Response: We have replaced this sentence with a more detailed description of the different Ca^{2+} thresholds for T17 vs Treg function on **page 8** of the revised *Results* section of the manuscript.

20th Jun 2022

Dear Dr. Weidinger,

Thank you for the submission of your manuscript to EMBO Molecular Medicine, and please accept my apologies for the delay in getting back to you, as one referee needed more time to complete his/her report and I was then away for a meeting. We have now received the three enclosed reports. As you will see, the referees are supportive of publication, and I am therefore pleased to inform you that we will be able to accept your manuscript once the following editorial points will be addressed:

1/ Main manuscript text:
- Please address the queries from our data editors in track changes mode in the main manuscript file labelled 'Data edited MS file'. Kindly use this file for any further modification, remove the highlighted text and only keep in track changes mode any new modification.
- Please note that we can accommodate a maximum of 5 keywords (15 keywords currently on page 1).
- The Material and Methods section should be after the Discussion.
o Please include a statement that the experiments conformed to the principles set out in the WMA Declaration of Helsinki and the Department of Health and Human Services Belmont Report.
o Please indicate the dilutions/concentrations used for the antibodies.
o Please specify the origin of the mice and their housing conditions (access to food, light-night cycle).
- Acknowledgements: please update the submission system according to the information included in this section (TRR167 B05, Irma T. Hirschl career scientist award, Clinician Scientist Program of the Berlin Institute of Health and by the Fritz-Thyssen-Foundation (10.19.2.028MN) are missing).
- Author contributions: either Kenneth Stauderman or Katherine Sun is missing. Please note that CRediT has replaced the traditional author contributions section because it offers a systematic machine-readable author contributions format that allows for more effective research assessment. You are encouraged to use the free text boxes beneath each contributing author's name to add specific details on the author's contribution. More information is available in our guide to authors.
- Please rename the COI section: We updated our journal's competing interests policy in January 2022 and request authors to consider both actual and perceived competing interests. Please review the policy https://www.embopress.org/competing-interests and update your competing interests if necessary.
- Data Availability section: Please remove the first sentence.
- The references should be listed alphabetically, with 10 authors before et al.

2/ Figures and Appendix:
- Please upload your figures individually as .eps, .tif, .jpg (one file per figure).
- Please indicate in the legends or in the figures (including appendix figures) the exact p values, not a range. Some people found that to keep the figures clear, providing a supplemental table with all exact p-values was preferable. You are welcome to do this if you want to.
- Figure callouts in the text: please change Fig 1E to Fig 1D and Fig 1F to Fig 1E; a reference is missing for Fig 6F.
- EV Figure nomenclature needs to be corrected: please change to Expanded View Figures in the legends, and Figure EV1 etc when figures are referenced in the manuscript.
- Appendix: kindly include a table of content and correct the nomenclature to Appendix Figure S1, etc.

3/ Source Data: Thank you for providing Source Data. Please upload them to have 1 file per figure.

4/ Synopsis: Thank you for providing a nice synopsis figure. Please upload it as a PNG/TIFF/JPEG file 550 px wide x 300-600 px high and make sure that the text remains legible.
I slightly shortened your synopsis text to make it fit with our format, please let me know if you agree with the following:

The immune cell composition, signaling cascades and cytokine networks controlling inflammation in therapy-refractory inflammatory bowel diseases (IBD) remain incompletely understood.
• The colon lamina propria (LP) of ulcerative colitis (UC) and Crohn's disease (CD) patients is enriched with CD4+ and CD8+ T cells, IL-17-producing innate immune cells (ILC) and Treg cells.
• Pharmacological inhibition of store-operated Ca^{2+} Entry (SOCE) inhibits the production of proinflammatory cytokines and certain activation markers by human LP T cells, B cells, ILCs and myeloid cells.
• Inhibition of SOCE does not impair the differentiation and function of human or mouse intestinal epithelial cells in colonic organoid cultures.
• Pharmacologic inhibition of SOCE or T cell specific deletion of the SOCE genes Orai1 and Stim1 in T cells ameliorates intestinal inflammation in mouse models of colitis.
• SOCE is an important regulator of intestinal immune cell function and a potential drug target for the treatment of IBD.

5/ As part of the EMBO Publications transparent editorial process initiative (see our Editorial at

http://embomolmed.embopress.org/content/2/9/329), EMBO Molecular Medicine will publish online a Review Process File (RPF) to accompany accepted manuscripts.
This file will be published in conjunction with your paper and will include the anonymous referee reports, your point-by-point response and all pertinent correspondence relating to the manuscript. Let us know whether you agree with the publication of the RPF and as here, if you want to remove or not any figures from it prior to publication.
Please note that the Authors checklist will be published at the end of the RPF.

I look forward to receiving your revised manuscript.

Yours sincerely,

Lise Roth

Lise Roth, PhD
Senior Editor
EMBO Molecular Medicine

***** Reviewer's comments *****

Referee #1 (Comments on Novelty/Model System for Author):

Use of human subjects in critical for these studies

Referee #1 (Remarks for Author):

The authors have done well in addressing most of the comments raised by this reviewer, but maybe in future studies they might use adaptive transfer using mouse models to see if they can recapitulate the human data as well as establish the timeline.However, this is not a critic, rather a suggestion for future studies.

Referee #2 (Comments on Novelty/Model System for Author):

The Authors answered all my points to my full satisfaction. This is a study of extremely high value and I strongly recommend publishing it!

Referee #2 (Remarks for Author):

Great work!

Referee #3 (Remarks for Author):

The authors have very thoroughly addressed all concerns.

The authors performed the requested editorial changes.

5th Jul 2022

Dear Dr. Weidinger,

Thank you for submitting the revised files. I am pleased to inform you that your manuscript is accepted for publication and is now being sent to our publisher to be included in the next available issue of EMBO Molecular Medicine!

Please read below for additional IMPORTANT information regarding your article, its publication and the production process.

Congratulations on your interesting work,

With kind regards,

Lise Roth

Lise Roth, Ph.D
Senior Editor
EMBO Molecular Medicine

Follow us on Twitter @EmboMolMed

EMBO Press Author Checklist

Corresponding Author Name: Stefan Feske & Carl Weidinger	
Journal Submitted to: Embo Mol Med	
Manuscript Number: EMM-2022-15687	

USEFUL LINKS FOR COMPLETING THIS FORM
The EMBO Journal - Author Guidelines
EMBO Reports - Author Guidelines
Molecular Systems Biology - Author Guidelines
EMBO Molecular Medicine - Author Guidelines

Reporting Checklist for Life Science Articles (updated January 2022)
This checklist is adapted from Materials Design Analysis Reporting (MDAR) Checklist for Authors. MDAR establishes a minimum set of requirements in transparent reporting in the life sciences (see Statement of Task: 10.31222/osf.io/9sm4x). Please follow the journal's guidelines in preparing your manuscript.
Please note that a copy of this checklist will be published alongside your article.

Abridged guidelines for figures
1. Data
The data shown in figures should satisfy the following conditions:
- the data were obtained and processed according to the field's best practice and are presented to reflect the results of the experiments in an accurate and unbiased manner.
- ideally, figure panels should include only measurements that are directly comparable to each other and obtained with the same assay.
- plots include clearly labeled error bars for independent experiments and sample sizes. Unless justified, error bars should not be shown for technical replicates.
- if n<5, the individual data points from each experiment should be plotted. Any statistical test employed should be justified.
- Source Data should be included to report the data underlying figures according to the guidelines set out in the authorship guidelines on Data Presentation.

2. Captions
Each figure caption should contain the following information, for each panel where they are relevant:
- a specification of the experimental system investigated (eg cell line, species name).
- the assay(s) and method(s) used to carry out the reported observations and measurements.
- an explicit mention of the biological and chemical entity(ies) that are being measured.
- an explicit mention of the biological and chemical entity(ies) that are altered/varied/perturbed in a controlled manner.
- the exact sample size (n) for each experimental group/condition, given as a number, not a range;
- a description of the sample collection allowing the reader to understand whether the samples represent technical or biological replicates (including how many animals, litters, cultures, etc.).
- a statement of how many times the experiment shown was independently replicated in the laboratory.
- definitions of statistical methods and measures:
  - common tests, such as t-test (please specify whether paired vs. unpaired), simple χ2 tests, Wilcoxon and Mann-Whitney tests, can be unambiguously identified by name only, but more complex techniques should be described in the methods section;
  - are tests one-sided or two-sided?
  - are there adjustments for multiple comparisons?
  - exact statistical test results, e.g., P values = x but not P values < x;
  - definition of 'center values' as median or average;
  - definition of error bars as s.d. or s.e.m.

Please complete ALL of the questions below.
Select "Not Applicable" only when the requested information is not relevant for your study.

Materials

Newly Created Materials	Information included in the manuscript?	In which section is the information available? (Reagents and Tools Table, Materials and Methods, Figures, Data Availability Section)
New materials and reagents need to be available; do any restrictions apply?	Not Applicable	No newly created materials were used within this manuscript

Antibodies	Information included in the manuscript?	In which section is the information available? (Reagents and Tools Table, Materials and Methods, Figures, Data Availability Section)
For **antibodies** provide the following information:<br>- Commercial antibodies: RRID (if possible) or supplier name, catalogue number and or/clone number<br>- Non-commercial: RRID or citation	Yes	All informations including supplier names, clone numbers and fluochromes are provided in the Appendix

DNA and RNA sequences	Information included in the manuscript?	In which section is the information available? (Reagents and Tools Table, Materials and Methods, Figures, Data Availability Section)
**Short novel DNA or RNA including primers, probes**: provide the sequences.	Yes	All sequences of RNA primers are provided in the Materials and Methods section

Cell materials	Information included in the manuscript?	In which section is the information available? (Reagents and Tools Table, Materials and Methods, Figures, Data Availability Section)
**Cell lines:** Provide species information, strain. Provide accession number in repository **OR** supplier name, catalog number, clone number, and/**OR** RRID.	Yes	The information on human HEK cells is provided in the Materials and Methods section
**Primary cultures:** Provide species, strain, sex of origin, genetic modification status.	Yes	The information is porvided in the Materials and Methods section
Report if the cell lines were recently **authenticated** (e.g., by STR profiling) and tested for mycoplasma contamination.	Not Applicable	-

Experimental animals	Information included in the manuscript?	In which section is the information available? (Reagents and Tools Table, Materials and Methods, Figures, Data Availability Section)
**Laboratory animals or Model organisms:** Provide species, strain, sex, age, genetic modification status. Provide accession number in repository **OR** supplier name, catalog number, clone number, **OR** RRID.	Yes	All relevant information is provoded in the Materials and Methods section
**Animal observed in or captured from the field:** Provide species, sex, and age where possible.	Not Applicable	
Please detail **housing and husbandry conditions**.	Yes	Animal conditions are specified in the Materials and Methods section

Plants and microbes	Information included in the manuscript?	In which section is the information available? (Reagents and Tools Table, Materials and Methods, Figures, Data Availability Section)
**Plants:** provide species and strain, ecotype and cultivar where relevant, unique accession number if available, and source (including location for collected wild specimens).	Not Applicable	No plants were used in this study
**Microbes:** provide species and strain, unique accession number if available, and source.	Not Applicable	no microbes were used in this study

Human research participants	Information included in the manuscript?	In which section is the information available? (Reagents and Tools Table, Materials and Methods, Figures, Data Availability Section)
If collected and within the bounds of privacy constraints report on age, sex and gender or ethnicity for all study participants.	Yes	Patients characteristics such as Age, Sex and Disease entity are detailed in the Appendix (Supplementary Table 1)

Core facilities	Information included in the manuscript?	In which section is the information available? (Reagents and Tools Table, Materials and Methods, Figures, Data Availability Section)
If your work benefited from core facilities, was their service mentioned in the acknowledgments section?	Yes	Core Facilties are mentioned in the acknowledgements section

Design

Study protocol	Information included in the manuscript?	In which section is the information available? (Reagents and Tools Table, Materials and Methods, Figures, Data Availability Section)
If study protocol has been **pre-registered, provide DOI in the manuscript**. For clinical trials, provide the trial registration number **OR** cite DOI.	Not Applicable	the study was not pre-registerd as it is not a clinical study
Report the **clinical trial registration number** (at ClinicalTrials.gov or equivalent), where applicable.	Not Applicable	The the study is a basic research study and not a clinical study

Laboratory protocol	Information included in the manuscript?	In which section is the information available? (Reagents and Tools Table, Materials and Methods, Figures, Data Availability Section)
Provide DOI OR other citation details if **external detailed step-by-step protocols** are available.	Not Applicable	

Experimental study design and statistics	Information included in the manuscript?	In which section is the information available? (Reagents and Tools Table, Materials and Methods, Figures, Data Availability Section)
Include a statement about **sample size** estimate even if no statistical methods were used.	Yes	A statement about the sample size estimate for CyTOF analyses has been included in the methods section
Were any steps taken to minimize the effects of subjective bias when allocating animals/samples to treatment (e.g. **randomization procedure**)? If yes, have they been described?	Not Applicable	No randomization steps were performed
Include a statement about **blinding** even if no blinding was done.	Yes	All histologic analyses were performed in a blinded manner. This step has been described in the Materials and Methods section.
Describe **inclusion/exclusion criteria** if samples or animals were excluded from the analysis. Were the criteria pre-established?<br><br>If sample or data points were omitted from analysis, report if this was due to attrition or intentional exclusion and provide justification.	Not Applicable	No data points were omitted from analyses
For every figure, are **statistical tests** justified as appropriate? Do the data meet the assumptions of the tests (e.g., normal distribution)? Describe any methods used to assess it. Is there an estimate of variation within each group of data? Is the variance similar between the groups that are being statistically compared?	Yes	All statitsic tests are described in the Materials and Methods sections and the Figure legends

Sample definition and in-laboratory replication	Information included in the manuscript?	In which section is the information available? (Reagents and Tools Table, Materials and Methods, Figures, Data Availability Section)
In the figure legends: state number of times the experiment was **replicated** in laboratory.	Yes	Informations are provided in the figure legends
In the figure legends: define whether data describe **technical or biological replicates**.	Yes	Imformations are provided in the figure legends

Ethics

Ethics	Information included in the manuscript?	In which section is the information available? (Reagents and Tools Table, Materials and Methods, Figures, Data Availability Section)
Studies involving **human participants**: State details of **authority granting ethics approval** (IRB or equivalent committee(s), provide reference number for approval.	Yes	The reference number and the authoriy granting the ethics approval has been stated in the Materials and Methods section
Studies involving **human participants**: Include a statement confirming that **informed consent** was obtained from all subjects and that the experiments conformed to the principles set out in the WMA Declaration of Helsinki and the Department of Health and Human Services Belmont Report.	Yes	A statement has been included in to the Materials and Methods section
Studies involving **human participants:** For publication of **patient photos**, include a statement confirming that consent to publish was obtained.	Not Applicable	No photos of patients are included
Studies involving experimental **animals**: State details of **authority granting ethics approval** (IRB or equivalent committee(s), provide reference number for approval. Include a statement of compliance with ethical regulations.	Yes	Details are provided in the Materials and Methods section
Studies involving **specimen and field samples:** State if relevant **permits** obtained, provide details of authority approving study; if none were required, explain why.	Not Applicable	

Dual Use Research of Concern (DURC)	Information included in the manuscript?	In which section is the information available? (Reagents and Tools Table, Materials and Methods, Figures, Data Availability Section)
Could your study fall under dual use research restrictions? Please check biosecurity documents and list of **select agents and toxins** (CDC): https://www.selectagents.gov/sat/list.htm	Not Applicable	
If you used a select agent, is the security level of the lab appropriate and reported in the manuscript?	Not Applicable	
If a study is subject to dual use research of concern regulations, is the name of the **authority granting approval and reference number** for the regulatory approval provided in the manuscript?	Not Applicable	

Reporting

The MDAR framework recommends adoption of discipline-specific guidelines, established and endorsed through community initiatives. Journals have their own policy about requiring specific guidelines and recommendations to complement MDAR.

Adherence to community standards	Information included in the manuscript?	In which section is the information available? (Reagents and Tools Table, Materials and Methods, Figures, Data Availability Section)
State if relevant guidelines or checklists (e.g., **ICMJE, MIBBI, ARRIVE, PRISMA**) have been followed or provided.	Not Applicable	
For **tumor marker prognostic studies**, we recommend that you follow the **REMARK** reporting guidelines (see link list at top right). See author guidelines, under 'Reporting Guidelines'. Please confirm you have followed these guidelines.	Not Applicable	
For **phase II and III randomized controlled trials**, please refer to the **CONSORT** flow diagram (see link list at top right) and submit the CONSORT checklist (see link list at top right) with your submission. See author guidelines, under 'Reporting Guidelines'. Please confirm you have submitted this list.	Not Applicable	

Data Availability

Data availability	Information included in the manuscript?	In which section is the information available? (Reagents and Tools Table, Materials and Methods, Figures, Data Availability Section)
Have **primary datasets** been deposited according to the journal's guidelines (see 'Data Deposition' section) and the respective accession numbers provided in the Data Availability Section?	Yes	This study does not contain data that requires deposition in a public database. A statement has been provided in the Data Availability Section
Were **human clinical and genomic datasets** deposited in a public access-controlled repository in accordance to ethical obligations to the patients and to the applicable consent agreement?	Not Applicable	No human clinical or genomic data sets were deposited
Are **computational models** that are central and integral to a study available without restrictions in a machine-readable form? Were the relevant accession numbers or links provided?	Not Applicable	
If publicly available data were reused, provide the respective **data citations in the reference list**.	Yes	Citations for publicly available data sets are provided in the reference list
